# Population genomic data reveal genes related to important traits of quail

**DOI:** 10.1093/gigascience/giy049

**Published:** 2018-05-11

**Authors:** Yan Wu, Yaolei Zhang, Zhuocheng Hou, Guangyi Fan, Jinsong Pi, Shuai Sun, Jiang Chen, Huaqiao Liu, Xiao Du, Jie Shen, Gang Hu, Wenbin Chen, Ailuan Pan, Pingping Yin, Xiaoli Chen, Yuejin Pu, He Zhang, Zhenhua Liang, Jianbo Jian, Hao Zhang, Bin Wu, Jing Sun, Jianwei Chen, Hu Tao, Ting Yang, Hongwei Xiao, Huan Yang, Chuanwei Zheng, Mingzhou Bai, Xiaodong Fang, David W Burt, Wen Wang, Qingyi Li, Xun Xu, Chengfeng Li, Huanming Yang, Jian Wang, Ning Yang, Xin Liu, Jinping Du

**Affiliations:** 1 Institute of Animal Husbandry and Veterinary, Hubei Academy of Agricultural Science, Wuhan 430064, China; 2 BGI-Shenzhen, Shenzhen 518083, China; 3 Key Laboratory of Animal Embryo Engineering and Molecular Breeding of Hubei Province,Wuhan 430064, China; 4 National Engineering Laboratory for Animal Breeding and MOA Key Laboratory of Animal Genetics and Breeding, China; Agricultural University, Beijing 100193, China; 5 BGI-Qingdao, BGI-Shenzhen, Qingdao, 266555, China; 6 Hubei Innovation Center of Agricultural Science and Technology, Wuhan, Hubei, 430064, China; 7 Hubei Shendan Healthy Food Co., Ltd., Wuhan 430206, China; 8 State Key Laboratory of Quality Research in Chinese Medicine and Institute of Chinese Medical Sciences, Macao, China; 9 The Roslin Institute and Royal (Dick) School of Veterinary Studies, University of Edinburgh, Midlothian EH25 9RG, UK; 10 Kunming Institute of Zoology, Chinese Academy of Sciences (CAS), Kunming, China; 11 China National GeneBank-Shenzhen, BGI-Shenzhen, Shenzhen 518083, China; 12 James D. Watson Institute of Genome Sciences, Hangzhou 310058, China

**Keywords:** Japanese quail, genome assembly, early maturation, phylogeny, resequencing, plumage color, quail breeding

## Abstract

**Background:**

Japanese quail (*Coturnix japonica*), a recently domesticated poultry species, is important not only as an agricultural product, but also as a model bird species for genetic research. However, most of the biological questions concerning genomics, phylogenetics, and genetics of some important economic traits have not been answered. It is thus necessary to complete a high-quality genome sequence as well as a series of comparative genomics, evolution, and functional studies.

**Results:**

Here, we present a quail genome assembly spanning 1.04 Gb with 86.63% of sequences anchored to 30 chromosomes (28 autosomes and 2 sex chromosomes Z/W). Our genomic data have resolved the long-term debate of phylogeny among Perdicinae (Japanese quail), Meleagridinae (turkey), and Phasianinae (chicken). Comparative genomics and functional genomic data found that four candidate genes involved in early maturation had experienced positive selection, and one of them encodes follicle stimulating hormone beta (*FSHβ*), which is correlated with different *FSHβ* levels in quail and chicken. We re-sequenced 31 quails (10 wild, 11 egg-type, and 10 meat-type) and identified 18 and 26 candidate selective sweep regions in the egg-type and meat-type lines, respectively. That only one of them is shared between egg-type and meat-type lines suggests that they were subject to an independent selection. We also detected a haplotype on chromosome Z, which was closely linked with maroon/yellow plumage in quail using population resequencing and a genome-wide association study. This haplotype block will be useful for quail breeding programs.

**Conclusions:**

This study provided a high-quality quail reference genome, identified quail-specific genes, and resolved quail phylogeny. We have identified genes related to quail early maturation and a marker for plumage color, which is significant for quail breeding. These results will facilitate biological discovery in quails and help us elucidate the evolutionary processes within the Phasianidae family.

## Introduction

Most of the poultry, eggs and meat products in the world come from species that are members of the Phasianidae family, including chicken (*Gallus gallus*), turkey (*Meleagris gallopavo*), and Japanese quail (*Coturnix japonica*), within the order Galliformes. The genomes of the two widely domesticated avian species, chicken and turkey, have already been sequenced [1, 2]. Accordingly, the first quail draft genome sequence was reported with the N50 contig length of 1.5 kb by Tokyo University of Agriculture in 2013. Subsequently, the same group developed an improved draft and extended the N50 contig length to 32 kb (NCBI BioSample: SAMD00009971) [3]. Recently, another chromosome-level draft genome for the Japanese quail was published by using the quail inbred line, Cons DD (INRA) (NCBI BioSample: SAMN03989050). However, with these reference quail genome assemblies, most biological questions involving genomics and phylogenetics are still unresolved.

The Phasianidae family has its origin about 30–46 million years ago (MYA) [1, 4–7]. Even though high degrees of conservation of synteny and chromosome homology have been observed between quail and chicken [8, 9], these species display a great diversity of phenotypes among the three widely used domesticated birds. Japanese quail reach sexual maturity at 5–6 weeks of age [10], while chicken and turkey reach this stage in about 18–22 weeks [11]. Body mass at the maturity stage of meat-type quail is about 10% that of broiler chicken and 2.5% that of turkey [11], yet quail have the fastest growth rate of all species in the Phasianidae family [12, 13]. Furthermore, female quails generally present a heavier body weight than males [14], while the reverse is true both in chickens and turkeys [15]. In addition, there are very distinct differences between subpopulations of quail, even though quail branched off from the Phasianidae family fairly recently [16]. According to historical records, the domestication of Japanese quail began in the 11^th^ century and was initially based on birds selected for their crowing abilities [16]. However, the resulting domestic strains, which were selected for commercial egg and meat production, were improved only from the 1910s [17]. Today, the domestic quail differs from the wild population in many traits, such as variations in plumage color, increased body size, acceleration of sexual maturity, lengthening of the reproductive phase, and the disappearance of migratory characteristics [18]. Because of the important roles of plumage color in signaling, mate choice, and evolution, mapping the gene conferring sex-linked plumage color is significant for commercial breeding in quail [13, 17–20]. Additionally, it has been established that the quail has an advantage over the chicken concerning reproduction interval and space requirements, so the quail is also considered to be an excellent avian model for embryonic development, reproduction, sexual differentiation, environmental toxicant indication, and disease resistance [19–27].

The phylogeny and genetic relationships for some of the key avian model systems (e.g., chicken, turkey, and Japanese quail) are not well resolved [28]. Even though a preliminary understanding of Phasianidae phylogenies has been gained via archaeologic and demographic techniques, the evolution of the Phasianidae family is still under debate. Now that sequencing data are available, several conclusions that were drawn about Phasianidae evolution based on fossil evidence are inconsistent with results from mtDNA analysis, mainly those concerning phylogenies and divergence time. We believe this is likely due to the rapid diversification of the Phasianidae family [5, 6, 29]. Because of this rapid diversification (observed during the Eocene), as well as the short divergence times within some lineages, the phylogenies of galliform birds (including chicken, turkey, and quail) usually have low bootstrap support values [30]. Phylogenies based on the *CR1* retrotransposon support the hypothesis that quail and turkey are more closely related than quail and chicken, while those based on mitochondrial genome data support the hypothesis that quail is more closely related to chicken than turkey [30]. However, phylogenies of the Perdicinae (Japanese quail), Meleagridinae (turkey), and Phasianinae (chicken) subfamilies are still not clear based only on information that is inferred from either current fossil evidence or partial genome data. Therefore, comparing these species at a whole-genome level will enable us to better understand the process of speciation of Phasianidae family. A high-quality genome assembly of the quail with population genomic data of the quail is necessary to address these questions [30, 31].

Here, we report the completion of an additional genome assembly of Japanese quail (*Coturnix japonica*), as well as the resequencing of 71 quail, both domestic and wild, and we describe experimental results concerning several important quail traits. These results were then used to characterize the mechanisms of early sexual maturity in quail, resolve the phylogeny and divergence time of the Phasianidae family, and detect footprints of artificial selection in the quail genome. We have also identified the genetic basis for a plumage color marker that is widely used in quail breeding. These results will facilitate biological discovery, the improvement of quail for meat and egg production, and help resolve the basis of evolution within the Phasianidae family.

## Results

### Characteristics of the quail genome

**Figure 1:**
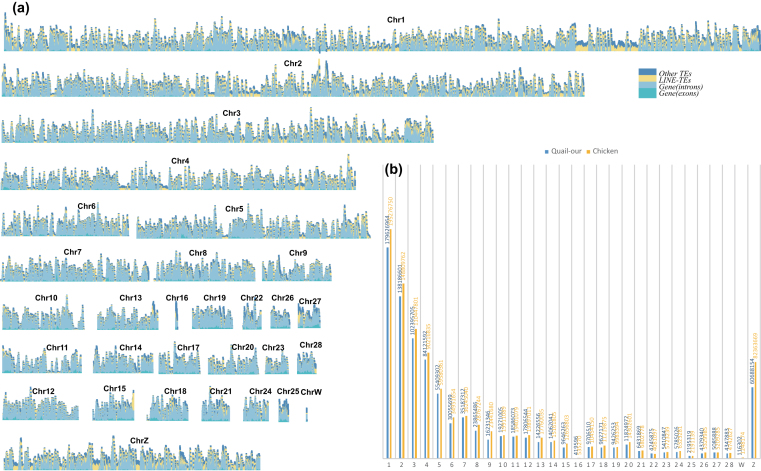
Chromosomes of quail. **A**) Gene and TE density of each quail chromosome. **B**) Comparison of the chromosome lengths of quail and chicken.

High-quality genomic DNA extracted from a female quail (Shendan quail 1) was used to generate 262 Gb of sequence (approximately 238-fold coverage of the whole genome) (Table S1) using the Illumina HiSeq 2000 platform. The genome assembled using SOAP*denovo2* [32] spans 1.04 Gb(93.9% of the estimated genome size for quail; Fig. S1) with contig N50 and scaffold N50 lengths of 27.9 kb and 1.8 Mb, respectively (Table S2). About 901 Mb of sequence (86.6% of the whole genome) was anchored to 30 chromosomes using a previously reported genetic linkage map [33] (Fig. 1a). We aligned these chromosomes back to a previously reported quail genome assembly (NCBI BioSample: SAMN03989050) and found that the two genomes had a high degree (92.14%) of consistency (Fig. S2). The length and GC distribution of chromosomes are also highly consistent between quail and chicken genome sequences (Figs 1b and S3). To evaluate the quality of the assembled quail genome, seven fosmid clones, each about 40 kb in length, were sequenced and mapped back to the quail genome assembly with a high coverage ratio (>92% for all, and six of seven fosmids >98.4%) (Table S3 and Fig. S4). To assess the integrity of protein-coding genes in the quail genome assembly, all transcripts assembled from RNA-Seq data sampled from the hypothalamus and ovary of three stages of quail maturity (before-laying [BL], laying [L], and peak-laying [LP]) were mapped to the assembled genome (Table S4), and ∼96.33% of total complete BUSCO genes can be identified in the genome (Table S5). These measures demonstrated the high quality of our genome assembly, allowing it to serve as a reference genome for subsequent quail genome research.

Genome annotation of our quail genome assembly included transposable elements (TEs) and protein-coding genes. TEs comprise 12.4% of the genome, which is a little higher than the average value in the class Aves [34], and 9.4% of the genome consists of long interspersed nuclear elements (Fig. 1a and Table S6). Gene prediction was performed using a combination of several methods, including homology searches, *ab initio* prediction, and RNA-Seq data. The merged results revealed evidence for 16,210 protein-coding genes in the quail genome (Table S7), and 15,972 (∼98.5%) genes were supported by known protein-coding entries in at least one of the following databases: Swiss-Prot, InterPro, Gene Ontology (GO), TrEMBL, or KEGG (Table S8).

### Evolutionary relationships within the Phasianidae family

**Figure 2:**
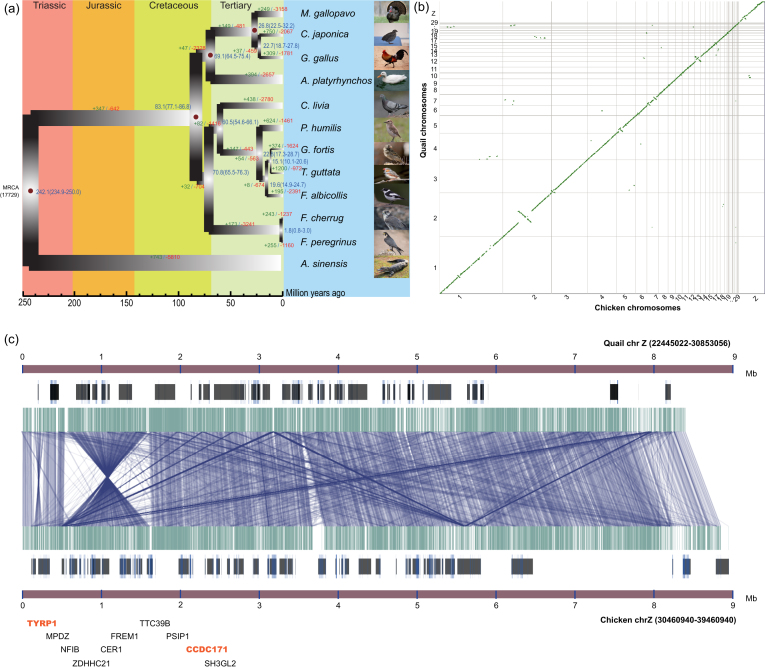
Comparative evolutionary analysis of 12 avian species. **A**) The phylogenetic tree of *Coturnix japonica* (quail), *Gallus gallus* (chicken), *Anas platyrhynchos* (duck), *Columba livia* (pigeon), *Falco cherrug* (Saker falcon), *Falco peregrinus* (Peregrine falcon), *Ficedula albicollis* (collared flycatcher), *Geospiza fortis* (medium ground finch), *Meleagris gallopavo* (turkey), *Pseudopodoces humilis* (ground tit), *and Taeniopygia guttata* (zebra finch), with *Alligator sinensis* (Chinese alligator) as an outgroup. **B**) Syntenic relationships between the quail and chicken genomes. **C**) An inversion detected in chromosome Z between quail and chicken.

To resolve the phylogenetic debate in the Phasianidae family and establish the phylogenetic position of the quail in relation to other avian species, we defined 12,178 gene families in quail and 10 other representative bird species, with *Alligator sinensis* (Chinese alligator) serving as an outgroup (Fig. S5). A total 9,631 gene families were shared among four species (*Taeniopygia guttata*, *Pseudopodoces humilis*, *Gallus gallus*, and *Coturnix japonica*; Fig. S6), and 4,393 single-copy orthologs were shared among 12 species. These single-copy orthologous genes were used to construct a phylogenetic tree (Fig. 2a and Fig. S7) and estimate the divergence times of the quail from other birds. Quail was mapped to the evolutionary branch containing domesticated poultry and was most closely related to the chicken lineage, sharing a common ancestor about 22.2 MYA (Fig. 2a). We used our genome-wide comparative data to estimate the divergence time of Galliformes and Anseriformes at 69.1 (64.5–75.4) MYA. Our results, therefore, fully support a closer relationship between quail and chicken than between quail and turkey. The phylogeny we generated implied that the quail and chicken genomes likely share significant similarities, which makes further comparison of their genomes intriguing.

In total, 95.5% of quail genome sequences occurred in blocks colinear with those in chicken (Fig. 2b and Table S9). However, a total 131 large inversions (block length >5 kb) between quail and chicken chromosomes were also identified and most of these were located on chromosomes 1 (24 breakpoints) and Z (24 breakpoints) (Table S10). Next, to investigate the nature of chromosome breaks that differentiate the quail and chicken genomes and to associate these differences with possible phenotypic changes during their divergence, we tested for gene set enrichments at the boundaries. We identified 433 genes located within the 1-kb regions flanking the breakpoints of these inversions (Table S10). We tested for gene function enrichment within these inversions and searched for candidate mutations that might contribute to specific phenotypes in quail compared with chicken. The results of GO term enrichment analysis of these genes revealed the terms GO:0005882: intermediate filament (*P* = 1.53e-05) and GO:0005200: structural constituent of cytoskeleton (*P* = 0.00029) were significantly enriched (Figs. S8 and S9). In particular, a gene encoding tyrosinase-related protein 1 (*TYRP1*) was identified in the flanking region of an inversion on chromosome Z, which has been reported as a candidate locus for the recessive, sex-linked roux (br(r)) phenotype in Japanese quail [35] (Fig. 2c).

### Nucleotide diversity and population structure

To obtain a comprehensive understanding of genetic diversity in a quail population, we collected a total 31 samples for genome re-sequencing, which includes 10 quails from wild population, 11 egg-type quails, and 10 meat-type quails from domesticated subpopulations (Table S11). We sequenced these samples with an average read depth of 19X and mapped the reads to our reference genome (Shendan quail 1) with average coverage of 96.72% (Table S12). Eventually, we identified a total 43,108,723 biallelic SNPs (single nucleotide polymorphisms) among the 31 re-sequenced samples, which included 21,597,713; 18,966,964; and 35,182,459 SNPs in wild quails, egg-type quails, and meat-type quails, respectively (Table 1). Of the ∼43 M high-quality SNPs, only 649,024 SNPs were located in exonic regions, yet there were 17,227,986 SNPs in intron regions. Thus, the ratio of the number of SNPs in exons and introns was 3.77e-3, roughly equivalent to that of turkey (4.30e-2) [36] and chicken (3.50e-2) [37]. Accordingly, we found that the non-synonymous SNPs (N) and the synonymous SNPs (S) in quail were 202,742 and 446,282, respectively, with a ratio of N/S of 0.454, which does not show a significant difference to chicken (0.41) or turkey (0.45). To evaluate the genetic diversity of our quail population, we calculated two common summary statistics across the whole genome, *π* and *θ*_w_ [38, 39], by using 100-kb sliding overlapped window with step length of 10 kb (Tables 1 and S13). Compared with the two domesticated subpopulations, the wild quail population displayed both higher *π* and higher *θ*_w_ on autosomes, indicating greater genetic diversity in the wild population. The same pattern was found on chromosome Z. The genetic diversity on chromosome Z was reduced compared with the autosomes in all three populations, a phenomenon that has been observed in a variety of other ZW system studies [40, 41]. The fact that sex chromosomes and autosomes differ in their effective population size, mutation characteristics, and demography contributes to the differential genetic diversity within the genome [42].

**Table 1:** Statistics of SNPs in different genome regions of wild and domesticated quails’ subpopulations

Region	Subpopulation	Autosomes				Chromosome Z			
		# of SNPs	*π* (10^-3^)	*θ*_w_ (10^-3^)	Tajima's *D*	# of SNPs	*π* (10^-3^)	*θ*_w_ (10^-3^)	Tajima's *D*
Exon^a^	Wild	520,747	7.324	8.979	−0.502	13,963	5.191	5.940	−0.309
	Egg-type	320,629	7.206	6.449	0.256	7201	5.448	4.479	0.425
	Meat-type	282,019	7.152	6.238	0.331	6967	5.120	4.549	0.275
Intron^a^	Wild	13,632,632	11.868	14.302	−0.548	555,643	8.100	9.197	−0.354
	Egg-type	8468,826	10.735	9.668	0.270	285,930	7.934	6.483	0.489
	Meat-type	7418,909	10.167	9.005	0.299	278,361	8.546	7.482	0.365
All regions^a^	Wild	33,744,246	8.679	10.709	−0.763	1438,213	5.813	6.683	−0.628
	Egg-type	20,848,043	7.338	6.617	0.467	749,670	4.031	3.389	0.666
	Meat-type	18,250,531	6.901	6.052	0.600	716,433	3.843	3.328	0.593

a All regions refers to the total genome regions; exon and intron refer to exon and intron regions, respectively.

**Figure 3:**
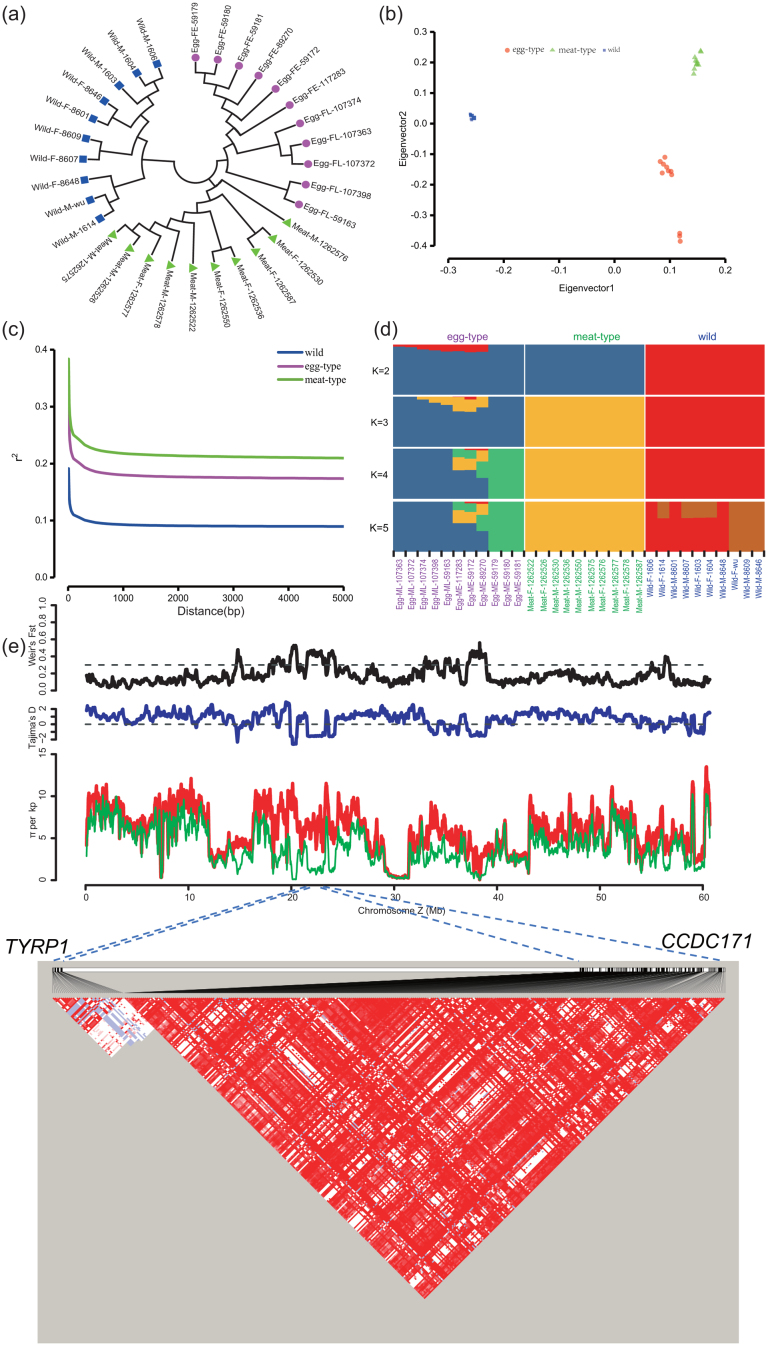
Analyses of the phylogenetic relationships, population structure, LD decay, and genetic diversity between wild and domesticated quail. **A**) Evolutionary history was inferred using the neighbor-joining method in MEGA 6.0. **B**) PCA of wild quail and domesticated quail. **C**) LD decay curves were estimated by squared pairwise correlations of alleles against physical distance in wild quail, egg-type quail, and meat-type quail, respectively. **D**) Population structure analysis with the maximum likelihood score for the model *K* = 2. **E**) Nucleotide diversity between wild quail and egg-type quail across chromosome Z. Both the wild quail (red line) and the egg-type quail (green line) showed difference of diversity on chromosome Z. Plotting of Tajima's *D* for the egg-type group (blue line) in a 100-kb sliding window in 10-kb steps revealed the selective signal on chromosome Z. Likewise, plotting Weir's *F*_st_ (black line) on chromosome Z indicates the level of differentiation between the wild group and the egg-type group. Both *CCDC171* and *TYRP1* genes were located within a selective sweep region (from ∼21.5 Mb to 23.2 Mb), in which the positive signal was detected in the egg-type group. However, they exhibited a weak linkage due to the location on the different haplotype blocks.

To investigate the phylogenetic relationships and population structure among the 31 quail samples (Table S11), we constructed a neighbor-joining tree by using pairwise genetic distance matrix (Fig. 3a) and performed principal component analysis (PCA) based on the variance-standardized genotype relationship matrix (Fig. 3b). The neighbor-joining tree showed that our samples could be divided into two major clusters, corresponding to wild quails and domesticated quails, with a further subdivision of domesticated quails into egg-type quails and meat-type quails. This pattern was further confirmed by the PCA results. Specifically, the first principal component (PC1) in PCA successfully separated the wild from the domesticated populations, and the second principal component (PC2) separated the egg-type and meat-type quails (Fig. 3b). To better estimate the ancestral component in our quail populations, we adopted likelihood models embedded in structure by using ADMIXTURE [43]. The initialization of population number (*K*) was tried from 2 to 5, and the minimum estimated cross-validation error occurred with *K* = 2 (Figs. 3d and S10). These results suggest that there was a distinct background between the wild population and domesticated population, similar to the results observed in NJ tree and PCA. The likelihood model based on *K* = 2 grouped the three quail populations into two genetic clusters (Fig. 3d): one that includes the wild quails and another that includes the domesticated quails. Considering the physiological and ethological characteristics of the quails investigated, we would have preferred to divide the 31 samples into three populations (egg-type, meat-type, and wild quail). *K* = 3 actually provides strong support for this scenario. This model groups the egg, meat, and wild quails into three distinct genetic clusters (Fig. 3d), and the slight mixing shown between the egg- and meat-types could also explain the spread along the PC2 axis in the PCA plot. To characterize linkage disequilibrium (LD) blocks in wild and domesticated quails, we estimated the squared correlations (*r*^2^) of pairwise SNPs with sliding window lengths from 1 to 50 kb. LD decayed to one-half of its maximum within a window length of ∼20 bp for wild quail, ∼100 bp for egg-type quail, and ∼230 bp for meat-type quail, respectively (Fig. 3c). Such a rapid decay of LD in each population might be due to the high density of SNPs in the quail genome (one SNP in every ∼20 bases, on average) and a high degree of recombination within the quail genome. Similarly, other studies involving the population structure of Aves animals also revealed the low level of LD corresponding to the open genome and fluid genomic background in the bird population, which could facilitate adaptive variation [44, 45].

### Signals of selection across the quail genome

Due to the low level of population divergence in our wild quails, any evidence of selective sweeps in short genomic regions could be masked in domesticated populations. Thus, we sought to detect the large-scale regions in the whole genome that may have been subjected to successive selective sweeps between the domesticated and wild populations by using a 100-kb overlapping sliding window in 10-kb steps. The reduction of diversity (ROD), defined as ROD = 1- *π*_domesticated_/*π*_wild_, was introduced to measure the loss of diversity in the domesticated population compared with the wild population. Additionally, to avoid ROD being excessively affected by diversity in the wild population, we also added a significantly negative Tajima's *D* (*D* < -2) in the domesticated population as a parallel criterion, which could represent a recent selective sweep or population expansion following a strong bottleneck [46–48]. From the comparison between egg-type quails and wild quails, we identified a total of 18 large-scale regions of selection sweep with the spans all >100 kb, where the sliding windows presented high ROD values and a small Tajima's *D* in the 1% tail of the distribution (Fig. S11 and Table S14). The fixation index (*F*_ST_), a measure of population divergence due to genetic structure, was substantially used as an additional condition to infer selection sweep at a high level (*F*_ST_ > 0.3). We identified 18 selective sweep regions with a total length of 7.9 Mb between egg-type quail and wild quail. Interestingly, we observed the longest 1.8-Mb sweep region was located on chromosome Z, and the length of sweep regions on chromosome Z was up to 5.57 Mb (Fig. 3e). Furthermore, we noticed a gene, *CCDC171*, which is significantly associated with quail plumage color, was just located on the longest region and included in a 182-kb haplotype block (*see the association study described below*). This may indicate that positive selection for plumage color might have resulted in a strong selective sweep on chromosome Z in egg-type quail. A total of 88 genes involving these selective sweeps has been annotated in GO terms. By using the Kyoto Encyclopedia of Genes and Genomes (KEGG) database, we noticed that some of these genes possibly played a role in sex hormones [49], embryo development [50, 51], increase of egg weight [52 ], and plumage color [53] (Table S15). Thus, we surmise that these important traits in egg-type quail might have suffered stronger artificial selection, leading to many large-scale selection sweeps on chromosome Z. Similarly, we also identified 26 large regions of selective sweep between meat-type quail and wild quail on chromosomes 1, 2, 13, and Z (Fig. S11 and Table S16). The total length of selective sweeps in meat-type quail was estimated at ∼8.77 Mb, and the longest span could be as long as 1.2 Mb on chromosome 2. That led us to infer that breed improvement for meat-type quail was likely not restricted to chromosome Z, but could affect many other genomic regions. Subsequently, we annotated the biological functions of 118 genes in those meat-type quail sweep regions (Table S17). Although most genes that correspond to relevant traits had not been previously verified in meat-type quail, a more detailed investigation of these candidate genes could further our understanding of the domestication process in future studies. However, it is worth mentioning that there was hardly any selective sweep shared between wild quail and egg-type quail or between wild quail and meat-type quail, except for a 130-kb region (40.15–40.28 Mb) on chromosome 4, which contains one annotated gene: COT02188 (vascular endothelial growth factor C/D) (Tables S15 and S17). With such differentiated large selective sweeps, we speculate that egg-type quail and meat-type quail might have undergone selection independently subsequent to their initial domestication in the early 20^th^ century [17].

Despite that selective sweep gave us a new insight into evidence of domestication process, we also found that a number of functional genes dispersed across the whole genome were contributed to the divergent traits observed when comparing domesticated quails and wild quails. Thus, we picked up the highly differentiated non-synonymous SNPs with *F*ST values >0.5 that show high population differentiation in the between wild and egg-type population. Specifically, we found 1,943 highly differentiated non-synonymous SNPs in 1,213 genes between egg-type and wild quail and 3,508 highly differentiated non-synonymous SNPs in 2,032 genes between meat-type and wild quail. We then performed functional enrichment analysis of KEGG pathways using these sets of genes for both egg-type quail and meat-type quail and found that 8 of the top 20 most enriched pathways were shared by egg-type and meat-type quail (Figs S12 and S13). Of these, the most significantly enriched pathway was ECM-receptor interaction (ko04512), an activity that plays important roles in both the integrity of tissue and intramuscular fat metabolism [54]. Other functional pathways, specific to either egg-type or meat-type quail, were not significantly enriched, indicating that artificial selection targeting these traits was not strong. In summary, the independent selection for similar traits in the two domesticated subpopulations suggests that the phenotypic difference between egg-type and meat-type quail is not that significant, which is consistent with the observation during the population stratification analysis that the optimal number of populations was two rather than three.

### Genes involved in early sexual maturity

**Figure 4:**
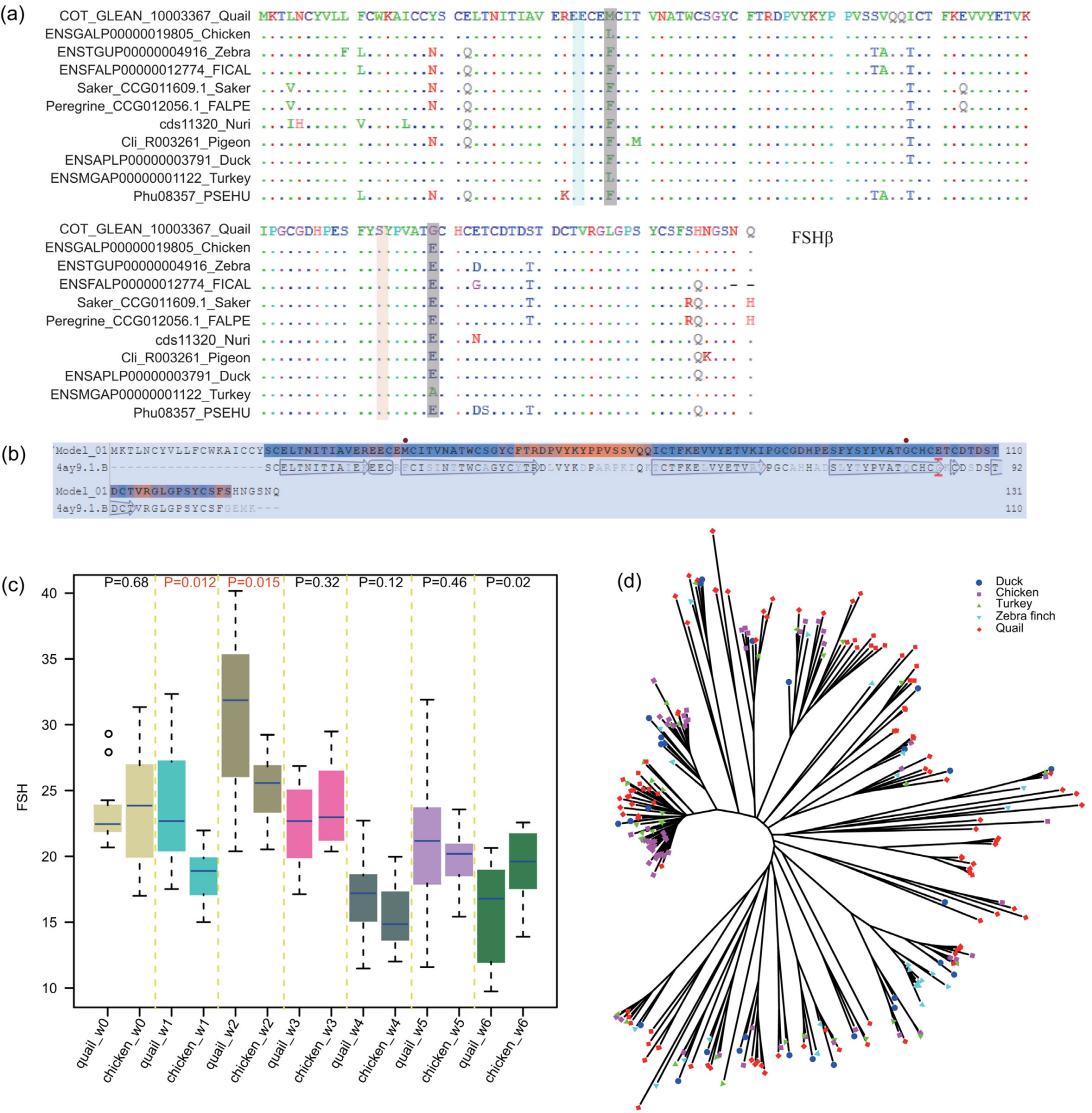
Genes related to early sexual maturity and immune system function in quail and another avian species. **A**) Positions of amino acids under positive selection in the FSHβ protein. **B**) Location of two amino acids under positive selection on the predicted 3D structure of the FSHβ protein. **C**) Circulating FSHβ levels in blood during early development stages of quail and chicken for 6 weeks. **D**) Phylogenetic tree of Immunoglobulin-like and Immunoglobulin subtype proteins of quail, chicken, duck, turkey, and zebra finch.

To explore the biological mechanism of very early sexual maturity in quail, genes were traced from both gene family evolution and positive selection events in the quail lineage. We found that several gene families have expanded in the quail genome compared with those of other domesticated birds. These families include those encoding gonadotropin-releasing hormone 1 (*GnRH1*, Fig. S14), the lysophospholipase catalytic domain and phospholipase A2 (Table S18). Moreover, four positively selected genes (PSGs) were detected in the quail lineage, and the proteins encoded by these genes (*FSHβ*, *PLCB4*, *ITPR1*, and *PLA2G4*) are involved in the *GnRH* signaling pathway. *FSHβ* protein is a glycoprotein polypeptide hormone that, in conjunction with luteinizing hormone, contributes to growth and reproduction [55]. Transcription of the *FSHβ* gene is the rate-limiting step in hormone synthesis [56] that is required for ovarian folliculogenesis in females and for spermatogenesis in males, in conjunction with testosterone [57]. We identified two amino acids in the quail *FSHβ* protein at position 37 (M→F/L) and position 99 (G→E/A) that were predicted to be under positive selection (Fig. 4a). We used ELISA to measure the level of *FSHβ* protein during early developmental stages and found that the level of *FSHβ* in early-maturing quail blood is consistently higher than that in chicken (*P* <0.05) (Fig. 4c). We used SWISS-MODEL to model the structure of quail *FSHβ* using the Follitropin subunit beta (4ay9.1.B) protein [58] as a template. These two amino acid substitutions were mapped to the 3D protein structure and were located near the *β*-pleated sheet that interacts with the *FSH* receptor (Fig. 4b). *PLCB4*, *ITPR1*, and *PLA2G4* (Figs S15–17), together with other molecules (e.g., inositol 1,4,5-trisphosphate, diacylglycerol, and protein kinase C) stimulate release of gonadotropins including luteinizing hormone and FSH [59, 60]. Gene expansions in the *GnRH* families, and also PSGs in the *GnRH* signaling pathway, are likely to be involved in the acceleration of growth and sexual maturity in the quail. Subsequently, we scanned both synonymous and non-synonymous SNPs found in the coding sequence (CDS) of these four genes in the 31 wild and domestic individuals and found that all but four of the 83 SNPs were synonymous substitutions. However, all the divergent alleles of SNP loci did not generally segregate according to the three subpopulations, and the domestic and wild quails presented no large-scale selective sweeps around these genes.

### Gene families related to immune system function

We identified a total of 1,587 immune response-related genes in quail (Table S19) by aligning the entire predicted gene set of quail against 2,257 genes that have been annotated with roles in the innate immune responses of *Homo sapiens*, *Mus musculus*, or *Bos taurus* at InnateDB or GO databases. Compared with chicken, turkey, and duck, several expanded gene families were identified in quail. These included *Klf4*, which is indispensable for differentiation of inflammatory monocytes [61] and negative regulation of innate immune response against several viruses in human embryonic kidney 293 cells [62] (22 copies in quail, 17 in chicken, 16 in turkey, and 12 in duck); *Foxa2*, which regulates genetic programs that influence pulmonary inflammation mediated by Th2 cells [63] (13 copies in quail, 7 in chicken, 4 in turkey, and 5 in duck); and *ITCH*, which acts in T-helper cell differentiation and T-cell activation and tolerance [64] (7 copies in quail, 3 in chicken, 4 in turkey, and 2 in duck). Moreover, we focused on the number of “Immunoglobulin subtype” genes and found that there are 109 in quail, while chicken, duck, zebra finch (*Taeniopygia* g*uttata* ), and turkey each had 62 or fewer immunoglobulin-related genes (62 copies in chicken, 29 in duck, 29 in zebra finch, and 34 in turkey; Fig. 4d). We detected 69 genes encoding a putative “Reverse transcriptase or Reverse transcriptase domain” in quail but found only two and four such genes in chicken and turkey, respectively (Fig. S18). These domains are signatures of retroviruses integrated into the host genome. Next we compared the *MHC-B* region between quail and chicken (Fig. S19) and found that there was an inversion including the genes encoding the proteins *TAP1* and *TAP2*, which transport peptides from the cytosol into the endoplasmic reticulum to bind *MHC* class I molecules that are being synthesized [65], and *BFIV21*, which encodes a protein that presents antigens such as the avian leukosis virus [66]. We also found four copies of the *BLEC2* (C-type lectin-like NK cell receptor) gene in quail, but only one in chicken. However, several other *MHC* genes (e.g., *KIFC1*, *V-BG1*, and *BG2*) were not detected in the quail genome. A better understanding of these immune-related genetic changes will help us characterize the immune response in quail and facilitate the development of targeted vaccines for quail.

### Genome-wide association analysis of plumage color

To identify sexed-linked genes conferring plumage color, we bred a set of egg-type quails with maroon or yellow plumage, a trait that has been confirmed to have sex-linked inheritance in quail and is consistent with a Mendelian segregation ratio according to our previous investigation (*see more details in Method*s). From this set we sampled 40 quails, including 20 male and 20 female quails, and re-sequenced their genomes for case control analysis (Table S20). We identified ∼20 Mbi-allelic SNPs in these 40 quails at a sequencing depth of 20–30×. After controlling for SNP quality and redundant LD (*see more details in Methods*), a final total of 864,292 SNPs was retained for subsequent analyses. A genome similarity test of the 40 quail samples was conducted using high-quality SNPs, and we found that similarities between any pair of individuals ranged from 70.4% to 86.5%, indicating relatively high homology between them.

**Figure 5:**
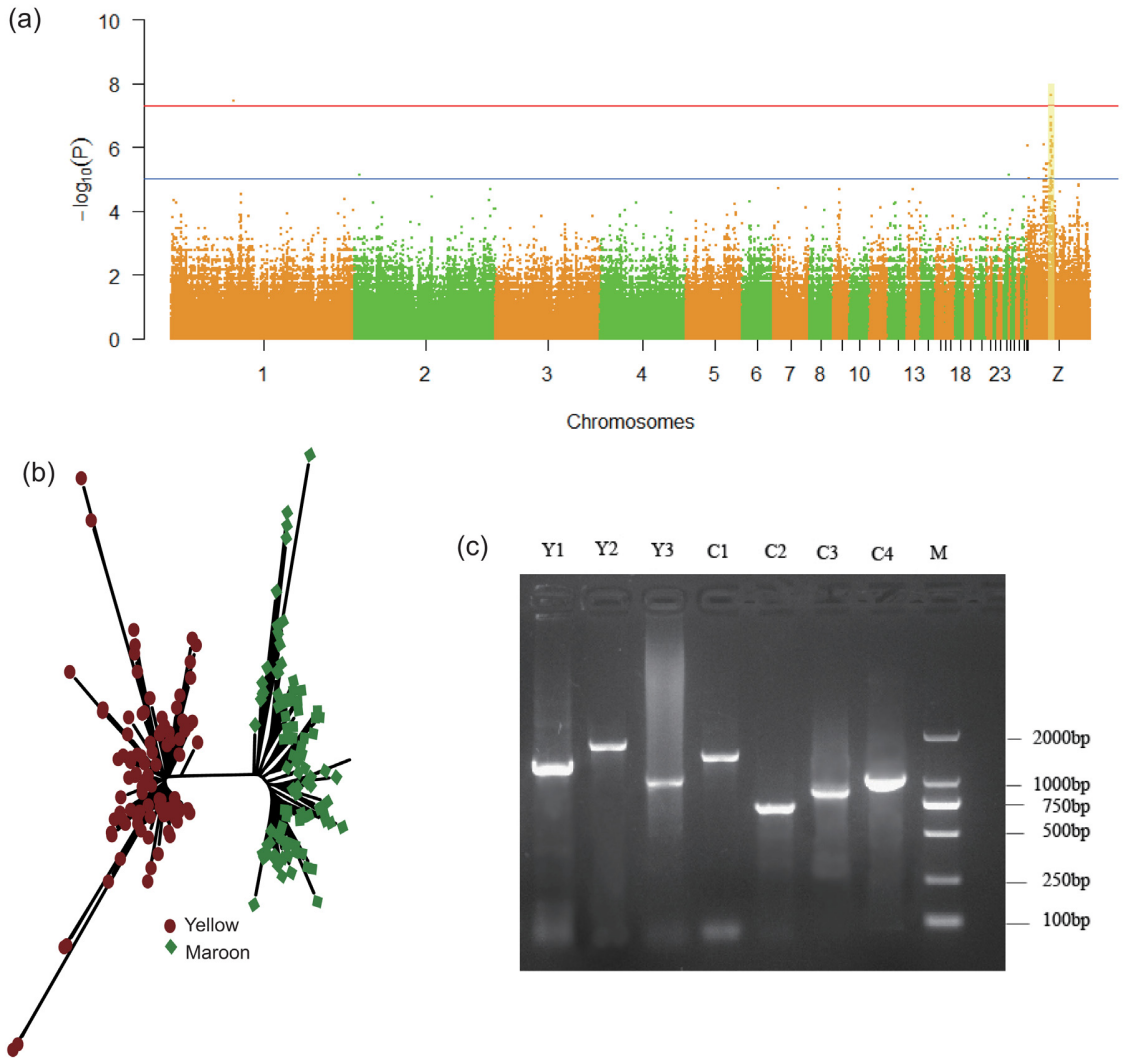
GWAS analysis of quail plumage color. **a**) Manhattan plot of each chromosome showing the GWAS results for quail plumage color. **b**) Validation of eight candidate SNPs in 200 random individual quail. **c)** The clones of *CCDC171* gene transcripts from quail with “yellow” and “maroon” plumage.

Due to the relatively close relationships between the 40 samples, the effect of relativeness matrix affecting the variance of plumage color was considered as the covariance. We assessed the relationships matrix of the 40 samples by using GEMMA v0.94 [67] and adopted the linear mixed model for association analysis. By Bonferroni correction, the association analysis showed that two SNPs, 61102026 on chromosome 1 and 23173971 on chromosome Z, had significant effects on plumage color (adjusted *P* = 0.028 and *P* = 0.019, respectively) (Figs 5a and S20 and Table S21). However, unlike the locus on chromosome 1, SNPs on chromosome Z near 23173971 showed a continuous peak on the Manhattan plot. In our previous analysis of plumage color heredity, we suggested that the locus on chromosome Z was most likely associated with plumage color. In a confirmation study, we added the 21 previously re-sequenced quails with “maroon” plumage (including 10 wild quails and 11 egg-type quails, Table S11) to rerun the association analysis for the two loci. The locus on chromosome Z was found to be more significantly associated with plumage color than before (adjusted *P* = 0.015). Conversely, there was no significant signal on chromosome 1 (adjusted *P*-value for SNP 61102026 fell to 1.000). Subsequently, the SNP 23173971 on chromosome Z, we found, was located closely to gene Coiled-coil domain-containing 171 (*CCDC171*) with a length of 135 kb. Therefore, we chose SNP 23173971 on chromosome Z as the index SNP within the region of 200 kb for conditional haplotype-based association testing. Consequently, 47 SNPs with *r*^2^ >0.7 and adjusted *P*>0.01 were clumped together for association testing. Using 5,000 permutations, a highly linked haplotype with a range of 182 kb could significantly explain the maroon/yellow variation (*χ*^2^ = 37.7, *P* = 8.563e-06). The well-known *TYRP1* gene that confers variable plumage color [53, 68] was located approximately 531 kb away from *CCDC171* (Fig. 2c). The average of LD value between them was estimated at <0.2. These observations suggested that the gene controlling plumage color in our population was different from *TYRP1*. We then chose eight SNPs significantly associated with plumage color, five of which are located within *CCDC171*, and designed PCR primers to amplify these SNP markers to genotype an additional 100 “maroon” and 100 “yellow” quails. Interestingly, 99.75% of these SNPs were consistent between genotype and phenotype, suggesting that the *CCDC171* gene does control plumage color in quail (Fig. 5b). We cloned the *CCDC171* gene from yellow and maroon quail and found that this gene encoded different transcripts in quail depending on plumage color (Figs 5c and S21). To examine the nature of the *CCDC171* genetic variants, we characterized the transcripts from the maroon and yellow alleles. The transcript from the yellow allele was longer than the maroon transcript (about 232 bp) at the upstream region of the translation initiation site of maroon and has a deletion (147 bp) at position 787. In addition, we examined the differential expression of *CCDC171* in yellow and maroon quails and found there was no significant difference between the collected samples (*t*-test, *P* >0.05).

## Discussion

Birds represent the most widespread class of domesticated animals in the world and are the subjects of many evolutionary, biological, and pathology studies that illustrate the relationships among these avian species [30]. The timing of sexual maturity is critical for both plants and animals. Quail have a unique maturation program compared with other birds and reach sexual maturity in a very short time. We detected four promising genes for this trait under positive selection in the *GnRH* signaling pathway in quail. Gonadotropins act on the testis and ovary to promote their development and the production of steroid hormones [69]. Further functional analysis of these genes should provide new insights into the genetic mechanisms that regulate avian sexual maturity.

Analyzing the genes and mutations related to the development and evolution of agronomic traits in quail will also improve our understanding of the genetics of domestication. Genome-wide comparisons of domesticated (egg-type and meat-type lines) with wild quail identified several footprints of artificial selection. These selective sweep regions harbor candidate genes associated with important agro-economic traits. Genetic variations in these genes will be a rich resource for improving quail egg and meat production *via* genetic selection. It is worth noting that egg-type and meat-type quails did not share selective sweep regions when compared with wild quails (Fig. S11), meaning that egg-type/meat-type quails might have been independently selected after domestication or that there were two separate domestication events in quails. Further studies are required to fully describe the domestication history of quails. Based on re-sequencing data, we have also identified a haplotype that is completely correlated with the control of “maroon/yellow” plumage color, a trait that has been used extensively in the breeding of domestic quail as a sex-linked marker.

Some recent studies [70] that use genomic data support our current understanding of the phylogeny of the Perdicinae, Meleagridinae, and Phasianinae families. However, without genome-wide data we have not been able to make strong conclusions. Here, we used whole-genome sequences of Japanese quail, turkey, and chicken to represent each clade and resolve the phylogenetic relationships among the Perdicinae, Meleagridinae, and Phasianinae families. Our study provided fully resolved branches with genome-scale data, supporting a split of the Perdicinae and Phasianinae branches from the Meleagridinae branch about 69 MYA. Calibration based on fossils of early penguins, together with mitochondrial genome sequences of a modern albatross (*Diomedea melanophris*), petrel (*Pterodroma brevirostris* ), and loon (*Gavia stellata*), allowed the divergence time of the Anseriformes and Galliformes to be estimated as 77.1± 2.5 MYA [5]. Other recent avian genome data were used to estimate the divergence of Anseriformes and Galliformes at about 66 MYA [71]. The resolution of their phylogeny will improve our understanding of the genetics of speciation of quail, chicken, and turkey. In this research, we obtained a high-quality draft of the Japanese quail genome and whole-genome resequencing data of multiple quail sub-populations that will provide new opportunities to further understand quail biology and develop molecular markers for improving economically important agronomic traits.

## Conclusions

In conclusion, we accomplished the genome assembly of quail with high-depth sequencing and carried out re-sequencing for 71 domestic and wild quail. We have addressed many of the long-term arguments of phylogeny of quail, turkey, and chicken and interpreted the biological mechanism of very early sexual maturity for quail. From the GWAS analysis, we detected a haplotype marker on chromosome Z, which is important for quail breeding. These analyses should provide a valuable resource for the future studies for quail.

## Materials and Methods

### Animal samples collection

All 31 wild and domestic quails were collected from China. Of these, the 10 wild quails were sampled from the common habitats of wild quails in Henan province and Shandong province, respectively. The other domestic quails were provided by local breeding companies (Table S11). The maroon or yellow plumage population were derived from two pure lines offered from Hubei Shendan Healthy Food Co., Ltd. In our previous investigation, we found that the F2 population from a crossing of the maroon plumage line and the yellow plumage line showed a 3:1 segregation ratio in plumage color. Thus, we randomly chose 20 yellows and 20 maroons from the pure lines for the association study (Table S20). All 71 quail samples were used for re-sequencing by BGI-Shenzhen. Additionally, the 100 maroon ones and the 100 yellow ones were also derived from the 2 pure lines for validation of the plumage color gene.

### Genome sequencing and assembly

A female quail collected from the maroon population was used for all genome sequencing. All experiments in this project were performed according to the principles of the animal ethics committee at BGI (China). DNA samples were isolated from blood following standard molecular biology techniques. A series of libraries of different insert sizes ranging from 170 bp to 40 kb (170 bp, 500 bp, 800 bp, 2 kb, 5 kb, 10 kb, 20 kb, and 40 kb) was constructed and used for a shotgun sequencing strategy. The Illumina HiSeq 2000 system was used to generate paired-end reads. A total of 262 Gb of raw data was obtained and reads were filtered based on the following criteria. Reads with >10% unidentified (N) bases, with >40% low-quality bases, were contaminated by adaptors, or were duplicated during PCR were discarded; about 199 Gb of clean data remained. The genome size (G) of quail was first estimated at about 1.1 Gb using the 17-mer depth frequency distribution method: G = K-mer_num/Peak_depth (Fig. S1). The genome was assembled using SOAPdenovo2 v2.04.4 (SOAPdenovo2, RRID:SCR_014986) [32]. Next, paired-end reads were mapped back to the initial assembled genome to link contigs into long scaffolds. Benchmarking Universal Single-Copy Orthologs v2 (BUSCO, RRID:SCR_015008) [72] was used to assess the assembly of genome with lineage dataset vertebrata_odb9.

### Genome annotation

RepeatMasker v4.0.5 (RepeatMasker, RRID:SCR_012954) [73] and Repeat-ProteinMask v4.0.5 were used to search for TEs (transposable elements) against the RepBase library v20.04 [74] to detect known repeats. A custom TE library was then constructed using RepeatModeller v1.0.8 and LTR_FINDER v1.0.6 [75] for *de novo* detection of repeats. Tandem Repeat Finder v4.0.7 [76] was also used to predict tandem repeats. Final results of TE detection were integrated using in-house scripts.

Homology-based and *ab initio* gene prediction methods, assisted by transcriptome sequencing, were used to analyze coding DNA sequences and to model genes. Initially, protein data for *Homo sapiens* (human), *Meleagris gallopavo* (turkey), *Gallus gallus* (chicken), *Taeniopygia guttata* (zebra finch), and *Anas platyrhynchos* (duck) were downloaded from the Ensembl v80 database [77] and aligned to the predicted proteins encoded by the quail genome using BLAT (BLAT, RRID:SCR_011919) [78]. GeneWise v2.2.0 (GeneWise, RRID:SCR_015054) [79] was then used to further improve the accuracy of alignments and predict gene structures. AUGUSTUS v3.1 (Augustus: Gene Prediction, RRID:SCR_008417) [80] and GENSCAN v1.0 (GENSCAN, RRID:SCR_012902) [81] were then used for *ab initio* gene prediction. Transcriptome reads were mapped to the genome with TopHat v1.2 (TopHat, RRID:SCR_013035) [82] and Cufflinks v2.2.1 (Cufflinks, RRID:SCR_014597) [83] was used to confirm gene structures. Subsequently, we combined the homology-based and *de novo*-predicted gene sets using GLEAN [84] and integrated the GLEAN and transcriptome results with in-house scripts to generate a representative and non-redundant gene set.

### Gene evolutionary analysis

Gene families in quail, chicken (Ensembl v80), duck (Ensembl v80), *Columba livia* (pigeon, [85]), *Falco cherrug* (Saker falcon, [86]), *Falco peregrinus* (Peregrine falcon, [87]), *Ficedula albicollis* (collared flycatcher, Ensembl v80), *Geospiza fortis* (medium ground finch, [88]), turkey (Ensembl v80), *Pseudopodoces humilis* (ground tit, [89]), zebra finch (Ensembl v80), and *Alligator sinensis* (Chinese alligator, ASM45574v1), as an outgroup, were defined using TreeFam [90]. Phylogenetic trees were then constructed using MrBayes (MrBayes, RRID:SCR_012067) [91] and PhyML (PhyML, RRID:SCR_014629) [92] with four-fold degenerate (4D) sites of 4,393 single-copy orthologs shared among the 12 species analyzed here. Divergence times were estimated using MCMCTree [93] from the PAML (PAML, RRID:SCR_014932) package [94] together with three fossil dates from the TimeTree database [95, 96] for calibration. Analyses of the expansion and contraction of gene families were carried out using Computational Analysis of Gene Family Evolution [97] using a random birth and death model with a global parameter, λ, which represents the probability of both gain and loss of a gene over a given time interval. Conditional *P*-values were calculated and defined as significant at values of <0.05. To detect PSGs, the coding sequences of all the single-copy orthologous genes were aligned using PRANK [98 ], and poorly aligned sites were removed using gBlocks [99]. High-quality alignments were then filtered to estimate the ratios (ω = d_N_/d_S_) of nonsynonymous nucleotide substitutions (d_N_) to synonymous nucleotide substitutions (d_S_) for these genes in the target quail branch (ω_0_), other branches (ω_1_ ), or all branches (ω_2_) using the codeml program with an improved branch-site model (TEST-II) [100] (model = 2, NSsites = 2) and the maximum likelihood method in the PAML package [94]. TEST-II is a likelihood ratio test that compares a null hypothesis with fixed ω = 1 with model A that allows ω_2_>1 in the foreground lineages. TEST-II can discriminate relaxed selective constraints analysis from positive selection and is a direct test for positive selection on the foreground lineages [101]. Positively selected sites were detected by using Bayes Empirical Bayes method [102 ], which can avoid an excessive false positive rate [103].

### Resequencing and SNP calling

A total of 71 individuals were chosen for resequencing (see more information regarding samples). Genomic DNAs were isolated and then used to construct Illumina libraries with an insert size of 500 bp. The Illumina HiSeq 2000 platform was used to generate paired-end reads, and raw data were filtered by removing reads containing >50% low-quality bases (Q value ≤5), reads containing >5% unidentified (N) bases, and those with adapter contamination. The clean reads were mapped to the assembled reference genome using BWA software v0.7.12 (BWA, RRID:SCR_010910) [104] with parameters “-m 200000 -o 1 -e 30 -i 15 -L -I -t 4 -n 0.04 -R 20”, and the results were transformed into indexed BAM files using SMtools v0.1.18 [105]. The picard package v1.105 and Genome Analysis Toolkit v 3.3-0 (GATK, RRID:SCR_001876), ) [106] were then used for SNP calling. To obtain high-quality SNPs, we: 1) deleted duplicate reads; 2) improved alignments using the IndelRealigner package in GATK; 3) recalibrated base quality scores using the BaseRecalibrator package in GATK; 4) called SNPs using the UnifiedGenotyper package in GATK with a minimum phred-scaled confidence value of 50 and a minimum phred-scaled confidence threshold of 10 for calling variants; 5) assessed variant quality using the VariantRecalibrator and ApplyRecalibration packages with truth sensitivity filter level of 99 in GATK; and 6) filtered SNPs using the VariantFiltration package in GATK with parameters “–filterExpression “QD < 2.0 ||MQ < 40.0 || ReadPosRankSum< -8.0 ||FS > 60.0 || HaplotypeScore> 13.0 || MQRankSum <-12.5” –filterNameLowQualFilter –missingValuesInExpressionsShouldEvaluateAsFailing”.

### SNP quality control (QC)

The chromosomal variant call format (VCF) files were transformed into PLINK format by using VCFtools v0.1.13 [107 ], and subsequent analyses were performed by using PLINK v1.07 (PLINK, RRID:SCR_001757) [108]. As the default chromosome handling type in PLINK is for human (1:22, X, Y), the PLINK files for male quail (ZZ) and the female quail (WZ) were swapped with each other before data were analyzed because the heterogametic gender in quail is female. Additionally, the command –dog (39n) was added at the beginning of each command line to ensure that all quail chromosomes would be included.

Individual quality control consisted of the following three steps: 1) determining the sex of individuals, 2) detecting individuals with missing genotypes and 3) identifying duplicate or highly related individuals. Any discordant sex information was checked in terms of the heterozygosity rates on the Z chromosome as described by the *F* statistic, that is, any individual quail for which the *F*-value was <0.8 in a male quail (ZZ) or >0.2 in a female quail (ZW) would be removed from the sample set. The missing genotype rate for each individual was set to <10% to filter out individuals with unreliable genotype information. Case-control association studies assume that all individuals in a population are unrelated. We used a complete linkage agglomerative clustering method that was based on pairwise identity-by-state (IBS) to identify the genomic similarity of pairs of individuals. Any individual with an IBS >0.9 would be filtered out of the sample set.

SNP quality control consisted of the following four steps: 1) estimating the missing genotype rate (MGR) for each SNP, 2) determining whether there was a significant difference in the rate of missing SNP genotypes between the case and control groups, 3) filtering out SNPs with very low minor allele frequencies, and 4) filtering out SNPs with frequencies that deviate significantly from Hardy-Weinberg Equilibrium. Filtering out the low-quality SNPs helped not only to avoid false-positives, but also to enhance our ability to identify the loci significantly associated with traits. Therefore, the criteria for filtering were MGR >0.05, a significant difference in MGR between case and control according to *t* test, at *P* < 0.05, minor allele frequencies <0.05, and a *P*-value for deviation from Hardy-Weinberg Equilibrium <0.0001.

Extensive genome-wide regions of high linkage disequilibrium (LD) in quail strongly influenced the results of population structure, principal component, and association analyses. Thus, we pruned out the pairwise SNPs with *r*^2^ values of >0.2 in each 50-bpsliding window, and set 10-bpsteps for sliding window analysis to ensure 80% overlaps between any two adjacent windows.

### Population structure analysis

The phylogenic tree was constructed using the neighbor-joining method in MEGA v6.0 (MEGA Software, RRID:SCR_000667) [109] based on a pairwise distance matrix that was estimated using IBS distances in PLINK v1.07. Analysis of population stratification was conducted by performing complete linkage clustering of individuals using autosomal genome-wide SNP data in PLINK. PCA was carried out using the smartpca script [110 ], and the scatter plots were drawn by using R v3.2.2 [111]. We used ADMIXTURE v1.3 [43] to analyze population structure, which uses the likelihood model-based manner from large autosomal SNP genotype datasets. The number of populations (*K*) was set from *K* = 2 to 5 to obtain the maximum likelihood estimates that would allow us to infer population structure. The cross-validation procedure was performed to exhibit a low cross-validation error, which made it fairly clear what the optimal *K* value was. The parameter standard errors were estimated by using 100 bootstrap replicates. The cross-validation plot was drawn by using R v3.3.2. The average LD of a pair of SNPs in a 300-kbsliding window was estimated by using PopLDdecay v2.69 [112], and the LD decay curves for the three populations were drawn by using R v3.3.2.

### Calculation of nucleotide diversity and estimation of population differentiation using *F*_ST_

Watterson's estimator *θ*_w_ [38] and the average number of pairwise differences per sequence estimator *π* [39] were calculated using in-house Perl scripts. Tajima's *D* [39] was estimated using *θ*_w_, *π*, and the number of sequences. We scanned the whole genome to calculate the three estimators by using the 90% overlapped sliding window with a size of 100 kb or 50 kb. The fixation index (*F*_ST_), a measure of population differentiation due to genetic structure [113], was estimated by using VCFtools v0.1.13 also with 50-k band 100-kb 90% overlapped sliding windows on a genome-wide scale.

### Association analysis and conditional haplotype-based association testing

The post-QC data was saved as PED format and later was used for GWAS via GEMMA v0.94. The centered relatedness matrix was calculated with the parameter “–gk 1”. The relatedness matrix was considered as a covariance using a linear mixed model to perform the Wald test, likelihood ratio test, and score test. The GWAS results were shown as a Manhattan plot and Q-Q plot and were drawn by using the *qqman* package in R v3.2.2. The SNP with the most significant effect on phenotypic variation was regarded as the index SNP, and the flanking 100-kb region of the index SNP was scanned for haplotype construction. In this region, the SNPs with high LD (*r*^2^ >0.7) and significant association to plumage color (adjusted *P* <0.01) were grouped into a clump. Then, the SNPs gathered in a clump were extracted by using PLINK v1.07 and transformed into Haploview format for conditional haplotype association testing. The haplotypes in block were estimated with a permutation of 5,000, and the LD plot between gene *CDCC171* and *TYRP1* was drawn using Haplotype v4.2 [114].

### Molecular experiments

#### FSH testing by ELISA

We selected 100 male quails, 100 female quails, 100 hens, and 100 cocks from Hubei in China, and they were raised under the same conditions. In four populations, blood samples from 10 individuals were collected every week (0–6 weeks). The sera were separated from the blood and stored at -20°C for testing. The *FSH* hormone of quails and chicken was tested using the FSH ELISA Kit (Abcam, UK) according to the manufacturer's instructions.

#### *CCDC171* transcript cloning and expression

We designed three and four pairs of primers (Table S22) to clone transcripts of *CCDC171* in yellow and maroon quails, respectively. For determining the differential expression of *CCDC171* in yellow and maroon quail, we collected hair follicles, back skin, and abdomen skin from three “yellow” quails and three “maroon” quails. Two pairs of primers were designed to detect the differential expression of *CCDC171* by qPCR.

#### Validation of SNPs

DNA from the different plumage quails was extracted from blood samples following standard molecular biology techniques and stored at -80°C, and we used software Primer 6.0 for designing primers to validate the eight SNPs that were significantly associated with plumage color. The PCR products were sequenced on the Sanger sequencing platform.

## Data availability

Resequencing and transcriptomic raw data are available from NCBI SRP104331. Data supporting the manuscript, including sequence assembly files, annotations, and BUSCO results, are also available via the *GigaScience* database, GigaDB [115].

## Additional files

**Additional file 1: Figures S1–21, Tables S1–12, Table S18, Tables S20–22**.

**Additional file 2: Table S13**. Diversity statistics of 31 quails.

**Additional file 3: Table S14**. The large scale of genomic regions showing ROD between egg-type quail and wild quail at 100-kboverlapping sliding window in 10-kb steps.

**Additional file 4: Table S15**. Functional enrichment for selective sweep regions between egg-type and wild quails.

**Additional file 5: Table S16**. The large scale of genomic regions showing ROD between meat-type quail and wild quail at 100-kboverlapping sliding window in 10-kb steps.

**Additional file 6: Table S17**. Functional enrichment for selective sweep regions between meat-type and wild quails.

**Additional file 7: Table S19**. Copy numbers of innate immune response-related genes among quail, chicken, turkey, and duck.

## Authors’ contributions

Y.W. was responsible for collecting samples for sequencing, carrying out experimental verification, and co-drafting the manuscript. Y.Z. made contributions to genome annotation, phylogenetic analysis, immune-related gene analysis, detecting genes under positive selection, SNP calling in re-sequencing data, and co-drafting of the manuscript. Z.H. and J.P. designed the scientific objectives and co-drafted the manuscript. S.S. and J.C. carried out SNP quality control, GWAS analysis, detecting selective sweep regions, and co-drafting of the manuscript. G.F. participated in genome assembly, analyzing inversions, and co-drafting of the manuscript. W.C., X.C., J.J., X.F., and X.X. participated in project management and manuscript revision. H.L., J.S., A.P., Y.P., Z.L., H.Z., J.S., C.Z., H.T., H.X., and C.L. worked on sample preparation and experimental verification. X.D., G.H., P.Y., H.Z., T.Y., B.W., H.Y., and M.B. took part in data processing. D.B. and W.W. provided suggestions and revised the manuscript. N.Y., X.L., and J.D. designed this project, provided suggestions, and revised the manuscript.

## Competing interests

The authors declare that they have no competing interests.

## Abbreviations

NCBI: National Center for Biotechnology Information; MYA: million years ago; BUSCO: Benchmarking Universal Single-Copy Orthologs; KEGG: Kyoto Encyclopedia of Genes and Genomes; GO: Gene Ontology; LD: linkage disequilibrium; SNP: Single Nucleotide Polymorphism; ROD: reduction of diversity; MHC: major histocompatibility complex; GWAS: Genome-wide association study.

## Supplementary Material

GIGA-D-17-00216_Original_Submission.pdf

GIGA-D-17-00216_Revision_1.pdf

Response_to_Reviewer_Comments_Original_Submission.pdf

Reviewer_1_Report_(Original_Submission) -- Dominic Wright 10/24/2017 Reviewed

Reviewer_2_Report_(Original_Submission) -- Shyam Gopalakrishnan 10/31/2017 Reviewed

Reviewer_2_Report_(Revision_1) -- Shyam Gopalakrishnan 4/4/2018 Reviewed

Reviewer_3_Original_Submission_Attachment_Reviewer_Figures.pdf

Reviewer_3_Report_(Original_Submission) -- Jason Travis Howard 12/3/2017 Reviewed

Reviewer_3_Report_(Revision_1) -- Jason Travis Howard 3/19/2018 Reviewed

Additional Files
