# Supplementary material for: Population genomic data reveal genes related to important traits of quail
Source: Gigascience. 2018 May 11;7(5):giy049. doi: 10.1093/gigascience/giy049 (PMC5961004; doi:10.1093/gigascience/giy049)
Supplement: GIGA-D-17-00216_Original_Submission.pdf [file giy049_giga-d-17-00216_original_submission.pdf]

GigaScience
Population genomic data reveal genes related to important traits of quail
--Manuscript Draft--

**Manuscript Number:**	GIGA-D-17-00216
**Full Title:**	Population genomic data reveal genes related to important traits of quail
**Article Type:**	Research
**Funding Information:**	Study and application of functional genes in quail precocious traits based on whole genome analysis (2012tsxk09) — Dr Jinping Du<br>Molecular breeding of poultry (2014-2017 ZD146) — Dr Yan Wu
**Abstract:**	Abstract<br>Background: Japanese quail (Coturnix japonica), a recently domesticated poultry species, is important not only as an agricultural product, but also as a model bird species for genetic research. However, most of the biological questions, genomics, phylogenetics and genetics of some important economic traits, are not solved. In view of this, it is necessary to complete a high-quality genome sequence as well as series of comparative genomics, evolution and functional studies.<br>Result: Here we present a quail genome assembly spanning 1.04Gb with 86.63% of sequences anchored to 30 chromosomes (28 autosomes and two sex chromosomes Z/W). Our genomic data solved the long-term debate of phylogeny among Perdicinae (Japanese quail), Meleagridinae (turkey) and Phasianinae (chicken). Comparative genomics and functional genomic data found that four candidate genes involved in early-maturation had experienced positive selection, and one of them encodes follicle stimulating hormone beta (FSHβ), which is correlated with different FSHβ levels in quail and chicken. We re-sequenced 31 quails (10 wild, 11 egg-type and 10 meat-type) and identified 11 and 26 candidate selective sweep regions in egg-type and meat-type lines, respectively. That only one of them shared between egg-type and meat-type lines suggested that they were subject to an independent selection. We also detected a haplotype on chromosome Z which was closely linked with marron/yellow plumage in quail using population resequencing and genome-wide association study. This haplotype block would be useful for quail breeding program.<br>Conclusions: This study provided a high-quality quail reference genome, identified quail specific genes and solved quail phylogeny. We have identified genes related to quail's early-maturation and a marker for plumage color which is significant for quail breeding. The present results will facilitate biological discovery in quail and help us to elucidate the evolutionary process within the Phasianidae family.
**Corresponding Author:**	Xin Liu, Ph.D.<br>BGI<br>CHINA
**Corresponding Author Secondary Information:**	
**Corresponding Author's Institution:**	BGI
**Corresponding Author's Secondary Institution:**	
**First Author:**	Yan Wu
**First Author Secondary Information:**	
**Order of Authors:**	Yan Wu<br>Yaolei Zhang<br>Jinsong Pi<br>Jiang Chen<br>Guangyi Fan

Powered by Editorial Manager® and ProduXion Manager® from Aries Systems Corporation

	Huaqiao Liu
	Jie Shen
	Wenbin Chen
	Ailuan Pan
	Xiaoli Chen
	Yuejin Pu
	He Zhang
	Zhenhua Liang
	jianbo Jian
	Hao Zhang
	Bin Wu
	Jing Sun
	Jianwei Chen
	Hu Tao
	Ting Yang
	Hongwei Xiao
	Huan Yang
	Chuanwei Zheng
	Mingzhou Bai
	Xiaodong Fang
	David W. Burt
	Wen Wang
	Qingyi Li
	Xun Xu
	Chengfeng Li
	Huanming Yang
	Jian Wang
	Zhuocheng Hou
	Ning Yang
	Xin Liu, Ph.D.
	Jinping Du
**Order of Authors Secondary Information:**	
**Opposed Reviewers:**	Yoichi Matsuda<br>Laboratory of animal genetics, Graduate school of Bioagricultural sciences, Nagoya University, Furo-cho, Chikusa-ku, Nagoya 464-8601, Japan
	Tomohiro Kono<br>Genome Research Center, NODAI Research Institute, Tokyo University of Agriculture, 1-1-1 Sakuragaoka, Setagaya-ku, Tokyo 156-8502, Japan
	Ryouka Kawahara-Miki<br>Genome Research Center, NODAI Research Institute, Tokyo University of Agriculture, 1-1-1, Sakuragaoka, Setagaya-ku, Tokyo 156-8502, Japan

Powered by Editorial Manager® and ProduXion Manager® from Aries Systems Corporation

	Wesley Warren<br>McDonnell Genome Institute - Washington University School of Medicine
**Additional Information:**	
**Question**	**Response**
Are you submitting this manuscript to a special series or article collection?	No
**Experimental design and statistics**<br><br>Full details of the experimental design and statistical methods used should be given in the Methods section, as detailed in our Minimum Standards Reporting Checklist. Information essential to interpreting the data presented should be made available in the figure legends.<br><br>Have you included all the information requested in your manuscript?	Yes
**Resources**<br><br>A description of all resources used, including antibodies, cell lines, animals and software tools, with enough information to allow them to be uniquely identified, should be included in the Methods section. Authors are strongly encouraged to cite Research Resource Identifiers (RRIDs) for antibodies, model organisms and tools, where possible.<br><br>Have you included the information requested as detailed in our Minimum Standards Reporting Checklist?	Yes
**Availability of data and materials**<br><br>All datasets and code on which the conclusions of the paper rely must be either included in your submission or deposited in publicly available repositories (where available and ethically appropriate), referencing such data using a unique identifier in the references and in the “Availability of Data and Materials” section of your manuscript.<br><br>Have you have met the above requirement as detailed in our Minimum Standards Reporting Checklist?	Yes

Powered by Editorial Manager® and ProduXion Manager® from Aries Systems Corporation

Powered by Editorial Manager® and ProduXion Manager® from Aries Systems Corporation

Manuscript
Click here to download Manuscript Quail genome manuscript.docx

Population genomic data reveal genes related to important traits of quail

Yan Wu[1,3,*], Yaolei Zhang[2,6,10,*], Jinsong Pi[1,*], Jiang Chen[2,10,*], Guangyi Fan[2,7,10,*], Huaqiao Liu[5,*], Jie Shen[1], Wenbin Chen[2], Ailuan Pan[1], Xiaoli Chen[2], Yuejin Pu[1], He Zhang[2], Zhenhua Liang[1], Jianbo Jian[2], Hao Zhang[1], Bin Wu[2], Jing Sun[1], Jianwei Chen[2], Hu Tao[1], Ting Yang[2], Hongwei Xiao[1], Huan Yang[2], Chuanwei Zheng[1], Mingzhou Bai[2], Xiaodong Fang[2], David W. Burt[8], Wen Wang[9], Qingyi Li[5], Xun Xu[2,10], Chengfeng Li[5], Huanming Yang[2,11], Jian Wang[2,11], Zhuocheng Hou[4,†], Ning Yang[4,†], Xin Liu[2,10,†], Jinping Du[1,†]

Abstract

Background: Japanese quail (*Coturnix japonica*), a recently domesticated poultry species, is important not only as an agricultural product, but also as a model bird species for genetic research. However, most of the biological questions, genomics, phylogenetics and genetics of some important economic traits, are not solved. In view of this, it is necessary to complete a high-quality genome sequence as well as series of comparative genomics, evolution and functional studies.

Result: Here we present a quail genome assembly spanning 1.04Gb with 86.63% of sequences anchored to 30 chromosomes (28 autosomes and two sex chromosomes Z/W). Our genomic data solved the long-term debate of phylogeny among Perdicinae (Japanese quail), Meleagridinae (turkey) and Phasianinae (chicken). Comparative genomics and functional genomic data found that four candidate genes involved in early-maturation had experienced positive selection, and one of them encodes follicle stimulating hormone beta (*FSHβ*), which is correlated with different *FSHβ* levels in quail and chicken. We re-sequenced 31 quails (10 wild, 11 egg-type and 10 meat-type) and identified 11 and 26 candidate selective sweep regions in egg-type and meat-type lines, respectively. That only one of them shared between egg-type and meat-type lines suggested that they were subject to an independent selection. We also detected a haplotype on chromosome Z which was closely linked with marron/yellow plumage in quail using population resequencing and genome-wide association study. This haplotype block would be useful for quail breeding program.

Conclusions: This study provided a high-quality quail reference genome, identified

quail specific genes and solved quail phylogeny. We have identified genes related to quail's early-maturation and a marker for plumage color which is significant for quail breeding. The present results will facilitate biological discovery in quail and help us to elucidate the evolutionary process within the Phasianidae family.

Keywords: Japanese quail, Genome assembly, early-maturation, Phylogeny, Resequencing, plumage color, Quail breeding

Background

Most of the poultry eggs and meat products in the world come from species that are members of the Phasianidae family including chicken (*Gallus gallus*), turkey (*Meleagris gallopavo*) and Japanese quail (*Coturnix japonica*) within the order Galliformes. The genomes of the two widely domesticated avian species, chicken and turkey, have already been sequenced[1, 2]. Accordingly, the first quail draft genome sequence had been reported with the length of contig N50 of 1.5kb by Tokyo University of Agriculture in 2013, and then the group lately developed an improved draft and lengthened the contig N50 to 32kb (NCBI BioSample: SAMD00009971)[3]. Recently, another Japanese quail draft genome at a level of chromosome was published by using quail inbred line Cons DD (INRA) (NCBI BioSample: SAMN03989050). However, with these reference genome assemblies, most of biological questions involving genomics and phylogenetics are still unsolved in quail.

The Phasianidae family has its origin about 30–46 million years ago (MYA)[1, 4-7]. Even though high degrees of conservation of synteny and chromosome homology have been observed between quail and chicken[8, 9], these species display a great diversity of phenotypes among the three widely used domesticated birds. Japanese quail reach sexual maturity at 5–6 weeks of age[10], while chicken and turkey reach this stage in about 18–22 weeks[11]. Body mass at maturity stage of meat-type quail is about 10% that of broiler chicken and 2.5% that of turkey[11], but quail have the fastest growth rate of all species in the Phasianidae family[12, 13]. Furthermore, female quails generally present a larger body size than that do in males,

while the reverse is true both in chickens and turkeys. Not only of that, there are also quite distinct differences existing between subpopulations of quail, though yet it has been not much long period since quail branched off from Phasianidae family. According to historical records, the domestication of Japanese quail was based on birds selected for their crowing abilities since 11th century. However, the domestic strains, which were selected for commercial egg and meat productions, were improved only from 1910s[14] . Today, the domestic quails has differed from wild population in many traits, such as variations in plumage color, increased body size, acceleration of sexual maturity, lengthening of the reproductive phase, and disappearance of migratory characteristics[15]. Especially because of the important roles of plumage color in signaling, mate choice and evolution, mapping the gene conferring sex-linked plumage color is significant for commercial breeding in quail[13-17]. Additionally, it is proved that quail provides more advantage over reproduction interval and space requirements than chicken, so quail has been also considered as an excellent avian model for embryonic development, reproduction, sexual differentiation, environmental toxicant indication and disease resistance[16-24].

Some of the key avian model systems (i.e., the chicken, turkey, and Japanese quail) are not well solved even more locus were included[25]. Even some preliminary understanding towards phylogenies of *Phasianidae* has been learnt from archaeologic and demographic techniques, the issue that how the *Phasianidae* family evolved has been under debate for years. Due to the rapid differentiation within the *Phasianidae*

family, several studies based on fossil evidence are inconsistent with those based on mtDNA mainly in phylogenies and divergence time[5, 6, 26]. With rapid diversification observed during the Eocene and the short divergence times within some lineages, the phylogenies of galliform birds (including the most widely used domestic bird species, chicken, turkey and quail) usually have low bootstrap support values[27]. Phylogenies based on the *CR1* retrotransposon support the hypothesis that quail and turkey are more closely related than quail and chicken, while those based on mitochondrial genome data support the hypothesis that quail is more closely to chicken than turkey[27]. However, Phylogenies of the *Perdicinae* (Japanese quail), *Meleagridinae* (turkey) and *Phasianinae* (chicken) subfamilies are still not clear with the information that only are inferred from either current fossil evidence or parts of genome. Therefore, comparing these species at a whole-genome level will enable us to recognize the process of speciation of *Phasianidae* family better. A high-quality genome assembly of the quail together with population genomic data of the quail will help to address the above questions[27, 28].

Here we report another sequence of the genome of Japanese quail (*Coturnix japonica*), the resequencing of 71 domestic and wild quail, and experimental results about early sexual maturity and plumage color. These data were used to characterize mechanisms of early sexual maturity in quail; resolve the phylogeny and divergence time of the Phasianidae family (quail, chicken and turkey); and detect footprints of artificial selection in quail genome. We have also identified the genetic basis for one plumage color marker that is widely used in quail breeding. These results will

facilitate biological discovery and improvement of quail for meat and egg production and help to elucidate the basis of evolution within the Phasianidae family.

Results

Characteristics of the quail genome

High-quality genomic DNA extracted from a female quail (Shendan quail 1) was used to generate 262Gb of sequence (approximately 238-fold coverage of the whole genome) (**Table S1**) using the Illumina HiSeq 2000 platform. The genome assembled using SOAP*denovo2*[29] spans 1.04Gb (93.9% of the estimated genome size for quail, **Figure S1**) with contig N50 and scaffold N50 of 27.9 Kb and 1.8 Mb, respectively (**Table S2**). About 901 Mb of sequence (86.6% of the whole genome) was anchored to 30 chromosomes using a previously reported genetic linkage map[30] (**Figure 1a)**. We aligned these chromosomes back to the previous assembled quail genome (NCBI BioSample: SAMN03989050) and found that the two genomes came with a high degree (90.24%) of consistency (**Figure S2**). The length and GC distribution of chromosomes are highly consistent between quail and chicken genome sequences (**Figure 1b** and **Figure S3**). Further, to evaluate the quality of the assembled quail genome, seven fosmid clones, each about 40 kb in length, were sequenced and mapped back to the quail genome assembly with a high coverage ratio (>92% for all, and six of seven fosmids >98.4%) (**Table S3** and **Figure S4**). In addition, to assess the integrity of protein-coding genes in the quail genome assembly, all transcripts assembled from RNA-Seq reads sampled from hypothalamus and ovary of three

stages of quail maturity, including before-laying (BL), laying (L) and peak-laying (LP) (**Table S4**). These quality checks demonstrated the high quality of the quail genome sequence, which can serve as a reference genome for further quail genome research.

Genome annotation of the quail genome assembly included transposable elements (TEs) and protein-coding genes. TEs comprise 12.4% of the genome, which is a little higher than the average value in the class Aves[31], and 9.4% consists of long interspersed nuclear elements (LINEs) (**Figure 1a** and **Table S5**). Gene prediction combined several methods, including homology searches, *ab initio* prediction and RNA-Seq data. The merged results revealed evidence for 16,210 protein-coding genes in the quail genome (**Table S6**), and 15,972 (98.5%) genes were also supported by known protein-coding entries in at least one of the following databases: Swiss-Prot, InterPro, GO, TrEMB or KEGG (**Table S7**).

Evolutionary relationships within the Phasianidae family

To resolve phylogenetic debate in the Phasianidae family and establish the phylogenetic position of the quail with other avian species, we defined 12,178 gene families in quail and 10 other representative bird species, with *Alligator sinensis* (Chinese alligator) serving as an outgroup (**Figure S5**). 9,631 gene families were shared among four species (*Taeniopygia guttata*, *Pseudopodoces humilis*, *Gallus gallus* and *Coturnix japonica*, **Figure S6**) and 4,393 single-copy orthologous were shared among 12 species. These single-copy orthologous genes were used to construct a phylogenetic tree (**Figure 2a** and **Figure S7**) and used to estimate the divergence

times of the quail from other birds. Quail mapped to the evolutionary branch containing domesticated poultry and was most closely related to the chicken lineage sharing a common ancestor about 22.2 MYA (**Figure 2a**). We used our genome-wide comparative data to estimate the divergence time of *Galliformes* and *Anseriformes* at 69.1 (64.5–75.4) MYA. Our results therefore fully supported a closer relationship between quail and chicken than between quail and turkey. The phylogeny we generated implied that the quail and chicken genomes likely share significant similarities, which makes further comparison of their genomes intriguing.

In total, 95.5% of quail genome sequences occurred in blocks colinear with those in chicken (**Figure 2b** and **Table S8**). However, a total of 131 large inversions (block length >5 kb) between quail and chicken chromosomes were also identified and most of these were located on chromosomes 1 (24 breakpoints) and Z (24 breakpoints) (**Table S9**). Next, to investigate the nature of chromosome breaks that differentiate the quail and chicken genomes, and to associate these differences with possible phenotypic changes during their divergence, we tested for gene set enrichments at the boundaries. We identified 433 genes located within the 1-kb regions flanking the breakpoints of these inversions (**Table S9**). We tested for gene functions enrichment within these inversions and searched for candidate mutations that might contribute to specific phenotypes in quail compared with chicken. Results of GO term enrichment analysis of these genes revealed the terms GO:0005882: intermediate filament (P=1.53e-05) and GO:0005200: structural constituent of cytoskeleton (P=0.00029) was significantly enriched (**Figure S8** and **Figure S9**). In particular, a gene encoding

tyrosinase-related protein 1 (*TYRP1*) was identified in the flanking region of an inversion on chromosome Z, which had been reported as a candidate locus for the recessive, sex-linked roux (br(r)) phenotype in Japanese quail[32] (**Figure 2c**).

Nucleotide diversity and population structure

To obtain a comprehensive understanding of genetic diversity in quail population, we collected a total of 31 samples for genome re-sequencing, which included 10 quails from wild population,11 egg-type quails and 10 meat-type quails from domesticated subpopulations (**Table S10**). We sequenced these samples with an average of reads coverage of ~3.5×and mapped the reads to our reference genome (Shendan quail 1). Eventually, we identified a total of 22,495,404 bi-allelic SNPs among the 31 re-sequenced samples. Of these, the average of genotype missing rates in wild, egg-type and meat-type quails were 11.2%, 8.6% and 7.3%, respectively. Through a series of quality controls for SNP filtration (*See more detail in Method*), we obtained a final total of 21,886,307 high-quality SNPs, which included 15,573,075, 12,722,389 and 14,107,546 SNPs in wild quails, egg-type quails and meat-type quails, respectively (**Table 1**). Of the ~21M high-quality SNPs, only 319,052 SNPs were located in exon regions, yet there were 8,929,259 SNPs in intron regions. Thus, the ratio of the number of SNPs in exon regions and intron regions was 3.57e-2, which was lower than that of turkey (4.30e-2)[33] but was similar to that of chicken (3.50e-2)[34]. Accordingly, we found that the non-synonymous SNPs (N) and the synonymous SNPs (S) in quail were 90,031 and 229,019, respectively, with a ratio of

N/S of 0.39, which was lower than that both of chicken (0.41) and turkey (0.45). It was suggested that much more genes in quail had been subjected to different degree of selections compared to other poultries. Meanwhile, we also found that no matter in whole genome scale or in genic regions, the number of SNP within wild quail population was more than that within other two domesticated subpopulations.

To evaluate the genetic diversity of our quail population, we calculated two common summary statistics across the whole genome, π and θ_w values[35, 36], by using 100-kb sliding overlapped window with step length of 10 kb (**Table 1** and **Table S11**). The estimated amount of θ_w in wild quail population was substantially higher than that in other two domesticated subpopulations on autosomes, but the difference of π estimators among the three populations was minimal, which indicated that our wild population still contained greater genetic diversity though the divergence within wild population was not very apparent yet. On chromosome Z, however, whether π or θ_w of egg-type quail was dramatically lower than that of both wild quail and meat-type quail, even if all the egg-type quails were sampled from female individuals whose effective number of chromatid Z (N_e = 11) was slightly smaller than that of wild quails (N_e = 15) and meat-type quails (N_e = 15), respectively. Furthermore, one thing that caught our attention was that all the three populations still kept a relatively low level of diversity on genic regions of chromosome Z. It was implied that a stronger positive selection might act on chromosome Z of egg-type quail, which led to an accelerated reduction of genetic diversity on a large scale. Additionally, we also assessed an estimator of neutral test, Tajima's *D*[36], with a purpose to distinguish

between a DNA sequence evolving randomly and one evolving under a non-random process. It was found that the three populations were under neutral evolution at chromosomal level, except of the chromosome Z of wild population. This led us to suspect that the relatively conservative chromosome Z in evolutionary history exposed a narrow genetic background within our wild population.

To investigate the phylogenetic relationships and population structure among the 31 quail samples (**Table S10**), we constructed a neighbor-joining tree by using pairwise genetic distance matrix (**Figure 3a**) and performed principal component analysis (PCA) based on the variance-standardized genotype relationship matrix (**Figure 3b**).The neighbor-joining tree showed that our samples could be divided into two major clusters, corresponding to wild quails and domesticated quails, with a further subdivision of domesticated quails into egg-type quails and meat-type quails. Similarly, PCA also revealed the main divergence between the wild population and domesticated population in eigenvector 1, and the secondary divergence between the egg-type subpopulation and the meat-type subpopulation in eigenvector 2 (**Figure 3b**). However, our wild samples clustered together and it was hard to distinguish in PCA scatter plot. That was, it was likely to be a similar genetic background in the wild population, even if they exhibited rich variations in the number of segregation sites. In order to better estimate the ancestral component in our quail populations, we adopted likelihood models embedded in structure by using ADMIXTURE[37]. The initialization of population number (*K*) was tried from 2 to 7, and the cross-validation error was estimated at the minimum when *K* was set to 2 (**Figure 3d** and **Figure S10**).

It was suggested that the ideal population stratification manifested the wild population and domesticated population rather than three populations. It was because the difference between egg-type quails and meat-type quails was much less than that of the former two. Additionally, we could see that the wild populations began to appear the differentiation when K was equal to 5, indicating that the wild quails had distinct ancestors. However, we would have preferred to divide the 31 samples into three populations (egg-type, meat-type and wild quail) according to their physiological and ethological characteristics. To characterize linkage disequilibrium (LD) blocks in wild and domesticated quails, we estimated the squared correlations (r^2) of pairwise SNPs with sliding window lengths from 1 to 300 kb. LD decayed to half of its maximum within a window length of ~30 bp for wild quail, ~50 bp for egg-type quail and ~60 bp for meat-type quail, respectively (**Figure 3c**). Such rapid decay of LD in each population might be due to the high density of SNP in quail genome (one SNP in every ~70 bases on average), indicating that the heterozygosity among individuals and high heterogeneity of the quail population resulted from gene segregation and a high degree of recombination within the quail genome. Similarly, other studies involving the population structure of Aves animals also revealed the low level of LD corresponding to the open genome and fluid genomic background in bird population which could facilitate adaptive variation[38, 39].

Signals of selection across the quail genome

Due to the low level of population divergence in our wild quails, some signals of selective sweeps from short genomic regions could be masked in domesticated populations. Thus, we sought to detect the large-scale regions that exhibited successive selective sweep in whole genome between domesticated population and wild population by using 100-kb overlapping sliding window in 10-kb step. The reduction of diversity (ROD), defined as ROD = 1- $\pi_{domesticated}/\pi_{wild}$, was introduced to measure the loss of diversity in domesticated populations comparing with wild population. Additionally, in order to avoid ROD being excessively affected by the diversity in wild population, we also added significantly negative Tajima's D ($D < -2$) in domesticated population as a parallel criterion, which meant recent selective sweep or a population expansion following a complete bottleneck[40-42]. From the comparison between egg-type quails and wild quails, we identified a total of 11 large scale regions of selection sweep with the spans all greater than 100 kb, where the sliding windows presented high ROD values in the 5% right tail of the distribution of ROD frequency (**Figure S11** and **Table S12**). We also found that the fixation index (F_{ST}), a measure of population divergence due to genetic structure, was substantially calculated at a high level ($F_{ST} > 0.4$) in each sliding window of these regions. Interestingly, we observed, 10 of the 11 regions were located on chromosome Z and the longest one was ~1.8 Mb in length (**Figure 3e**). Furthermore, we identified the gene (*CCDC171*) significantly associated with quail plumage color, included in a 182-kb haplotype block (*see the association study described below*), was just located on the longest region. It was indicated that positive selection for plumage color might

have resulted in a strong selective sweep on chromosome Z in egg-type quail. More than that, a total of 73 gene sequences involving these selective sweeps were annotated by the related terms of Gene Ontology (GO). By using Kyoto Encyclopedia of Genes and Genomes (KEGG) database, we noticed that some of genes possibly played a role in sex hormones[43], embryo development[44, 45], increase of egg weight[46] and plumage color[47] (**Table S13**). Thus, we surmise that these important traits in egg-type quail might have suffered stronger artificial selection, which led to many large scales of selection sweep around these key loci on chromosome Z. Similarly, we also identified 26 large regions of selective sweep between meat-type quail and wild quail on chromosomes 1, 2, 3, 8, 13 and Z (**Figure 11** and **Table S14**). The longest span could be as long asw~1 Mb on chromosome 2. The total length of selective sweeps in meat-type quail was estimated at ~8.6 Mb, which was longer than that of egg-type quail (~5.3 Mb). That brought us to an inference that the breed improvement for meat-type quail was likely to aim at much more regions besides of some ones on chromosome Z. Subsequently, we annotated 127 genes except of ones on chromosome 8, and described their biological function by using enrichment analysis (**Table S15**). Although most of genes that correspond to relevant traits had not been verified in meat-type quail, these candidate genes would help us to facilitate understanding of domestication process in further study. However, it was worth mentioning that there was hardly any selective sweep shared between egg-type quail and meat-type quail except for only a 160-kb region (20.38-20.54 Mb) on chromosome Z, where we annotated two genes with the codes of COT10486 and

COT10487 (**Table S13** and **Table S15**). Such more different large selective sweeps, we speculated, egg-type quail and meat-type quail might be domesticated from their respective ancestral populations independently.

Despite selective sweep gave us a new insight into an evidence of domestication process, a fair number of differentially expressed genes dispersed on the whole genome, however, also contributed to the divergent traits between domesticated quails and wild quails. Thus, we picked up all the non-synonymous SNPs where the alleles were divided into domesticated group and wild group, with a loose measure of F_{ST}>0.3. The genes involved in these eligible SNPs could be annotated by GO database. According to this method, we detected 8206 divergent non-synonymous SNPs and annotated a total of 1453 genes between egg-type quail and wild quail, as similar as 11085 divergent non-synonymous SNPs and 1189 genes between meat-type quail and wild quail. Then we showed the top 20 KEGG pathways by using functional enrichment analysis for these genes, respectively for egg-type quail and meat-type quail, and found that there were 12 common biological pathways shared by egg-type quail and meat-type quail (**Figure S12** and **Figure S13**). Of these, the most significant pathway reflected the neuroactive ligand-receptor interaction, which might be related to embryonic development and sexual maturation[48, 49]. Other functional pathways, specific to egg-type quail and meat-type quail respectively, seemed not very significant in enrichments, indicating that artificial selections that targeted at these traits were not strong. In summary, many similar selections in both two domesticated subpopulations revealed that the phenotypic difference between egg-type quail and

meat-type quail was not that significant, which was consistent with the population stratification that the optimal number of populations was two rather than three.

Gene related to early sexual maturity

To explore the biological mechanism of very early sexual maturity in quail, genes were traced from both gene family evolution and positive selection events in the quail lineage. We found that several gene families have expanded in the quail genome compared with those of other domesticated birds. These expanded gene families include those encoding gonadotropin-releasing hormone 1 (*GnRH1*, **Figure S14**), the lysophospholipase catalytic domain and phospholipase A2 (**Table S16**). Moreover, four positively selected genes (PSGs) were detected in the quail lineage and the proteins encoded by these genes (*FSHβ*, *PLCB4*, *ITPR1* and *PLA2G4*) are involved in the *GnRH* (gonadotropin-releasing hormone) signaling pathway. Follicle stimulating hormone *beta* (*FSHβ*) protein, is a glycoprotein polypeptide hormone that, in conjunction with luteinizing hormone, contributes to growth and reproduction[50]. Transcription of the *FSHβ* gene limits the rate of production of mature *FSH*[51] which is required for ovarian folliculogenesis in females and for spermatogenesis in males in conjunction with testosterone[52]. We identified two amino acids in the quail FSHβ protein at position 37 (M→F/L) and position 99 (G→E/A) that were predicted to be under positive selection (**Figure 4a**). We used ELISA to measure the level of *FSHβ* protein during early developmental stages and found that the level of *FSHβ* in early maturing quail blood is consistently higher than that in chicken ($P<0.05$) (**Figure 4c**).

We used SWISS-MODEL to model the structure of quail *FSHβ* using the Follitropin subunit beta (4ay9.1.B) protein[53] as a template. These two amino acid substitutions were mapped to the 3D protein structure and were located near the *β*-pleated sheet that interacts with the *FSH* receptor (**Figure 4b**). *PLCB4*, *ITPR1* and *PLA2G4* (**Figure S15-17**), together with other molecules (e.g., inositol 1,4,5-trisphosphate, diacylglycerol and protein kinase C) stimulate release of gonadotropins including luteinizing hormone and follicle stimulating hormone[54, 55]. Gene expansions in the *GnRH* families, and also PSGs in the *GnRH* signaling pathway, are likely to be involved in the acceleration of growth and sexual maturity in the quail. Subsequently, we scanned the SNPs in the coding sequence (CDS) of the four genes within the 31 wild and domestic individuals that whether they were synonymous or non-synonymous mutations, and found that all the 83 SNPs except four were synonymous mutations. However, all the divergent alleles of SNP loci did not generally segregate according to the three subpopulations, and the domesticated and wild quails presented no large-scale selective sweeps around these genes.

Gene families related to immune system function

We identified a total of 1,587 immune response-related genes in quail (**Table S17**) by aligning the entire predicted gene set of quail against 2,257 genes that have been annotated with roles in the innate immune responses of *Homo sapiens*, *Mus musculus* or *Bos taurus* at InnateDB or Gene Ontology databases. Compared to chicken, turkey and duck, several expanded gene families were identified in quail. These included

Klf4, which is indispensable for differentiation of inflammatory monocytes[56] and negative regulation of innate immune response against several viruses in human embryonic kidney 293 cells[57] (22 copies in quail, 17 in chicken, 16 in turkey and 12 in duck); *Foxa2*, which regulates genetic programs that influence pulmonary inflammation mediated by Th2 cells[58] (13 copies in quail, seven in chicken, four in turkey and five in duck); and *ITCH*, which acts in T-helper cell differentiation and T-cell activation and tolerance[59] (7 copies in quail, three in chicken, four in turkey and two in duck). Moreover, we focused on the number of 'Immunoglobulin subtype' genes and found that there are 109 in quail, while chicken, duck, zebra finch (*Taeniopygia* g*uttata*) and turkey each had 62 or fewer immunoglobulin-related genes (62 copies in chicken, 29 in duck, 29 in zebra finch and 34 in turkey; **Figure 4d**). We detected 69 genes encoding a putative 'Reverse transcriptase or Reverse transcriptase domain' in quail but only two and four such genes in chicken and turkey, respectively (**Figure S18**). These domains are signatures of retroviruses integrated into the host genome. Next we compared the *MHC-B* region between quail and chicken (**Figure S19**) and found that there was an inversion including the genes encoding the proteins *TAP1* and *TAP2*, which transport peptides from the cytosol into the endoplasmic reticulum to bind *MHC* class I molecules that are being synthesized[60], and *BFIV21*, which encodes a protein that presents antigens such as the avian leukosis virus[61]. We also found four copies of the *BLEC2* (C-type lectin-like NK cell receptor) gene in quail, but only one in chicken. However, several other *MHC* genes (e.g., *KIFC1*, *V-BG1*and *BG2*) were not detected in the quail genome. A better understanding of

these immune-related genetic changes will help us characterize the immune response in quail and facilitate the development of targeted vaccines for quail.

Genome wide association analysis of plumage color

In order to identify sexed-linked genes conferring plumage color, we bred a set of egg-type quails with maroon or yellow plumage, which had been confirmed as sex-linked inheritance in quail and were consistent in a segregation ratio of Mendelian law in our previous investigation (*See more details in Method*). Of these, we sampled 40 quails, including 20 male quails and 20 female quails, and re-sequenced their genomes for case-control analysis (**Table S18**). We identified ~20M bi-allelic SNPs in these 40 individual quails at a sequencing depth of 20–30×. After controlling for SNP quality and redundant LD (*see more details in Methods*), a final total of 864,292 SNPs was retained for subsequent analyses. A genome similarity test of the 40 quail samples was conducted using high-quality SNPs, and we found that similarities between any pair of individuals ranged from 70.4 to 86.5%, which indicated relatively high homology between them.

Due to the relatively close relationships between the 40 samples, the effect of relativeness matrix affecting the variance of plumage color would be considered as the covariance. We assessed the relationships matrix of the 40 samples by using GEMMA v0.94[62] and adopted the linear mixed model for association analysis. By Bonferroni correction, the association analysis showed that two SNPs, 61102026 on chromosome 1 and 23173971on chromosome Z had significant effects on plumage

color (adjusted *P*=0.028 and *P*=0.019, respectively) (**Figure 5a**, **Figure S20** and **Table S19**). However, unlike the locus on chromosome 1, SNPs on chromosome Z near 23173971 showed a continuous peak on the Manhattan plot. In our previous analysis of plumage color heredity, we suggested that the locus on chromosome Z was most likely associated with plumage color. In a confirmation study, we added the 21 previous re-sequenced quails with “maroon” plumage (including 10 wild quails and 11 egg-type quails, **Table S10**) to rerun the association analysis for the two loci. The locus on chromosome Z was found to be more significantly associated with plumage color than before (adjusted *P*=0.015). Conversely, there was no significant signal on chromosome 1 (adjusted *P*-value for SNP 61102026 fell to 1.000). Subsequently, the SNP 23173971 on chromosome Z, we found, was located closely to gene Coiled-coil domain-containing 171 (*CCDC171*) with a length of 135 kb. Therefore, we chose SNP 23173971 on chromosome Z as the index SNP within the region of 200 kb for conditional haplotype-based association testing. Then, 47 SNPs with r^2>0.7 and adjusted *P*>0.01 had been clumped for association testing. Using 5,000 permutations, a high linked haplotype with a range of 182 kb could significantly explain the maroon/yellow variation (χ^2=37.7, *P*=8.563e-06). The well-known *TYRP1* gene that be conferred variation plumage color[47, 63] was located approximately 531 kb away from *CCDC171* (**Figure 2c**). The average of LD value between them was estimated at less than 0.2. It was suggested that the gene controlling plumage color in our population was different from *TYRP1*. Furthermore, we chose eight SNPs significantly associated with plumage color, five of which are located within

CCDC171, and designed PCR primers to amplify these SNP markers to genotype further 100 “maroon” and 100 “yellow” quails. Interestingly, 99.75% of these SNPs were consistent between genotype and phenotype, suggesting that the *CCDC171* gene controls plumage color in quail (**Figure 5b**). Therefore, we cloned the *CCDC171* gene from yellow and maroon quail and found that this gene encoded different transcripts in quail depending on plumage color (**Figure 5c** and **Figure S21**). To examine the nature of the *CCDC171* genetic variant, we characterized the transcripts from the maroon and yellow alleles. The transcript from yellow was longer than maroon’s (about 232 bp) at the upstream of the translation initiation site of maroon and has a deletion (147 bp) at position 787. In addition, we examined the differential expression of *CCDC171* in yellow and maroon quails and found there were no significant difference between the collected samples (*t*-test, $P>0.05$).

Discussion

Birds represent the most widespread class of domesticated animals in the world and are the subjects of many evolutionary, biological and pathology studies that illustrate the relationships among these avian species[27]. The timing of sexual maturity is critical for both plants and animals. Quail have a unique maturation program compared with other birds and reach sexual maturity in a very short time. We detected four promising genes for this trait under positive selection in the *GnRH* signaling pathway in quail. Gonadotropins act on the testis and ovary to promote their development and the production of steroid hormones[64]. Further functional analysis

of these genes should provide new insights into the genetic mechanisms that regulate avian sexual maturity.

Analyzing the genes and mutations related to the development and evolution of agronomic traits in quail will also improve our understanding of the genetics of domestication. Genome-wide comparisons of domesticated (egg-type and meat-type lines) and wild quail identified several footprints of artificial selection. These selective-sweep regions harbor candidate genes associated with important agro-economic traits. Genetic variations in these genes will be a rich resource for improving quail egg and meat production *via* genetic selection. It is noting that egg-type and meat-type quails didn't share selective sweep regions when compared with wild quails (**Figure S11**). It is meaning that egg-type/meat-type quails might be independently selected after domestication or two separate domestication events in quails. Further studies would be carried out for the domestication history of quails. Based on resequencing data, we have also identified a haplotype that is completely correlated with the control of "maroon/yellow" plumage color, a trait that has been used extensively in the breeding of domestic quail as a sex-linked marker.

Some recent studies[65] which use several gene data also support the phylogeny of among the *Perdicinae*, *Meleagridinae* and *Phasianinae* families. However, lack of genome-wide data cannot make strong conclusions. We used whole-genome sequences of Japanese quail, turkey and chicken to represent each clade and resolve the phylogenetic relationships among the *Perdicinae*, *Meleagridinae* and *Phasianinae* families. Our study provided a fully resolved branches with genome-scale data. Our

results support a split of the *Perdicinae* and *Phasianinae* branches from the *Meleagridinae* branch about 69 MYA. Calibration based on fossils of early penguins together with mitochondrial genome sequences of a modern albatross (*Diomedea melanophris*), petrel (*Pterodroma brevirostris*) and loon (*Gavia stellata*) allowed the divergence time of the *Anseriformes* and *Galliformes* to be estimated as 77.1± 2.5 MYA[5]. Other recent avian genome data was used to estimate the divergence of *Anseriformes* and *Galliformesat* about 66 MYA[66]. The resolution of their phylogeny will improve our understanding of the genetics of speciation of quail, chicken and turkey. In this research, we obtained a high quality draft of the Japanese quail genome and whole-genome resequencing data of multiple quail sub-populations which will provide new opportunities to further understand quail biology and develop molecular markers for improving economically important agronomic traits.

Conclusions

In conclusion, we accomplished genome assembly of quail with high-depth sequence and carried out resequencing for 71 domestic and wild quail. We solved the long-term arguments of phylogeny of quail, turkey and chicken and interpreted the biological mechanism of very early sexual maturity for quail. From the GWAS analysis, we detected a haplotype marker on chromosome Z which is important for quail breeding. These analyses should provide valuable resource and information for the future studies for quail.

Materials and Methods

Animal samples collection

All the 31 wild and domestic quails were collected from China. Of these, the 10 wild quails were sampled from the common habitats of wild quails in Henan province and Shandong province, respectively. The other domestic quails were offered from some of local breeding companies for quail improvement of different provinces (**Table S10**). The maroon or yellow plumage population was respectively derived from two pure lines offered from Hubei Shendan Healthy Food Co., Ltd. In our previous investigation, we found that the F_2 population from a crossing of the maroon plumage line and the yellow plumage line showed a 3:1 segregation ratio in plumage color. Thus, we randomly chose 20 yellows and 20 maroons from the pure lines respectively for association study (**Table S18**). All the 71 quail samples were used for re-sequencing by BGI-Shenzhen. Additionally, the 100 maroon ones and the 100 yellow ones were also derived from the two pure lines for validation of plumage color gene.

Genome sequencing and assembly

A female quail was used for all genome sequencing. All experiments in this project were performed according to the principles of the animal ethics committee at BGI (China). DNA samples were isolated from blood following standard molecular biology techniques. A series of libraries of different insert sizes ranging from 170 bp to 40 kb (170 bp, 500 bp, 800 bp, 2 kb, 5 kb, 10 kb, 20 kb and 40 kb) were constructed and used for a shotgun sequencing strategy. The Illumina HiSeq 2000

system was used to generate paired-end reads. A total of 262Gb of raw data was obtained and reads were filtered based on the following criteria. Reads with 1) >10 percent unidentified (N) bases, 2) >40% low-quality bases, or 3) contaminated by adaptors or duplicated during PCR were discarded; about 199Gb of clean data remained. The genome size (G) of quail was first estimated at about 1.1Gb using the 17-mer depth frequency distribution method: G = K-mer_num/Peak_depth (**Figure S1**). The genome was assembled using *SOAPdenovo*2 v2.04.4[29].Next, paired-end reads were then mapped back to the initial assembled genome to link contigs into long scaffolds.

Genome annotation

RepeatMasker v4.0.5[67] and Repeat-ProteinMask v4.0.5 were used to search for TEs against the RepBase library v20.04[68] to detect known repeats. A custom TE library was then constructed using RepeatModeller v1.0.8 and LTR_FINDER v1.0.6[69] for *denovo* detection of repeats. Tandem Repeat Finder v4.0.7[70] was also used to predict tandem repeats. Final results of TE detection were integrated using in-house scripts.

Homology-based and *ab initio* gene prediction methods, assisted by transcriptome sequencing, were used to analyze coding DNA sequences and to model genes. Initially, protein data for *Homo sapiens* (human), *Meleagris gallopavo* (turkey), *Gallus gallus* (chicken), *Taeniopygia guttata* (zebra finch) and *Anas platyrhynchos* (duck) were downloaded from the Ensembl v80 database[71] and aligned to the predicted proteins encoded by the quail genome using BLAT[72]. GeneWise

v2.2.0[73] was then used to further improve the accuracy of alignments and predict gene structures. AUGUSTUS v3.1[74] and GENSCAN v1.0[75] were then used for *ab initio* gene prediction. Transcriptome reads were mapped to the genome with TopHat v1.2[76] and Cufflinks v2.2.1[77] was used to confirm gene structures. Subsequently, we combined the homology-based and *denovo* predicted gene sets using GLEAN[78] and integrated the GLEAN and transcriptome results with in-house scripts to generate a representative and non-redundant gene set.

Gene evolutionary analysis

Gene families in quail, *Gallus gallus* (chicken), *Anas platyrhynchos* (duck), *Columba livia* (pigeon), *Falco cherrug* (Saker falcon), *Falco peregrinus* (Peregrine falcon), *Ficedula albicollis* (collared flycatcher), *Geospiza fortis* (medium ground finch), *Meleagris gallopavo* (turkey), *Pseudopodoces humilis* (ground tit), *Taeniopygia guttata* (zebra finch) and *Alligator sinensis* (Chinese alligator), as an outgroup, were defined using TreeFam (http://www.treefam.org/). Phylogenetic trees were then constructed using MrBayes[79] and PhyML [80] with fourfold degenerate (4D) sites of 4,393 single-copy orthologs shared among the 12 species analyzed here. Divergence times were estimated using MCMCTree (http://abacus.gene.ucl.ac.uk/software/paml.html) from the PAML package[81] together with three fossil dates from the TimeTree database[82] (http://www.timetree.org/) for calibration. Analyses of the expansion and contraction of gene families were carried out using CAFE (Computational Analysis of Gene Family Evolution)[83] using a random birth and death model with a global parameter

λ, which represents the probability of both gain and loss of a gene over a given time interval. Conditional *p*-values were calculated and defined as significant at values of less than 0.05. To detect PSGs, the coding sequences of all the single-copy orthologous genes were aligned using PRANK[84] and poorly aligned sites were removed using gBlocks[85]. High-quality alignments were then filtered to estimate the ratios ($\omega=d_N/d_S$) of nonsynonymous nucleotide substitutions (d_N) to synonymous nucleotide substitutions (d_S) for these genes in the target quail branch (ω_0), other branches (ω_1) or all branches (ω_2) using the codeml program with an improved branch-site model (TEST-II) [86] (model = 2, NSsites = 2) and the maximum likelihood method in the PAML package[81]. TEST-II is a likelihood ratio test that compares a null hypothesis with fixed $\omega=1$ with model A that allows $\omega_2>1$ in the foreground lineages. TEST-II can discriminate relaxed selective constraints analysis from positive selection and is a direct test for positive selection on the foreground lineages [87]. Positively selected sites were detected by using Bayes Empirical Bayes (BEB) method [88] which can avoid excessive false positive rate[89].

Resequencing and SNP calling

A total of 71 individuals were chosen for resequencing (see more information regarding samples). Genomic DNAs were isolated and then used to construct Illumina libraries with an insert size of 500bp. Illumina HiSeq 2000 system was used to generate paired-end reads and raw data was filtered by removing reads containing greater than 50% low-quality bases (Q value ≤5), reads containing more than 5% unidentified (N) bases and those with adapter contamination. The clean reads were

mapped to the assembled reference genome using BWA software v0.7.12[90] with parameters “-m 200000 -o 1 -e 30 -i 15 -L -I -t 4 -n 0.04 -R 20”, and the results were transformed into indexed BAM files using SAMtools v0.1.18[91]. The picard package v1.105 and Genome Analysis Toolkit (GATK, v 3.3-0)[92] were then used for SNP calling. To obtain high-quality SNPs, we: 1) deleted duplicate reads; 2) improved alignments using the IndelRealigner package in GATK; 3) recalibrated base quality scores using the BaseRecalibrator package in GATK; 4) called SNPs using the UnifiedGenotyper package in GATK with a minimum phred-scaled confidence value of 50 and a minimum phred-scaled confidence threshold of 10 for calling variants; 5) assessed variant quality using the VariantRecalibrator and ApplyRecalibration packages with truth sensitivity filter level of 99 in GATK; and 6) filtered SNPs using the VariantFiltration package in GATK with parameters “--filterExpression "QD < 2.0 || MQ < 40.0 || ReadPosRankSum< -8.0 || FS > 60.0 || HaplotypeScore> 13.0 || MQRankSum< -12.5" –filterNameLowQualFilter –missingValuesInExpressionsShouldEvaluateAsFailing”.

SNP quality control (QC)

The chromosomal variant cell format (VCF) files were transformed into PLINK format by using VCFtools v0.1.13 (https://vcftools.github.io/index.html) and subsequent analyses were performed by using PLINK v1.07[93]. As the default chromosome handling type in PLINK is for human (1:22, X, Y), the PLINK files for male quail (ZZ) and the female quail (WZ) were swapped with each other before data were analyzed because the heterogametic gender in quail is female. Additionally, the

command --dog (39n) was added at the beginning of each command line to ensure that all quail chromosomes would be included.

Individual quality control consisted of the following three steps: 1) determining the sex of individuals, 2) detecting individuals with missing genotypes and 3) identifying duplicate or highly related individuals. Any discordant sex information was checked in terms of the heterozygosity rates on the Z chromosome as described by the F statistic. That is, any individual quail for which the F-value was less than 0.8 in a male quail (ZZ) or greater than 0.2 in a female quail (ZW) would be removed from the sample set. The missing genotype rate for each individual was set to less than 10% to filter out individuals with unreliable genotype information. Case-control association studies assume that all individuals in a population are unrelated. We used a complete linkage agglomerative clustering method that was based on pairwise identity-by-state (IBS) to identify the genomic similarity of pairs of individuals. Any individual with an IBS greater than 0.9 would be filtered out of the sample set.

SNP quality control consisted of the following four steps: 1) estimating the missing genotype rate (MGR) for each SNP, 2) determining whether there was a significant difference in the rate of missing SNP genotypes between the case and control groups, 3) filtering out SNPs with very low minor allele frequencies (MAF), and 4) filtering out SNPs with frequencies that deviate significantly from Hardy-Weinberg Equilibrium (HWE). Filtering out the low-quality SNPs helped not only to avoid false-positives, but also to enhance our ability to identify the loci significantly associated with traits. Therefore, the criteria for filtering were

MGR>0.05, a significant difference in MGR between case and control according to *t*-test, at $P<0.05$, MAF<0.05 and a *P*-value for deviation from HWE<0.0001.

Extensive genome-wide regions of high linkage disequilibrium (LD) in quail strongly influenced the results of population structure, principal component and association analyses. Thus, we pruned out the pairwise SNPs with r^2 values of greater than 0.2 in each 50-bp sliding window, and set 10-bp steps for sliding window analysis to ensure 80% overlaps between any two adjacent windows.

Population structure analysis

The phylogenic tree was constructed using the neighbor joining method in MEGA v6.0[94] based on pairwise distance matrix, which were estimated using IBS distances in PLINK v1.07. Analysis of population stratification was conducted by performing complete linkage clustering of individuals using autosomal genome-wide SNP data in PLINK. Principle component analysis (PCA) was carried out using the smartpca script (https://github.com/argriffing/eigensoft/blob/master/bin/smartpca.perl) and the scatter plots were drawn by using R v3.2.2 (https://www.r-project.org/). We used ADMIXTURE v1.3[37] to analyze population structure, which uses the likelihood model-based manner from large autosomal SNP genotype datasets. The number of populations (*K*) was set from $K = 2$ to 7 to obtain the maximum likelihood estimates that would allow us to infer population structure. The cross-validation procedure was performed to exhibit a low cross-validation error, which made it fairly clear that what the optimal *K* value was. The parameter standard errors were estimated by using 100 bootstrap replicates. The cross-validation plot was drawn by using R

v3.3.2. The average LD of a pair of SNPs in a 300-kb sliding window was estimated by using PopLDdecay v2.69 (https://github.com/BGI-shenzhen/PopLDdecay), and the LD decay curves for the three populations were drawn by using R v3.3.2.

Calculation of Nucleotide diversity and estimation of population differentiation using F_{ST}

Watterson's estimator θ_w[35] and the average number of pairwise differences per sequence estimator π[36] were calculated using in-house Perl scripts. Tajima's *D*[36] was estimated using θ_w, π and the number of sequences. We scanned the whole genome to calculate the three estimators by using the 90% overlapped sliding window with size of 100 kb or 50 kb. The fixation index (F_{ST}), a measure of population differentiation due to genetic structure[95], was estimated by using VCFtools v0.1.13 also with 50-kb and 100-kb 90% overlapped sliding window on a genome-wide scale.

Association analysis and conditional haplotype-based association testing

The post-QC data was saved as PED format and later was used for GWAS via GEMMA v0.94. The centered relatedness matrix was calculated with the parameter (–gk 1). And then, the relatedness matrix was considered as a covariance using a linear mixed model to perform the Wald test, likelihood ratio test and score test. The GWAS results were shown as a Manhattan plot and Q-Q plot and were drawn by using the *qqman* package in R v3.2.2. SNP with the most significant effect on phenotypic variation was regarded as the index SNP, and the flanking 100-kb region of the index SNP was scanned for haplotype construction. In this region, the SNPs with high LD (r^2>0.7) and significantly associated to plumage color (adjusted P<0.01) were

grouped into a clump. Then, the SNPs gathered in a clump were extracted by using PLINK v1.07 and transformed into Haploview format for conditional haplotype association testing. The haplotypes in block were estimated with permutation of 5,000, and the LD plot between gene *CDCC171* and *TYRP1* was drawn using Haplotype v4.2[96].

Molecular experiments

(a) Follicle-Stimulation Hormone (FSH) testing by ELISA

We selected 100 male quails, 100 female quails, 100 hens and 100 cocks from Hubei in China and they were raised under the same conditions. In four populations, blood samples from 10 individuals were collected every week (0-6 weeks). And then the serums were separated from the blood and stored in -20℃ for testing. The *FSH* hormone of quails and chicken were tested using Follicle-Stimulation Hormone (FSH) ELISA Kit (abcam, UK) followed the protocol.

(b) *CCDC171* transcripts cloning, expression

We designed three and four pairs of primers (**Table S20**) to clone transcripts of *CCDC171* in yellow and maroon quails respectively. For researching the differential expression of *CCDC171* in yellow and maroon quail, we collected hair follicle, skin of back and skin of abdomen from three “yellow” quail and three “maroon” quail respectively. Two pairs of primers were designed to detect the differential expression of *CCDC171* by qPCR.

(c) Validation of SNPs

DNA from the different plumage quails was extracted from blood samples following standard molecular biology techniques and stored in -80℃, and used software Primer 6.0 for designing primers to validate the eight SNPs which were significantly associated with plumage color. The PCR products were sequenced by sanger platform

Additional files

Additional file 1: Figures S1-21, Tables S1-10, Table S16, Tables S18-20.

Additional file 2: Table S11. Diversity statistics of 31 quails.

Additional file 3: Table S12. The large scale of genomic regions showing reduction of diversity (ROD) between egg-type quail and wild quail at 100-kb overlapping sliding window in 10-kb step.

Additional file 4: Table S13. Functional enrichment for selective sweep regions between egg-type and wild quails.

Additional file 5: Table S14. The large scale of genomic regions showing reduction of diversity (ROD) between meat-type quail and wild quail at 100-kb overlapping sliding window in 10-kb step.

Additional file 5: Table S15. Functional enrichment for selective sweep regions between meat-type and wild quails

Additional file 6: Table S17. Copy numbers of innate immune response-related genes among quail, chicken, turkey and duck.

Data availability

The assembled genome data is accessible through NCBI BioSample: SAMN05787330 and the resequencing data has been uploaded to NCBI(unpublicized).

Acknowledgements

We would like to thank Dr. Leif Andersson, Dr. SangeetLamichhaney, Dr. Susanne Bornelöv and Prof. Maxwell Hinckegave comments for this manuscript. This project was supported by the Agricultural Science and Technology Innovation Center of Hubei (2012tsxk09 and 2014jzxjh02) and the Key Laboratory of Animal Embryo and Molecular Breeding of Hubei (2014-2017 ZD146).

Authors' contributions

Y.W. was responsible for collecting samples for sequencing, carrying out experimental verification and co-drafting the manuscript. Y.Z. made contributions to genome annotation, phylogenetic analysis, immune-related gene analysis, detecting genes under positive selection, SNP calling in re-sequencing data and co-drafting of the manuscript. Z.H. and J.P. designed the scientific objectives and co-drafted manuscript. J.C. carried out SNP quality control, GWAS analysis, detecting selective sweep regions and co-drafting of the manuscript. G.F. participated in genome assembly, analyzing inversions and co-drafting of the manuscript. W.C., X.C., J.J., X.F. and X.X. participated in project management and manuscript revision. H.L., J.S., A.P., Y.P., Z.L., H.Z., J.S., C.Z., H.T., H.X. and C.L. worked on sample preparation and experimental verification. H.Z., T.Y., B.W., H.Y. and M.B. took part in data processing. D.B. and W.W. provided suggestions and revised the manuscript. N.Y.,

X.L. and J.D. designed this project, provided suggestions and revised the manuscript.

Competing interests

The authors declare that they have no competing interests.

Author details

[1]Institute of Animal Husbandry and Veterinary, Hubei Academy of Agricultural Science, Wuhan 430064, China

[2]BGI-Shenzhen, Shenzhen 518083, China

[3]Hubei Key Laboratory of Animal Embryo and Molecular Breeding, Wuhan 430064, China

[4]National Engineering Laboratory for Animal Breeding and MOA Key Laboratory of Animal Genetics and Breeding, China Agricultural University, Beijing 100193, China

[5]Hubei Shendan Healthy Food Co., Ltd., Wuhan 430206, China

[6]School of Life Science and Technology, University of Electronic Science and Technology of China, Chengdu 610054, China

[7]State Key Laboratory of Quality Research in Chinese Medicine and Institute of Chinese Medical Sciences, Macao, China

[8]The Roslin Institute and Royal (Dick) School of Veterinary Studies, University of Edinburgh, Midlothian EH25 9RG, UK

[9]Kunming Institute of Zoology, Chinese Academy of Sciences (CAS), Kunming, China

[10]China National GeneBank-Shenzhen, BGI-Shenzhen, Shenzhen 518083, China

[11]James D. Watson Institute of Genome Sciences, Hangzhou 310058, China

[*]These authors contributed equally to this work.

†Correspondence should be addressed to Jinping Du (ddjinpin@163.com), Xin Liu (liuxin@genomics.cn) Ning Yang (nyang@cau.edu.cn) or ZhuoCheng Hou (zchou@cau.edu.cn).

Figure legends

Figure 1. Chromosomes of quail. (**a**) Gene and TE density of each quail chromosome. (**b**) Comparison of the chromosome lengths of quail and chicken.

Figure 2. Comparative evolutionary analysis of 12 avian species. (**a**) The phylogenetic tree of *Coturnix japonica* (quail), *Gallus gallus* (chicken), *Anas platyrhynchos* (duck), *Columba livia*(pigeon), *Falco cherrug* (Saker falcon), *Falco peregrinus* (Peregrine falcon), *Ficedula albicollis* (collared flycatcher), *Geospiza fortis* (medium ground finch), *Meleagris gallopavo* (turkey), *Pseudopodoces humilis*(ground tit), *Taeniopygia guttata* (zebra finch), with *Alligator sinensis* (Chinese alligator) as an outgroup. (**b**) Syntenic relationships between the quail and chicken genomes. (**c**) An inversion detected in chromosome Z between quail and chicken.

Figure 3. Analyses of the phylogenetic relationships, population structure, LD decay and genetic diversity between wild and domesticated quail. (**a**) Evolutionary history was inferred using the Neighbor-Joining method in MEGA 6.0. (**b**) Principle component analysis of wild quail and domesticated quail. (**c**) LD decay curves were estimated by squared pairwise correlations of alleles against physical distance in wild quail, egg-type quail and meat-type quail, respectively. (**d**) Population structure analysis with the maximum likelihood score for the model K = 2. (**e**) Nucleotide diversity between wild quail and egg-type quail across chromosome Z. Both the wild quail (red line) and the egg-type quail (green line) showed of the difference of diversity on chromosome Z. Plotting of Tajima's D for the egg-type group (blue line) in a 100-kb sliding window in 10-kb steps revealed the selective signal on chromosome Z. Likewise, plotting Weir's F_{st} (black line) on chromosome Z indicates the level of differentiation between the wild group and the egg-type group. Both of gene *CCDC171* and *TYRP1* were located within a selective sweep region (from ~21.5 Mb to 23.2 Mb), in which the positive signal was detected in the egg-type group. However, they exhibited a weak linkage due to the location on the different

haplotype blocks.

Figure 4. Genes related to early sexual maturity and immune system function in quail and another avian species. (**a**) Positions of amino acids under positive selection in the follicle stimulating hormone beta (FSHβ) protein. (**b**) Location of two amino acids under positive selection on the predicted 3D structure of the FSHβ protein. (**c**) Circulating FSHβ levels in blood during early development stages of quail and chicken for 6 weeks. (**d**) Phylogenetic tree of Immunoglobulin-like and Immunoglobulin subtype proteins of quail, chicken, duck, turkey and zebra finch.

Figure 5. GWAS analysis of quail plumage color. (**a**) Manhattan plot of each chromosome showing the GWAS results for quail plumage color. (**b**) Validation of eight candidate SNPs in 200 random individual quail. **(c)** The clones of *CCDC171* gene transcripts from quail with “yellow” and “maroon” plumage.

Tables

Table 1 Statistics of SNPs in whole genome and genic regions of wild and domesticated quail

References

1. Dalloul RA, Long JA, Zimin AV, Aslam L, Beal K, Blomberg LA, et al. Multi-platform next-generation sequencing of the domestic turkey (Meleagris gallopavo): genome assembly and analysis. PLoS Biol. 2010;8 9:e1000475.
2. Wallis JW, Aerts J, Groenen MA, Crooijmans RP, Layman D, Graves TA, et al. A physical map of the chicken genome. Nature. 2004;432 7018:761-4.
3. Kawahara-Miki R, Sano S, Nunome M, Shimmura T, Kuwayama T, Takahashi S, et al. Next-generation sequencing reveals genomic features in the Japanese quail. Genomics. 2013;101 6:345-53.
4. Jetz W, Thomas G, Joy J, Hartmann K and Mooers A. The global diversity of birds in space and time. Nature. 2012;491 7424:444-8.
5. Slack KE, Jones CM, Ando T, Harrison GA, Fordyce RE, Arnason U, et al. Early penguin fossils, plus mitochondrial genomes, calibrate avian evolution. Mol Biol Evol. 2006;23 6:1144-55.
6. van Tuinen M and Dyke GJ. Calibration of galliform molecular clocks using multiple fossils and genetic partitions. Mol Phylogenet Evol. 2004;30 1:74-86.
7. van Tuinen M and Hedges SB. Calibration of avian molecular clocks. Mol Biol Evol. 2001;18 2:206-13.
8. Kayang BB, Fillon V, Inoue-Murayama M, Miwa M, Leroux S, Fève K, et al. Integrated maps in quail (Coturnix japonica) confirm the high degree of synteny conservation with chicken (Gallus gallus) despite 35 million years of divergence. BMC Genomics. 2006;7 1:1.
9. Shibusawa M, Minai S, Nishida-Umehara C, Suzuki T, Mano T, Yamada K, et al. A comparative cytogenetic study of chromosome homology between chicken and Japanese quail. Cytogenet Genome Res. 2002;95 1-2:103-9.
10. Hazard D, Couty M, Faure J and Guémené D. Relationship between hypothalamic-pituitary-adrenal axis responsiveness and age, sexual maturity status, and sex in Japanese quail selected for long or short duration of tonic immobility. Poult Sci. 2005;84 12:1913-9.
11. Siopes T. Initiation of egg production by turkey breeder hens: Sexual maturation and age at lighting. Poult Sci. 2010;89 7:1490-6.
12. Caetano-Anolles K, Seo M, Rodriguez-Zas S, Oh J-D, Han JY, Lee K, et al. Comprehensive Identification of Sexual Dimorphism-Associated Differentially Expressed Genes in Two-Way Factorial Designed RNA-Seq Data on Japanese Quail (Coturnix coturnix japonica). PLoS ONE. 2015;10 9:e0139324.
13. Ricklefs R. Patterns of growth in birds. II. Growth rate and mode of development. Ibis. 1973;115 2:177-201.
14. Cécilia H, Catherine Gh, Sophie L and Jean‐Pierre R. Daily organization of laying in Japanese and European quail: Effect of domestication. J Exp Zoolog A Comp Exp Biol. 2004;301 2:186-94.
15. Mills AD, Crawford LL, Domjan M and Faure JM. The behavior of the Japanese or domestic quail Coturnix japonica. Neurosci Biobehav Rev. 1997;21 3:261-81.
16. Andersson MB. Sexual selection. Princeton University Press; 1994.
17. Hill G and McGraw K. Bird coloration. Function and evolution, vol. 2. Cambridge, MA: Harvard University Press, 2006.
18. Theron E, Hawkins K, Bermingham E, Ricklefs RE and Mundy NI. The molecular basis of an avian plumage polymorphism in the wild: a melanocortin-1-receptor point mutation is perfectly

associated with the melanic plumage morph of the bananaquit, Coereba flaveola. Curr Biol. 2001;11 8:550-7.

19. Mundy NI, Badcock NS, Hart T, Scribner K, Janssen K and Nadeau NJ. Conserved genetic basis of a quantitative plumage trait involved in mate choice. Science. 2004;303 5665:1870-3.
20. Nadeau NJ, Burke T and Mundy NI. Evolution of an avian pigmentation gene correlates with a measure of sexual selection. Proc R Soc Lond B Biol Sci. 2007;274 1620:1807-13.
21. Ottinger MA, Abdelnabi M, Li Q, Chen K, Thompson N, Harada N, et al. The Japanese quail: a model for studying reproductive aging of hypothalamic systems. Exp Gerontol. 2004;39 11:1679-93.
22. Yasuo S, Ebihara S and Yoshimura T. Circadian expression of clock gene in the optic tectum of Japanese quail. Brain Res. 2004;1005 1:193-6.
23. Creuzet S, Schuler B, Couly G and Le Douarin NM. Reciprocal relationships between Fgf8 and neural crest cells in facial and forebrain development. Proc Natl Acad Sci U S A. 2004;101 14:4843-7.
24. Nain S, Bour A, Chalmers C and Smits J. Immunotoxicity and disease resistance in Japanese quail (Corturnix coturnix japonica) exposed to malathion. Ecotoxicology. 2011;20 4:892-900.
25. Kimball RT and Braun EL. Does more sequence data improve estimates of galliform phylogeny? Analyses of a rapid radiation using a complete data matrix. PeerJ. 2014;2:e361.
26. Pereira SL and Baker AJ. A molecular timescale for galliform birds accounting for uncertainty in time estimates and heterogeneity of rates of DNA substitutions across lineages and sites. Mol Phylogenet Evol. 2006;38 2:499-509.
27. Kaiser VB, van Tuinen M and Ellegren H. Insertion events of CR1 retrotransposable elements elucidate the phylogenetic branching order in galliform birds. Mol Biol Evol. 2007;24 1:338-47.
28. Guan X, Silva P, Gyenai KB, Xu J, Geng T, Tu Z, et al. The mitochondrial genome sequence and molecular phylogeny of the turkey, Meleagris gallopavo. Anim Genet. 2009;40 2:134-41.
29. Luo R, Liu B, Xie Y, Li Z, Huang W, Yuan J, et al. SOAPdenovo2: an empirically improved memory-efficient short-read de novo assembler. GigaScience. 2012;1 1:1.
30. Recoquillay J, Pitel F, Arnould C, Leroux S, Dehais P, Moréno C, et al. A medium density genetic map and QTL for behavioral and production traits in Japanese quail. BMC Genomics. 2015;16 1:1.
31. Zhang G, Li C, Li Q, Li B, Larkin DM, Lee C, et al. Comparative genomics reveals insights into avian genome evolution and adaptation. Science. 2014;346 6215:1311-20.
32. Nadeau N, Mundy N, Gourichon D and Minvielle F. Association of a single - nucleotide substitution in TYRP1 with roux in Japanese quail (Coturnix japonica). Anim Genet. 2007;38 6:609-13.
33. Aslam ML, Bastiaansen JW, Elferink MG, Megens H-J, Crooijmans RP, Blomberg LA, et al. Whole genome SNP discovery and analysis of genetic diversity in Turkey (Meleagris gallopavo). BMC genomics. 2012;13 1:391.
34. Guo X, Fang Q, Ma C, Zhou B, Wan Y and Jiang R. Whole-genome resequencing of Xishuangbanna fighting chicken to identify signatures of selection. Genetics Selection Evolution. 2016;48 1:62.
35. Watterson G. On the number of segregating sites in genetical models without recombination. Theoretical population biology. 1975;7 2:256-76.
36. Tajima F. Evolutionary relationship of DNA sequences in finite populations. Genetics. 1983;105 2:437-60.
37. Alexander DH, Novembre J and Lange K. Fast model-based estimation of ancestry in unrelated individuals. Genome research. 2009;19 9:1655-64.

38. Balakrishnan CN and Edwards SV. Nucleotide variation, linkage disequilibrium and founder-facilitated speciation in wild populations of the zebra finch (Taeniopygia guttata). Genetics. 2009;181 2:645-60.
39. Li M-H and Merilä J. Sex-specific population structure, natural selection, and linkage disequilibrium in a wild bird population as revealed by genome-wide microsatellite analyses. BMC Evol Biol. 2010;10 1:1.
40. Charlesworth B, Morgan M and Charlesworth D. The effect of deleterious mutations on neutral molecular variation. Genetics. 1993;134 4:1289-303.
41. Zeng K, Fu Y-X, Shi S and Wu C-I. Statistical tests for detecting positive selection by utilizing high-frequency variants. Genetics. 2006;174 3:1431-9.
42. Zeng K, Shi S and Wu C-I. Compound tests for the detection of hitchhiking under positive selection. Molecular biology and evolution. 2007;24 8:1898-908.
43. Ukena K, Ubuka T and Tsutsui K. Distribution of a novel avian gonadotropin-inhibitory hormone in the quail brain. Cell and tissue research. 2003;312 1:73-9.
44. Andrew A, Rawdon BB and Alison BC. Failure of insulin cells to develop in cultured embryonic chick pancreas: a model system for the detection of factors supporting insulin cell differentiation. In Vitro Cellular & Developmental Biology-Animal. 1994;30 10:664-70.
45. Kee AJ, Gunning PW and Hardeman EC. Diverse roles of the actin cytoskeleton in striated muscle. Journal of muscle research and cell motility. 2009;30 5-6:187.
46. Ipek A and Dikmen BY. The relationship between growth traits and egg weight in pheasants (P. colchicus). Journal of Biological and Environmental Sciences. 2007;1 3.
47. Xu Y, Zhang X-H and Pang Y-Z. Association of Tyrosinase (TYR) and Tyrosinase-related Protein 1 (TYRP1) with Melanic Plumage Color in Korean Quails (Coturnix coturnix). Asian-Australasian Journal of Animal Sciences. 2013;26 11:1518-22. doi:10.5713/ajas.2013.13162.
48. YU J, YAN L, CHEN Z, LI H, YING S, ZHU H, et al. Investigating right ovary degeneration in chick embryos by transcriptome sequencing. Journal of Reproduction and Development. 2017:2016-134.
49. Yoshimura Y and Nishikori M. Identification of apoptotic oocytes in the developing ovary of embryonic and post-hatched chicks in Japanese quail (Coturnix japonica). The Journal of Poultry Science. 2004;41 1:64-8.
50. Shen S-T and Yu JY-L. Cloning and gene expression of a cDNA for the chicken follicle-stimulating hormone (FSH)-β-subunit. General and comparative endocrinology. 2002;125 3:375-86.
51. Wang Y, Fortin J, Lamba P, Bonomi M, Persani L, Roberson MS, et al. Activator protein-1 and smad proteins synergistically regulate human follicle-stimulating hormone beta-promoter activity. Endocrinology. 2008;149 11:5577-91. doi:10.1210/en.2008-0220.
52. Apter D. Development of the hypothalamic-pituitary-ovarian axis. Ann N Y Acad Sci. 1997;816:9-21.
53. Jiang X, Liu H, Chen X, Chen P-H, Fischer D, Sriraman V, et al. Structure of follicle-stimulating hormone in complex with the entire ectodomain of its receptor. Proceedings of the National Academy of Sciences. 2012;109 31:12491-6.
54. Suh P-G, Park J-I, Manzoli L, Cocco L, Peak JC, Katan M, et al. Multiple roles of phosphoinositide-specific phospholipase C isozymes. 2008.
55. Kiesel L, Przylipiak A, Emig E, Rabe T and Runnebaum B. Stimulation of gonadotropin release by arachidonic acid and its lipoxygenase metabolites in superfused pituitary cells. Life Sci. 1987;40 9:847-51.

56. Alder JK, Georgantas RW, Hildreth RL, Kaplan IM, Morisot S, Yu X, et al. Kruppel-like factor 4 is essential for inflammatory monocyte differentiation in vivo. J Immunol. 2008;180 8:5645-52.
57. Luo W-W, Lian H, Zhong B, Shu H-B and Li S. Krüppel-like factor 4 negatively regulates cellular antiviral immune response. Cell Mol Immunol. 2016;13 1:65-72.
58. Chen G, Wan H, Luo F, Zhang L, Xu Y, Lewkowich I, et al. Foxa2 programs Th2 cell-mediated innate immunity in the developing lung. J Immunol. 2010;184 11:6133-41.
59. Aki D, Zhang W and Liu YC. The E3 ligase Itch in immune regulation and beyond. Immunol Rev. 2015;266 1:6-26.
60. Daumke O and Knittler MR. Functional asymmetry of the ATP‐binding‐cassettes of the ABC transporter TAP is determined by intrinsic properties of the nucleotide binding domains. Eur J Biochem. 2001;268 17:4776-86.
61. Fulton JE, Thacker EL, Bacon LD and Hunt HD. Functional analysis of avian class I (BFIV) glycoproteins by epitope tagging and mutagenesis in vitro. Eur J Immunol. 1995;25 7:2069-76.
62. Zhou X and Stephens M. Genome-wide efficient mixed-model analysis for association studies. Nat Genet. 2012;44 7:821-4.
63. Minvielle F, Cecchi T, Passamonti P, Gourichon D and Renieri C. Plumage colour mutations and melanins in the feathers of the Japanese quail: a first comparison. Animal genetics. 2009;40 6:971-4.
64. Nishiwaki-Ohkawa T and Yoshimura T. Molecular basis for regulating seasonal reproduction in vertebrates. J Endocrinol. 2016;229 3:R117-27. doi:10.1530/JOE-16-0066.
65. Wang N, Kimball RT, Braun EL, Liang B and Zhang Z. Assessing phylogenetic relationships among Galliformes: a multigene phylogeny with expanded taxon sampling in Phasianidae. PLoS One. 2013;8 5:e64312.
66. Jarvis ED, Mirarab S, Aberer AJ, Li B, Houde P, Li C, et al. Whole-genome analyses resolve early branches in the tree of life of modern birds. Science. 2014;346 6215:1320-31. doi:10.1126/science.1253451.
67. Tarailo‐Graovac M and Chen N. Using RepeatMasker to identify repetitive elements in genomic sequences. Current Protocols in Bioinformatics. 2009:4.10. 1-4.. 4.
68. Jurka J, Kapitonov VV, Pavlicek A, Klonowski P, Kohany O and Walichiewicz J. Repbase Update, a database of eukaryotic repetitive elements. Cytogenet Genome Res. 2005;110 1-4:462-7.
69. Xu Z and Wang H. LTR_FINDER: an efficient tool for the prediction of full-length LTR retrotransposons. Nucleic Acids Res. 2007;35 suppl 2:W265-W8.
70. Benson G. Tandem repeats finder: a program to analyze DNA sequences. Nucleic Acids Res. 1999;27 2:573.
71. Aken BL, Ayling S, Barrell D, Clarke L, Curwen V, Fairley S, et al. The Ensembl gene annotation system. Database : the journal of biological databases and curation. 2016;2016 doi:10.1093/database/baw093.
72. Kent WJ. BLAT--the BLAST-like alignment tool. Genome research. 2002;12 4:656-64. doi:10.1101/gr.229202. Article published online before March 2002.
73. Birney E, Clamp M and Durbin R. GeneWise and genomewise. Genome Res. 2004;14 5:988-95.
74. Stanke M, Keller O, Gunduz I, Hayes A, Waack S and Morgenstern B. AUGUSTUS: ab initio prediction of alternative transcripts. Nucleic Acids Res. 2006;34 suppl 2:W435-W9.
75. Burge C and Karlin S. Prediction of complete gene structures in human genomic DNA. J Mol Biol. 1997;268 1:78-94.

76. Trapnell C, Pachter L and Salzberg SL. TopHat: discovering splice junctions with RNA-Seq. Bioinformatics. 2009;25 9:1105-11.
77. Trapnell C, Williams BA, Pertea G, Mortazavi A, Kwan G, Van Baren MJ, et al. Transcript assembly and quantification by RNA-Seq reveals unannotated transcripts and isoform switching during cell differentiation. Nat Biotechnol. 2010;28 5:511-5.
78. Elsik CG, Mackey AJ, Reese JT, Milshina NV, Roos DS and Weinstock GM. Creating a honey bee consensus gene set. Genome Biol. 2007;8 1:1.
79. Ronquist F, Teslenko M, van der Mark P, Ayres DL, Darling A, Höhna S, et al. MrBayes 3.2: efficient Bayesian phylogenetic inference and model choice across a large model space. Syst Biol. 2012;61 3:539-42.
80. Guindon S, Delsuc F, Dufayard J-F and Gascuel O. Estimating maximum likelihood phylogenies with PhyML. Bioinformatics for DNA sequence analysis. 2009:113-37.
81. Yang Z. PAML: a program package for phylogenetic analysis by maximum likelihood. CABIOS 13, 555–556. Supplementary material The following supplementary material is available for this article online: Fig S. 1997;1.
82. Hedges SB, Dudley J and Kumar S. TimeTree: a public knowledge-base of divergence times among organisms. Bioinformatics. 2006;22 23:2971-2.
83. De Bie T, Cristianini N, Demuth JP and Hahn MW. CAFE: a computational tool for the study of gene family evolution. Bioinformatics. 2006;22 10:1269-71.
84. Löytynoja A. Phylogeny-aware alignment with PRANK. Multiple Sequence Alignment Methods. 2014:155-70.
85. Talavera G and Castresana J. Improvement of phylogenies after removing divergent and ambiguously aligned blocks from protein sequence alignments. Syst Biol. 2007;56 4:564-77.
86. Zhao H, Yang J-R, Xu H and Zhang J. Pseudogenization of the umami taste receptor gene Tas1r1 in the giant panda coincided with its dietary switch to bamboo. Mol Biol Evol. 2010;27 12:2669-73.
87. Yang Z. PAML 4: phylogenetic analysis by maximum likelihood. Molecular biology and evolution. 2007;24 8:1586-91.
88. Zhang J, Nielsen R and Yang Z. Evaluation of an improved branch-site likelihood method for detecting positive selection at the molecular level. Molecular biology and evolution. 2005;22 12:2472-9.
89. Suzuki Y and Nei M. False-positive selection identified by ML-based methods: examples from the Sig1 gene of the diatom Thalassiosira weissflogii and the tax gene of a human T-cell lymphotropic virus. Molecular Biology and Evolution. 2004;21 5:914-21.
90. Li H and Durbin R. Fast and accurate short read alignment with Burrows–Wheeler transform. Bioinformatics. 2009;25 14:1754-60.
91. Li H, Handsaker B, Wysoker A, Fennell T, Ruan J, Homer N, et al. The sequence alignment/map format and SAMtools. Bioinformatics. 2009;25 16:2078-9.
92. McKenna A, Hanna M, Banks E, Sivachenko A, Cibulskis K, Kernytsky A, et al. The Genome Analysis Toolkit: a MapReduce framework for analyzing next-generation DNA sequencing data. Genome Res. 2010;20 9:1297-303.
93. Purcell S, Neale B, Todd-Brown K, Thomas L, Ferreira MA, Bender D, et al. PLINK: a tool set for whole-genome association and population-based linkage analyses. Am J Hum Genet. 2007;81 3:559-75.
94. Tamura K, Stecher G, Peterson D, Filipski A and Kumar S. MEGA6: molecular evolutionary genetics

analysis version 6.0. Mol Biol Evol. 2013;30 12:2725-9.
95. Weir BS and Cockerham CC. Estimating F-statistics for the analysis of population structure. evolution. 1984:1358-70.
96. Barrett JC, Fry B, Maller J and Daly MJ. Haploview: analysis and visualization of LD and haplotype maps. Bioinformatics. 2005;21 2:263-5.

Table 1 Statistics of SNPs in whole genome and genic regions of wild and domesticated quail

Whole genome							
Autosomes	Number of SNPs	π (10^{-3})	θ_W (10^{-3})	Tajima's *D*	Non-synonymous SNPs	Synonymous SNPs	Nonsyn/Syn
Wild quail	15,163,308	5.139	7.052	-1.123	61,682	173,409	0.36
Egg-type quail	12,580,606	5.125	4.249	0.825	53,119	132,347	0.40
Meat-type quail	13,763,901	5.493	4.823	0.544	58,481	147,227	0.40
Chromosome Z							
Wild quail	409,767	1.646	3.311	-2.213	1,528	2,797	0.55
Egg-type quail	141,783	0.732	0.798	-0.449	560	921	0.61
Meat-type quail	343,645	1.848	1.741	0.180	1,288	2,196	0.59
Total	21,886,307				90,031	229,019	0.39

Genic regions	Exon				Intron			
Autosomes	Number of SNPs	π (10^{-3})	θ_W (10^{-3})	Tajima's *D*	Number of SNPs	π (10^{-3})	θ_W (10^{-3})	Tajima's *D*
Wild quail	235,091	2.729	3.472	-0.878	6,470,391	5.142	6.650	-0.935
Egg-type quail	185,466	3.154	3.379	-0.276	5,339,318	6.077	6.318	-0.256
Meat-type quail	205,708	3.114	3.472	-0.402	5,851,802	6.014	6.650	-0.416
Chromosome Z								
Wild quail	4,325	1.031	2.013	-1.967	167,808	2.115	4.393	-2.134
Egg-type quail	1,481	1.116	1.960	-1.694	56,617	2.304	4.277	-1.852
Meat-type quail	3,484	1.311	2.014	-1.341	138,653	2.826	4.394	-1.406
Total	319,052				8,929,259			

Figure

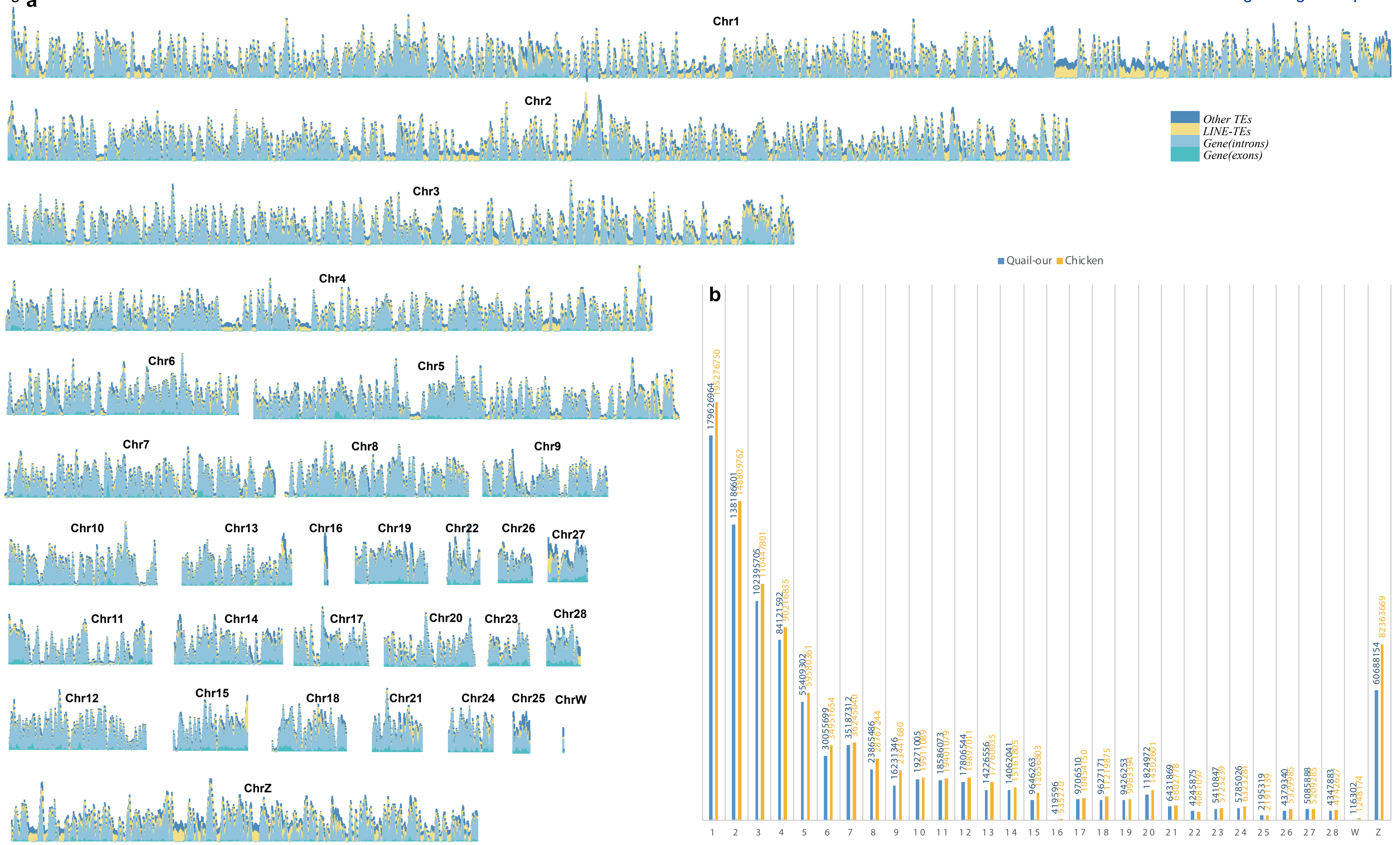
a
Chr1
Chr2
Other TEs
LINE-TEs
Gene(introns)
Gene(exons)
Chr3
Chr4
Chr6
Chr5
Chr7
Chr8
Chr9
Chr10
Chr13
Chr16
Chr19
Chr22
Chr26
Chr27
Chr11
Chr14
Chr17
Chr20
Chr23
Chr28
Chr12
Chr15
Chr18
Chr21
Chr24
Chr25
ChrW
ChrZ
b
Quail-our
Chicken
179626964
195276750
138186601
148809762
102395705
110447801
84121592
90216835
55409302
59580361
30055699
34951654
35187312
36245040
23865486
28767244
16231346
23441680
19271005
19911089
18586073
19401079
17806544
19897011
14226556
17760035
14062041
15161805
9646263
12656803
419596
535270
9706510
10454150
9627171
11219875
9426253
9983394
11824972
14302601
6431869
6802778
4245875
4081097
5410847
5723239
5785026
6323281
2195319
2191139
4379340
5329985
5085888
5209285
4347883
4742627
116302
1248174
60688154
82363669
1
2
3
4
5
6
7
8
9
10
11
12
13
14
15
16
17
18
19
20
21
22
23
24
25
26
27
28
W
Z

Figure

Click here to download Figure Figure. 2.pdf

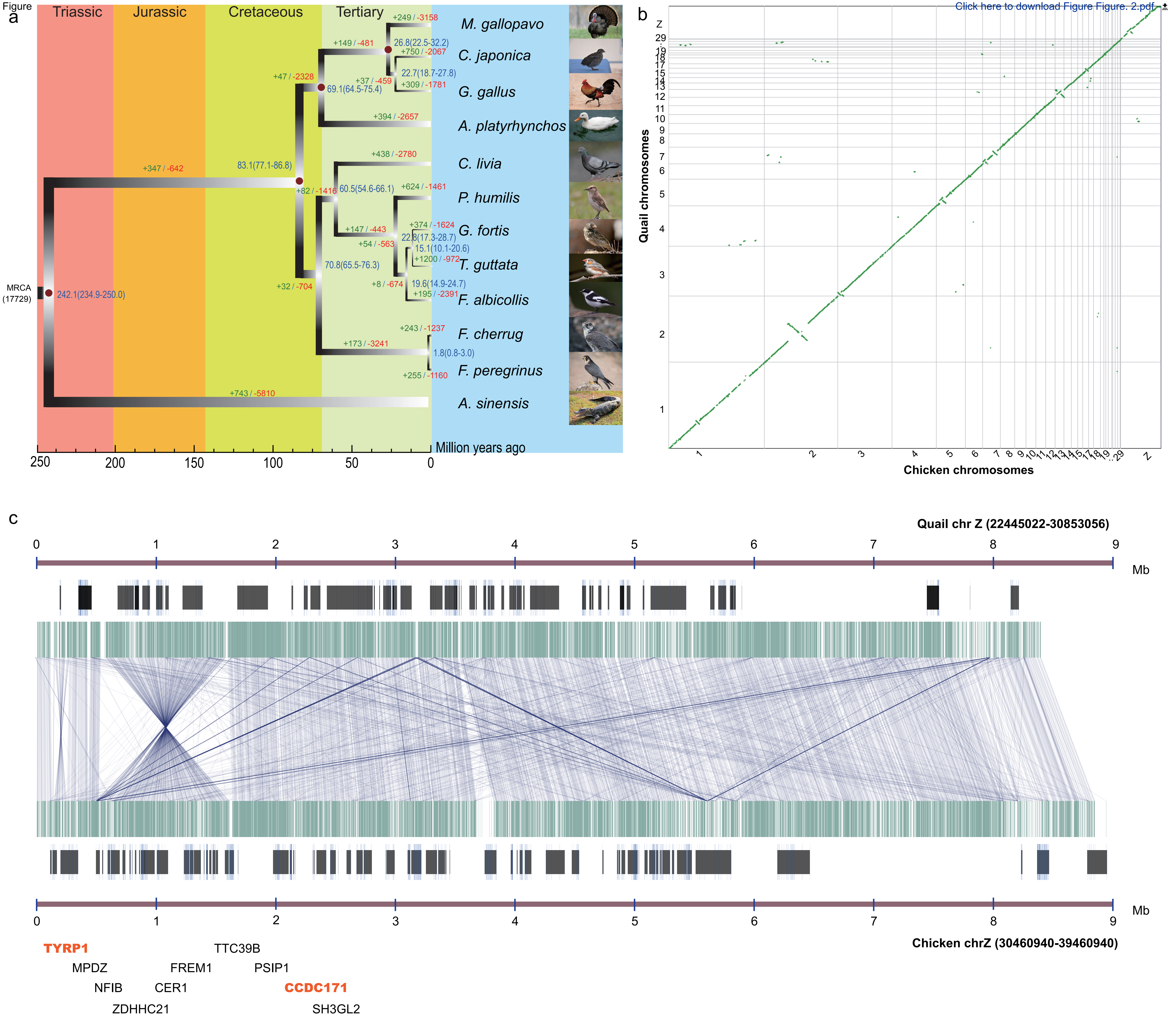

Figure

Click here to download Figure Figure. 3.pdf

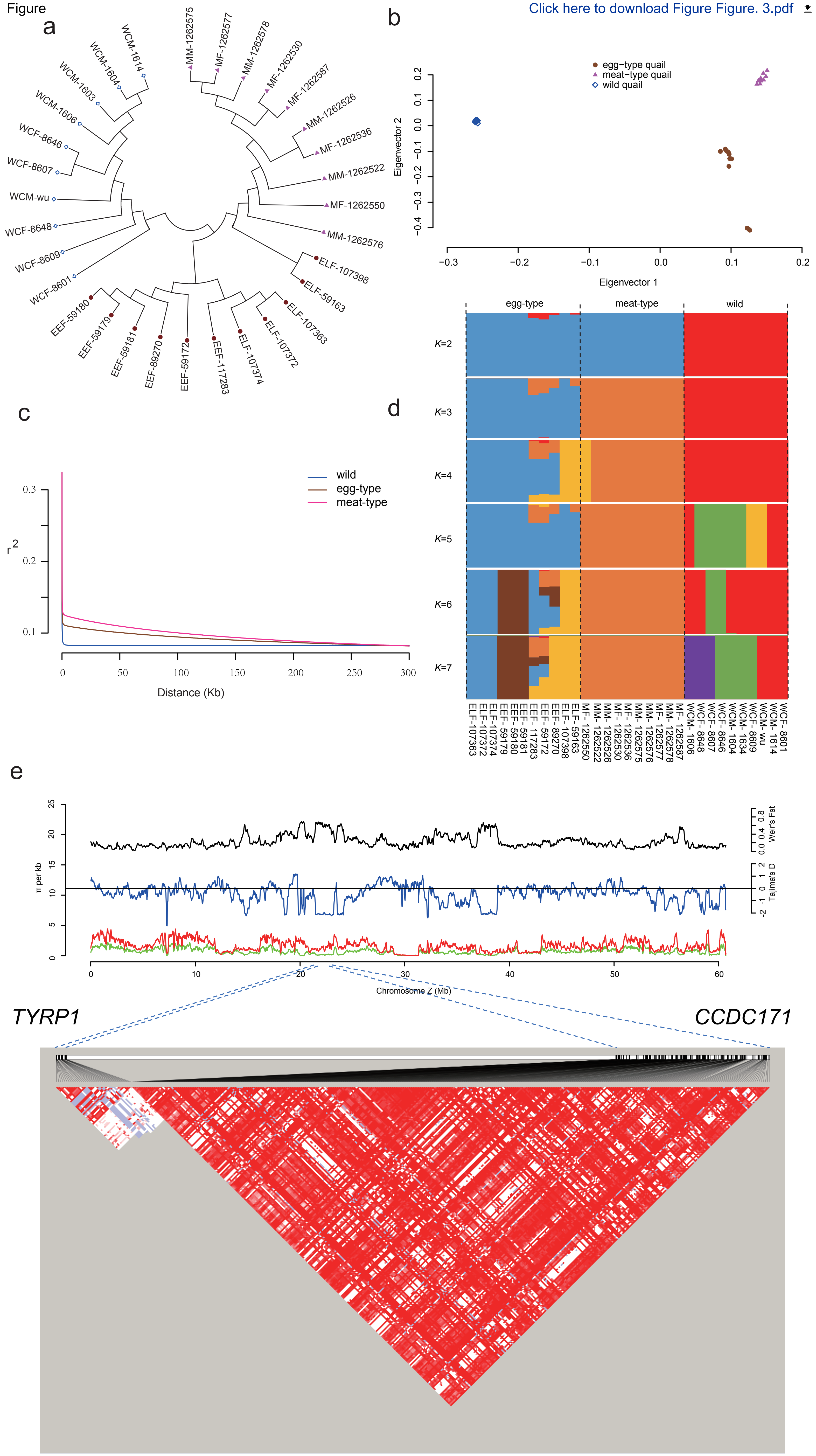

Figure
Click here to download Figure Figure. 4.pdf

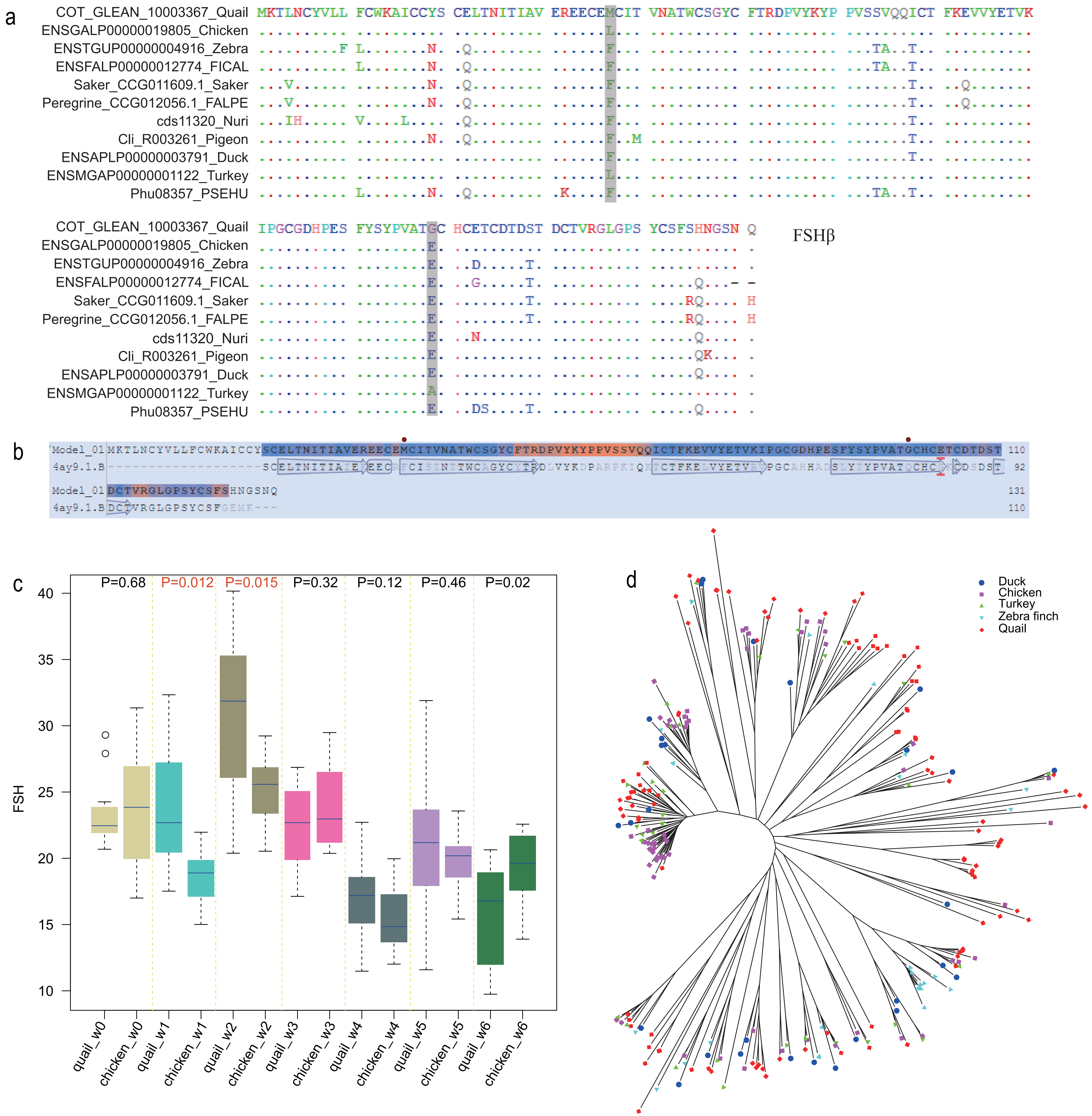

Figure

Click here to download Figure Figure. 5.pdf

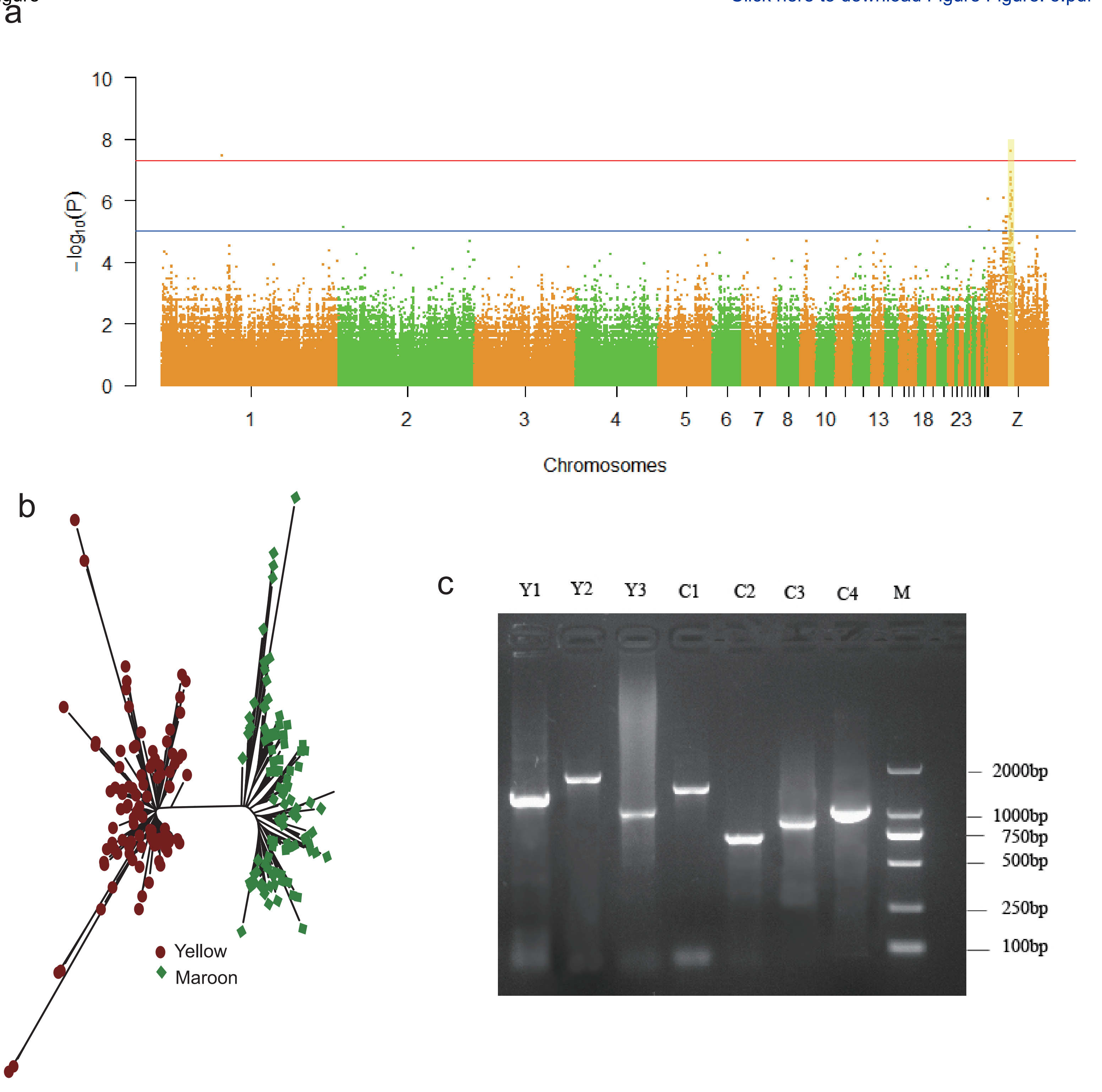

Click here to access/download
Supplementary Material
Additional file 1.docx

Click here to access/download
Supplementary Material
Table S11 diversity statistics of 31 quails.xlsx

Click here to access/download
Supplementary Material
Table S12.xlsx

Click here to access/download
Supplementary Material
Table.S13.xls

Click here to access/download
Supplementary Material
Table S14.xlsx

Click here to access/download
Supplementary Material
Table.S15.xls

Click here to access/download
Supplementary Material
Table S17.xlsx
