# Supplementary material for: Population genomic data reveal genes related to important traits of quail
Source: Gigascience. 2018 May 11;7(5):giy049. doi: 10.1093/gigascience/giy049 (PMC5961004; doi:10.1093/gigascience/giy049)
Supplement: GIGA-D-17-00216_Revision_1.pdf [file giy049_giga-d-17-00216_revision_1.pdf]

GigaScience

Population genomic data reveal genes related to important traits of quail

--Manuscript Draft--

**Manuscript Number:**	GIGA-D-17-00216R1
**Full Title:**	Population genomic data reveal genes related to important traits of quail
**Article Type:**	Research
**Funding Information:**	Study and application of functional genes in quail precocious traits based on whole genome analysis (2012tsxk09) — Dr Jinping Du<br>Molecular breeding of poultry (2014-2017 ZD146) — Dr Yan Wu
**Abstract:**	Abstract<br>Background: Japanese quail (Coturnix japonica), a recently domesticated poultry species, is important not only as an agricultural product, but also as a model bird species for genetic research. However, most of the biological questions, genomics, phylogenetics and genetics of some important economic traits, are not solved. In view of this, it is necessary to complete a high-quality genome sequence as well as series of comparative genomics, evolution and functional studies.<br>Result: Here we present a quail genome assembly spanning 1.04Gb with 86.63% of sequences anchored to 30 chromosomes (28 autosomes and two sex chromosomes Z/W). Our genomic data solved the long-term debate of phylogeny among Perdicinae (Japanese quail), Meleagridinae (turkey) and Phasianinae (chicken). Comparative genomics and functional genomic data found that four candidate genes involved in early-maturation had experienced positive selection, and one of them encodes follicle stimulating hormone beta (FSHβ), which is correlated with different FSHβ levels in quail and chicken. We re-sequenced 31 quails (10 wild, 11 egg-type and 10 meat-type) and identified 18 and 26 candidate selective sweep regions in egg-type and meat-type lines, respectively. That only one of them shared between egg-type and meat-type lines suggested that they were subject to an independent selection. We also detected a haplotype on chromosome Z which was closely linked with marron/yellow plumage in quail using population resequencing and genome-wide association study. This haplotype block would be useful for quail breeding program.<br>Conclusions: This study provided a high-quality quail reference genome, identified quail specific genes and solved quail phylogeny. We have identified genes related to quail's early-maturation and a marker for plumage color which is significant for quail breeding. The present results will facilitate biological discovery in quail and help us to elucidate the evolutionary process within the Phasianidae family.
**Corresponding Author:**	Xin Liu, Ph.D.<br>BGI<br>CHINA
**Corresponding Author Secondary Information:**	
**Corresponding Author's Institution:**	BGI
**Corresponding Author's Secondary Institution:**	
**First Author:**	Yan Wu
**First Author Secondary Information:**	
**Order of Authors:**	Yan Wu<br>Yaolei Zhang<br>Zhuocheng Hou<br>Guangyi Fan<br>Jinsong Pi

Powered by Editorial Manager® and ProduXion Manager® from Aries Systems Corporation

	Shuai Sun
	Jiang Chen
	Huaqiao Liu
	Xiao Du
	Jie Shen
	Gang Hu
	Wenbin Chen
	Ailuan Pan
	Pingping Yin
	Xiaoli Chen
	Yuejin Pu
	He Zhang
	Zhenhua Liang
	jianbo Jian
	Hao Zhang
	Bin Wu
	Jing Sun
	Jianwei Chen
	Hu Tao
	Ting Yang
	Hongwei Xiao
	Huan Yang
	Chuanwei Zheng
	Mingzhou Bai
	Xiaodong Fang
	David W. Burt
	Wen Wang
	Qingyi Li
	Xun Xu
	Chengfeng Li
	Huanming Yang
	Jian Wang
	Ning Yang
	Xin Liu, Ph.D.
	Jinping Du
**Order of Authors Secondary Information:**	
**Response to Reviewers:**	We also attached a docx file named "Respond to Reviewers.docx" which shows respond details for all Reviewers.<br><br>Responses for Reviewers' comments:

Powered by Editorial Manager® and ProduXion Manager® from Aries Systems Corporation

Reviewer #1: I have previously reviewed this manuscript for Nature Communications, and felt it was an excellent study then. It represents a large body of work and is well thought-out and executed. Previously my main points regarding the manuscript comprised of: 1) The use of PLINK for the association analysis and a recommendation to use GEMMA or GEMMAX to control for population substructure 2) Further details on the origin of the birds used for the study, in particular the origins of the different wild and domesticated breeds. 3) The conclusion for the selective sweep mapping experiments that two separate domestication events occurred due to a lack of shared sweeps (when these could be a sign of more recent 'improvement' genes being differentially selected in the broiler and layer breeds). 4) Gene expansion evidence indicating that GnRH is causal to the onset of sexual maturity in the quail. All of these points have been well addressed. The authors have used GEMMAX and provided more details on the cross. They have toned down the assertion that this is proof of two independent domestication events (they could maybe mention 'improvement selection', but this is very minor). For the gene expansion pertaining to GnRH and sexual maturity, I couldn't find the details of the numbers of different gene families that were identified as expanding, which I would like to see (as it allows the reader to evaluate how these genes in particular were targeted). They have toned down the assertion that GnRH is strongly controlling time to sexual maturity and they have also mentioned that no selective sweeps overlapped the GnRH genes as a caveat. The language could be tidied up a little in places (the usual refrain about getting a naïve reader to look through it with fresh eyes to weed these out). I am very happy with the manuscript (in fact, I was surprised it wasn't accepted by Nature Communications as I didn't feel that any of these were fatal flaws), and have no hesitation in endorsing it.

RE: First of all, the authors are all thankful of your appreciation for our work. A very short sexual maturity time is the unique characteristic among all compared birds. It means that the quail has obtained this attribute after the split of quails from other birds. This is the reason we can detect gene expansion (Table S18) and positively selected genes in the GnRH pathway; some functional studies also support our hypothesis. Selective sweep regions didn’t harbor any sexual maturity-related genes in this study. The major reason is that we compared selective regions among three different types of quails. As all quails have this unique trait, we cannot detect selective sweep regions for sexual maturity.

Reviewer #2: In this manuscript, the authors present a chromosome level assembly of the quail genome, and through comparative genomics and RNA-seq address a large variety of questions regarding the relationship of the quail to other galliformes, and identify several important and interesting genes related to various traits such as immunity, sexual maturity and plumage. This is very interesting work, containing well designed experiments and a lot of useful results. Line 61-65: references required. Or the authors need to clarify that these statements are anecdotal. RE: We have added related papers, see references 14-16. Line 78: The sentence "Some of the key avian … were included[25]." is a poorly formed sentence. I am not sure what the authors are referring to here. I assume that the authors mean that the phylogeny and genetic relationships between the three avian model systems are not well resolved.

RE: We have revised the sentence. The revised sentence is as follows: “The phylogeny and genetic relationships for some of the key avian model systems (i.e., the chicken, turkey, and Japanese quail) are not well resolved”. See line 78-79.

Line 118: What do the authors mean by consistency between the previously released genome and their assembly? From Fig S2, it looks like it is synteny, but a little more information on the statistics used to test for consistency between the genomes would be useful.

RE: We compared these two genomes at whole genome level to detect all synteny blocks which were defined as consistency regions and have already supplemented the statistic method and it is as follows “Here we employed Lastz (v1.02.00) with parameters ‘T=2 C=2 H=2000 Y=3400 L=6000 K=2200’ to compare 30 chromosomes of the two genomes and then calculated the synteny ratio of them after excluding ‘N’ bases. The figure showed syntenic relationships of blocks more than 5 kb”. See legend of Figure S2.

Line 123-126: The sentence "In addition … peak-laying (LP) (Table S4)." does not make any sense. It looks like the authors are missing some part of the sentence.

RE: We have revised the sentence. The revised sentence is as follows: “To assess the

integrity of protein-coding genes in the quail genome assembly, all transcripts assembled from RNA-Seq data sampled from the hypothalamus and ovary of three stages of quail maturity (before-laying (BL), laying (L) and peak-laying (LP)), were mapped to the assembled genome (Table S4) and ~96.33% of total complete BUSCO genes can be identified in the genome (Table S5)." See line 124-129.
Line 178-181: Number of SNPs identified seems very high implying a very high nucleotide diversity. Were the SNPs identified in the combined populations - so all 31 samples together - or were the SNPs identified using single sample callers. There are caveats in both these methods that could lead to a lot of spurious SNP calls.
RE: The method of joint variant calling, the tool of "UnifiedGenotyper" in GATK3, was used in the combined populations, which means the SNPs were identified in all 31 samples together. The high depth of sequencing data could help to reduce the probability of spurious SNPs. We have verified SNPs by PCR in the GWAS section which indicates the method is reliable (line 414-419).

Line 181-183: I was a bit confused by what the authors meant by the different number of SNPs in the different populations. Were these the number of SNPs among the ~22 million total SNPs that were polymorphic in each of the three populations?
RE: Firstly, joint variant calling was used in the combined population so we could get the total number of SNPs. Secondly, we checked whether those SNPs were polymorphic in each of the three subpopulations (meat-type, egg-type and wild) so we could get the number of SNPs for each subpopulation. See line 181-186.

Line 189-191: The differences in the ratio of non-synonymous to synonymous SNPs in the quail compared to the two other galliforms mentioned might be due to differences in sample size, quality of SNPs, the filtering criteria etc. This might indicate that this ratio is not necessarily significantly different from these other two birds.
RE: Just as you said, the differences of N/S ratio in quail population compared to chicken and turkey were influenced by many factors. We have improved the sequencing depth and more SNPs were detected. There was no strong support for the difference in N/S ratio between quail and chicken and turkey. See line 190-193.

Line 195-216: This entire paragraph needs to be rewritten for clarity. It was very difficult to follow the sequence of logical conclusions being laid out by the authors. Firstly, they mention that their estimates of θw are different in the wild quail compared to the two domestic forms for the autosomes but that their estimate of π is similar in the three populations. Their conclusion from this is that diversity in the wild quail population is quite high but that the three populations are pretty homogeneous within themselves. This conclusion can be tested by using dimension reduction methods such as PCA, MDS or even AMOVA to see if the within and between group variance support this conclusion. The Z chromosome section talking about higher selective pressure across the entire Z chromosome in the egg type quail is a bit hard to believe and looks like it might be artefactual, since all the genic regions on the Z have similarly low levels of diversity in all three populations.
RE: The entire paragraph has been rewritten for clarity as suggested. Some of the conclusions were also updated due to the increased resequencing coverage (from 3.5X to 19X). The conclusions in this part are much more concise this time. Here, we emphasize that the greater genetic diversity in the wild quail as compared to the domesticated populations. The phenomenon that genetic diversity on chromosome Z was reduced compared to the autosomes; and that this was a common finding according to previous studies. See line 196-203.
This implies that the differences on Z have to be driven by non genic regions. I would need strong evidence to believe that non-genic regions are driving selection across the entire chromosome for this population. An alternative hypothesis for the reduced diversity on the Z might lie in the demography of the egg type. The egg type is also the population with the lowest number of SNPs, indicating that possibly the effective population size of this type is lower than both the wild and meat type, and this in turn means that the Ne of the Z chromosome is lower in these and since Z is more adversely affected by the reduced Ne than the autosomes, this effect is still detectable on the Z. Finally, Tajima's D is a statistic based on the differences in θw and π, I am a bit confused that the autosomes do not show any signal in the D statistic, as the authors previously mention that the θw for the wild quail is different than that for the egg type and the meat type, while the π are similar. A clarification would be immensely helpful.

	RE: This is a similar or related question to the one above. We have rewritten these sections.<br>Lines 227-229: These sentences talk about the the clustering of the wild quail samples on PC1 and PC2. The authors claim that the clustering indicates the homogeneity in wild quail. This is completely false. The first PC is separating out the wild from the domestics and the second PC is separating the egg and meat type. In both these cases, they do not load SNPs that explain the variance within the wild quails. This close clustering of wild quail is to be expected, especially given the low sample sizes in all three groups.<br>RE: We rewrote the PCA plot result to make it clearer. We deleted the descriptions of variance within in the wild quails (homogeneity) because we agree that this is not much related to the PCA plot. See line 204-214.<br><br>Lines 234-241: Interpretation of the ADMIXTURE results. Firstly, given the admixture plots, it is clear that the admixture proportions being calculated for the 31 samples are not stable for the cases from K=4 to K=7. The ancestry clusters appear and disappear and clusters found at lower Ks are not found at higher Ks. Interpreting any of these results is in appropriate and does not lead to valid inferences. Therefore, I suggest that the authors restrict their interpretations to K=2 and K=3 which are very nice results. The slight mixing shown between egg and meat type also explains the spread along PC2 in the PCA plot.<br>RE: Interpretations of the ADMIXTURE results have been updated by restricting interpretations to K=2 and K=3 as the reviewer suggested. See line 214-228.<br>Lines 245-248: I am not sure I follow how the quick LD decay is indicative of heterogeneity in populations resulting from gene segregation and high recombination. Firstly, LD computed here is r2, which is not an indicator of recombination rates or frequencies, since it depends on the matching of allele frequencies of the two variants under consideration. Second, D or D' is a much better candidate for measuring recombination. Third, measure mean pairwise r2 in windows is highly affected by low frequency variants which by default have low r2 with other variants. A lower bound on the minor allele frequency would help in this regard. One other thing which might be causing trouble in this and other analyses might be the excess of singletons. An additional column indicating the number of singletons and the proportion of private variants in each of the three populations would be very useful in table 1.<br>RE: We have revised this conclusion. See line 228-238.<br><br>Line 265: "complete bottleneck" is a meaningless term. I think the authors mean to say strong bottleneck.<br>RE: We agree with your opinion and have replaced the word “complete” with “strong”. See line 250.<br>Lines 283-284: If the authors mean many large scale selection events in the genome, then I agree, but the sentence might need rephrasing. It now sounds like the authors claim multiple selective sweeps in the same loci, which is not possible to tell from the data they use.<br>RE: Yes, we were trying to say there were many large-scale selection events in the genome. We rephrased this part to make it clearer. See 270-272.<br>Lines 300-301: We do not need to invoke 2 domestications, but can say that the quail was probably domesticated for other traits (as mentioned earlier probably crowing), and the egg and meat traits were selected upon independently from this domesticated from starting from the early 20th century.<br>RE: That’s a very good suggestion. We rephrased the description by stating “It is worth noting that egg-type and meat-type quails did not share selective sweep regions when compared with wild quails (Figure S11), meaning that egg-type/meat-type quails might have been independently selected after domestication or that there were two separate domestication events in quails.” See line 447-451.<br><br>Lines 302-304: Did the authors perform RNA seq or similar to measure gene expression? The following paragraph (Lines 305-) does not say anything about expression but about different non-synonymous SNPs in domestics compared to the wild. Although this is very nice analysis, it has nothing to do with differential expression.<br>RE: Unfortunately, we did not have these RNAseq data.<br>Lines 335-340: The authors mention that higher FSHβ leads to reduction in mature FSH, which is required for sexual maturity. But they also mention that this FSHβ is higher in quail compared to chicken, which would imply later maturity in quail. I might

Powered by Editorial Manager® and ProduXion Manager® from Aries Systems Corporation

have missed something but this is counterintuitive.

RE：We revised the description in this sentence. FSHβ is the major gene for controlling hormone synthesis rate. It means that higher FSHβ would have higher hormone synthesis rate, and the hormone level could determine the sex maturity in animals. The revised sentence is as follows: “Transcription of the FSHβ gene is the rate-limiting step for in hormone synthesis which is required for ovarian folliculogenesis in females and for spermatogenesis in males, in conjunction with testosterone.” See line 322-324.

Lines 411-415: The additional quails added here come from different populations, which have been shown to the different before, so it would be useful to know what kind of corrections were performed for this, since the lower p-value might be an artifact of population structure in the samples.

RE：Yes, the p-value was the corrected one by Bonferroni method.

Language related comments: Line 27: "That only one of them shared" should read "That only one of them is shared"

Done.

Line 29: "marron" should reads "maroon" I think.

Done

Line 60: "than that do in males" should read "than males".

Done.

I stopped here, but there are lots of English related errors in the manuscript, and in its current form, it cannot be published due to the language issues, although the content deserves the platform.

RE: We invited native English speakers to polish the manuscript to resolve any language issues.

Reviewer #3: In the Wu et al manuscript, the authors describe the sequencing of a Japanese quail at 238 fold coverage. They generated an assembly with an N50 contig size of 27.9 kb and an N50 scaffold size of 1.8 Mb. This assembly is less complete than a separate quail assembly with an N50 contig size of 511 kb and N50 scaffold size of 3 Mb (1). The authors used their assembly to build a tree for the Phasianidae family using three different species (Japanese quail, chicken, and turkey). Their results were consistent with the Hackett et al study (2) and several other studies (3-5). These reports all show that the quail and chicken are more closely related to each other than they are to the turkey. It is unclear how impactful these results are other than they confirm previous studies.

RE: Although some investigations have been done to explore the phylogenetic relationship, there are many conflicts among the various studies. We present, for the first time, the use of whole genome data to perform the phylogenetic analysis as this can better confirm the relationship between these species.

The authors compared their quail assembly to 10 other bird genomes, along with a Chinese alligator as an outgroup. The authors did a good job resolving the divergence times of the phasianidae. Including an aniseriform was a critical inclusion of the analysis. Figure S7 showing the MrBayes and PhyML based phylogenic trees was a critical part of the study. Space permitting, the authors may consider moving this figure to the main text.

RE: We agree with your opinion. However, there is not enough space to move this figure to the main text.

The authors then undertook the task of analyzing the genetic diversity in 3 distinct quail for a total of 31 individuals. The authors sequenced approximately 10 individuals at an average of 3.5X coverage. However, there was no listing of the coverage for each individual. There could be some individuals under 1X coverage.

RE: We have increased the average sequencing coverage to ~19X of the genome. All data have been updated accordingly.

The authors did a nice analysis on gene families that potentially lead to early sexual maturity. The quail reaches maturity rapidly relative to other birds. It would be interesting to see a follow up study in a separate manuscript that compared the genes families found to the same gene families in other birds that reach sexual maturity early, like some parrots. Likewise, it would be good to compare these gene families to other birds, beyond chickens that reach sexual maturity later in life.

RE: Thank you for your opinion. We plan to do more research in the future and expect a good result.

For the GNRH1 gene, they found one extra copy compared to the other genomes they studied. They based this on a peptide region (VFLLLLWENLPPVQAGKAREGWVRLVGEKRQESLVHMWQSQLCITLGYVQEYDYIN

LDAPAVTMSLLTELKP) of the protein shown in figure S14. However, when I used this "unique" peptide sequence, I was able to find it in chicken using a blast search (see reviewer fig 1 below). I also noticed a similar observation in the PLCB4 fig S15. See my reviewer fig 2 below. It would be good if the authors could explain this. I also saw a similar observation in figure S16 (chicken protein XP_015148438) (not shown). This could be due to outdated annotations.

RE:

(1)For GNRH1 gene (Figure S14)

On the one hand, the data we used were from Ensembl release80 database and in the database, there is only one copy of this gene. On the other hand, we have checked the NCBI sequence ID X69491.1 supplied by you. The positive match may only occur at the nucleic acid level and the true translated protein is as below(MEKSRKILVGVLLFTASVAICLAQHWSYGLQPGGKRNAENLVESFQEIANEME SLGEGQKAECPGSYQHPRLSDLKETMASLIEGEARRKEI). The entire information of X69491.1 is also as below. The sequence did not contain VFLLLLWENLPPVQAGKAREGWVRLVGEKRQESLVHMWQSQLCITLGYVQEYDYINL DAPAVTMSLLTELKP.

```
G.gallus gene for gonadotrophin releasing hormone I
GenBank: X69491.1
FASTA Graphics
Go to:
LOCUS       X69491                6282 bp    DNA     linear   VRT 14-NOV-2006
DEFINITION  G.gallus gene for gonadotrophin releasing hormone I.
ACCESSION   X69491
VERSION     X69491.1
KEYWORDS    gonadotropin-releasing hormone; gonadotropin-releasing hormone-I;
            neuroendocrine  protein.
SOURCE      Gallus gallus (chicken)
  ORGANISM  Gallus gallus
            Eukaryota; Metazoa; Chordata; Craniata; Vertebrata; Euteleostomi;
            Archelosauria; Archosauria; Dinosauria; Saurischia; Theropoda;
            Coelurosauria; Aves; Neognathae; Galloanserae; Galliformes;
            Phasianidae; Phasianinae; Gallus.
REFERENCE   1  (bases 1 to 6281)
  AUTHORS   Dunn,I.C., Chen,Y., Hook,C., Sharp,P.J. and Sang,H.M.
  TITLE     Characterization of the chicken preprogonadotrophin-releasing
            hormone-I gene
  JOURNAL   J. Mol. Endocrinol. 11 (1), 19-29 (1993)
   PUBMED   7902095
REFERENCE   2
  AUTHORS   Dunn,I.
  TITLE     Direct Submission
  JOURNAL   Submitted (30-NOV-1992) I. Dunn, Institute of Animal Physiology
            and, Genetics Research, AFRC, Roslin, Midlothian EH 9PS, Scotland,
            UK
  REMARK    revised by [3]  MAT
REFERENCE   3  (bases 1 to 6282)
  AUTHORS   Dunn,I.
  TITLE     Direct Submission
  JOURNAL   Submitted (31-MAY-1994) I. Dunn, Institute of Animal Physiology
            and, Genetics Research, AFRC, Roslin, Midlothian EH 9PS, Scotland,
            UK
COMMENT     On Jun 4, 1994 this sequence version replaced gi:311611.
FEATURES             Location/Qualifiers
     source          1..6282
                     /organism="Gallus gallus"
                     /mol_type="genomic DNA"
                     /strain="white leghorn"
                     /db_xref="taxon:9031"
                     /clone="lambda52"
                     /sub_clone="EDP1"
                     /clone_lib="SAU3AI digest genomic library in EMBL3"
                     /dev_stage="adult"
     mRNA            join(2996..3047,3485..3626,4296..4391,5899..5998)
```

exon 2996..3047
/number=1
intron 3048..3484
/number=1
exon 3485..3626
/number=2
gene order(3486..3626,4296..4391,5899..5940)
/gene="cGnRH-I"
CDS join(3486..3626,4296..4391,5899..5940)
/gene="cGnRH-I"
/codon_start=1
/product="chicken gonadotrophin releasing hormone-I"
/protein_id="CAA49246.1"
/db_xref="GOA:P37042"
/db_xref="InterPro:IPR002012"
/db_xref="InterPro:IPR004079"
/db_xref="InterPro:IPR019792"
/db_xref="UniProtKB/Swiss-Prot:P37042"

/translation="MEKSRKILVGVLLFTASVAICLAQHWSYGLQPGGKRNAENLVES
FQEIANEMESLGEGQKAECPGSYQHPRLSDLKETMASLIEGEARRKEI"
intron 3627..4295
/gene="cGnRH-I"
/number=2
exon 4296..4391
/gene="cGnRH-I"
/number=3
intron 4392..5898
/gene="cGnRH-I"
/number=3
exon 5899..5998
/number=4

(2) For PLCB4 gene (Figure S15)
We have checked the Ensembl database release 84/87/89/90/91 and for example, for the duck genome, we did not find any annotation information including sequence “KKAVEDSEQEN”. All the version of annotation information is as below.
>ENSAPLP00000013480.1 pep scaffold:BGI_duck_1.0:KB742503.1:690475:780114:1 gene:ENSAPLG00000013577.1 transcript:ENSAPLT00000014232
.1 gene_biotype:protein_coding transcript_biotype:protein_coding gene_symbol:PLCB4 description: phospholipase C beta 4 [Source:HGNCSymbol;Acc:HGNC:9059]
MAKPYEFNWQKPVPSFMQDGAVFDRYEEESFVFESNCLFQVDEFGFFLSWKSEGKE
GQVLECSLINSVRFGAIPKDPKILTALEAVGKSENELEGRIVCVCSGTDLVNISFTFMVA
ENTE
IAKQWVEGLGSIIHNFRANNVSPMTCLKKHWMKLAFLTNTNGKIPVRSITRTFASGKTE
K
VIFQALKELGLPSGKNDEIEPAAFTYDKFYELTQKICPRTDIEDLFKKINGDKTDYLTVD
QLVSFLNEHQRDPRLNEILFPFYDTKRAMQIIETYEPDEDLKSKGLISSDGFCRYLMSD
E
NAPVFLDRLELYQEMDHPLAHYFISSSHNTYLTGRQFGGKSSVEMYRQVLLAGCRCV
ED
CWDGKGEDQEPIITHGKAMCTDILFKDVIQAIKETAFVTSEYPVILSFENHCSKYQQYK
M
SKYCEDLFGDLLLKQPLETHPLDPGKALPSPNDLKRKILIKNKRLKPEVEKKQLDALKK
M
MEAGETAAPVNILEDDNEEEIESADQEEEAHPEYKFGSDLTVDDYCQKEAVAISVKKG
LV
TVEDEQAWMASYKYVGATTNIHPYLSTMVNYAQPVKFQGFDVAEERNIHHNMSSFN
ESG
LGYLKTHAIEFVNYNKRQMSRIYPKGGRVDSSNYMPQIFWNAGCQMVSLNYQTPDLA
ML
NQGKFEYNGSCGYLLKPDFMRRPDRTFDPFSETPVDGVIAATCSVQVISGQFLSDKKI
GT
YVEVDMYGLPTDTIRKEFRTRMVMNNGLNPVYNEESFVFRKVILPDLAVLRIAVYDDN

Powered by Editorial Manager® and ProduXion Manager® from Aries Systems Corporation

NK
LIGQRILPLDGLQAGYRHISLRNEGNKPLSLPTVFCNIVLKTYVPDGFGDIVDALSDPKK
FLSITEKRADQMRAMGIETSDIADVPNDTSKNDRKGKANNAKANVTPQSSSELRPSTT
AG
FGSGTDAKKGIDLIAQVKIEDLKQMKAYIKHLKKQQKELNALKKKHAKEHSAMQKLHC
TQ
IDKVVAQYDKEKVSYEKMLEKAIKKKGGSNCVELKKETEIKIQALTSDHKSKVKEIVAQ
H
TKEWSEMINTHSAEEQEIRDLHLNQQCELLKKLLINAHEQQTQQLKFSHDRESKEMR
ANQ
AKISMENSKAISQDKSIKNKAERERRVRELNSSNTKKFLEERKRLAMKQSKEMDQLKK
VQ
LEHLEVLEKQNEQKQLLKSCHAVSQTQGKGDAADGEAGSRDGSQASNSGLKPQHV
N

(3) For ITPR1 gene (Figure S16)
We thought it was due to different version of annotation. We checked Ensembl database release 80/84/90/91and did not find the sequence “MEGREPPPRGSKPE”. Only in Ensembl database release 87 did we find it.

The authors then did a nice analysis of genes from the immune system. This was very insightful. Were these extra copies validated with RNAseq or IsoSeq data?

RE: We used RNAseq data to validate three gene families of Klf4 / ITCH / Foxa2 and the average coverage of all genes of each family by RNA reads was 90.32%, 91.33% and 88.23% respectively, which indicated that the expansion of gene families was real.

The authors also did an analysis of the 40 genomes they sequenced for correlation with plumage color. They found the CCDC171 as a candidate. The authors then added additional sequence data from 100 maroon and 100 yellow quails. This was an excellent addition to the study.

Suggestions * Include NCBI genome IDs for other bird genomes used, since multiple versions are available

RE: We have supplied the corresponding version ID or websites of all used genomes. See at Methods section “Gene evolutionary analysis”. See line 538-547.

* I would have not done the saker falcon since it is redundant. The Peregrine falcon would have been enough. I would have added the budgie instead. That being said, I don't think it is necessary to redo the analysis with budgie.

RE: At the initial time of analysis, we added the budgie. However, we deleted it ultimately because we identified too few single copy genes when including this bird.

* For figures S14, S15, S16, S17 were chicken IsoSeq reads used for analysis?

RE: In these figures, we used the RNAseq of quail for analysis. We did not use chicken IsoSeq reads.

* BUSCO or CEGMA should have been used to determine completeness of all the individual quail genomes.

RE: BUSCO analysis were done for assembled genome, transcriptome and gene peptide set. We did not assemble all individual quail genomes because we only mapped the resequencing reads to the reference genome. We supplied BUSCO for the reference genome (Table S5) and the coverage of each individual quail reads to the reference genome.

* Table S10 should be expanded to include the sequence coverage of all the individuals or an separate supplemental table should be created to include coverage and BUSCO summary.

RE: This is a similar question to the one above and we have supplied data in Table S11 and S12.

* An average coverage of 3.5X of the entire data set is not very telling. Please report the standard deviation for the entire dataset. Also include the % mapped reads for each individual.

RE: We have increased the sequencing coverage to about 19X and supplied the mapping information (Table S12).

* Based on the text in 217-220, I was expecting a phylogenetic tree in the supplement, rather than just a table.

RE: Figure 3a was the exact phylogenetic tree

* In figures S14-S17, the authors may want to consider using a lighter shade coloring for easier reading.

RE: We agree with your opinion while we thought the current forms of these figures were ok for reading.

Minor comments * From what population was the reference genome sequenced? Maybe I missed this?

RE: We have supplemented the information in methods section “Genome sequencing and assembly”.

* Nice decision to include a good balance of both males and females in the study.

* In table S6 you could add more information on the gene sets used for the homology based portion of the annotations. Was IsoSeq data used? IsoSeq data is available to several of the species listed in table S6.

RE: We only used RNAseq data.

* In reference to table S8, was an alignment of the quail to the turkey done and not shown?

RE: Sorry, we did not carry out the alignment with turkey because our focus was mainly on the comparison with chicken.

Major criticisms * It is unclear why the authors did not use one of the other quail genomes assemblies from other groups' efforts for mapping as opposed to creating their own. The main reference genome is not better than the recently assembly genomes. They could have spent their resources on higher coverage of the individuals in the population.

RE: More de novo assembly provides a chance for pan-genome analysis. Our new quail genome has been assembled at the chromosome level which is much more powerful when conducting GWAS-like study than the previously available genome.

* 3.5X coverage is not enough coverage. The studies that I have read in the past two years have all had at least 8-10X coverage (6-8). Please give references to articles from journals showing that 3.5X coverage is enough. As genomics is a fast moving field, please only include articles published since 2015.

RE: We have increased the sequencing coverage to about 19X to improve the accuracy of SNP calling.

* BUSCO or CEGMA was not shown to validate the quality of the reference genome. This should be in the supplement along with a comparison to the previously sequenced quail genomes. I have heard that you will report this separately. However, I would have liked to have seen the BUSCO or CEGMA analysis in this paper.

RE: We have supplied the BUSCO analysis of the reference genome. See Table S5.

Conclusion Overall, the authors did a very good job analyzing the population data they had. However, I do not think the high coverage sequencing of another reference genome added much value to the paper since the quail genome was already sequenced using a similar sequencing strategy (1). Based on the literature over the past couple years, I think studies should aim for a minimum of 10X coverage for resequencing. Given this opinion, I do not think this study is appropriate for Gigascience. However, if the editors feel 3.5X coverage is enough now or if the authors can show a number of recent papers with 3.5X or below coverage, then I will reconsider my opinion.

RE: We have increased the sequencing coverage to about 19X to improve the accuracy of SNPs calling. All data have been updated. Conclusions were drawn based on the updated data.

References 1. https://www.ncbi.nlm.nih.gov/assembly/GCA_001577835.1#/st 2. Hackett SJ, Kimball RT, Reddy S, Bowie RC, Braun EL, Braun MJ, Chojnowski JL, Cox WA, Han KL, Harshman J, Huddleston CJ, Marks BD, Miglia KJ, Moore WS, Sheldon FH, Steadman DW, Witt CC, Yuri T. A phylogenomic study of birds reveals their evolutionary history. Science. 2008 Jun 27;320(5884):1763-8. doi: 10.1126/science.1157704. 3. Wang N, Kimball RT, Braun EL, Liang B, Zhang Z (2013) Assessing Phylogenetic Relationships among Galliformes: A Multigene Phylogeny with Expanded Taxon Sampling in Phasianidae. PLoS ONE 8(5): e64312. 4. Sibley CG, Ahlquist JE (1990) Phylogeny and Classification of Birds: A Study in Molecular Evolution. New Haven: Yale University Press. 5. Kimball and Braun (2014), Does more sequence data improve estimates of galliform phylogeny? Analyses of a rapid radiation using a complete data matrix. PeerJ 2:e361; DOI 10.7717/peerj.361 6. https://www.illumina.com/science/education/sequencing-coverage.html 7. David Sims, Ian Sudbery, Nicholas E. Ilott, Andreas Heger & Chris P. Ponting. Sequencing depth and coverage: key considerations in genomic analyses. Nature Reviews Genetics 15, 121-132 (2014) 8. Burri R, Nater A, Kawakami T, Mugal CF, Olason P, Smeds L, Suh A, Dutoit L, Bureš S, Garamszegi LZ, Hogner S, Moreno J, Qvarnström A, Ružić M, Sæther SA, Sætre GP, Török J, Ellegren H. Linked

	selection and recombination rate variation drive the evolution of the genomic landscape of differentiation across the speciation continuum of Ficedula flycatchers. Genome Res. 2015 Nov;25(11):1656-65.
**Additional Information:**	
**Question**	**Response**
Are you submitting this manuscript to a special series or article collection?	No
**Experimental design and statistics**<br><br>Full details of the experimental design and statistical methods used should be given in the Methods section, as detailed in our Minimum Standards Reporting Checklist. Information essential to interpreting the data presented should be made available in the figure legends.<br><br>Have you included all the information requested in your manuscript?	Yes
**Resources**<br><br>A description of all resources used, including antibodies, cell lines, animals and software tools, with enough information to allow them to be uniquely identified, should be included in the Methods section. Authors are strongly encouraged to cite Research Resource Identifiers (RRIDs) for antibodies, model organisms and tools, where possible.<br><br>Have you included the information requested as detailed in our Minimum Standards Reporting Checklist?	Yes
**Availability of data and materials**<br><br>All datasets and code on which the conclusions of the paper rely must be either included in your submission or deposited in publicly available repositories (where available and ethically appropriate), referencing such data using a unique identifier in the references and in the "Availability of Data and Materials" section of your manuscript.<br><br>Have you have met the above requirement as detailed in our Minimum Standards Reporting Checklist?	Yes

Manuscript

Click here to download Manuscript Quail genome manuscript-Final27_4.docx

Population genomic data reveal genes related to important traits of quail

Yan Wu[1,3,6,*], Yaolei Zhang[2,5,11,*], Zhuocheng Hou[4,*], Guangyi Fan[2,5,8,11], Jinsong Pi[1], Shuai Sun[5], Jiang Chen[2,11], Huaqiao Liu[7], Xiao Du[5], Jie Shen[1], Gang Hu[5], Wenbin Chen[2], Ailuan Pan[1], Pingping Yin[5], Xiaoli Chen[2], Yuejin Pu[1], He Zhang[2], Zhenhua Liang[1], Jianbo Jian[2], Hao Zhang[1], Bin Wu[2], Jing Sun[1], Jianwei Chen[2], Hu Tao[1], Ting Yang[2], Hongwei Xiao[1], Huan Yang[2], Chuanwei Zheng[1], Mingzhou Bai[2], Xiaodong Fang[2], David W. Burt[9], Wen Wang[10], Qingyi Li[5], Xun Xu[2,11], Chengfeng Li[5], Huanming Yang[2,12], Jian Wang[2,12], Ning Yang[4], Xin Liu[2,11,†], Jinping Du[1,†]

Abstract

Background: Japanese quail (*Coturnix japonica*), a recently domesticated poultry species, is important not only as an agricultural product, but also as a model bird species for genetic research. However, most of the biological questions concerning genomics, phylogenetics and genetics of some important economic traits have not been answered. It is thus necessary to complete a high-quality genome sequence as well as a series of comparative genomics, evolution and functional studies.

Results: Here, we present a quail genome assembly spanning 1.04Gb with 86.63% of sequences anchored to 30 chromosomes (28 autosomes and two sex chromosomes Z/W). Our genomic data has resolved the long-term debate of phylogeny among Perdicinae (Japanese quail), Meleagridinae (turkey) and Phasianinae (chicken). Comparative genomics and functional genomic data found that four candidate genes involved in early maturation had experienced positive selection, and one of them encodes follicle stimulating hormone beta (*FSHβ*), which is correlated with different *FSHβ* levels in quail and chicken. We re-sequenced 31 quails (10 wild, 11 egg-type and 10 meat-type) and identified 18 and 26 candidate selective sweep regions in the egg-type and meat-type lines, respectively. That only one of them is shared between egg-type and meat-type lines suggests that they were subject to an independent selection. We also detected a haplotype on chromosome Z which was closely linked with maroon/yellow plumage in quail using population resequencing and a genome-wide association study. This haplotype block will be useful for quail breeding programs.

Conclusions: This study provided a high-quality quail reference genome, identified quail-specific genes and resolved quail phylogeny. We have identified genes related to quail early maturation and a marker for plumage color which is significant for quail breeding. These results will facilitate biological discovery in quails and help us elucidate the evolutionary process within the Phasianidae family.

Keywords: Japanese quail, genome assembly, early maturation, phylogeny, resequencing, plumage color, quail breeding

Background

Most of the poultry eggs and meat products in the world come from species that are members of the Phasianidae family including chicken (*Gallus gallus*), turkey (*Meleagris gallopavo*) and Japanese quail (*Coturnix japonica*) within the order Galliformes. The genomes of the two widely domesticated avian species, chicken and turkey, have already been sequenced [1, 2]. Accordingly, the first quail draft genome sequence was reported with the N50 contig length of 1.5 kb by Tokyo University of Agriculture in 2013. Subsequently, the same group developed an improved draft and extended the N50 contig length to 32 kb (NCBI BioSample: SAMD00009971) [3]. Recently, another chromosome-level draft genome for the Japanese quail was published by using the quail inbred line, Cons DD (INRA) (NCBI BioSample: SAMN03989050). However, with these reference quail genome assemblies, most biological questions involving genomics and phylogenetics are still unresolved.

The Phasianidae family has its origin about 30–46 million years ago (MYA) [1, 4-7]. Even though high degrees of conservation of synteny and chromosome homology have been observed between quail and chicken [8, 9], these species display a great diversity of phenotypes among the three widely used domesticated birds. Japanese quail reach sexual maturity at 5–6 weeks of age [10], while chicken and turkey reach this stage in about 18–22 weeks [11]. Body mass at the maturity stage of meat-type quail is about 10% that of broiler chicken and 2.5% that of turkey [11], yet quail have the fastest growth rate of all species in the Phasianidae family [12, 13]. Furthermore, female quails generally present a heavier body weight than males [14],

while the reverse is true both in chickens and turkeys [15]. In addition, there are very distinct differences between subpopulations of quail, even though quail branched off from the Phasianidae family fairly recently [16]. According to historical records, the domestication of Japanese quail began in the 11th century and was initially based on birds selected for their crowing abilities [16]. However, the resulting domestic strains, which were selected for commercial egg and meat productions, were improved only from 1910s [17] . Today, the domestic quail differs from the wild population in many traits, such as variations in plumage color, increased body size, acceleration of sexual maturity, lengthening of the reproductive phase, and the disappearance of migratory characteristics [18]. Because of the important roles of plumage color in signaling, mate choice and evolution, mapping the gene conferring sex-linked plumage color is significant for commercial breeding in quail [13, 17-20]. Additionally, it has been established that the quail has an advantage over the chicken concerning reproduction interval and space requirements, so the quail is also considered to be an excellent avian model for embryonic development, reproduction, sexual differentiation, environmental toxicant indication and disease resistance [19-27].

The phylogeny and genetic relationships for some of the key avian model systems (e.g. chicken, turkey, and Japanese quail) are not well resolved [28]. Even though a preliminary understanding of Phasianidae phylogenies has been gained via archaeologic and demographic techniques, the evolution of the Phasianidae family is still under debate. Now that sequencing data are available, several conclusions that were drawn about Phasianidae evolution based on fossil evidence are inconsistent

with results from mtDNA analysis, mainly those concerning phylogenies and divergence time. We believe this is likely due to the rapid diversification of the Phasianidae family [5, 6, 29]. Because of this rapid diversification (observed during the Eocene), as well as the short divergence times within some lineages, the phylogenies of galliform birds (including chicken, turkey and quail) usually have low bootstrap support values [30]. Phylogenies based on the *CR1* retrotransposon support the hypothesis that quail and turkey are more closely related than quail and chicken, while those based on mitochondrial genome data support the hypothesis that quail is more closely related to chicken than turkey [30]. However, phylogenies of the Perdicinae (Japanese quail), Meleagridinae (turkey) and Phasianinae (chicken) subfamilies are still not clear based only on information that is inferred from either current fossil evidence or partial genome data. Therefore, comparing these species at a whole-genome level will enable us to better understand the process of speciation of Phasianidae family. A high-quality genome assembly of the quail with population genomic data of the quail is necessary to address these questions [30, 31].

Here, we report the completion of an additional genome assembly of Japanese quail (*Coturnix japonica*), as well as the resequencing of 71 quail, both domestic and wild, and we describe experimental results concerning several important quail traits. These results were then used to characterize the mechanisms of early sexual maturity in quail; resolve the phylogeny and divergence time of the Phasianidae family; and detect footprints of artificial selection in the quail genome. We have also identified the genetic basis for a plumage color marker that is widely used in quail breeding. These

results will facilitate biological discovery, the improvement of quail for meat and egg production, and help resolve the basis of evolution within the Phasianidae family.

Results

Characteristics of the quail genome

High-quality genomic DNA extracted from a female quail (Shendan quail 1) was used to generate 262Gb of sequence (approximately 238-fold coverage of the whole genome) (**Table S1**) using the Illumina HiSeq 2000 platform. The genome assembled using SOAP*denovo2* [32] spans 1.04Gb (93.9% of the estimated genome size for quail, **Figure S1**) with contig N50 and scaffold N50 lengths of 27.9 kb and 1.8 Mb, respectively (**Table S2**). About 901 Mb of sequence (86.6% of the whole genome) was anchored to 30 chromosomes using a previously reported genetic linkage map [33] (**Figure 1a)**. We aligned these chromosomes back to a previously reported quail genome assembly (NCBI BioSample: SAMN03989050) and found that the two genomes had a high degree (92.14%) of consistency (**Figure S2**). The length and GC distribution of chromosomes are also highly consistent between quail and chicken genome sequences (**Figure 1b** and **Figure S3**). To evaluate the quality of the assembled quail genome, seven fosmid clones, each about 40 kb in length, were sequenced and mapped back to the quail genome assembly with a high coverage ratio (>92% for all, and six of seven fosmids >98.4%) (**Table S3** and **Figure S4**). To assess the integrity of protein-coding genes in the quail genome assembly, all transcripts assembled from RNA-Seq data sampled from the hypothalamus and ovary of three

stages of quail maturity (before-laying (BL), laying (L) and peak-laying (LP)), were mapped to the assembled genome (**Table S4**) and ~96.33% of total complete BUSCO genes can be identified in the genome (**Table S5**). These measures demonstrated the high quality of our genome assembly, allowing it to serve as a reference genome for subsequent quail genome research.

Genome annotation of our quail genome assembly included transposable elements (TEs) and protein-coding genes. TEs comprise 12.4% of the genome, which is a little higher than the average value in the class Aves [34], and 9.4% of the genome consists of long interspersed nuclear elements (LINEs) (**Figure 1a** and **Table S6**). Gene prediction was performed using a combination of several methods, including homology searches, *ab initio* prediction and RNA-Seq data. The merged results revealed evidence for 16,210 protein-coding genes in the quail genome (**Table S7**), and 15,972 (~98.5%) genes were supported by known protein-coding entries in at least one of the following databases: Swiss-Prot, InterPro, GO, TrEMB or KEGG (**Table S8**).

Evolutionary relationships within the Phasianidae family

To resolve the phylogenetic debate in the Phasianidae family and establish the phylogenetic position of the quail in relation to other avian species, we defined 12,178 gene families in quail and 10 other representative bird species, with *Alligator sinensis* (Chinese alligator) serving as an outgroup (**Figure S5**). 9,631 gene families were shared among four species (*Taeniopygia guttata*, *Pseudopodoces humilis*, *Gallus*

gallus and *Coturnix japonica*, **Figure S6**) and 4,393 single-copy orthologs were shared among 12 species. These single-copy orthologous genes were used to construct a phylogenetic tree (**Figure 2a** and **Figure S7**) and estimate the divergence times of the quail from other birds. Quail was mapped to the evolutionary branch containing domesticated poultry and was most closely related to the chicken lineage, sharing a common ancestor about 22.2 MYA (**Figure 2a**). We used our genome-wide comparative data to estimate the divergence time of Galliformes and Anseriformes at 69.1 (64.5–75.4) MYA. Our results, therefore, fully support a closer relationship between quail and chicken than between quail and turkey. The phylogeny we generated implied that the quail and chicken genomes likely share significant similarities which makes further comparison of their genomes intriguing.

In total, 95.5% of quail genome sequences occurred in blocks colinear with those in chicken (**Figure 2b** and **Table S9**). However, a total of 131 large inversions (block length >5 kb) between quail and chicken chromosomes were also identified and most of these were located on chromosomes 1 (24 breakpoints) and Z (24 breakpoints) (**Table S10**). Next, to investigate the nature of chromosome breaks that differentiate the quail and chicken genomes, and to associate these differences with possible phenotypic changes during their divergence, we tested for gene set enrichments at the boundaries. We identified 433 genes located within the 1 kb regions flanking the breakpoints of these inversions (**Table S10**). We tested for gene function enrichment within these inversions and searched for candidate mutations that might contribute to specific phenotypes in quail compared with chicken. The results of GO term

enrichment analysis of these genes revealed the terms GO:0005882: intermediate filament (*P*=1.53e-05) and GO:0005200: structural constituent of cytoskeleton (*P*=0.00029) were significantly enriched (**Figure S8** and **Figure S9**). In particular, a gene encoding tyrosinase-related protein 1 (*TYRP1*) was identified in the flanking region of an inversion on chromosome Z, which has been reported as a candidate locus for the recessive, sex-linked roux (br(r)) phenotype in Japanese quail [35] (**Figure 2c**).

Nucleotide diversity and population structure

To obtain a comprehensive understanding of genetic diversity in a quail population, we collected a total of 31 samples for genome re-sequencing, which includes 10 quails from wild population, 11 egg-type quails and 10 meat-type quails from domesticated subpopulations (**Table S11**). We sequenced these samples with an average read depth of 19X and mapped the reads to our reference genome (Shendan quail 1) with average coverage of 96.72% (**Table S12**). Eventually, we identified a total of 43,108,723 biallelic SNPs (single nucleotide polymorphisms) among the 31 re-sequenced samples, which included 21,597,713, 18,966,964 and 35,182,459 SNPs in wild quails, egg-type quails and meat-type quails, respectively (**Table 1**). Of the ~43M high-quality SNPs, only 649,024 SNPs were located in exonic regions, yet there were 17,227,986 SNPs in intron regions. Thus, the ratio of the number of SNPs in exons and introns was 3.77e-3, roughly equivalent to that of turkey (4.30e-2) [36] and chicken (3.50e-2) [37]. Accordingly, we found that the non-synonymous SNPs (N)

and the synonymous SNPs (S) in quail were 202,742 and 446,282, respectively, with a ratio of N/S of 0.454, which don't show a significant difference to chicken (0.41) or turkey (0.45). To evaluate the genetic diversity of our quail population, we calculated two common summary statistics across the whole genome, π and θ_w [38, 39], by using 100 kb sliding overlapped window with step length of 10 kb (**Table 1** and **Table S13**). Compared to the two domesticated subpopulations, the wild quail population displayed both higher π and higher θ_w on autosomes, indicating greater genetic diversity in the wild population. The same pattern was found on chromosome Z. The genetic diversity on chromosome Z was reduced compared to the autosomes in all three populations, a phenomenon which has been observed in a variety of other ZW system studies [40, 41]. The fact that sex chromosomes and autosomes differ in their effective population size, mutation characteristics and demography contributes to the differential genetic diversity within the genome [42].

To investigate the phylogenetic relationships and population structure among the 31 quail samples (**Table S11**), we constructed a neighbor-joining tree by using pairwise genetic distance matrix (**Figure 3a**) and performed principal component analysis (PCA) based on the variance-standardized genotype relationship matrix (**Figure 3b**).The neighbor-joining tree showed that our samples could be divided into two major clusters, corresponding to wild quails and domesticated quails, with a further subdivision of domesticated quails into egg-type quails and meat-type quails. This pattern was further confirmed by the PCA results. Specifically, the first principal component (PC1) in PCA successfully separated the wild from the domesticated

populations, and the second principal component (PC2) separated the egg-type and meat-type quails (**Figure 3b**). In order to better estimate the ancestral component in our quail populations, we adopted likelihood models embedded in structure by using ADMIXTURE [43]. The initialization of population number (*K*) was tried from 2 to 5, and the minimum estimated cross-validation error occurred with *K*=2 (**Figure 3d** and **Figure S10**). These results suggest that there was a distinct background between the wild population and domesticated population, similar to the results observed in NJ tree and PCA. The likelihood model based on *K*=2 grouped the three quail populations into two genetic clusters (**Figure 3d**), one which includes the wild quails and another that includes the domesticated quails. Considering the physiological and ethological characteristics of the quails investigated, we would have preferred to divide the 31 samples into three populations (egg-type, meat-type and wild quail). *K*=3 actually provides strong support for this scenario. This model groups the egg, meat, and wild quails into three distinct genetic clusters (**Figure 3d**), and the slight mixing shown between the egg- and meat-types could also explain the spread along the PC2 axis in the PCA plot. To characterize linkage disequilibrium (LD) blocks in wild and domesticated quails, we estimated the squared correlations (r^2) of pairwise SNPs with sliding window lengths from 1 to 50 kb. LD decayed to half of its maximum within a window length of ~20 bp for wild quail, ~100 bp for egg-type quail and ~230 bp for meat-type quail, respectively (**Figure 3c**). Such a rapid decay of LD in each population might be due to the high density of SNPs in the quail genome (one SNP in every ~20 bases, on average), and a high degree of recombination within

the quail genome. Similarly, other studies involving the population structure of Aves animals also revealed the low level of LD corresponding to the open genome and fluid genomic background in bird population which could facilitate adaptive variation [44, 45].

Signals of selection across the quail genome

Due to the low level of population divergence in our wild quails, any evidence of selective sweeps in short genomic regions could be masked in domesticated populations. Thus, we sought to detect the large-scale regions in the whole genome that may have been subjected to successive selective sweeps between the domesticated and wild populations by using 100 kb overlapping sliding window in 10 kb step. The reduction of diversity (ROD), defined as ROD = 1- $\pi_{domesticated}/\pi_{wild}$, was introduced to measure the loss of diversity in the domesticated population compared to the wild population. Additionally, in order to avoid ROD being excessively affected by diversity in the wild population, we also added a significantly negative Tajima's *D* (*D* < -2) in the domesticated population as a parallel criterion, which could represent a recent selective sweep or population expansion following a strong bottleneck [46-48]. From the comparison between egg-type quails and wild quails, we identified a total of 18 large-scale regions of selection sweep with the spans all greater than 100 kb, where the sliding windows presented high ROD values and a small Tajima's *D* in the 1% tail of the distribution (**Figure S11** and **Table S14**). The fixation index (F_{ST}), a measure of population divergence due to genetic structure, was substantially used as an additional condition to infer selection sweep at a high level (F_{ST} > 0.3). We identified 18

selective sweep regions with total length of 7.9 Mb between egg-type quail and wild quail. Interestingly, we observed the longest 1.8 Mb sweep region was located on chromosome Z and the length of sweep regions on chromosome Z was up to 5.57 Mb (**Figure 3e**). Furthermore, we noticed a gene (*CCDC171*) which is significantly associated with quail plumage color, was just located on the longest region and included in a 182 kb haplotype block (*see the association study described below*). This may indicate that positive selection for plumage color might have resulted in a strong selective sweep on chromosome Z in egg-type quail. A total of 88 genes involving these selective sweeps have been annotated Gene Ontology (GO) terms. By using the Kyoto Encyclopedia of Genes and Genomes (KEGG) database, we noticed that some of these genes possibly played a role in sex hormones [49], embryo development [50, 51], increase of egg weight [52] and plumage color [53] (**Table S15**). Thus, we surmise that these important traits in egg-type quail might have suffered stronger artificial selection, leading to many large-scale selection sweeps on chromosome Z. Similarly, we also identified 26 large regions of selective sweep between meat-type quail and wild quail on chromosomes 1, 2, 13 and Z (**Figure S11** and **Table S16**). The total length of selective sweeps in meat-type quail was estimated at ~8.77 Mb, and the longest span could be as long as 1.2 Mb on chromosome 2. That led us to infer that breed improvement for meat-type quail was likely not restricted to chromosome Z, but could affect many other genomic regions. Subsequently, we annotated the biological functions of 118 genes in those meat-type quail sweep regions (**Table S17**). Although most of genes that correspond to relevant traits had not

been previously verified in meat-type quail, a more detailed investigation of these candidate genes could further our understanding of the domestication process in future studies. However, it is worth mentioning that there was hardly any selective sweep shared between wild quail and egg-type quail, or between wild quail and meat-type quail except for a 130 kb region (40.15-40.28 Mb) on chromosome 4 which contains one annotated gene: COT02188 (vascular endothelial growth factor C/D) (**Table S15** and **Table S17**). With such differentiated large selective sweeps, we speculate that egg-type quail and meat-type quail might have undergone selection independently subsequent to their initial domestication in the early 20th century [17].

Despite that selective sweep gave us a new insight into an evidence of domestication process, we also found that a number of functional genes dispersed across the whole genome were contributed to the divergent traits observed when comparing domesticated quails and wild quails. Thus, we picked up the highly differentiated non-synonymous SNPs with *F*st values over 0.5, that show high population differentiation in the between wild and egg-type population. Specifically, we found 1,943 highly differentiated non-synonymous SNPs in 1,213 genes between egg-type and wild quail; and 3,508 highly differentiated non-synonymous SNPs in 2,032 genes between meat-type and wild quail. We then performed functional enrichment analysis of KEGG pathways using these sets of genes for both egg-type quail and meat-type quail and found that 8 of the top 20 most enriched pathways were shared by egg-type and meat-type quail (**Figure S12** and **Figure S13**). Of these, the most significantly enriched pathway was ECM-receptor interaction (ko04512), an

activity which plays important roles in both the integrity of tissue and intramuscular fat metabolism [54]. Other functional pathways, specific to either egg-type or meat-type quail, were not significantly enriched, indicating that artificial selection targeting these traits was not strong. In summary, the independent selection for similar traits in the two domesticated subpopulations suggests that the phenotypic difference between egg-type and meat-type quail is not that significant, which is consistent with the observation during the population stratification analysis that the optimal number of populations was two rather than three.

Genes involved in early sexual maturity

To explore the biological mechanism of very early sexual maturity in quail, genes were traced from both gene family evolution and positive selection events in the quail lineage. We found that several gene families have expanded in the quail genome compared with those of other domesticated birds. These families include those encoding gonadotropin-releasing hormone 1 (*GnRH1*, **Figure S14**), the lysophospholipase catalytic domain and phospholipase A2 (**Table S18**). Moreover, four positively selected genes (PSGs) were detected in the quail lineage and the proteins encoded by these genes (*FSHβ*, *PLCB4*, *ITPR1* and *PLA2G4*) are involved in the *GnRH* (gonadotropin-releasing hormone) signaling pathway. Follicle stimulating hormone *beta* (*FSHβ*) protein, is a glycoprotein polypeptide hormone that, in conjunction with luteinizing hormone, contributes to growth and reproduction [55]. Transcription of the *FSHβ* gene is the rate-limiting step in hormone synthesis [56]

which is required for ovarian folliculogenesis in females and for spermatogenesis in males, in conjunction with testosterone [57]. We identified two amino acids in the quail *FSHβ* protein at position 37 (M→F/L) and position 99 (G→E/A) that were predicted to be under positive selection (**Figure 4a**). We used ELISA to measure the level of *FSHβ* protein during early developmental stages and found that the level of *FSHβ* in early-maturing quail blood is consistently higher than that in chicken ($P<0.05$) (**Figure 4c**). We used SWISS-MODEL to model the structure of quail *FSHβ* using the Follitropin subunit beta (4ay9.1.B) protein[58] as a template. These two amino acid substitutions were mapped to the 3D protein structure and were located near the *β*-pleated sheet that interacts with the *FSH* receptor (**Figure 4b**). *PLCB4*, *ITPR1* and *PLA2G4* (**Figure S15-17**), together with other molecules (e.g., inositol 1,4,5-trisphosphate, diacylglycerol and protein kinase C) stimulate release of gonadotropins including luteinizing hormone and follicle stimulating hormone [59, 60]. Gene expansions in the *GnRH* families, and also PSGs in the *GnRH* signaling pathway, are likely to be involved in the acceleration of growth and sexual maturity in the quail. Subsequently, we scanned both synonymous and non-synonymous SNPs found in the coding sequence (CDS) of these four genes in the 31 wild and domestic individuals, and found that all but four of the 83 SNPs were synonymous substitutions. However, all the divergent alleles of SNP loci did not generally segregate according to the three subpopulations, and the domestic and wild quails presented no large-scale selective sweeps around these genes.

Gene families related to immune system function

We identified a total of 1,587 immune response-related genes in quail (**Table S19**) by aligning the entire predicted gene set of quail against 2,257 genes that have been annotated with roles in the innate immune responses of *Homo sapiens*, *Mus musculus* or *Bos taurus* at InnateDB or Gene Ontology databases. Compared to chicken, turkey and duck, several expanded gene families were identified in quail. These included *Klf4*, which is indispensable for differentiation of inflammatory monocytes [61] and negative regulation of innate immune response against several viruses in human embryonic kidney 293 cells [62] (22 copies in quail, 17 in chicken, 16 in turkey and 12 in duck); *Foxa2*, which regulates genetic programs that influence pulmonary inflammation mediated by Th2 cells [63] (13 copies in quail, 7 in chicken, 4 in turkey and 5 in duck); and *ITCH*, which acts in T-helper cell differentiation and T-cell activation and tolerance [64] (7 copies in quail, 3 in chicken, 4 in turkey and 2 in duck). Moreover, we focused on the number of 'Immunoglobulin subtype' genes and found that there are 109 in quail, while chicken, duck, zebra finch (*Taeniopygia* g*uttata*) and turkey each had 62 or fewer immunoglobulin-related genes (62 copies in chicken, 29 in duck, 29 in zebra finch and 34 in turkey; **Figure 4d**). We detected 69 genes encoding a putative 'Reverse transcriptase or Reverse transcriptase domain' in quail but found only two and four such genes in chicken and turkey, respectively (**Figure S18**). These domains are signatures of retroviruses integrated into the host genome. Next we compared the *MHC-B* region between quail and chicken (**Figure S19**) and found that there was an inversion including the genes encoding the proteins *TAP1* and *TAP2*, which transport peptides from the cytosol into the endoplasmic

reticulum to bind *MHC* class I molecules that are being synthesized [65], and *BFIV21*, which encodes a protein that presents antigens such as the avian leukosis virus [66]. We also found four copies of the *BLEC2* (C-type lectin-like NK cell receptor) gene in quail, but only one in chicken. However, several other *MHC* genes (e.g., *KIFC1*, *V-BG1* and *BG2*) were not detected in the quail genome. A better understanding of these immune-related genetic changes will help us characterize the immune response in quail and facilitate the development of targeted vaccines for quail.

Genome wide association analysis of plumage color

In order to identify sexed-linked genes conferring plumage color, we bred a set of egg-type quails with maroon or yellow plumage, a trait that has been confirmed to have sex-linked inheritance in quail, and is consistent with a Mendelian segregation ratio according to our previous investigation (*see more details in Method*). From this set we sampled 40 quails, including 20 male and 20 female quails, and re-sequenced their genomes for case-control analysis (**Table S20**). We identified ~20M bi-allelic SNPs in these 40 quails at a sequencing depth of 20–30×. After controlling for SNP quality and redundant LD (*see more details in Methods*), a final total of 864,292 SNPs was retained for subsequent analyses. A genome similarity test of the 40 quail samples was conducted using high-quality SNPs, and we found that similarities between any pair of individuals ranged from 70.4 to 86.5%, indicating relatively high homology between them.

Due to the relatively close relationships between the 40 samples, the effect of

relativeness matrix affecting the variance of plumage color was considered as the covariance. We assessed the relationships matrix of the 40 samples by using GEMMA v0.94 [67] and adopted the linear mixed model for association analysis. By Bonferroni correction, the association analysis showed that two SNPs, 61102026 on chromosome 1 and 23173971 on chromosome Z, had significant effects on plumage color (adjusted P=0.028 and P=0.019, respectively) (**Figure 5a**, **Figure S20** and **Table S21**). However, unlike the locus on chromosome 1, SNPs on chromosome Z near 23173971 showed a continuous peak on the Manhattan plot. In our previous analysis of plumage color heredity, we suggested that the locus on chromosome Z was most likely associated with plumage color. In a confirmation study, we added the 21 previously re-sequenced quails with "maroon" plumage (including 10 wild quails and 11 egg-type quails, **Table S11**) to rerun the association analysis for the two loci. The locus on chromosome Z was found to be more significantly associated with plumage color than before (adjusted P=0.015). Conversely, there was no significant signal on chromosome 1 (adjusted P-value for SNP 61102026 fell to 1.000). Subsequently, the SNP 23173971 on chromosome Z, we found, was located closely to gene Coiled-coil domain-containing 171 (*CCDC171*) with a length of 135 kb. Therefore, we chose SNP 23173971 on chromosome Z as the index SNP within the region of 200 kb for conditional haplotype-based association testing. Consequently, 47 SNPs with r^2>0.7 and adjusted P>0.01 were clumped together for association testing. Using 5,000 permutations, a highly linked haplotype with a range of 182 kb could significantly explain the maroon/yellow variation (χ^2=37.7, P=8.563e-06). The well-known *TYRP1*

gene that confers variable plumage color [53, 68] was located approximately 531 kb away from *CCDC171* (**Figure 2c**). The average of LD value between them was estimated at less than 0.2. These observations suggested that the gene controlling plumage color in our population was different from *TYRP1*. We then chose eight SNPs significantly associated with plumage color, five of which are located within *CCDC171*, and designed PCR primers to amplify these SNP markers to genotype an additional 100 “maroon” and 100 “yellow” quails. Interestingly, 99.75% of these SNPs were consistent between genotype and phenotype, suggesting that the *CCDC171* gene does control plumage color in quail (**Figure 5b**). We cloned the *CCDC171* gene from yellow and maroon quail and found that this gene encoded different transcripts in quail depending on plumage color (**Figure 5c** and **Figure S21**). To examine the nature of the *CCDC171* genetic variants, we characterized the transcripts from the maroon and yellow alleles. The transcript from the yellow allele was longer than the maroon transcript (about 232 bp) at the upstream region of the translation initiation site of maroon and has a deletion (147 bp) at position 787. In addition, we examined the differential expression of *CCDC171* in yellow and maroon quails and found there were no significant difference between the collected samples (*t*-test, P>0.05).

Discussion

Birds represent the most widespread class of domesticated animals in the world and are the subjects of many evolutionary, biological and pathology studies that

illustrate the relationships among these avian species [30]. The timing of sexual maturity is critical for both plants and animals. Quail have a unique maturation program compared with other birds and reach sexual maturity in a very short time. We detected four promising genes for this trait under positive selection in the *GnRH* signaling pathway in quail. Gonadotropins act on the testis and ovary to promote their development and the production of steroid hormones [69]. Further functional analysis of these genes should provide new insights into the genetic mechanisms that regulate avian sexual maturity.

Analyzing the genes and mutations related to the development and evolution of agronomic traits in quail will also improve our understanding of the genetics of domestication. Genome-wide comparisons of domesticated (egg-type and meat-type lines) with wild quail identified several footprints of artificial selection. These selective sweep regions harbor candidate genes associated with important agro-economic traits. Genetic variations in these genes will be a rich resource for improving quail egg and meat production *via* genetic selection. It is worth noting that egg-type and meat-type quails did not share selective sweep regions when compared with wild quails (**Figure S11**), meaning that egg-type/meat-type quails might have been independently selected after domestication or that there were two separate domestication events in quails. Further studies are required to fully describe the domestication history of quails. Based on re-sequencing data, we have also identified a haplotype that is completely correlated with the control of “maroon/yellow” plumage color, a trait that has been used extensively in the breeding of domestic quail

as a sex-linked marker.

Some recent studies [70] which use genomic data support our current understanding of the phylogeny of the Perdicinae, Meleagridinae and Phasianinae families. However, without genome-wide data we have not been able to make strong conclusions. Here, we used whole-genome sequences of Japanese quail, turkey and chicken to represent each clade and resolve the phylogenetic relationships among the Perdicinae, Meleagridinae and Phasianinae families. Our study provided fully resolved branches with genome-scale data, supporting a split of the Perdicinae and Phasianinae branches from the Meleagridinae branch about 69 MYA. Calibration based on fossils of early penguins, together with mitochondrial genome sequences of a modern albatross (*Diomedea melanophris*), petrel (*Pterodroma brevirostris*) and loon (*Gavia stellata*), allowed the divergence time of the Anseriformes and Galliformes to be estimated as 77.1± 2.5 MYA [5]. Other recent avian genome data was used to estimate the divergence of Anseriformes and Galliformes at about 66 MYA[71]. The resolution of their phylogeny will improve our understanding of the genetics of speciation of quail, chicken and turkey. In this research, we obtained a high quality draft of the Japanese quail genome and whole-genome resequencing data of multiple quail sub-populations which will provide new opportunities to further understand quail biology and develop molecular markers for improving economically important agronomic traits.

Conclusions

In conclusion, we accomplished the genome assembly of quail with high-depth sequencing and carried out re-sequencing for 71 domestic and wild quail. We solved the long-term arguments of phylogeny of quail, turkey and chicken and interpreted the biological mechanism of very early sexual maturity for quail. From the GWAS analysis, we detected a haplotype marker on chromosome Z which is important for quail breeding. These analyses should provide a valuable resource for the future studies for quail.

Materials and Methods

Animal samples collection

All 31 wild and domestic quails were collected from China. Of these, the 10 wild quails were sampled from the common habitats of wild quails in Henan province and Shandong province, respectively. The other domestic quails were provided by local breeding companies (**Table S11**). The maroon or yellow plumage population were derived from two pure lines offered from Hubei Shendan Healthy Food Co., Ltd. In our previous investigation, we found that the F_2 population from a crossing of the maroon plumage line and the yellow plumage line showed a 3:1 segregation ratio in plumage color. Thus, we randomly chose 20 yellows and 20 maroons from the pure lines for the association study (**Table S20**). All 71 quail samples were used for re-sequencing by BGI-Shenzhen. Additionally, the 100 maroon ones and the 100 yellow ones were also derived from the two pure lines for validation of the plumage color gene.

Genome sequencing and assembly

A female quail collected from the maroon population was used for all genome sequencing. All experiments in this project were performed according to the principles of the animal ethics committee at BGI (China). DNA samples were isolated from blood following standard molecular biology techniques. A series of libraries of different insert sizes ranging from 170 bp to 40 kb (170 bp, 500 bp, 800 bp, 2 kb, 5 kb, 10 kb, 20 kb and 40 kb) were constructed and used for a shotgun sequencing strategy. The Illumina HiSeq 2000 system was used to generate paired-end reads. A total of

262Gb of raw data was obtained and reads were filtered based on the following criteria. Reads with less than 10 percent unidentified (N) bases, with less than >40% low-quality bases, were contaminated by adaptors, or were duplicated during PCR were discarded; about 199Gb of clean data remained. The genome size (G) of quail was first estimated at about 1.1Gb using the 17-mer depth frequency distribution method: G = K-mer_num/Peak_depth (**Figure S1**). The genome was assembled using SOAPdenovo2 v2.04.4 (SOAPdenovo2, RRID:SCR_014986) [32]. Next, paired-end reads were mapped back to the initial assembled genome to link contigs into long scaffolds. Benchmarking Universal Single-Copy Orthologs v2 (BUSCO, RRID:SCR_015008) [72] was used to assess the assembly of genome with lineage dataset vertebrata_odb9.

Genome annotation

RepeatMasker v4.0.5 (RepeatMasker, RRID:SCR_012954) [73] and Repeat-ProteinMask v4.0.5 were used to search for TEs (transposable elements) against the RepBase library v20.04 [74] to detect known repeats. A custom TE library was then constructed using RepeatModeller v1.0.8 and LTR_FINDER v1.0.6[75] for *de novo* detection of repeats. Tandem Repeat Finder v4.0.7 [76] was also used to predict tandem repeats. Final results of TE detection were integrated using in-house scripts.

Homology-based and *ab initio* gene prediction methods, assisted by transcriptome sequencing, were used to analyze coding DNA sequences and to model genes. Initially, protein data for *Homo sapiens* (human), *Meleagris gallopavo* (turkey),

Gallus gallus (chicken), *Taeniopygia guttata* (zebra finch) and *Anas platyrhynchos* (duck) were downloaded from the Ensembl v80 database [77] and aligned to the predicted proteins encoded by the quail genome using BLAT (BLAT, RRID:SCR_011919) [78]. GeneWise v2.2.0 (GeneWise, RRID:SCR_015054) [79] was then used to further improve the accuracy of alignments and predict gene structures. AUGUSTUS v3.1 (Augustus: Gene Prediction, RRID:SCR_008417) [80] and GENSCAN v1.0 (GENSCAN, RRID:SCR_012902) [81] were then used for *ab initio* gene prediction. Transcriptome reads were mapped to the genome with TopHat v1.2 (TopHat, RRID:SCR_013035) [82] and Cufflinks v2.2.1 (Cufflinks, RRID:SCR_014597) [83] was used to confirm gene structures. Subsequently, we combined the homology-based and *de novo* predicted gene sets using GLEAN [84] and integrated the GLEAN and transcriptome results with in-house scripts to generate a representative and non-redundant gene set.

Gene evolutionary analysis

Gene families in quail, *Gallus gallus* (chicken, Ensembl v80), *Anas platyrhynchos* (duck, Ensembl v80), *Columba livia* (pigeon, [85]), *Falco cherrug* (Saker falcon, [86]), *Falco peregrinus* (Peregrine falcon, [87]), *Ficedula albicollis* (collared flycatcher，Ensembl v80), *Geospiza fortis* (medium ground finch，[88]), *Meleagris gallopavo* (turkey，Ensembl v80), *Pseudopodoces humilis* (ground tit，[89]), *Taeniopygia guttata* (zebra finch，Ensembl v80) and *Alligator sinensis* (Chinese alligator，ASM45574v1), as an outgroup, were defined using TreeFam (http://www.treefam.org/). Phylogenetic trees were then constructed using MrBayes

(MrBayes, RRID:SCR_012067) [90] and PhyML (PhyML, RRID:SCR_014629) [91] with fourfold degenerate (4D) sites of 4,393 single-copy orthologs shared among the 12 species analyzed here. Divergence times were estimated using MCMCTree (http://abacus.gene.ucl.ac.uk/software/paml.html) from the PAML (PAML, RRID:SCR_014932) package [92] together with three fossil dates from the TimeTree database [93] (http://www.timetree.org/) for calibration. Analyses of the expansion and contraction of gene families were carried out using CAFE (Computational Analysis of Gene Family Evolution) [94] using a random birth and death model with a global parameter, λ, which represents the probability of both gain and loss of a gene over a given time interval. Conditional *p*-values were calculated and defined as significant at values of less than 0.05. To detect PSGs, the coding sequences of all the single-copy orthologous genes were aligned using PRANK [95] and poorly aligned sites were removed using gBlocks [96]. High-quality alignments were then filtered to estimate the ratios ($\omega = d_N/d_S$) of nonsynonymous nucleotide substitutions (d_N) to synonymous nucleotide substitutions (d_S) for these genes in the target quail branch (ω_0), other branches (ω_1) or all branches (ω_2) using the codeml program with an improved branch-site model (TEST-II) [97] (model = 2, NSsites = 2) and the maximum likelihood method in the PAML package [92]. TEST-II is a likelihood ratio test that compares a null hypothesis with fixed $\omega=1$ with model A that allows $\omega_2>1$ in the foreground lineages. TEST-II can discriminate relaxed selective constraints analysis from positive selection and is a direct test for positive selection on the foreground lineages [98]. Positively selected sites were detected by using Bayes

Empirical Bayes (BEB) method [99] which can avoid an excessive false positive rate [100].

Resequencing and SNP calling

A total of 71 individuals were chosen for resequencing (see more information regarding samples). Genomic DNAs were isolated and then used to construct Illumina libraries with an insert size of 500bp. The Illumina HiSeq 2000 platform was used to generate paired-end reads and raw data was filtered by removing reads containing greater than 50% low-quality bases (Q value ≤5), reads containing more than 5% unidentified (N) bases and those with adapter contamination. The clean reads were mapped to the assembled reference genome using BWA software v0.7.12 (BWA, RRID:SCR_010910) [101] with parameters "-m 200000 -o 1 -e 30 -i 15 -L -I -t 4 -n 0.04 -R 20", and the results were transformed into indexed BAM files using SAMtools v0.1.18 [102]. The picard package v1.105 and Genome Analysis Toolkit v 3.3-0 (GATK , RRID:SCR_001876),) [103] were then used for SNP calling. To obtain high-quality SNPs, we: 1) deleted duplicate reads; 2) improved alignments using the IndelRealigner package in GATK; 3) recalibrated base quality scores using the BaseRecalibrator package in GATK; 4) called SNPs using the UnifiedGenotyper package in GATK with a minimum phred-scaled confidence value of 50 and a minimum phred-scaled confidence threshold of 10 for calling variants; 5) assessed variant quality using the VariantRecalibrator and ApplyRecalibration packages with truth sensitivity filter level of 99 in GATK; and 6) filtered SNPs using the VariantFiltration package in GATK with parameters "--filterExpression "QD < 2.0 ||

MQ < 40.0 || ReadPosRankSum< -8.0 || FS > 60.0 || HaplotypeScore> 13.0 || MQRankSum< -12.5" –filterNameLowQualFilter –missingValuesInExpressionsShouldEvaluateAsFailing”.

SNP quality control (QC)

The chromosomal variant call format (VCF) files were transformed into PLINK format by using VCFtools v0.1.13 (https://vcftools.github.io/index.html) and subsequent analyses were performed by using PLINK v1.07 (PLINK, RRID:SCR_001757) [104]. As the default chromosome handling type in PLINK is for human (1:22, X, Y), the PLINK files for male quail (ZZ) and the female quail (WZ) were swapped with each other before data were analyzed because the heterogametic gender in quail is female. Additionally, the command --dog (39n) was added at the beginning of each command line to ensure that all quail chromosomes would be included.

Individual quality control consisted of the following three steps: 1) determining the sex of individuals, 2) detecting individuals with missing genotypes and 3) identifying duplicate or highly related individuals. Any discordant sex information was checked in terms of the heterozygosity rates on the Z chromosome as described by the *F* statistic, that is, any individual quail for which the *F*-value was less than 0.8 in a male quail (ZZ) or greater than 0.2 in a female quail (ZW) would be removed from the sample set. The missing genotype rate for each individual was set to less than 10% to filter out individuals with unreliable genotype information. Case-control association studies assume that all individuals in a population are unrelated. We used

a complete linkage agglomerative clustering method that was based on pairwise identity-by-state (IBS) to identify the genomic similarity of pairs of individuals. Any individual with an IBS greater than 0.9 would be filtered out of the sample set.

SNP quality control consisted of the following four steps: 1) estimating the missing genotype rate (MGR) for each SNP, 2) determining whether there was a significant difference in the rate of missing SNP genotypes between the case and control groups, 3) filtering out SNPs with very low minor allele frequencies (MAF), and 4) filtering out SNPs with frequencies that deviate significantly from Hardy-Weinberg Equilibrium (HWE). Filtering out the low-quality SNPs helped not only to avoid false-positives, but also to enhance our ability to identify the loci significantly associated with traits. Therefore, the criteria for filtering were MGR>0.05, a significant difference in MGR between case and control according to *t*-test, at $P<0.05$, MAF<0.05 and a *P*-value for deviation from HWE<0.0001.

Extensive genome-wide regions of high linkage disequilibrium (LD) in quail strongly influenced the results of population structure, principal component and association analyses. Thus, we pruned out the pairwise SNPs with r^2 values of greater than 0.2 in each 50 bp sliding window, and set 10 bp steps for sliding window analysis to ensure 80% overlaps between any two adjacent windows.

Population structure analysis

The phylogenic tree was constructed using the neighbor-joining method in MEGA v6.0 (MEGA Software, RRID:SCR_000667) [105] based on a pairwise distance matrix which was estimated using IBS distances in PLINK v1.07. Analysis of

population stratification was conducted by performing complete linkage clustering of individuals using autosomal genome-wide SNP data in PLINK. Principle component analysis (PCA) was carried out using the smartpca script (https://github.com/argriffing/eigensoft/blob/master/bin/smartpca.perl) and the scatter plots were drawn by using R v3.2.2 (https://www.r-project.org/). We used ADMIXTURE v1.3 [43] to analyze population structure, which uses the likelihood model-based manner from large autosomal SNP genotype datasets. The number of populations (K) was set from $K = 2$ to 5 to obtain the maximum likelihood estimates that would allow us to infer population structure. The cross-validation procedure was performed to exhibit a low cross-validation error, which made it fairly clear that what the optimal K value was. The parameter standard errors were estimated by using 100 bootstrap replicates. The cross-validation plot was drawn by using R v3.3.2. The average LD of a pair of SNPs in a 300 kb sliding window was estimated by using PopLDdecay v2.69 (https://github.com/BGI-shenzhen/PopLDdecay), and the LD decay curves for the three populations were drawn by using R v3.3.2.

Calculation of nucleotide diversity and estimation of population differentiation using F_{ST}

Watterson's estimator θ_w [38] and the average number of pairwise differences per sequence estimator π [39] were calculated using in-house Perl scripts. Tajima's D [39] was estimated using θ_w, π and the number of sequences. We scanned the whole genome to calculate the three estimators by using the 90% overlapped sliding window with size of 100 kb or 50 kb. The fixation index (F_{ST}), a measure of population

differentiation due to genetic structure [106], was estimated by using VCFtools v0.1.13 also with 50 kb and 100 kb 90% overlapped sliding windows on a genome-wide scale.

Association analysis and conditional haplotype-based association testing

The post-QC data was saved as PED format and later was used for GWAS via GEMMA v0.94. The centered relatedness matrix was calculated with the parameter "–gk 1". The relatedness matrix was considered as a covariance using a linear mixed model to perform the Wald test, likelihood ratio test and score test. The GWAS results were shown as a Manhattan plot and Q-Q plot and were drawn by using the *qqman* package in R v3.2.2. The SNP with the most significant effect on phenotypic variation was regarded as the index SNP and the flanking 100 kb region of the index SNP was scanned for haplotype construction. In this region, the SNPs with high LD (r^2>0.7) and significant association to plumage color (adjusted P<0.01) were grouped into a clump. Then, the SNPs gathered in a clump were extracted by using PLINK v1.07 and transformed into Haploview format for conditional haplotype association testing. The haplotypes in block were estimated with permutation of 5,000, and the LD plot between gene *CDCC171* and *TYRP1* was drawn using Haplotype v4.2 [107].

Molecular experiments

(a) Follicle-Stimulation Hormone (FSH) testing by ELISA

We selected 100 male quails, 100 female quails, 100 hens and 100 cocks from Hubei in China and they were raised under the same conditions. In four populations,

blood samples from 10 individuals were collected every week (0-6 weeks). The sera were separated from the blood and stored in -20℃ for testing. The *FSH* hormone of quails and chicken were tested using the Follicle-Stimulation Hormone (FSH) ELISA Kit (Abcam, UK) according to the manufacturer's instructions.

(b) *CCDC171* transcripts cloning and expression

We designed three and four pairs of primers (**Table S22**) to clone transcripts of *CCDC171* in yellow and maroon quails respectively. For determining the differential expression of *CCDC171* in yellow and maroon quail, we collected hair follicles, back skin and abdomen skin from three "yellow" quails and three "maroon" quails. Two pairs of primers were designed to detect the differential expression of *CCDC171* by qPCR.

(c) Validation of SNPs

DNA from the different plumage quails was extracted from blood samples following standard molecular biology techniques and stored in -80℃, and used software Primer 6.0 for designing primers to validate the eight SNPs which were significantly associated with plumage color. The PCR products were sequenced on the Sanger sequencing platform.

Additional files

Additional file 1: Figures S1-21, Tables S1-12, Table S18, Tables S20-22.

Additional file 2: Table S13. Diversity statistics of 31 quails.

Additional file 3: Table S14. The large scale of genomic regions showing reduction

of diversity (ROD) between egg-type quail and wild quail at 100 kb overlapping sliding window in 10 kb step.

Additional file 4: Table S15. Functional enrichment for selective sweep regions between egg-type and wild quails.

Additional file 5: Table S16. The large scale of genomic regions showing reduction of diversity (ROD) between meat-type quail and wild quail at 100 kb overlapping sliding window in 10 kb step.

Additional file 6: Table S17. Functional enrichment for selective sweep regions between meat-type and wild quails.

Additional file 7: Table S19. Copy numbers of innate immune response-related genes among quail, chicken, turkey and duck.

Data availability

Resequencing and transcriptomic raw data are available from NCBI SRP104331. Data supporting the manuscript, including sequence assembly files, annotations and BUSCO results, are also available via the GigaScience database, GigaDB [108].

Acknowledgements

We would like to thank Dr. Leif Andersson, Dr. Sangeet Lamichhaney, Dr. Susanne Bornelöv and Prof. Maxwell Hinckegave for their insightful comments on this manuscript. We also thank Dr. Lynn Fink for revising the manuscript. This project was supported by the Agricultural Science and Technology Innovation Center of Hubei (2012tsxk09 and 2014jzxjh02) and the Key Laboratory of Animal Embryo and Molecular Breeding of Hubei (2014-2017 ZD146).

Authors' contributions

Y.W. was responsible for collecting samples for sequencing, carrying out experimental verification and co-drafting the manuscript. Y.Z. made contributions to genome annotation, phylogenetic analysis, immune-related gene analysis, detecting genes under positive selection, SNP calling in re-sequencing data and co-drafting of the manuscript. Z.H. and J.P. designed the scientific objectives and co-drafted manuscript. S.S. and J.C. carried out SNP quality control, GWAS analysis, detecting selective sweep regions and co-drafting of the manuscript. G.F. participated in genome assembly, analyzing inversions and co-drafting of the manuscript. W.C., X.C., J.J., X.F. and X.X. participated in project management and manuscript revision. H.L., J.S., A.P., Y.P., Z.L., H.Z., J.S., C.Z., H.T., H.X. and C.L. worked on sample preparation and experimental verification. X.D., G.H., P.Y., H.Z., T.Y., B.W., H.Y. and M.B. took part in data processing. D.B. and W.W. provided suggestions and revised the manuscript. N.Y., X.L. and J.D. designed this project, provided suggestions and revised the manuscript.

Competing interests

The authors declare that they have no competing interests.

Author details

[1]Institute of Animal Husbandry and Veterinary, Hubei Academy of Agricultural Science, Wuhan 430064, China

[2]BGI-Shenzhen, Shenzhen 518083, China

[3]Hubei Key Laboratory of Animal Embryo and Molecular Breeding, Wuhan 430064, China

[4]National Engineering Laboratory for Animal Breeding and MOA Key Laboratory of Animal Genetics and Breeding, China Agricultural University, Beijing 100193, China

[5]BGI-Qingdao, Qingdao, Shandong Province, 266555, China.

[6]Hubei Innovation Center of Agricultural Science and Technology, Wuhan, Hubei, 430064, China

[7]Hubei Shendan Healthy Food Co., Ltd., Wuhan 430206, China

[8]State Key Laboratory of Quality Research in Chinese Medicine and Institute of Chinese Medical Sciences, Macao, China

[9]The Roslin Institute and Royal (Dick) School of Veterinary Studies, University of Edinburgh, Midlothian EH25 9RG, UK

[10]Kunming Institute of Zoology, Chinese Academy of Sciences (CAS), Kunming, China

[11]China National GeneBank-Shenzhen, BGI-Shenzhen, Shenzhen 518083, China

[12]James D. Watson Institute of Genome Sciences, Hangzhou 310058, China

[*]These authors contributed equally to this work.

[†]Correspondence should be addressed to Jinping Du (ddjinpin@163.com) or Xin Liu (liuxin@genomics.cn)

Figure legends

Figure 1. Chromosomes of quail. (**a**) Gene and TE density of each quail chromosome. (**b**) Comparison of the chromosome lengths of quail and chicken.

Figure 2. Comparative evolutionary analysis of 12 avian species. (**a**) The phylogenetic tree of *Coturnix japonica* (quail), *Gallus gallus* (chicken), *Anas platyrhynchos* (duck), *Columba livia*(pigeon), *Falco cherrug* (Saker falcon), *Falco peregrinus* (Peregrine falcon), *Ficedula albicollis* (collared flycatcher), *Geospiza fortis* (medium ground finch), *Meleagris gallopavo* (turkey), *Pseudopodoces humilis*(ground tit), *Taeniopygia guttata* (zebra finch), with *Alligator sinensis* (Chinese alligator) as an outgroup. (**b**) Syntenic relationships between the quail and chicken genomes. (**c**) An inversion detected in chromosome Z between quail and chicken.

Figure 3. Analyses of the phylogenetic relationships, population structure, LD decay and genetic diversity between wild and domesticated quail. (**a**) Evolutionary history was inferred using the neighbor-joining method in MEGA 6.0. (**b**) Principle component analysis of wild quail and domesticated quail. (**c**) LD decay curves were estimated by squared pairwise correlations of alleles against physical distance in wild quail, egg-type quail and meat-type quail, respectively. (**d**) Population structure analysis with the maximum likelihood score for the model K = 2. (**e**) Nucleotide diversity between wild quail and egg-type quail across chromosome Z. Both the wild quail (red line) and the egg-type quail (green line) showed of the difference of diversity on chromosome Z. Plotting of Tajima's D for the egg-type group (blue line) in a 100 kb sliding window in 10 kb steps revealed the selective signal on chromosome Z. Likewise, plotting Weir's F_{st} (black line) on chromosome Z indicates the level of differentiation between the wild group and the egg-type group. Both of gene *CCDC171* and *TYRP1* were located within a selective sweep region (from ~21.5 Mb to 23.2 Mb), in which the positive signal was detected in the egg-type group. However, they exhibited a weak linkage due to the location on the different

haplotype blocks.

Figure 4. Genes related to early sexual maturity and immune system function in quail and another avian species. (**a**) Positions of amino acids under positive selection in the follicle stimulating hormone beta (FSHβ) protein. (**b**) Location of two amino acids under positive selection on the predicted 3D structure of the FSHβ protein. (**c**) Circulating FSHβ levels in blood during early development stages of quail and chicken for 6 weeks. (**d**) Phylogenetic tree of Immunoglobulin-like and Immunoglobulin subtype proteins of quail, chicken, duck, turkey and zebra finch.

Figure 5. GWAS analysis of quail plumage color. (**a**) Manhattan plot of each chromosome showing the GWAS results for quail plumage color. (**b**) Validation of eight candidate SNPs in 200 random individual quail. **(c)** The clones of *CCDC171* gene transcripts from quail with “yellow” and “maroon” plumage.

Tables

Table 1 Statistics of SNPs in whole genome and genic regions of wild and domesticated quail

References

1. Dalloul RA, Long JA, Zimin AV, Aslam L, Beal K, Blomberg LA, et al. Multi-platform next-generation sequencing of the domestic turkey (Meleagris gallopavo): genome assembly and analysis. PLoS Biol. 2010;8 9:e1000475.
2. Wallis JW, Aerts J, Groenen MA, Crooijmans RP, Layman D, Graves TA, et al. A physical map of the chicken genome. Nature. 2004;432 7018:761-4.
3. Kawahara-Miki R, Sano S, Nunome M, Shimmura T, Kuwayama T, Takahashi S, et al. Next-generation sequencing reveals genomic features in the Japanese quail. Genomics. 2013;101 6:345-53.
4. Jetz W, Thomas G, Joy J, Hartmann K and Mooers A. The global diversity of birds in space and time. Nature. 2012;491 7424:444-8.
5. Slack KE, Jones CM, Ando T, Harrison GA, Fordyce RE, Arnason U, et al. Early penguin fossils, plus mitochondrial genomes, calibrate avian evolution. Mol Biol Evol. 2006;23 6:1144-55.
6. van Tuinen M and Dyke GJ. Calibration of galliform molecular clocks using multiple fossils and genetic partitions. Mol Phylogenet Evol. 2004;30 1:74-86.
7. van Tuinen M and Hedges SB. Calibration of avian molecular clocks. Mol Biol Evol. 2001;18 2:206-13.
8. Kayang BB, Fillon V, Inoue-Murayama M, Miwa M, Leroux S, Fève K, et al. Integrated maps in quail (Coturnix japonica) confirm the high degree of synteny conservation with chicken (Gallus gallus) despite 35 million years of divergence. BMC Genomics. 2006;7 1:1.
9. Shibusawa M, Minai S, Nishida-Umehara C, Suzuki T, Mano T, Yamada K, et al. A comparative cytogenetic study of chromosome homology between chicken and Japanese quail. Cytogenet Genome Res. 2002;95 1-2:103-9.
10. Hazard D, Couty M, Faure J and Guémené D. Relationship between hypothalamic-pituitary-adrenal axis responsiveness and age, sexual maturity status, and sex in Japanese quail selected for long or short duration of tonic immobility. Poult Sci. 2005;84 12:1913-9.
11. Siopes T. Initiation of egg production by turkey breeder hens: Sexual maturation and age at lighting. Poult Sci. 2010;89 7:1490-6.
12. Caetano-Anolles K, Seo M, Rodriguez-Zas S, Oh J-D, Han JY, Lee K, et al. Comprehensive Identification of Sexual Dimorphism-Associated Differentially Expressed Genes in Two-Way Factorial Designed RNA-Seq Data on Japanese Quail (Coturnix coturnix japonica). PLoS ONE. 2015;10 9:e0139324.
13. Ricklefs R. Patterns of growth in birds. II. Growth rate and mode of development. Ibis. 1973;115 2:177-201.
14. Moudgal R. Female quails are heavier than male: Cause of uniqueness. Indian Journal of Poultry Science. 1997;32 1:81-3.
15. Satterlee D, Parker L, Castille S, Cadd G and Jones R. Struggling behavior in shackled male and female broiler chickens. Poultry science. 2000;79 5:652-5.
16. Chang G, Chang H, Zhenl H, Liu X, Sun W, Geng R, et al. Study on Phylogenetic Relationship Between Wild Japanese Quails in the Weisl1a| 1 Lake Area and Domestic Quails. 2001.
17. Cécilia H, Catherine Gh, Sophie L and Jean-Pierre R. Daily organization of laying in Japanese and European quail: Effect of domestication. Journal of Experimental Zoology Part A: Comparative Experimental Biology. 2004;301 2:186-94.

18. Mills AD, Crawford LL, Domjan M and Faure JM. The behavior of the Japanese or domestic quail Coturnix japonica. Neurosci Biobehav Rev. 1997;21 3:261-81.
19. Andersson MB. Sexual selection. Princeton University Press; 1994.
20. Hill G and McGraw K. Bird coloration. Function and evolution, vol. 2. Cambridge, MA: Harvard University Press, 2006.
21. Theron E, Hawkins K, Bermingham E, Ricklefs RE and Mundy NI. The molecular basis of an avian plumage polymorphism in the wild: a melanocortin-1-receptor point mutation is perfectly associated with the melanic plumage morph of the bananaquit, Coereba flaveola. Curr Biol. 2001;11 8:550-7.
22. Mundy NI, Badcock NS, Hart T, Scribner K, Janssen K and Nadeau NJ. Conserved genetic basis of a quantitative plumage trait involved in mate choice. Science. 2004;303 5665:1870-3.
23. Nadeau NJ, Burke T and Mundy NI. Evolution of an avian pigmentation gene correlates with a measure of sexual selection. Proc R Soc Lond B Biol Sci. 2007;274 1620:1807-13.
24. Ottinger MA, Abdelnabi M, Li Q, Chen K, Thompson N, Harada N, et al. The Japanese quail: a model for studying reproductive aging of hypothalamic systems. Exp Gerontol. 2004;39 11:1679-93.
25. Yasuo S, Ebihara S and Yoshimura T. Circadian expression of clock gene in the optic tectum of Japanese quail. Brain Res. 2004;1005 1:193-6.
26. Creuzet S, Schuler B, Couly G and Le Douarin NM. Reciprocal relationships between Fgf8 and neural crest cells in facial and forebrain development. Proc Natl Acad Sci U S A. 2004;101 14:4843-7.
27. Nain S, Bour A, Chalmers C and Smits J. Immunotoxicity and disease resistance in Japanese quail (Corturnix coturnix japonica) exposed to malathion. Ecotoxicology. 2011;20 4:892-900.
28. Kimball RT and Braun EL. Does more sequence data improve estimates of galliform phylogeny? Analyses of a rapid radiation using a complete data matrix. PeerJ. 2014;2:e361.
29. Pereira SL and Baker AJ. A molecular timescale for galliform birds accounting for uncertainty in time estimates and heterogeneity of rates of DNA substitutions across lineages and sites. Mol Phylogenet Evol. 2006;38 2:499-509.
30. Kaiser VB, van Tuinen M and Ellegren H. Insertion events of CR1 retrotransposable elements elucidate the phylogenetic branching order in galliform birds. Mol Biol Evol. 2007;24 1:338-47.
31. Guan X, Silva P, Gyenai KB, Xu J, Geng T, Tu Z, et al. The mitochondrial genome sequence and molecular phylogeny of the turkey, Meleagris gallopavo. Anim Genet. 2009;40 2:134-41.
32. Luo R, Liu B, Xie Y, Li Z, Huang W, Yuan J, et al. SOAPdenovo2: an empirically improved memory-efficient short-read de novo assembler. GigaScience. 2012;1 1:1.
33. Recoquillay J, Pitel F, Arnould C, Leroux S, Dehais P, Moréno C, et al. A medium density genetic map and QTL for behavioral and production traits in Japanese quail. BMC Genomics. 2015;16 1:1.
34. Zhang G, Li C, Li Q, Li B, Larkin DM, Lee C, et al. Comparative genomics reveals insights into avian genome evolution and adaptation. Science. 2014;346 6215:1311-20.
35. Nadeau N, Mundy N, Gourichon D and Minvielle F. Association of a single-nucleotide substitution in TYRP1 with roux in Japanese quail (Coturnix japonica). Anim Genet. 2007;38 6:609-13.
36. Aslam ML, Bastiaansen JW, Elferink MG, Megens H-J, Crooijmans RP, Blomberg LA, et al. Whole genome SNP discovery and analysis of genetic diversity in Turkey (Meleagris gallopavo). BMC genomics. 2012;13 1:391.
37. Guo X, Fang Q, Ma C, Zhou B, Wan Y and Jiang R. Whole-genome resequencing of

Xishuangbanna fighting chicken to identify signatures of selection. Genetics Selection Evolution. 2016;48 1:62.

38. Watterson G. On the number of segregating sites in genetical models without recombination. Theoretical population biology. 1975;7 2:256-76.
39. Tajima F. Evolutionary relationship of DNA sequences in finite populations. Genetics. 1983;105 2:437-60.
40. Huynh LY, Maney DL and Thomas JW. Contrasting population genetic patterns within the white-throated sparrow genome (Zonotrichia albicollis). Bmc Genetics. 2010;11 1:1-10.
41. Sundström H, Webster MT and Ellegren H. Reduced Variation on the Chicken Z Chromosome. Genetics. 2004;167 1:377.
42. Ellegren H. The different levels of genetic diversity in sex chromosomes and autosomes. Trends in Genetics Tig. 2009;25 6:278.
43. Alexander DH, Novembre J and Lange K. Fast model-based estimation of ancestry in unrelated individuals. Genome research. 2009;19 9:1655-64.
44. Balakrishnan CN and Edwards SV. Nucleotide variation, linkage disequilibrium and founder-facilitated speciation in wild populations of the zebra finch (Taeniopygia guttata). Genetics. 2009;181 2:645-60.
45. Li M-H and Merilä J. Sex-specific population structure, natural selection, and linkage disequilibrium in a wild bird population as revealed by genome-wide microsatellite analyses. BMC Evol Biol. 2010;10 1:1.
46. Charlesworth B, Morgan M and Charlesworth D. The effect of deleterious mutations on neutral molecular variation. Genetics. 1993;134 4:1289-303.
47. Zeng K, Fu Y-X, Shi S and Wu C-I. Statistical tests for detecting positive selection by utilizing high-frequency variants. Genetics. 2006;174 3:1431-9.
48. Zeng K, Shi S and Wu C-I. Compound tests for the detection of hitchhiking under positive selection. Molecular biology and evolution. 2007;24 8:1898-908.
49. Ukena K, Ubuka T and Tsutsui K. Distribution of a novel avian gonadotropin-inhibitory hormone in the quail brain. Cell and tissue research. 2003;312 1:73-9.
50. Andrew A, Rawdon BB and Alison BC. Failure of insulin cells to develop in cultured embryonic chick pancreas: a model system for the detection of factors supporting insulin cell differentiation. In Vitro Cellular & Developmental Biology-Animal. 1994;30 10:664-70.
51. Kee AJ, Gunning PW and Hardeman EC. Diverse roles of the actin cytoskeleton in striated muscle. Journal of muscle research and cell motility. 2009;30 5-6:187.
52. Ipek A and Dikmen BY. The relationship between growth traits and egg weight in pheasants (P. colchicus). Journal of Biological and Environmental Sciences. 2007;1 3.
53. Xu Y, Zhang X-H and Pang Y-Z. Association of Tyrosinase (TYR) and Tyrosinase-related Protein 1 (TYRP1) with Melanic Plumage Color in Korean Quails (Coturnix coturnix). Asian-Australasian Journal of Animal Sciences. 2013;26 11:1518-22. doi:10.5713/ajas.2013.13162.
54. Cui H, Zheng M, Zhao G, Liu R and Wen J. Identification of differentially expressed genes and pathways for intramuscular fat metabolism between breast and thigh tissues of chickens. BMC genomics. 2018;19 1:55.
55. Shen S-T and Yu JY-L. Cloning and gene expression of a cDNA for the chicken follicle-stimulating hormone (FSH)-β-subunit. General and comparative endocrinology. 2002;125 3:375-86.
56. Wang Y, Fortin J, Lamba P, Bonomi M, Persani L, Roberson MS, et al. Activator protein-1 and

smad proteins synergistically regulate human follicle-stimulating hormone beta-promoter activity. Endocrinology. 2008;149 11:5577-91. doi:10.1210/en.2008-0220.

57. Apter D. Development of the hypothalamic-pituitary-ovarian axis. Ann N Y Acad Sci. 1997;816:9-21.
58. Jiang X, Liu H, Chen X, Chen P-H, Fischer D, Sriraman V, et al. Structure of follicle-stimulating hormone in complex with the entire ectodomain of its receptor. Proceedings of the National Academy of Sciences. 2012;109 31:12491-6.
59. Suh P-G, Park J-I, Manzoli L, Cocco L, Peak JC, Katan M, et al. Multiple roles of phosphoinositide-specific phospholipase C isozymes. 2008.
60. Kiesel L, Przylipiak A, Emig E, Rabe T and Runnebaum B. Stimulation of gonadotropin release by arachidonic acid and its lipoxygenase metabolites in superfused pituitary cells. Life Sci. 1987;40 9:847-51.
61. Alder JK, Georgantas RW, Hildreth RL, Kaplan IM, Morisot S, Yu X, et al. Kruppel-like factor 4 is essential for inflammatory monocyte differentiation in vivo. J Immunol. 2008;180 8:5645-52.
62. Luo W-W, Lian H, Zhong B, Shu H-B and Li S. Krüppel-like factor 4 negatively regulates cellular antiviral immune response. Cell Mol Immunol. 2016;13 1:65-72.
63. Chen G, Wan H, Luo F, Zhang L, Xu Y, Lewkowich I, et al. Foxa2 programs Th2 cell-mediated innate immunity in the developing lung. J Immunol. 2010;184 11:6133-41.
64. Aki D, Zhang W and Liu YC. The E3 ligase Itch in immune regulation and beyond. Immunol Rev. 2015;266 1:6-26.
65. Daumke O and Knittler MR. Functional asymmetry of the ATP-binding-cassettes of the ABC transporter TAP is determined by intrinsic properties of the nucleotide binding domains. Eur J Biochem. 2001;268 17:4776-86.
66. Fulton JE, Thacker EL, Bacon LD and Hunt HD. Functional analysis of avian class I (BFIV) glycoproteins by epitope tagging and mutagenesis in vitro. Eur J Immunol. 1995;25 7:2069-76.
67. Zhou X and Stephens M. Genome-wide efficient mixed-model analysis for association studies. Nat Genet. 2012;44 7:821-4.
68. Minvielle F, Cecchi T, Passamonti P, Gourichon D and Renieri C. Plumage colour mutations and melanins in the feathers of the Japanese quail: a first comparison. Animal genetics. 2009;40 6:971-4.
69. Nishiwaki-Ohkawa T and Yoshimura T. Molecular basis for regulating seasonal reproduction in vertebrates. J Endocrinol. 2016;229 3:R117-27. doi:10.1530/JOE-16-0066.
70. Wang N, Kimball RT, Braun EL, Liang B and Zhang Z. Assessing phylogenetic relationships among Galliformes: a multigene phylogeny with expanded taxon sampling in Phasianidae. PLoS One. 2013;8 5:e64312.
71. Jarvis ED, Mirarab S, Aberer AJ, Li B, Houde P, Li C, et al. Whole-genome analyses resolve early branches in the tree of life of modern birds. Science. 2014;346 6215:1320-31. doi:10.1126/science.1253451.
72. Simao FA, Waterhouse RM, Ioannidis P, Kriventseva EV and Zdobnov EM. BUSCO: assessing genome assembly and annotation completeness with single-copy orthologs. Bioinformatics. 2015;31 19:3210-2. doi:10.1093/bioinformatics/btv351.
73. Tarailo-Graovac M and Chen N. Using RepeatMasker to identify repetitive elements in genomic sequences. Current Protocols in Bioinformatics. 2009:4.10. 1-4.. 4.
74. Jurka J, Kapitonov VV, Pavlicek A, Klonowski P, Kohany O and Walichiewicz J. Repbase Update, a

database of eukaryotic repetitive elements. Cytogenet Genome Res. 2005;110 1-4:462-7.
75. Xu Z and Wang H. LTR_FINDER: an efficient tool for the prediction of full-length LTR retrotransposons. Nucleic Acids Res. 2007;35 suppl 2:W265-W8.
76. Benson G. Tandem repeats finder: a program to analyze DNA sequences. Nucleic Acids Res. 1999;27 2:573.
77. Aken BL, Ayling S, Barrell D, Clarke L, Curwen V, Fairley S, et al. The Ensembl gene annotation system. Database : the journal of biological databases and curation. 2016;2016 doi:10.1093/database/baw093.
78. Kent WJ. BLAT--the BLAST-like alignment tool. Genome research. 2002;12 4:656-64. doi:10.1101/gr.229202. Article published online before March 2002.
79. Birney E, Clamp M and Durbin R. GeneWise and genomewise. Genome Res. 2004;14 5:988-95.
80. Stanke M, Keller O, Gunduz I, Hayes A, Waack S and Morgenstern B. AUGUSTUS: ab initio prediction of alternative transcripts. Nucleic Acids Res. 2006;34 suppl 2:W435-W9.
81. Burge C and Karlin S. Prediction of complete gene structures in human genomic DNA. J Mol Biol. 1997;268 1:78-94.
82. Trapnell C, Pachter L and Salzberg SL. TopHat: discovering splice junctions with RNA-Seq. Bioinformatics. 2009;25 9:1105-11.
83. Trapnell C, Williams BA, Pertea G, Mortazavi A, Kwan G, Van Baren MJ, et al. Transcript assembly and quantification by RNA-Seq reveals unannotated transcripts and isoform switching during cell differentiation. Nat Biotechnol. 2010;28 5:511-5.
84. Elsik CG, Mackey AJ, Reese JT, Milshina NV, Roos DS and Weinstock GM. Creating a honey bee consensus gene set. Genome Biol. 2007;8 1:1.
85. Gilbert, M, P; Li, C; Wang, J; Zhang, G. Genomic data from the domestic pigeon (Columba livia). GigaScience database 2011. http://dx.doi.org/10.5524/100007
86. Bruford, M, W; Cao, C; Chen, Y; Chen, Y; Dixon, A; Fox, N, et al. Genomic data from the saker falcon (Falco cherrug). GigaScience Database 2014. http://dx.doi.org/10.5524/100075
87. Bruford, M, W; Cao, C; Chen, Y; Chen, Y; Dixon, A; Fox, N, et al. Genomic data from the peregrine falcon (Falco peregrinus). GigaScience Database 2014. http://dx.doi.org/10.5524/101006
88. The genome of Darwin's Finch (Geospiza fortis). Li, B; Li, H; Parker, P; Wang, J. The genome of Darwin's Finch (Geospiza fortis). GigaScience database 2012. http://dx.doi.org/10.5524/100040
89. Cai, Q; Cai, Y; Gou, C; Hao, M; He, R; Hui, Y, et al. Genomic data for the Tibetan ground tit (Pseudopoces humilis). GigaScience Database 2014. http://dx.doi.org/10.5524/100088
90. Ronquist F, Teslenko M, van der Mark P, Ayres DL, Darling A, Höhna S, et al. MrBayes 3.2: efficient Bayesian phylogenetic inference and model choice across a large model space. Syst Biol. 2012;61 3:539-42.
91. Guindon S, Delsuc F, Dufayard J-F and Gascuel O. Estimating maximum likelihood phylogenies with PhyML. Bioinformatics for DNA sequence analysis. 2009:113-37.
92. Yang Z. PAML: a program package for phylogenetic analysis by maximum likelihood. CABIOS 13, 555–556. Supplementary material The following supplementary material is available for this article online: Fig S. 1997;1.
93. Hedges SB, Dudley J and Kumar S. TimeTree: a public knowledge-base of divergence times among organisms. Bioinformatics. 2006;22 23:2971-2.
94. De Bie T, Cristianini N, Demuth JP and Hahn MW. CAFE: a computational tool for the study of gene family evolution. Bioinformatics. 2006;22 10:1269-71.

95. Löytynoja A. Phylogeny-aware alignment with PRANK. Multiple Sequence Alignment Methods. 2014:155-70.
96. Talavera G and Castresana J. Improvement of phylogenies after removing divergent and ambiguously aligned blocks from protein sequence alignments. Syst Biol. 2007;56 4:564-77.
97. Zhao H, Yang J-R, Xu H and Zhang J. Pseudogenization of the umami taste receptor gene Tas1r1 in the giant panda coincided with its dietary switch to bamboo. Mol Biol Evol. 2010;27 12:2669-73.
98. Yang Z. PAML 4: phylogenetic analysis by maximum likelihood. Molecular biology and evolution. 2007;24 8:1586-91.
99. Zhang J, Nielsen R and Yang Z. Evaluation of an improved branch-site likelihood method for detecting positive selection at the molecular level. Molecular biology and evolution. 2005;22 12:2472-9.
100. Suzuki Y and Nei M. False-positive selection identified by ML-based methods: examples from the Sig1 gene of the diatom Thalassiosira weissflogii and the tax gene of a human T-cell lymphotropic virus. Molecular Biology and Evolution. 2004;21 5:914-21.
101. Li H and Durbin R. Fast and accurate short read alignment with Burrows–Wheeler transform. Bioinformatics. 2009;25 14:1754-60.
102 Li H, Handsaker B, Wysoker A, Fennell T, Ruan J, Homer N, et al. The sequence alignment/map format and SAMtools. Bioinformatics. 2009;25 16:2078-9.
103. McKenna A, Hanna M, Banks E, Sivachenko A, Cibulskis K, Kernytsky A, et al. The Genome Analysis Toolkit: a MapReduce framework for analyzing next-generation DNA sequencing data. Genome Res. 2010;20 9:1297-303.
104. Purcell S, Neale B, Todd-Brown K, Thomas L, Ferreira MA, Bender D, et al. PLINK: a tool set for whole-genome association and population-based linkage analyses. Am J Hum Genet. 2007;81 3:559-75.
105. Tamura K, Stecher G, Peterson D, Filipski A and Kumar S. MEGA6: molecular evolutionary genetics analysis version 6.0. Mol Biol Evol. 2013;30 12:2725-9.
106. Weir BS and Cockerham CC. Estimating F-statistics for the analysis of population structure. evolution. 1984:1358-70.
107. Barrett JC, Fry B, Maller J and Daly MJ. Haploview: analysis and visualization of LD and haplotype maps. Bioinformatics. 2005;21 2:263-5.
108. Wu Y, Zhang Y, Hou Z, Fan G, Pi J, Sun S, et al. Supporting data for "Population genomic data reveal genes related to important traits of quail". GigaScience Database 2018. http://dx.doi.org/10.5524/100441.

Table 1 Statistics of SNPs in different genome regions of wild and domesticated quails' subpopulations

Region	Subpopulation	Autosomes				Chromosome Z			
		# of SNPs	π (10^{-3})	θ_w (10^{-3})	Tajima's *D*	# of SNPs	π (10^{-3})	θ_w (10^{-3})	Tajima's *D*
Exon*	Wild	520,747	7.324	8.979	-0.502	13,963	5.191	5.940	-0.309
	Egg-type	320,629	7.206	6.449	0.256	7,201	5.448	4.479	0.425
	Meat-type	282,019	7.152	6.238	0.331	6,967	5.120	4.549	0.275
Intron*	Wild	13,632,632	11.868	14.302	-0.548	555,643	8.100	9.197	-0.354
	Egg-type	8,468,826	10.735	9.668	0.270	285,930	7.934	6.483	0.489
	Meat-type	7,418,909	10.167	9.005	0.299	278,361	8.546	7.482	0.365
All regions*	Wild	33,744,246	8.679	10.709	-0.763	1,438,213	5.813	6.683	-0.628
	Egg-type	20,848,043	7.338	6.617	0.467	749,670	4.031	3.389	0.666
	Meat-type	18,250,531	6.901	6.052	0.600	716,433	3.843	3.328	0.593

* all regions refers to the total genome regions; Exon and Intron refer to exon and intron regions respectively.

Figure 1

Click here to download Figure Figure. 1-updated.pdf

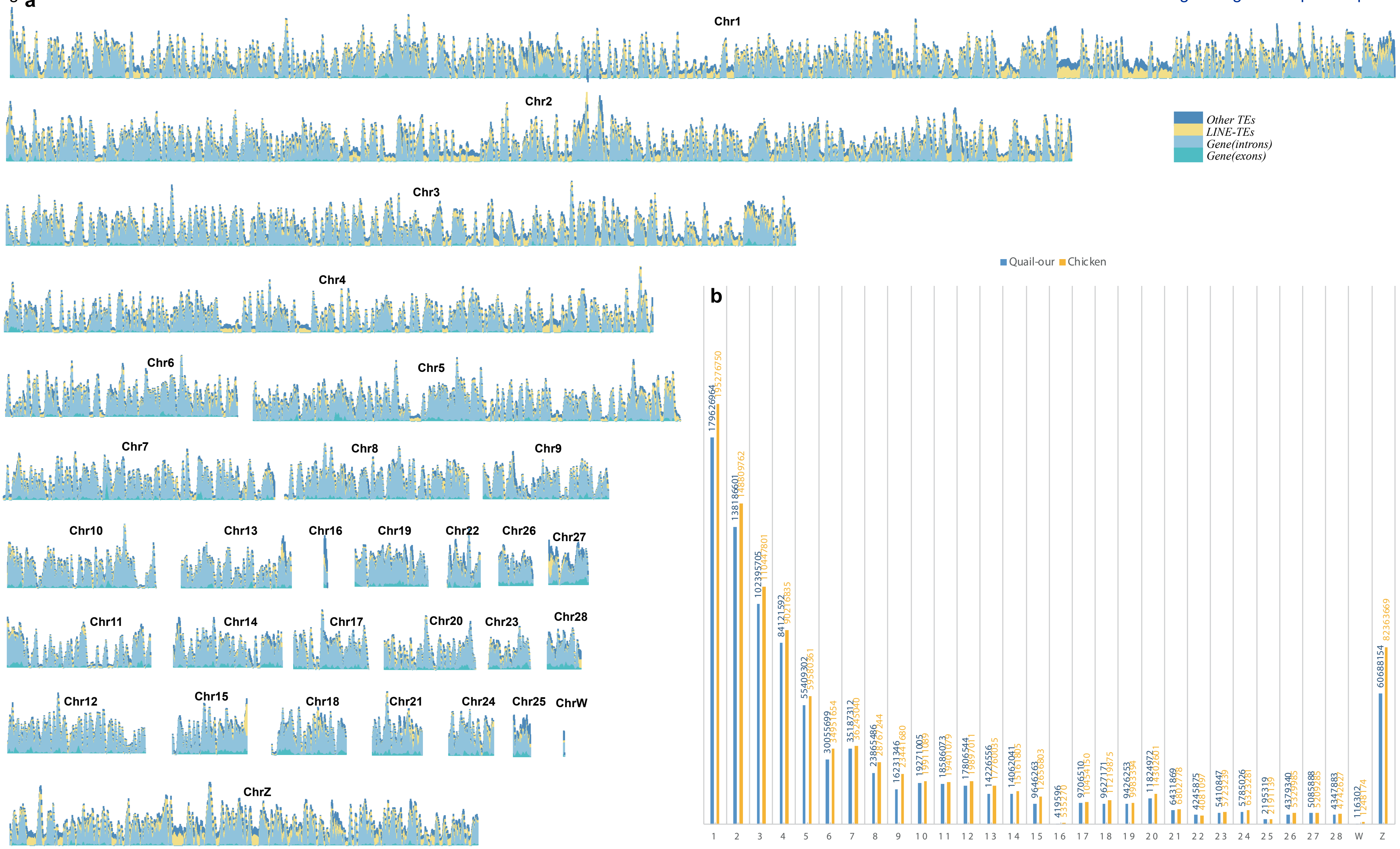

Figure 2

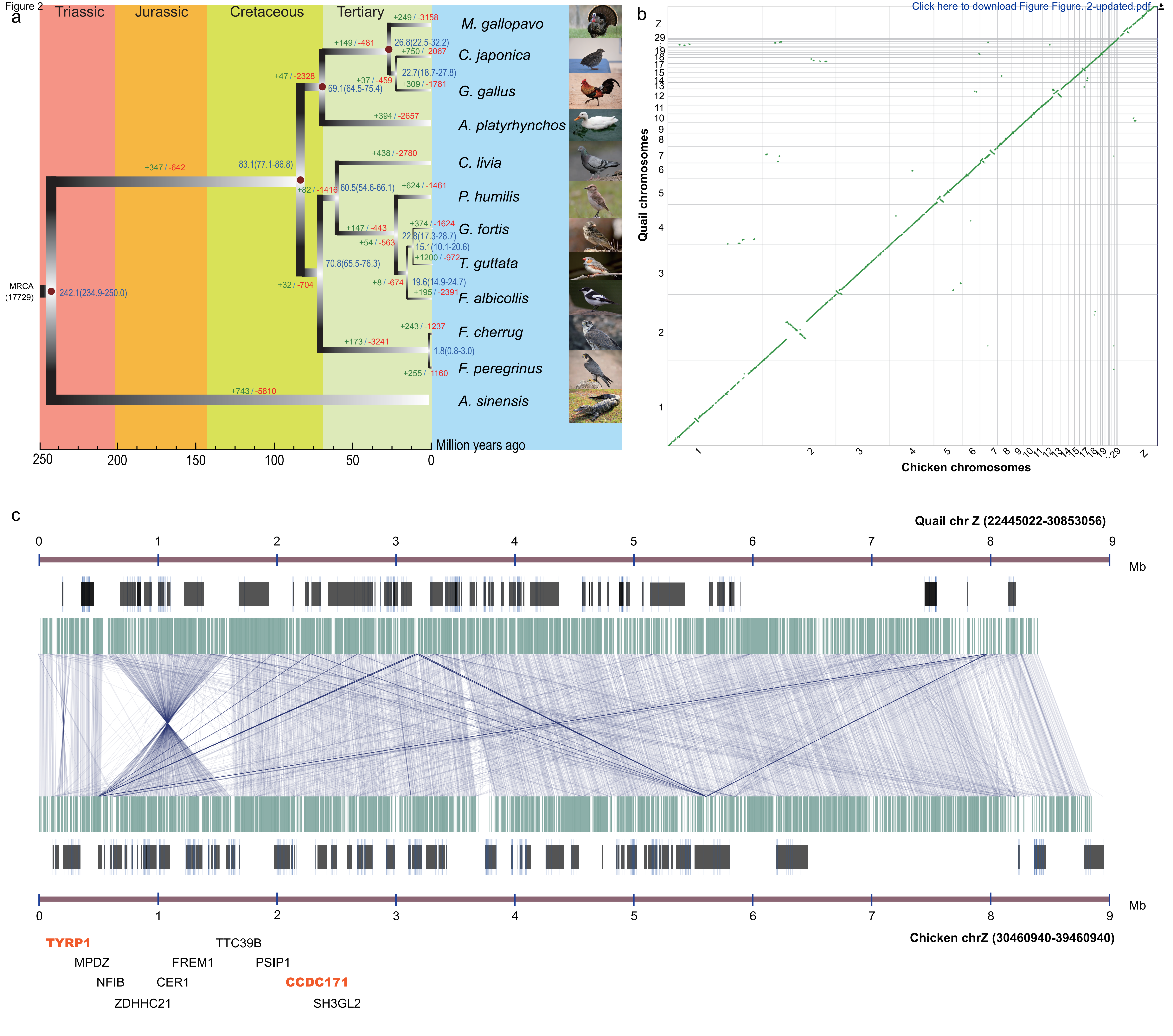

a
Triassic
Jurassic
Cretaceous
Tertiary
M. gallopavo
C. japonica
G. gallus
A. platyrhynchos
C. livia
P. humilis
G. fortis
T. guttata
F. albicollis
F. cherrug
F. peregrinus
A. sinensis
MRCA (17729)
242.1(234.9-250.0)
83.1(77.1-86.8)
69.1(64.5-75.4)
26.8(22.5-32.2)
22.7(18.7-27.8)
60.5(54.6-66.1)
70.8(65.5-76.3)
22.8(17.3-28.7)
15.1(10.1-20.6)
19.6(14.9-24.7)
1.8(0.8-3.0)
Million years ago
250 200 150 100 50 0
b
Quail chromosomes
Chicken chromosomes
Click here to download Figure Figure. 2-updated.pdf
c
Quail chr Z (22445022-30853056)
Chicken chrZ (30460940-39460940)
Mb
TYRP1
MPDZ
NFIB
ZDHHC21
FREM1
CER1
TTC39B
PSIP1
CCDC171
SH3GL2

Figure 3

Click here to download Figure Figure. 3-updated.pdf

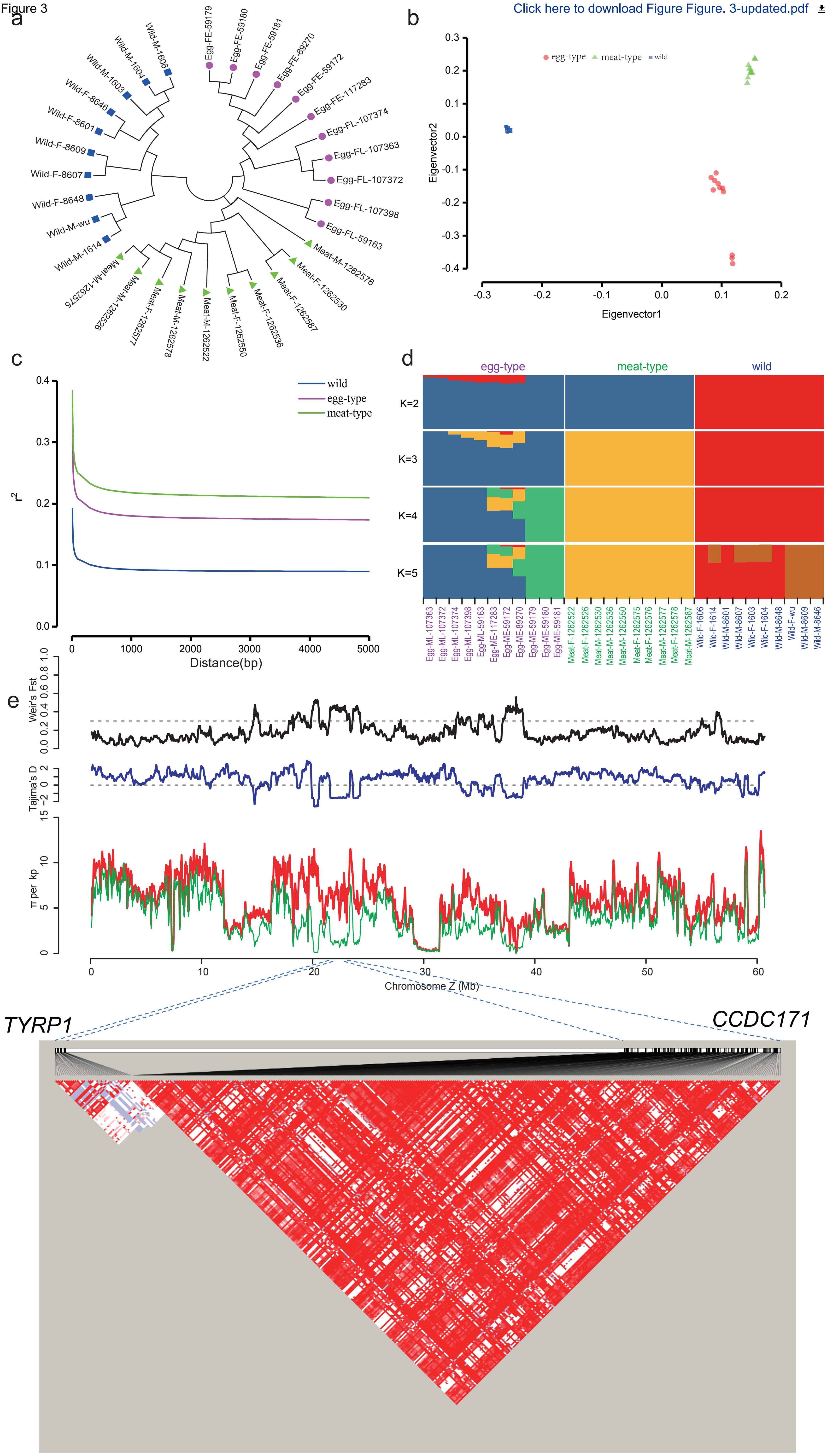

Click here to download Figure Figure. 4-updated.pdf

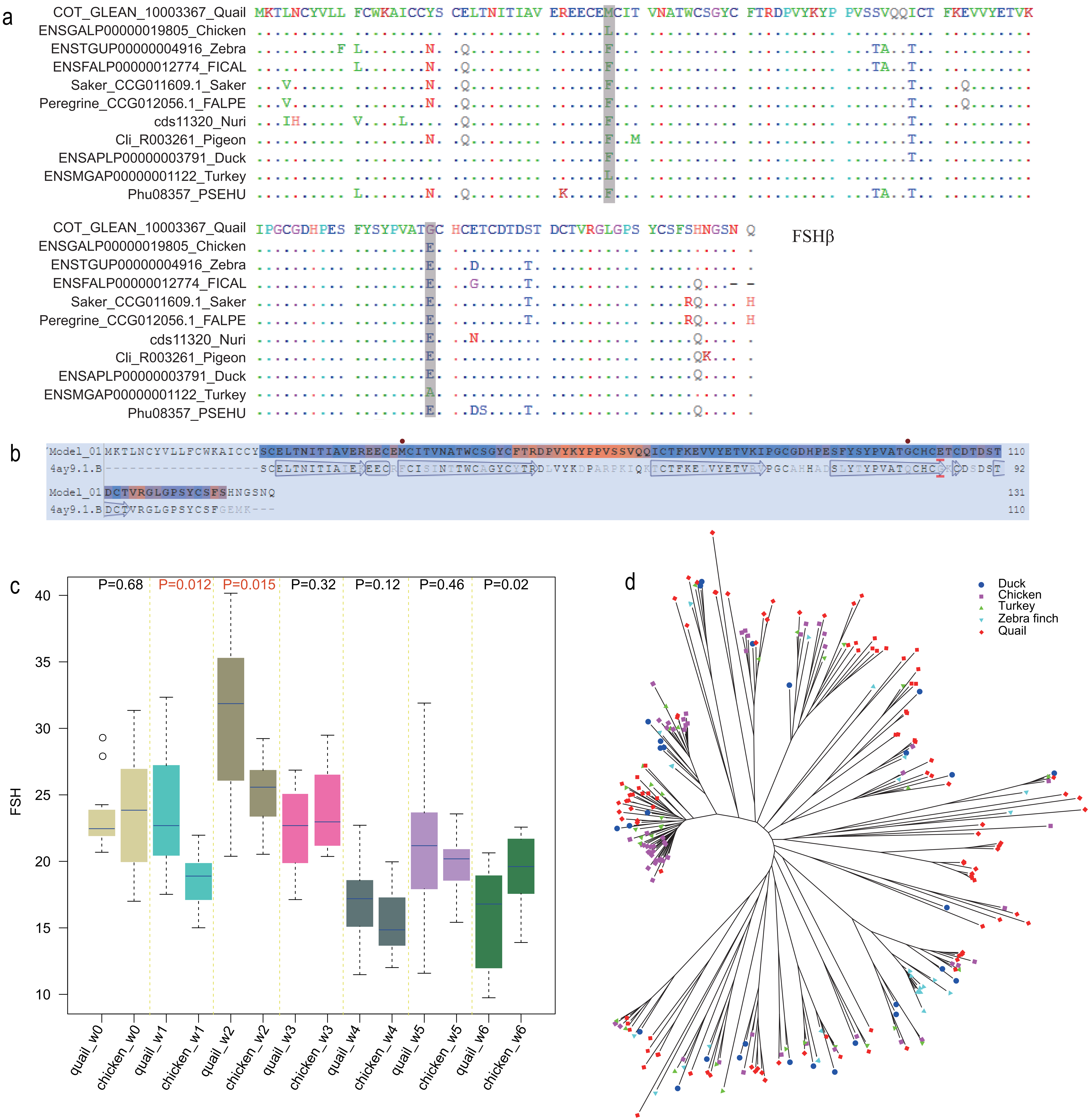

Figure 5

Click here to download Figure Figure. 5-updated.pdf

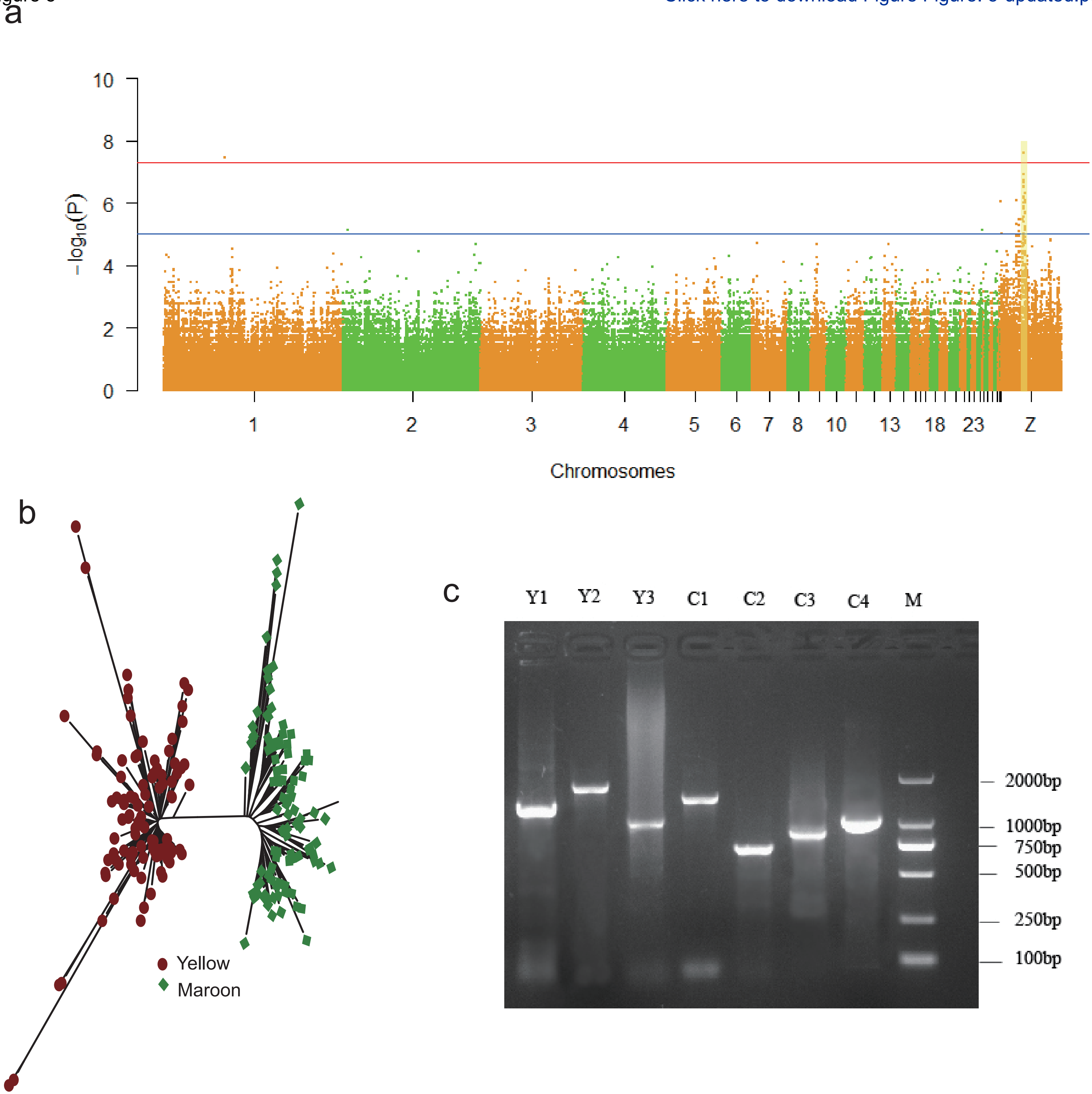

Click here to access/download
Supplementary Material
Additional file 1-updated.docx

Click here to access/download
Supplementary Material
Table S13 diversity statistics of 31 quails-updated.xlsx

Click here to access/download
Supplementary Material
Table S14-updated.xlsx

Click here to access/download
Supplementary Material
Table.S15-updated.xls

Click here to access/download
Supplementary Material
Table S16-updated.xlsx

Click here to access/download
Supplementary Material
Table.S17-updated.xls

Click here to access/download
Supplementary Material
Table S19-updated.xlsx

Supplementary Material

Click here to access/download
Supplementary Material
Respond to Reviewers.docx
