# Supplementary material for: Population genomic data reveal genes related to important traits of quail
Source: Gigascience. 2018 May 11;7(5):giy049. doi: 10.1093/gigascience/giy049 (PMC5961004; doi:10.1093/gigascience/giy049)
Supplement: Reviewer_3_Original_Submission_Attachment_Reviewer_Figures.pdf [file giy049_reviewer_3_original_submission_attachment_reviewer_figures.pdf]

Reviewer Figure 1

>GNRH1_quail2_region
VFLLLLWENLPPVQAGKAREGWVRLVGEKRQESLVHMWQSQLCITLGYVQEYDYINLDAPAVTMSLLTELKP

G.gallus gene for gonadotrophin releasing hormone I
Sequence ID: X69491.1Length: 6282Number of Matches: 1
Related Information
Gene-associated gene details
Map Viewer-aligned genomic context
Range 1: 4404 to 4619GenBankGraphicsNext MatchPrevious Match

Alignment statistics for match #1

Score	Expect	Method	Identities	Positives	Gaps	Fran
120 bits(300)	3e-31	Compositional matrix adjust.	60/72(83%)	62/72(86%)	0/72(0%)	+3

```
Query  1     VFLLLLWENLPPVQAGKAREGWVRLVGEKRQESLVHMWQSQLCITLGYVQEYDYINLDAP  60
             VFLLLLWENL PVQAGKAR G V +VGEKRQESLVHMW SQLCITL YVQEYD+INLDAP
Sbjct  4404  VFLLLLWENLRPVQAGKARGGRV*VVGEKRQESLVHMWHSQLCITLRYVQEYDHINLDAP  4583

Query  61    AVTMSLLTELKP  72
              V   LLTELKP
```

Reviewer Figure 2

>PLCB4_fragment
AVAISVKKAVEDSEQENKKGLVTVEDEQA

PREDICTED: Taeniopygia guttata phospholipase C, beta 4 (PLCB4), mRNA
Sequence ID: XM_012572366.1Length: 5414Number of Matches: 1
Related Information
Gene-associated gene details
Range 1: 1689 to 1778GenBankGraphicsNext MatchPrevious Match

Score	Expect	Method	Identities	Positives	Gaps	F
52.0 bits(123)	5e-09	Compositional matrix adjust.	29/30(97%)	29/30(96%)	1/30(3%)	+

```
Query  1     AVAISVKKAVEDSEQE-NKKGLVTVEDEQA  29
             AVAISVKKAVEDSEQE NKKGLVTVEDEQA
Sbjct  1689  AVAISVKKAVEDSEQENNKKGLVTVEDEQA  1778
```

DownloadGenBankGraphicsNextPreviousDescriptions
PREDICTED: Anas platyrhynchos phospholipase C beta 4 (PLCB4), transcript variant X1, mRNA
Sequence ID: XM_013108930.2Length: 5533Number of Matches: 1
Related Information
Gene-associated gene details
Range 1: 1782 to 1871GenBankGraphicsNext MatchPrevious Match

Score	Expect	Method	Identities	Positives	Gaps	F
52.0 bits(123)	6e-09	Compositional matrix adjust.	29/30(97%)	29/30(96%)	1/30(3%)	+

```
Query  1     AVAISVKKAVEDSEQE-NKKGLVTVEDEQA  29
             AVAISVKKAVEDSEQE NKKGLVTVEDEQA
Sbjct  1782  AVAISVKKAVEDSEQENNKKGLVTVEDEQA  1871
```

DownloadGenBankGraphicsNextPreviousDescriptions
PREDICTED: Anas platyrhynchos phospholipase C beta 4 (PLCB4), transcript variant X7, mRNA
Sequence ID: XM_013108952.2Length: 5499Number of Matches: 1
Related Information

Gene-associated gene details
Range 1: 1785 to 1874GenBankGraphicsNext MatchPrevious Match

Score	Expect	Method	Identities	Positives	Gaps	F
52.0 bits(123)	6e-09	Compositional matrix adjust.	29/30(97%)	29/30(96%)	1/30(3%)	+

```
Query  1     AVAISVKKAVEDSEQE-NKKGLVTVEDEQA  29
             AVAISVKKAVEDSEQE NKKGLVTVEDEQA
Sbjct  1785  AVAISVKKAVEDSEQENNKKGLVTVEDEQA  1874
```

DownloadGenBankGraphicsNextPreviousDescriptions

PREDICTED: Anas platyrhynchos phospholipase C beta 4 (PLCB4), transcript variant X3, mRNA

Sequence ID: XM_021278910.1Length: 4145Number of Matches: 1

Related Information

Gene-associated gene details
Range 1: 2096 to 2185GenBankGraphicsNext MatchPrevious Match

Score	Expect	Method	Identities	Positives	Gaps	F
52.0 bits(123)	6e-09	Compositional matrix adjust.	29/30(97%)	29/30(96%)	1/30(3%)	+

```
Query  1     AVAISVKKAVEDSEQE-NKKGLVTVEDEQA  29
             AVAISVKKAVEDSEQE NKKGLVTVEDEQA
Sbjct  2096  AVAISVKKAVEDSEQENNKKGLVTVEDEQA  2185
```

DownloadGenBankGraphicsNextPreviousDescriptions

PREDICTED: Anas platyrhynchos phospholipase C beta 4 (PLCB4), transcript variant X5, mRNA

Sequence ID: XM_021278916.1Length: 4071Number of Matches: 1

Related Information

Gene-associated gene details
Range 1: 2022 to 2111GenBankGraphicsNext MatchPrevious Match

Score	Expect	Method	Identities	Positives	Gaps	F
52.0 bits(123)	6e-09	Compositional matrix adjust.	29/30(97%)	29/30(96%)	1/30(3%)	+

```
Query  1     AVAISVKKAVEDSEQE-NKKGLVTVEDEQA  29
             AVAISVKKAVEDSEQE NKKGLVTVEDEQA
Sbjct  2022  AVAISVKKAVEDSEQENNKKGLVTVEDEQA  2111

Sbjct  4584  TVITPLLTELKP  4619
```
