# Supplementary material for: Population genomic data reveal genes related to important traits of quail
Source: Gigascience. 2018 May 11;7(5):giy049. doi: 10.1093/gigascience/giy049 (PMC5961004; doi:10.1093/gigascience/giy049)
Supplement: Additional Files [file giy049_supp.zip › Additional file 1-updated.docx]

**Supplementary part 1: Figures**


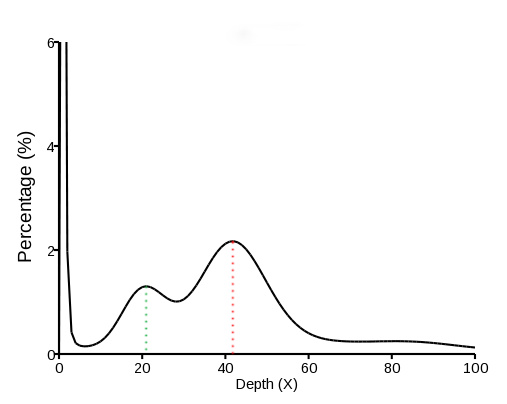


**Figure S1. 17-kmer analysis for estimation of genome size.** About 56.67 Gb of high-quality data from short-insert libraries was used for this analysis. The maximum peak (red dashed line) indicates Peak_depth and the estimated genome size is about 1.1 Gb. The second peak (green dashed line) suggests that the quail genome sample was heterozygous.

**

**

**Figure S2 Syntenic relationships between our assembled genome and NCBI genome (SAMN03989050).** Here we employed Lastz (v1.02.00) with parameters ‘T=2 C=2 H=2000 Y=3400 L=6000 K=2200’ to compare 30 chromosomes of the two genomes and then calculated the synteny ratio of them after excluding ‘N’ bases. The figure showed syntenic relationships of blocks more than 5 kb.

**
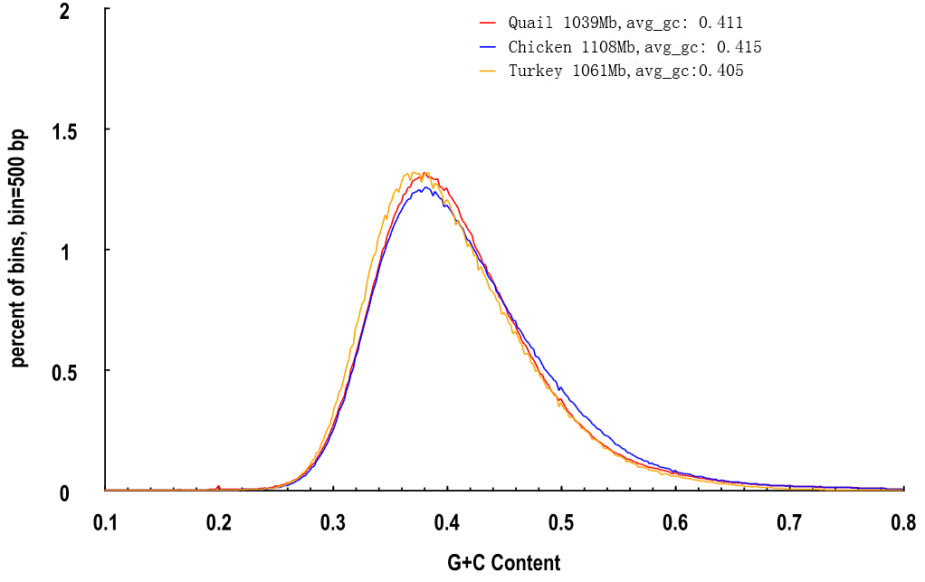
**

**Figure S3. GC content of quail, chicken and turkey genome assemblies.** GC-content (*x*-axis) quantified as final proportion of 500-bp windows or bins (*y*-axis). Quail (*Coturnix japonica*, chicken (*Gallus gallus*) and turkey (*Meleagris gallopavo*) have similar genomic GC contents, which is consistent with the location of these three species in one phylogenetic branch (Figure 2a)**.**

**
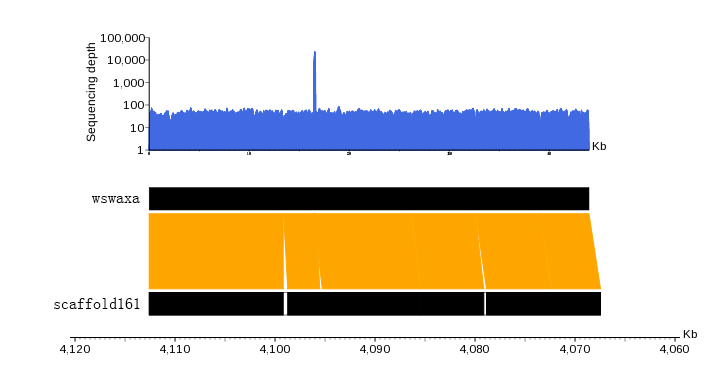
**

**
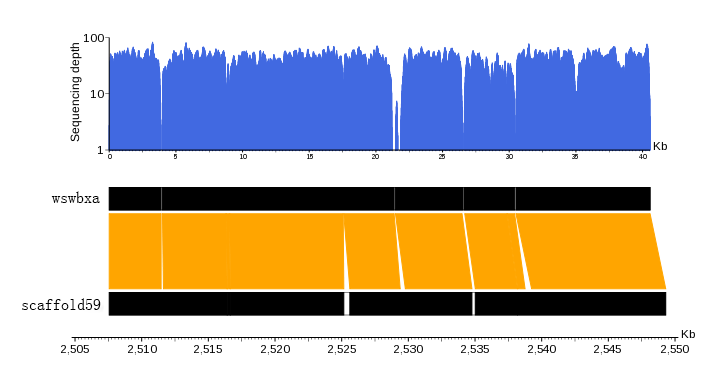
**

**
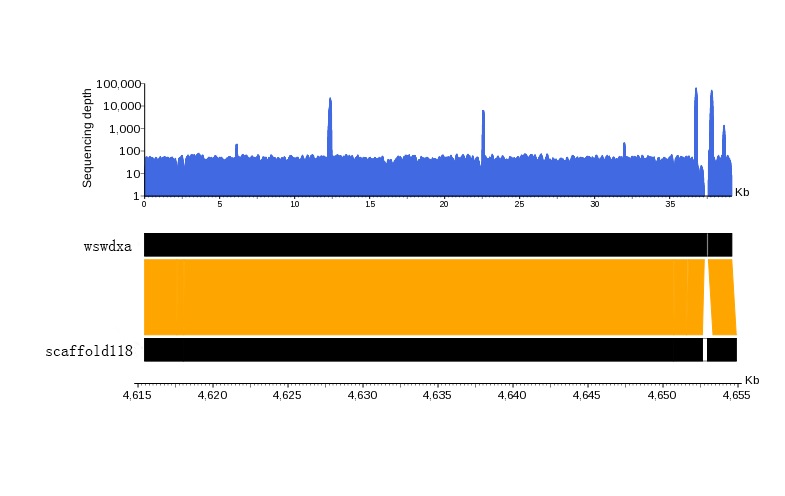
**

**
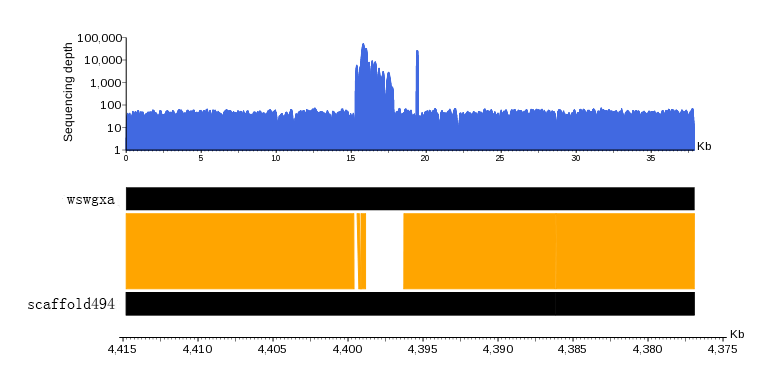
**

**
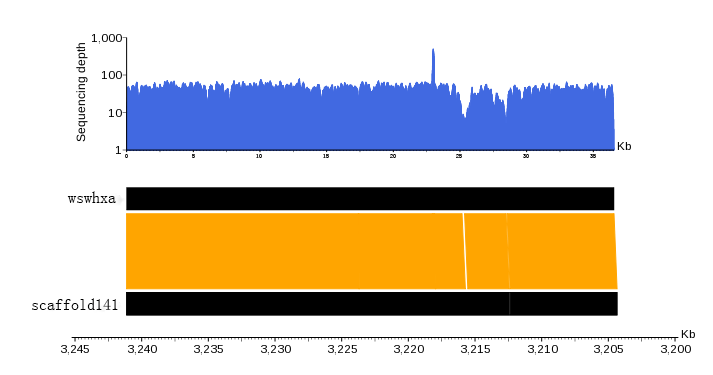
**

**
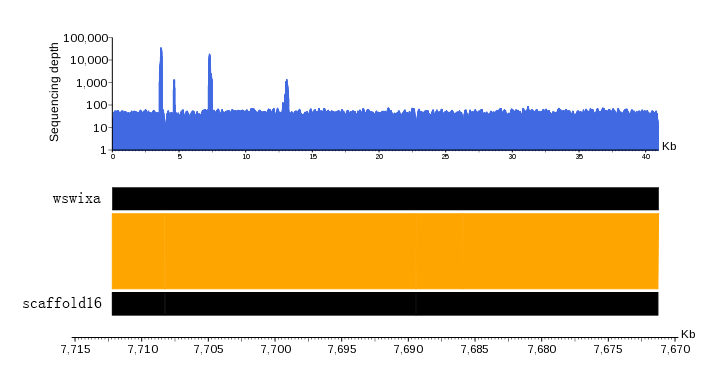
**

**
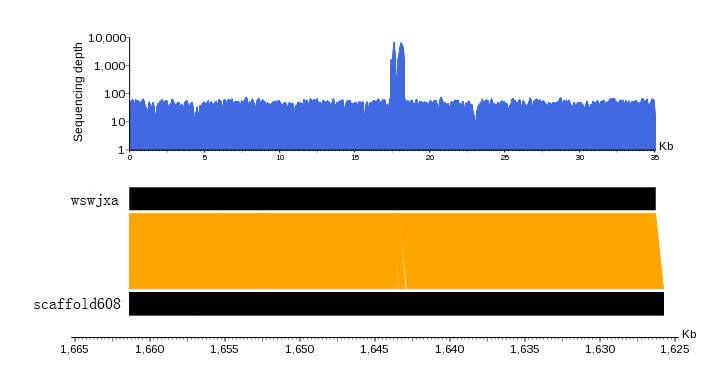
**

**Figure S4.** **Assembly evaluation from the sequence analysis of random fosmid clones.** Seven fosmid sequences were aligned to the assembled genome and displayed with a one-to-one correspondence as a single scaffold with some gaps. The white bars represent gaps and the yellow polygons represent regions of good alignment of the fosmid clone sequence with the scaffold sequences. Sequencing depths were calculated by mapping short reads to the fosmid sequences. All seven fosmid sequences showed conserved synteny with and high coverage of the assembled quail genome (**Table S3**).

**
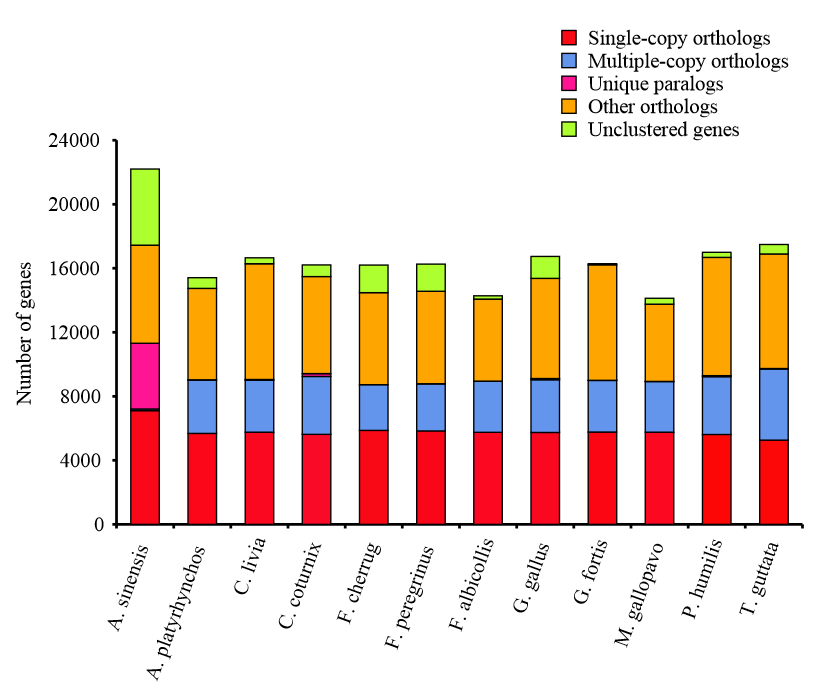
**

**Figure S5. Comparison of** **orthologous genes among 12 species.** The number of orthologous genes in 12 species including *Coturnix japonica* (quail), *Gallus gallus* (chicken), *Anas platyrhynchos* (duck), *Columba livia* (pigeon), *Falco cherrug* (Saker falcon), *Falco peregrinus* (Peregrine falcon), *Ficedula albicollis* (collared flycatcher), *Geospiza fortis* (medium ground finch), *Meleagris gallopavo* (turkey), *Pseudopodoces humilis* (ground tit), *Taeniopygia guttata* (zebra finch), with *Alligator sinensis* (Chinese alligator) as an outgroup, are shown. Single-copy orthologs represent conserved single-copy genes (1:1:1) in each species. Multiple-copy orthologs represent genes with multiple copies. Unique paralogs represent unique genes in each species.

**
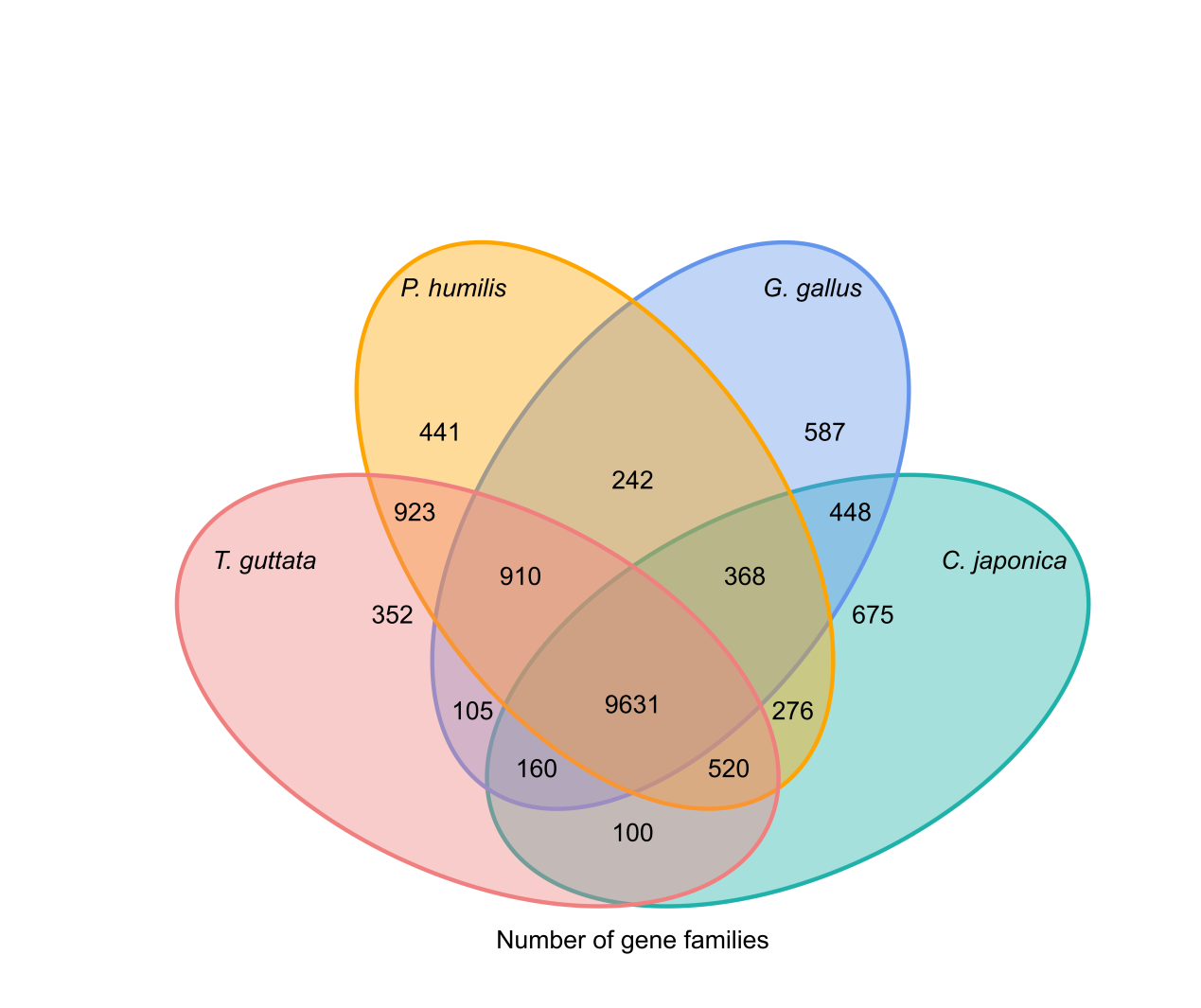
**

**Figure S6. Shared and specific orthologous genes for four avian species.** The numbers of genes shared between, or unique to, four avian species including *Coturnix japonica*, *Gallus gallus* (chicken), *Pseudopodoces humilis* (ground tit), *Taeniopygia guttata* (zebra finch). The quail genome contains more species-specific gene families than do the genomes of the other bird species.

**
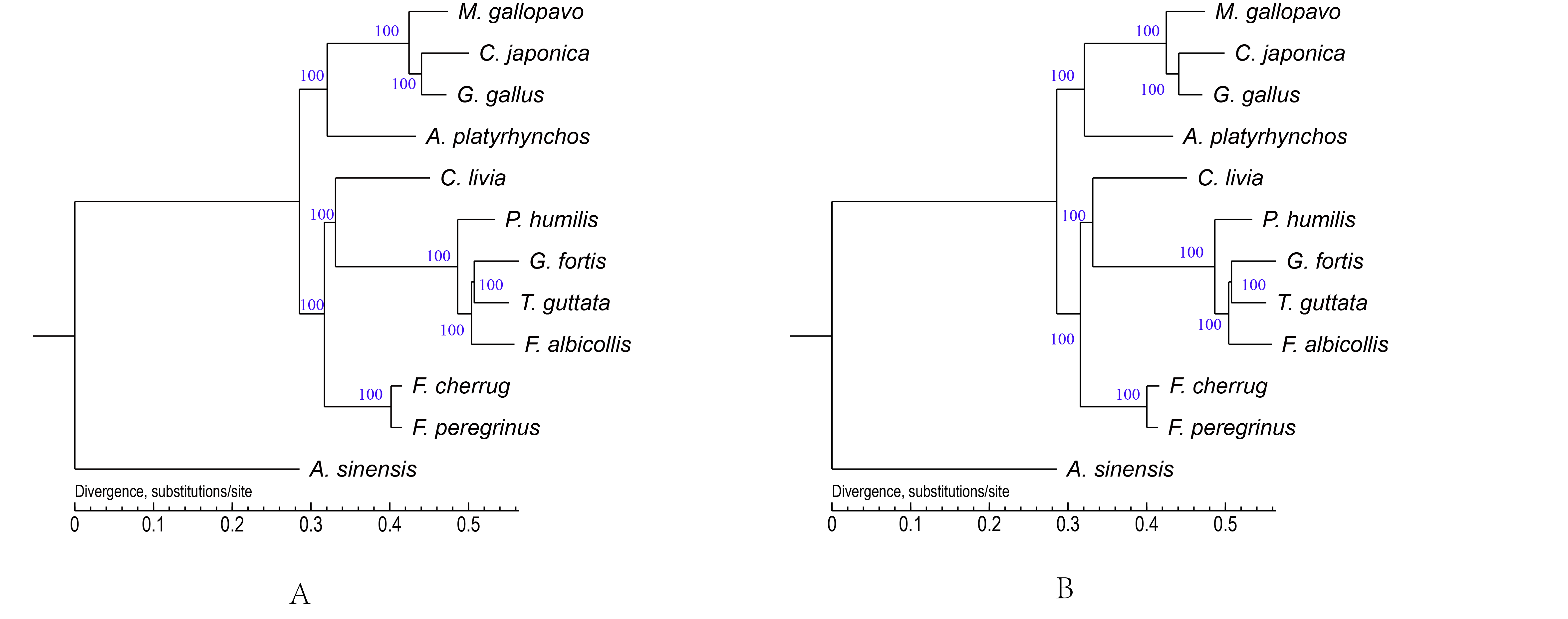
**

**Figure S7. Phylogenetic trees generated using MrBayes and PhyML based on fourfold degenerate (4D) sites.** The tree in panel A represents a tree generated using a GTR (General Time-Reversible) model in MrBayes. The tree shown in panel B represents a tree generated using a GTR model in PhyML. Species in the phylogeny include *Coturnix japonica* (quail), *Gallus gallus* (chicken), *Anas platyrhynchos* (duck), *Columba livia* (pigeon), *Falco cherrug* (Saker falcon), *Falco peregrinus* (Peregrine falcon), *Ficedula albicollis* (collared flycatcher), *Geospiza fortis* (medium ground finch), *Meleagris gallopavo* (turkey), *Pseudopodoces humilis* (ground tit), *Taeniopygia guttata* (zebra finch), with *Alligator sinensis* (Chinese alligator) as an outgroup.

**
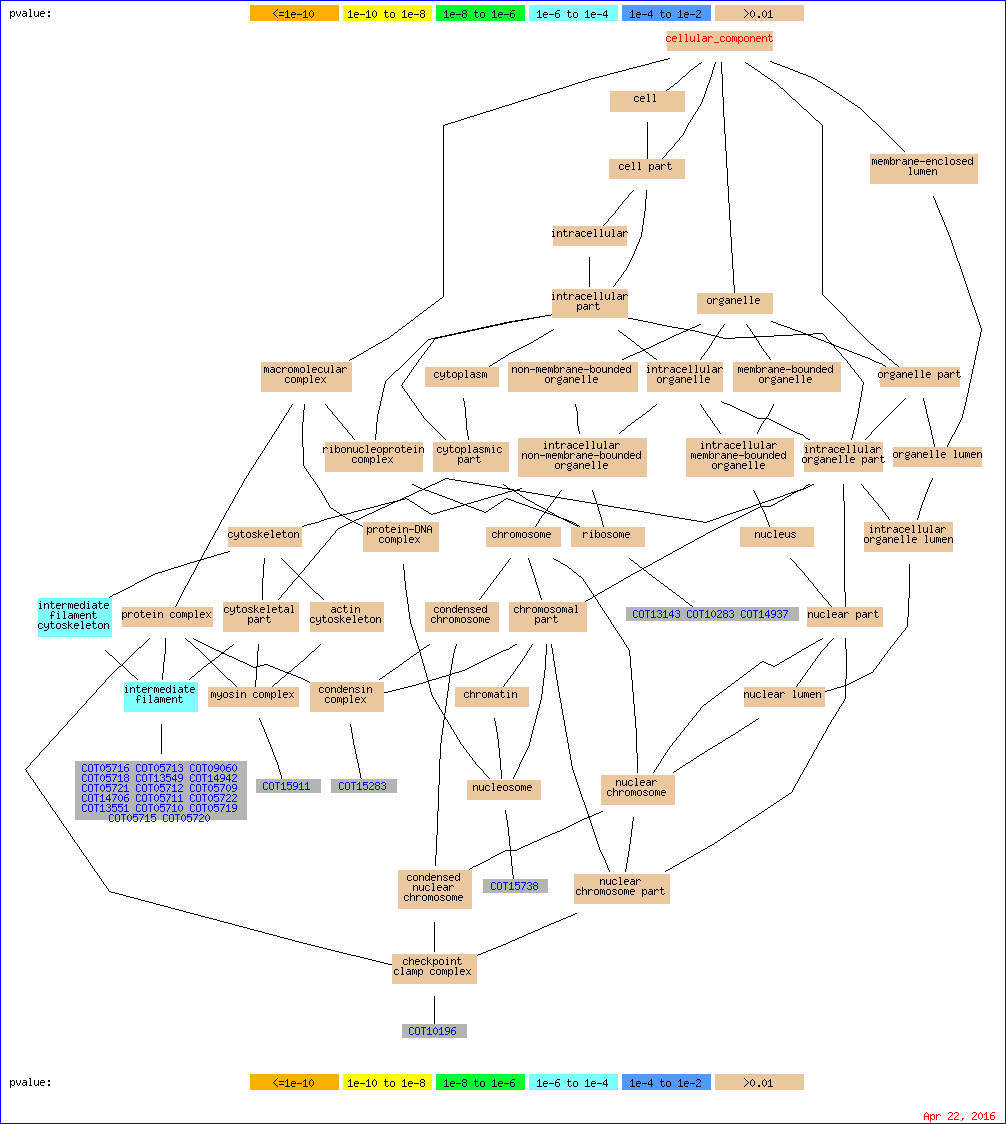
**

**Figure S8.** GO enrichment results (C) for genes located within the 1-kb regions flanking breakpoints of 131 large inversions in quail (*Coturnix japonica*) genome relative to that of chicken (*Gallus gallus*).

**
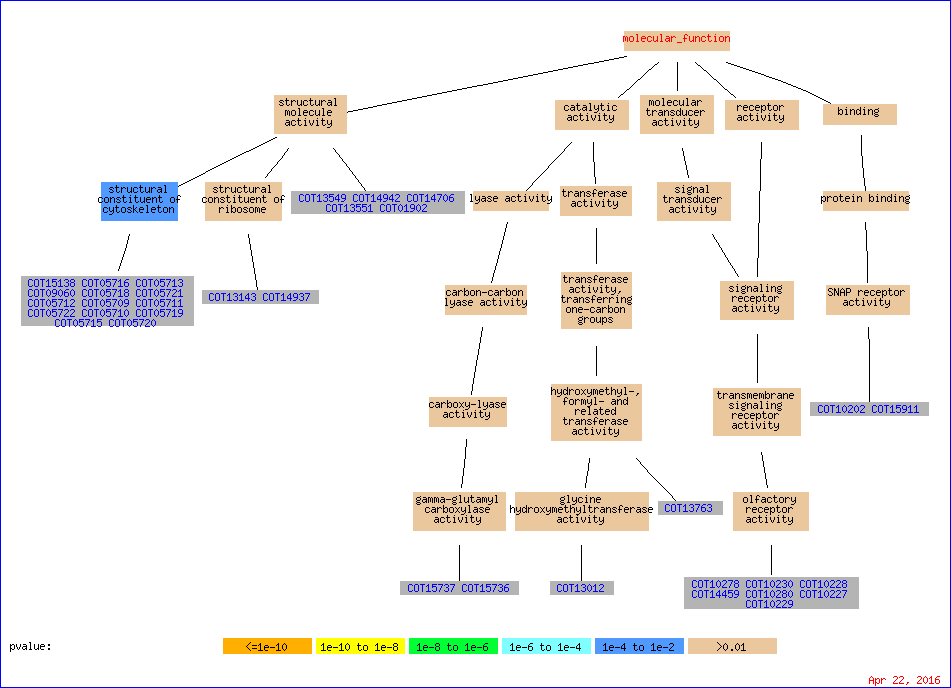
**

**Figure S9.** GO enrichment results (F) for genes located in the 1-kb regions flanking breakpoints of 131 large inversions in quail (*Coturnix japonica*) genome relative to that of chicken (*Gallus gallus*).





**Figure S10. Cross-validation plot for ADMIXTURE**


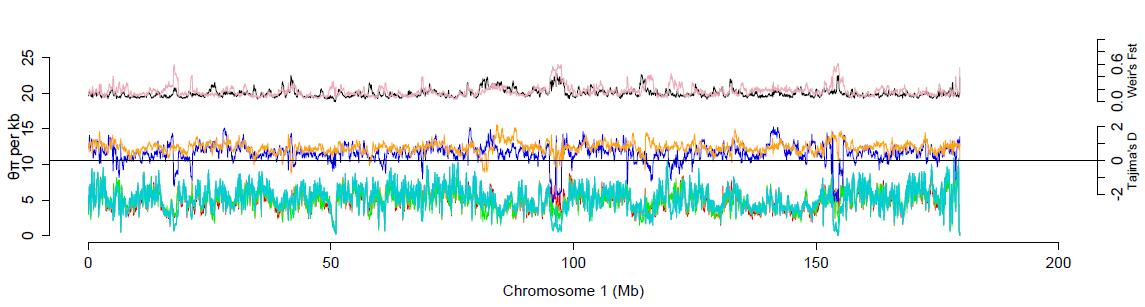


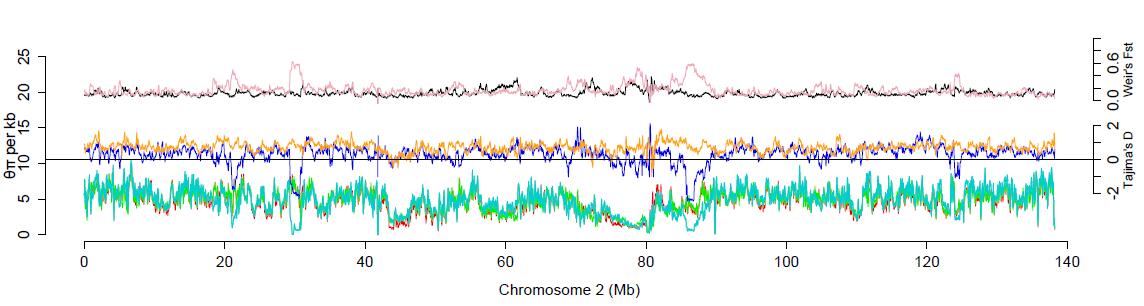


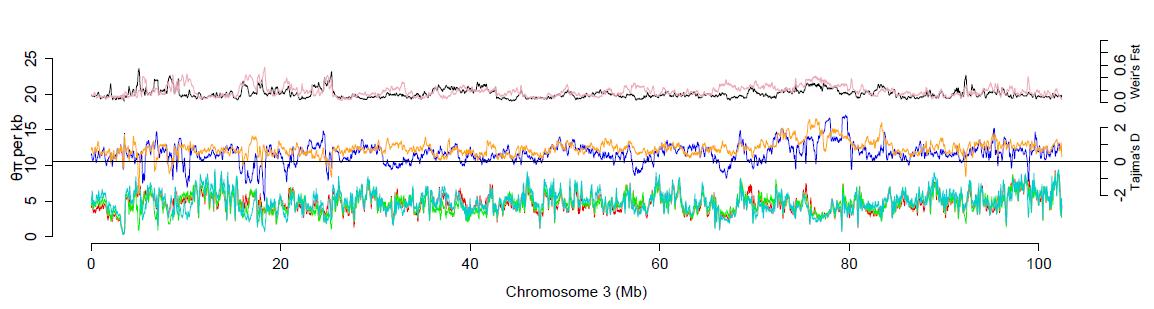

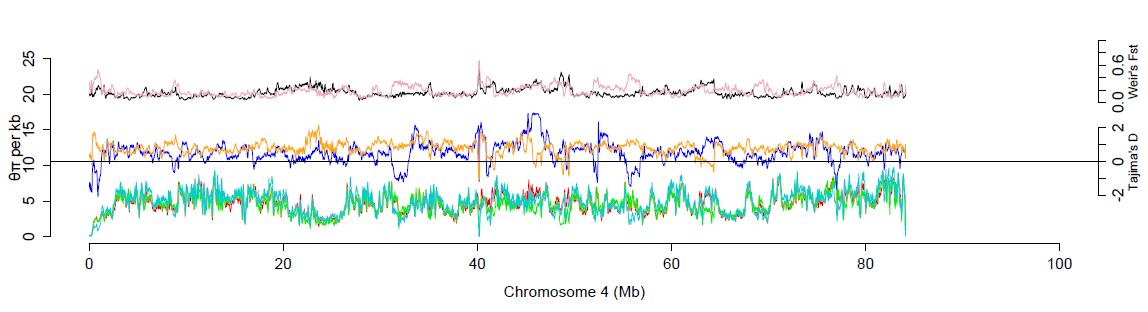


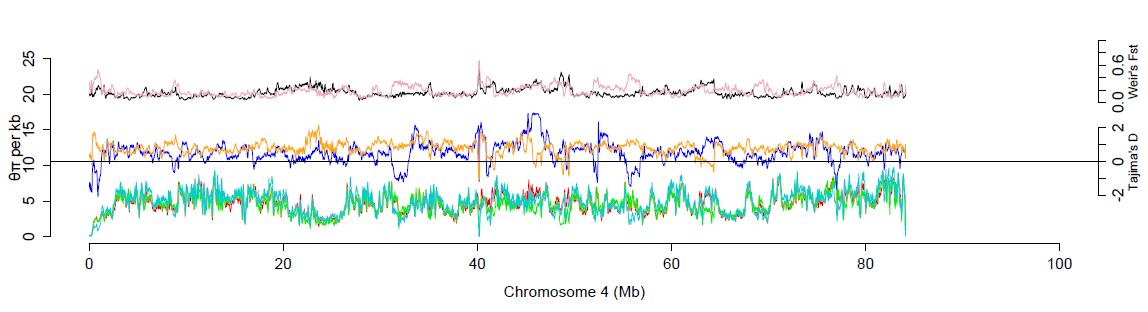


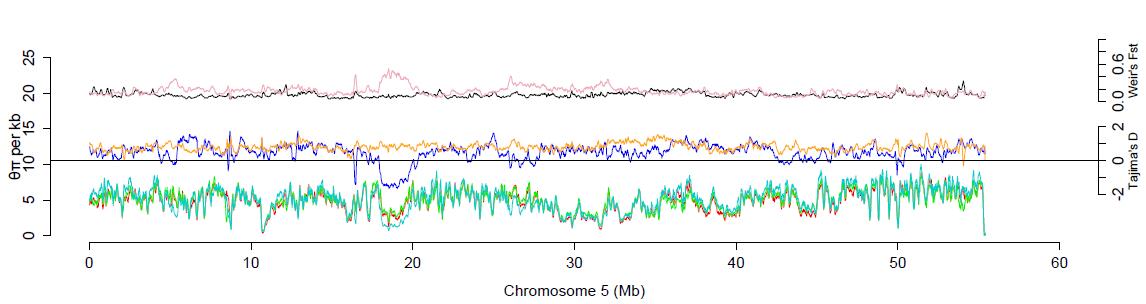


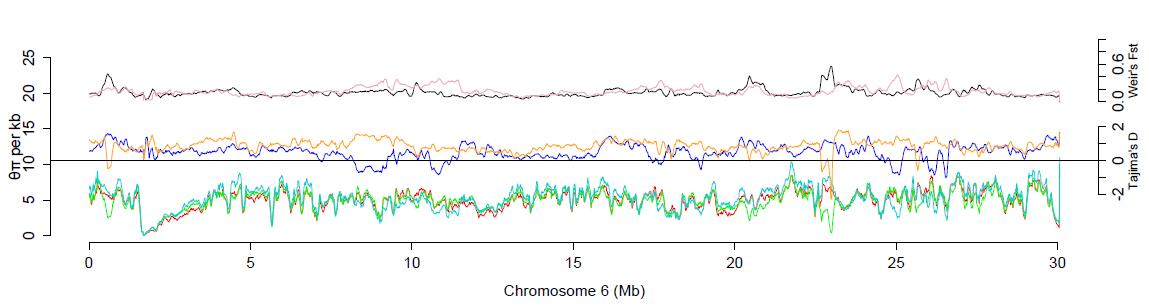


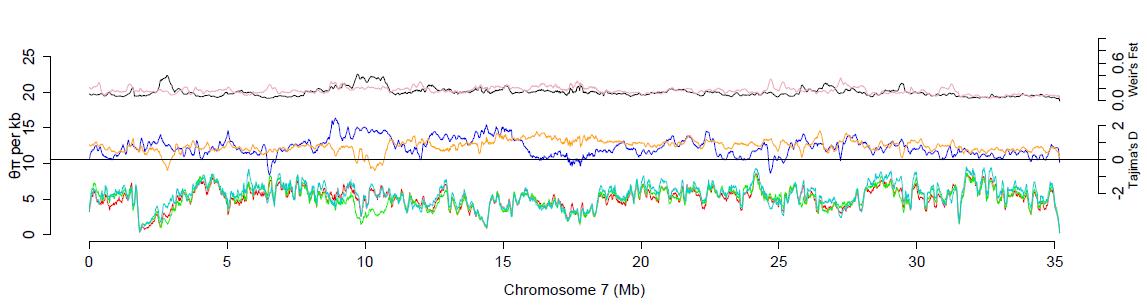


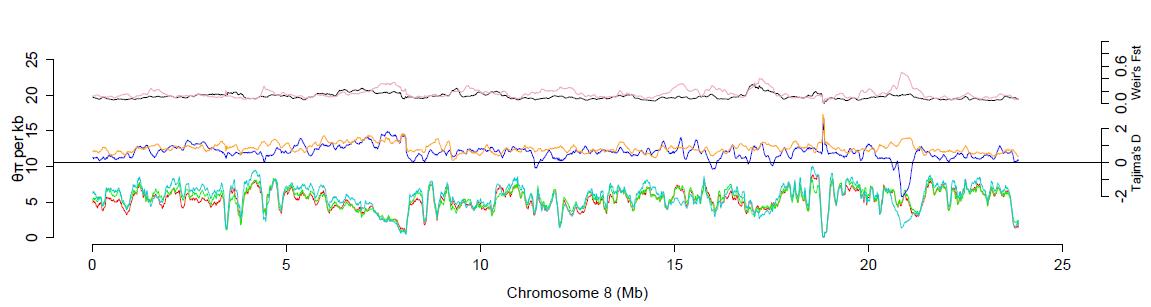


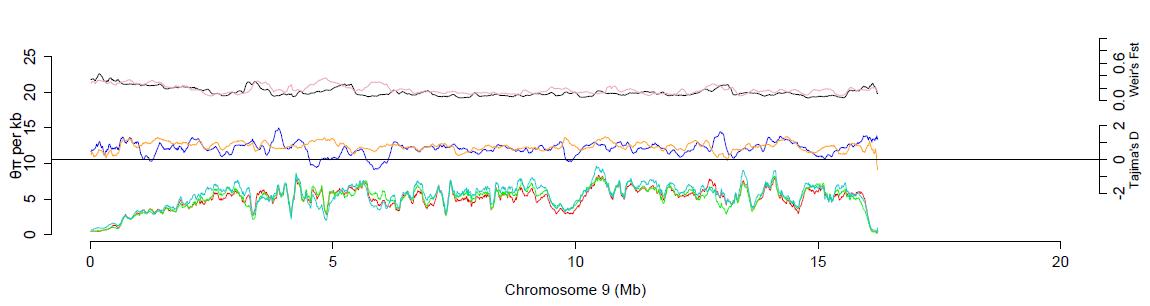


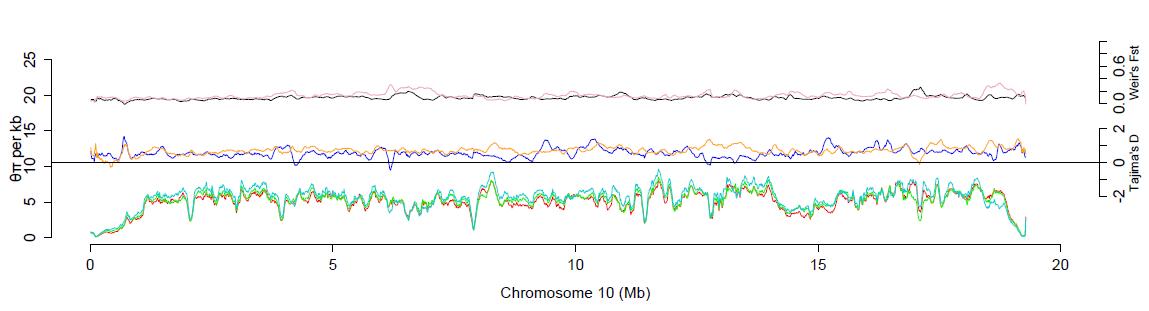


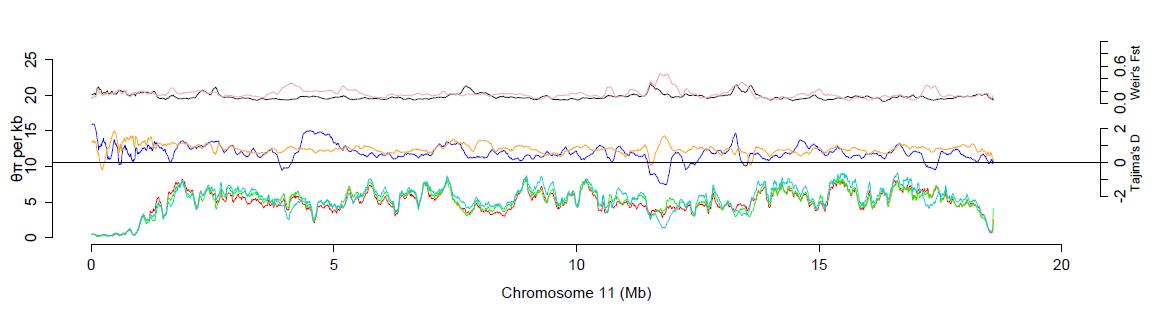


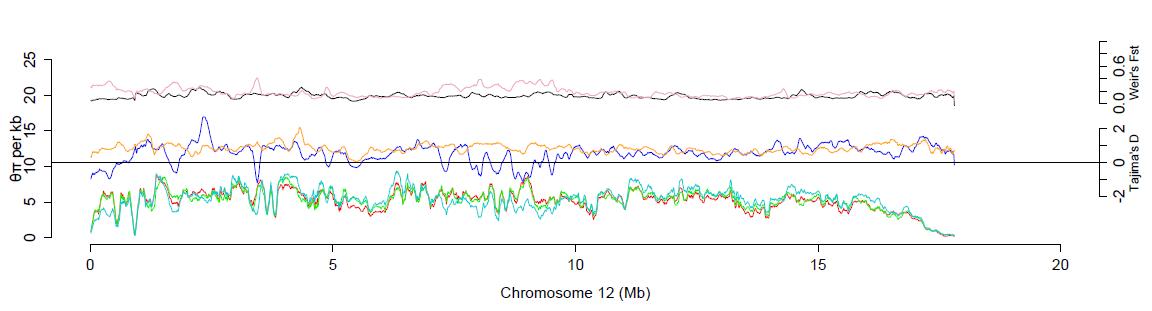


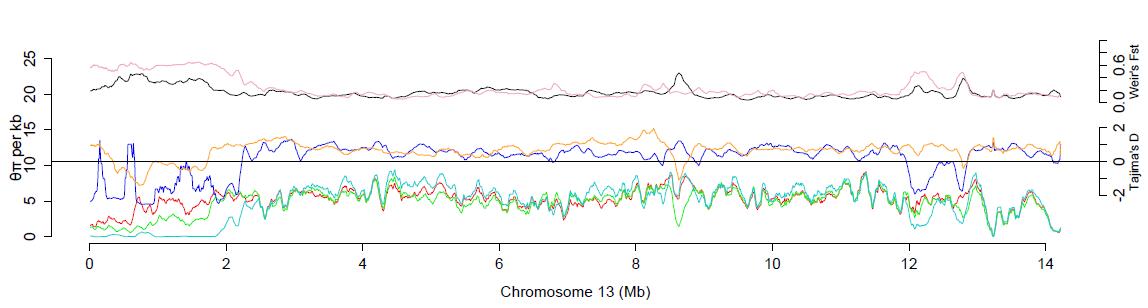


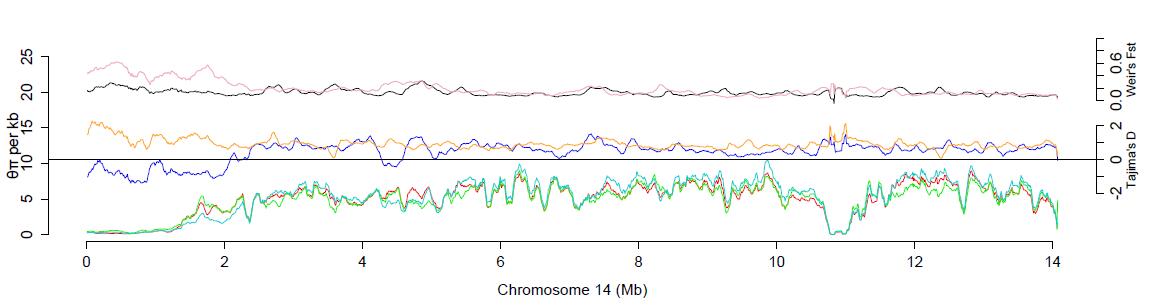


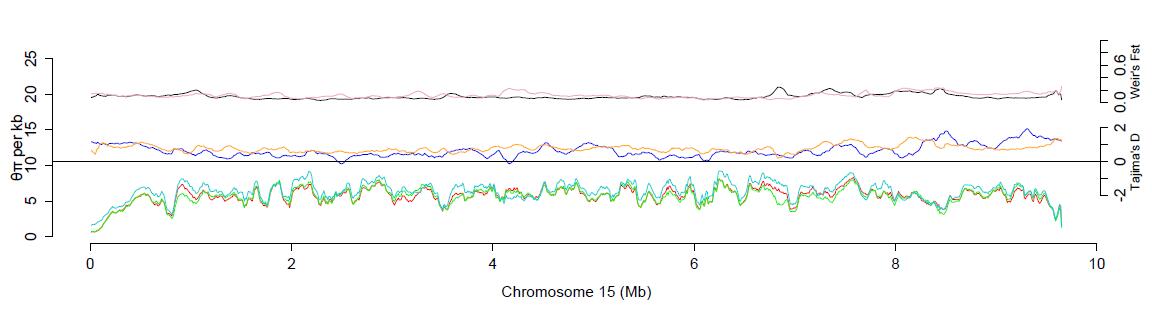


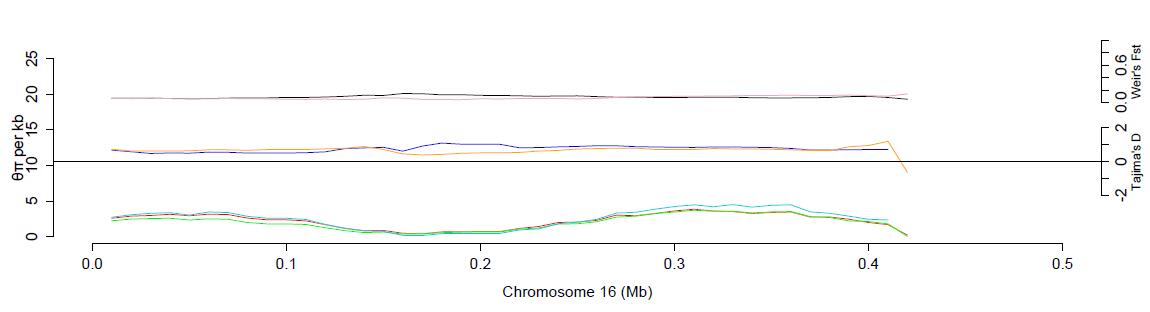


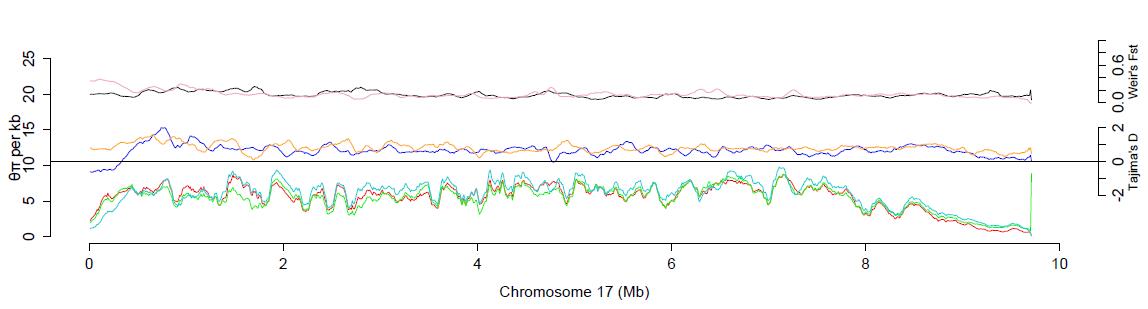


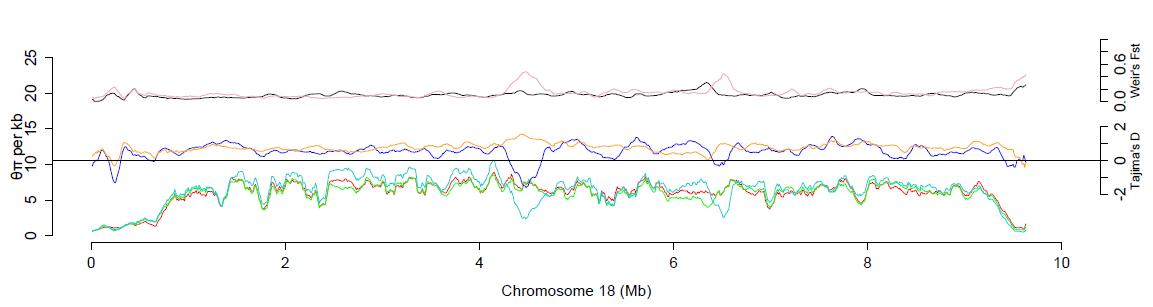


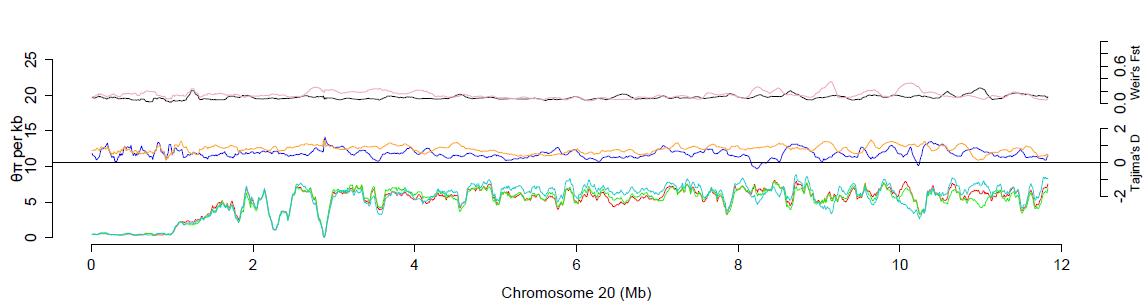


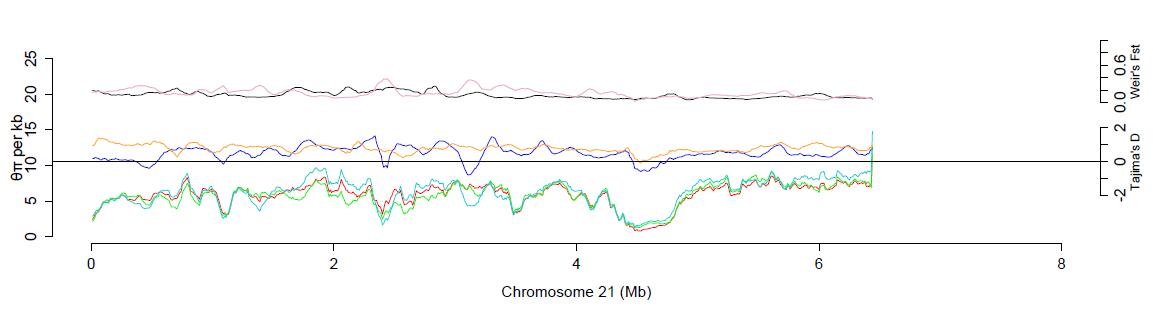


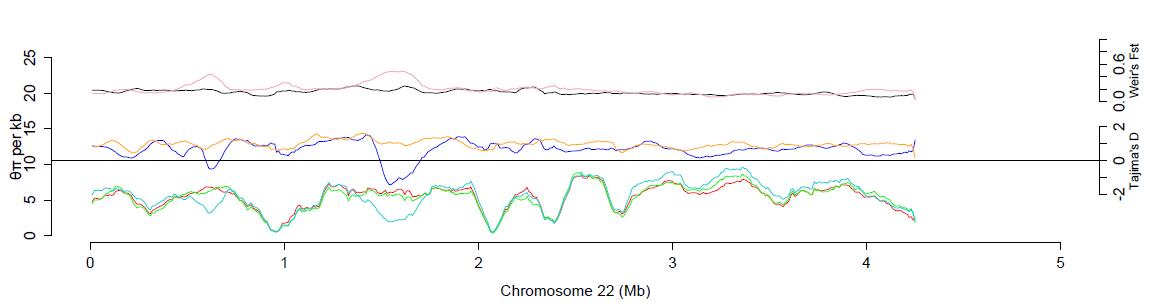


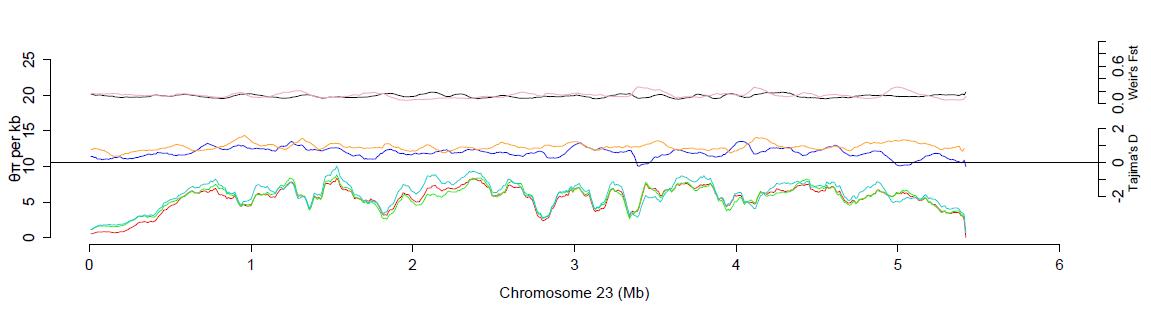


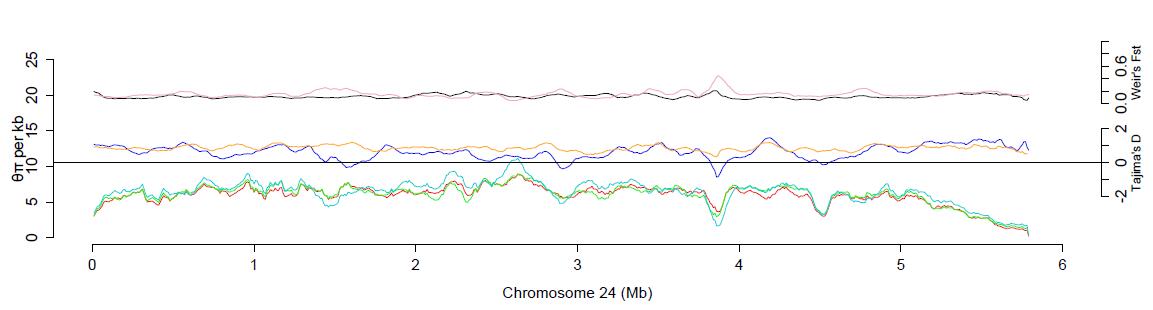


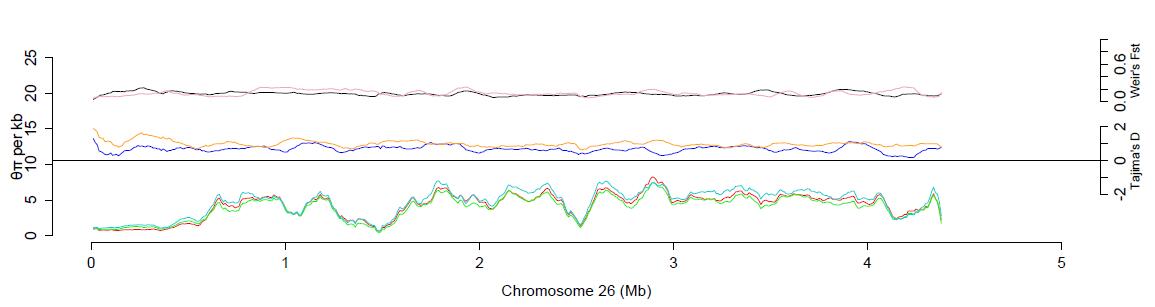


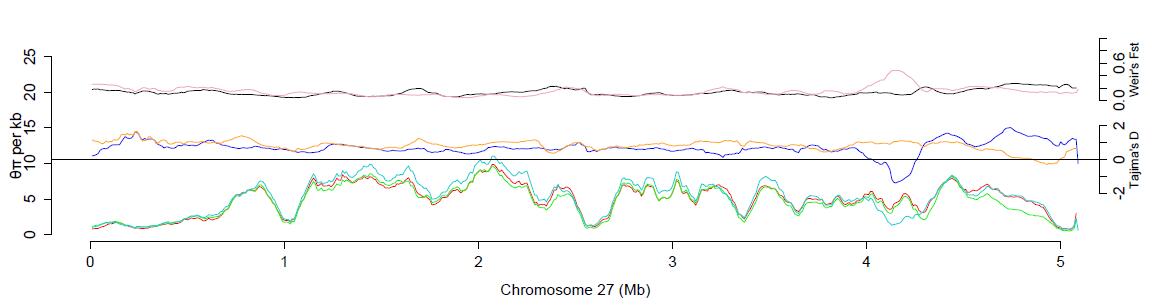


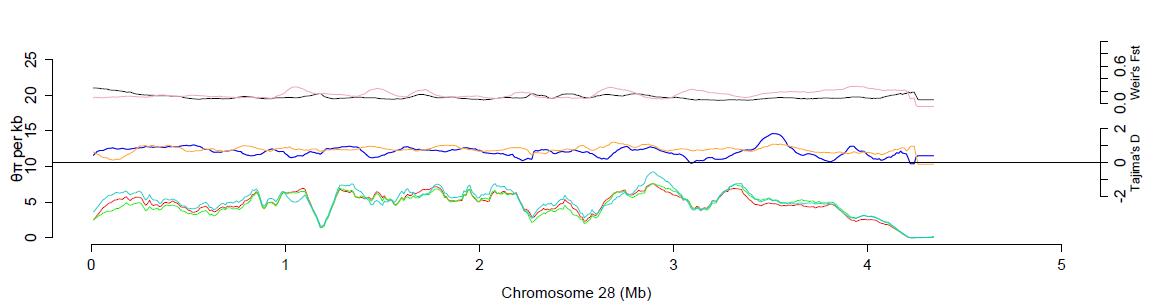


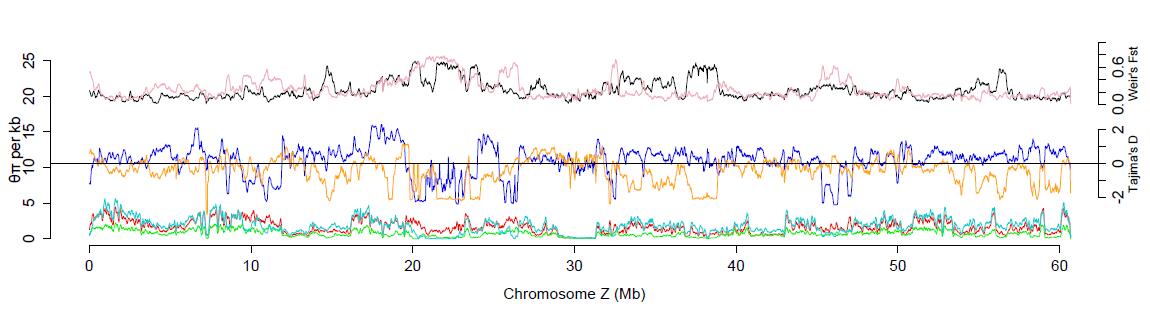


**Figure S11. Genome-wide nucleotide diversity, test of neutral evolutionary signals and population differentiation between wild and domesticated populations.** The red line, green line and cyan line indicate nucleotide diversity in wild, egg-type and meat-type quail, respectively. Tajima’s *D* values for the egg-type group (orange line) and meat-type group (blue line) reveal the selective signals on a genome-wide scale. *F*_st_ values (black line and pink line) that vary along each chromosome represent the level of population differentiation between wild and egg-type quail and between wild and meat-type quail, respectively. The chromosome 25 is not shown here due to bad SNPs which were filtered out during quality control procedure.


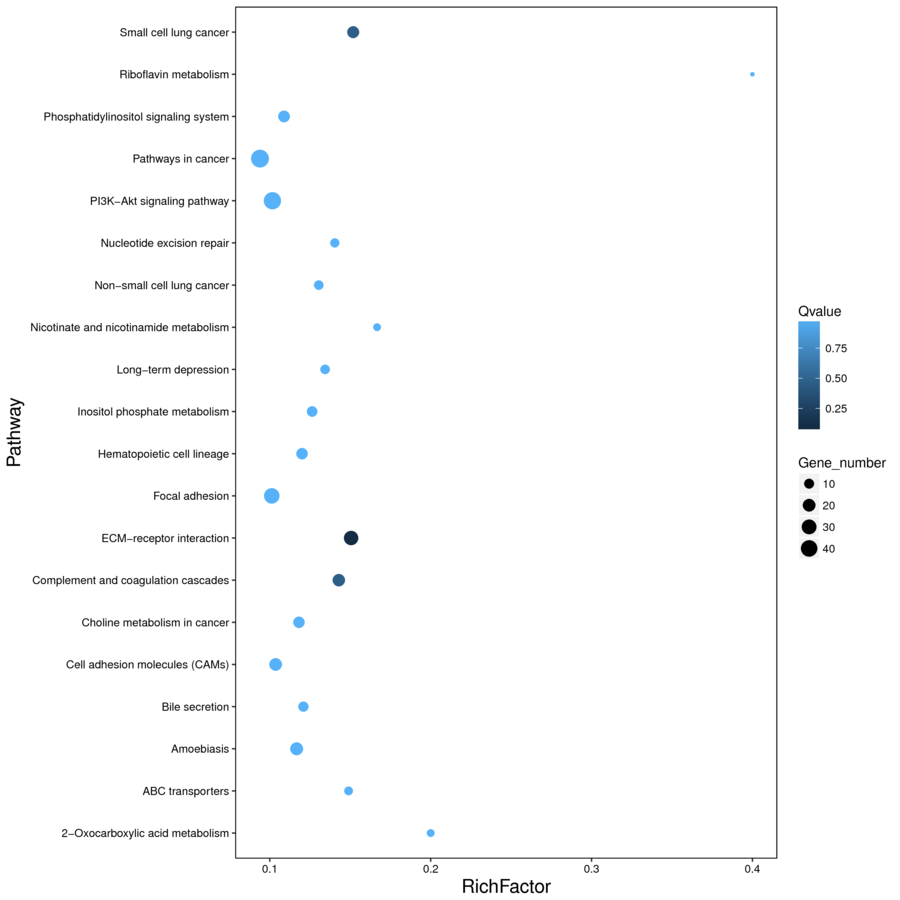


**Figure S12. Functional enrichment for divergent genes between egg-type and wild quails**


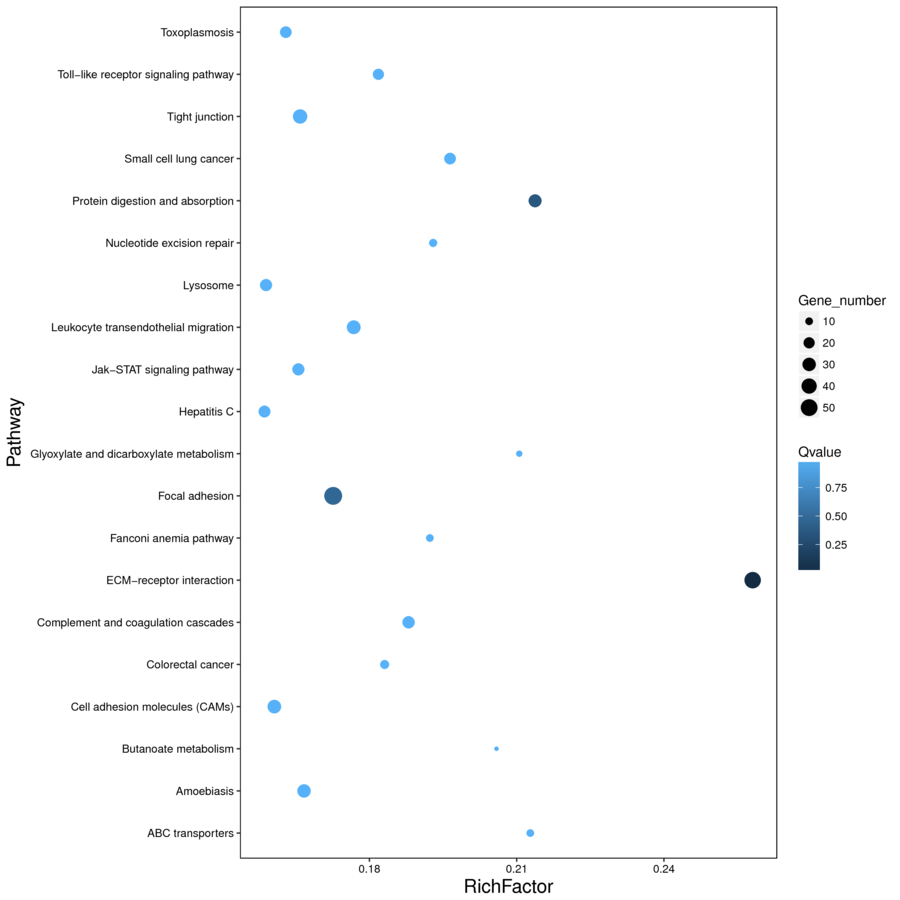


**Figure S13. Functional enrichment for divergent genes between meat-type and wild quails**

**
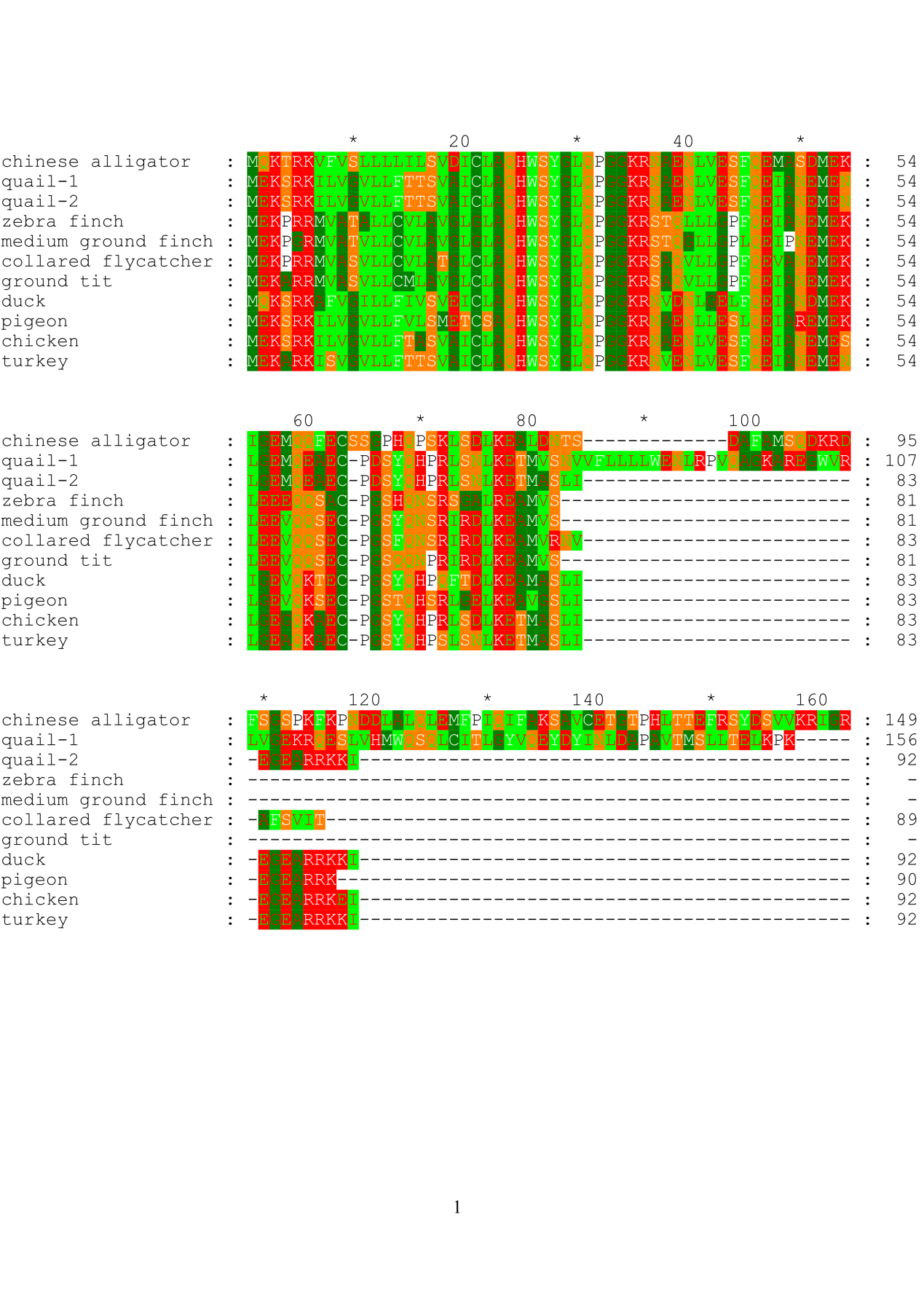
**

**Figure S14. *GNRH1* protein sequence alignment among nine avian species and the alligator.** In the figure, quail-1 and quail-2 represent the two copies of the *GNRH1*gene in quail that are not identical, while other analyzed species each possess only one copy. Predicted protein sequences are from *Coturnix japonica* (quail), *Gallus gallus* (chicken), *Anas platyrhynchos* (duck), *Columba livia* (pigeon), *Ficedula albicollis* (collared flycatcher), *Geospiza fortis* (medium ground finch), *Meleagris gallopavo* (turkey), *Pseudopodoces humilis* (ground tit), *Taeniopygia guttata* (zebra finch), with *Alligator sinensis* (Chinese alligator) as an outgroup.

**
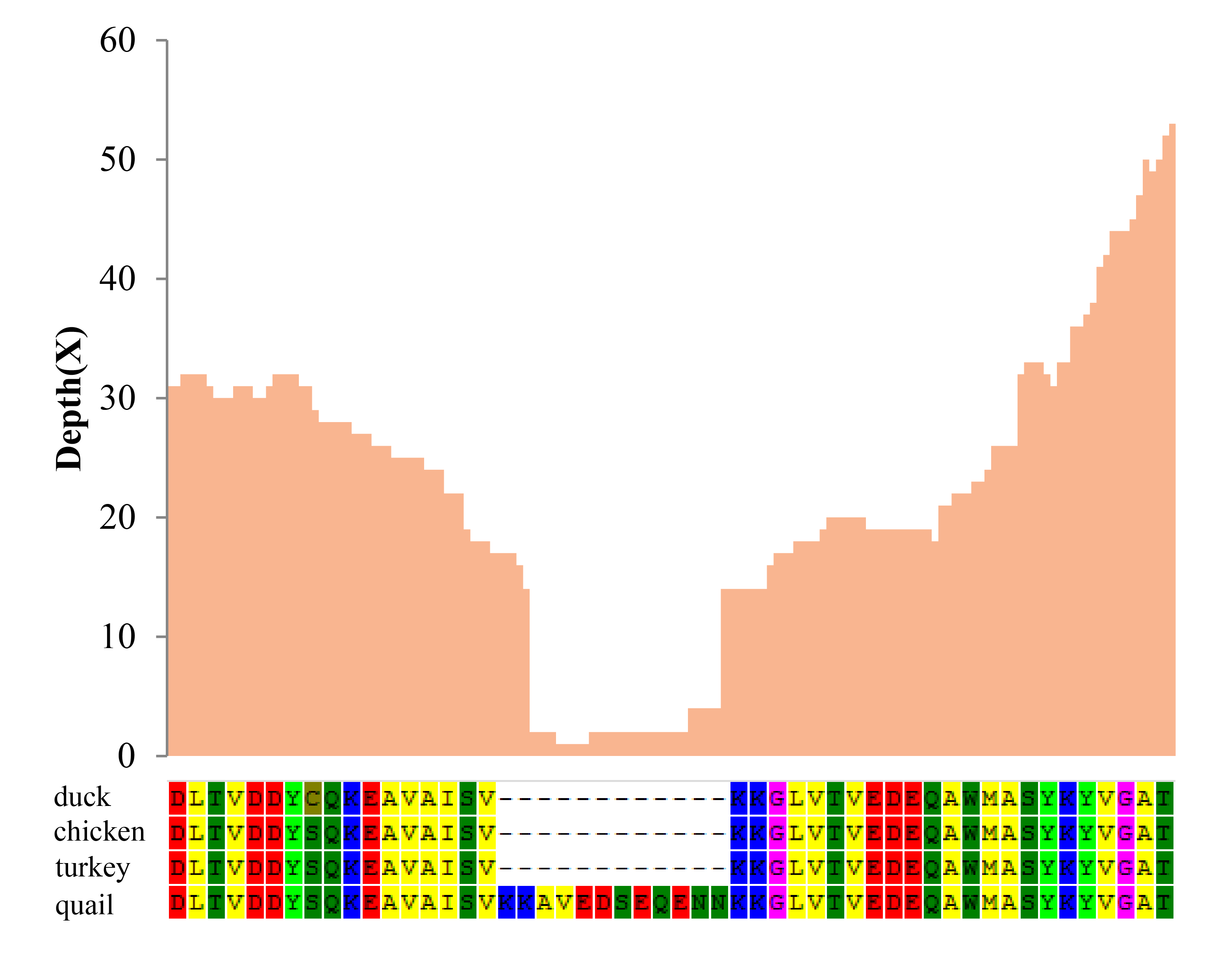
**

**Figure S15. Quail *PLCB4* protein sequence from amino acid residues 520 to 570.** The orange histogram bars indicate the depth of corresponding coding sequence mapped using clean RNA-Seq reads.

**
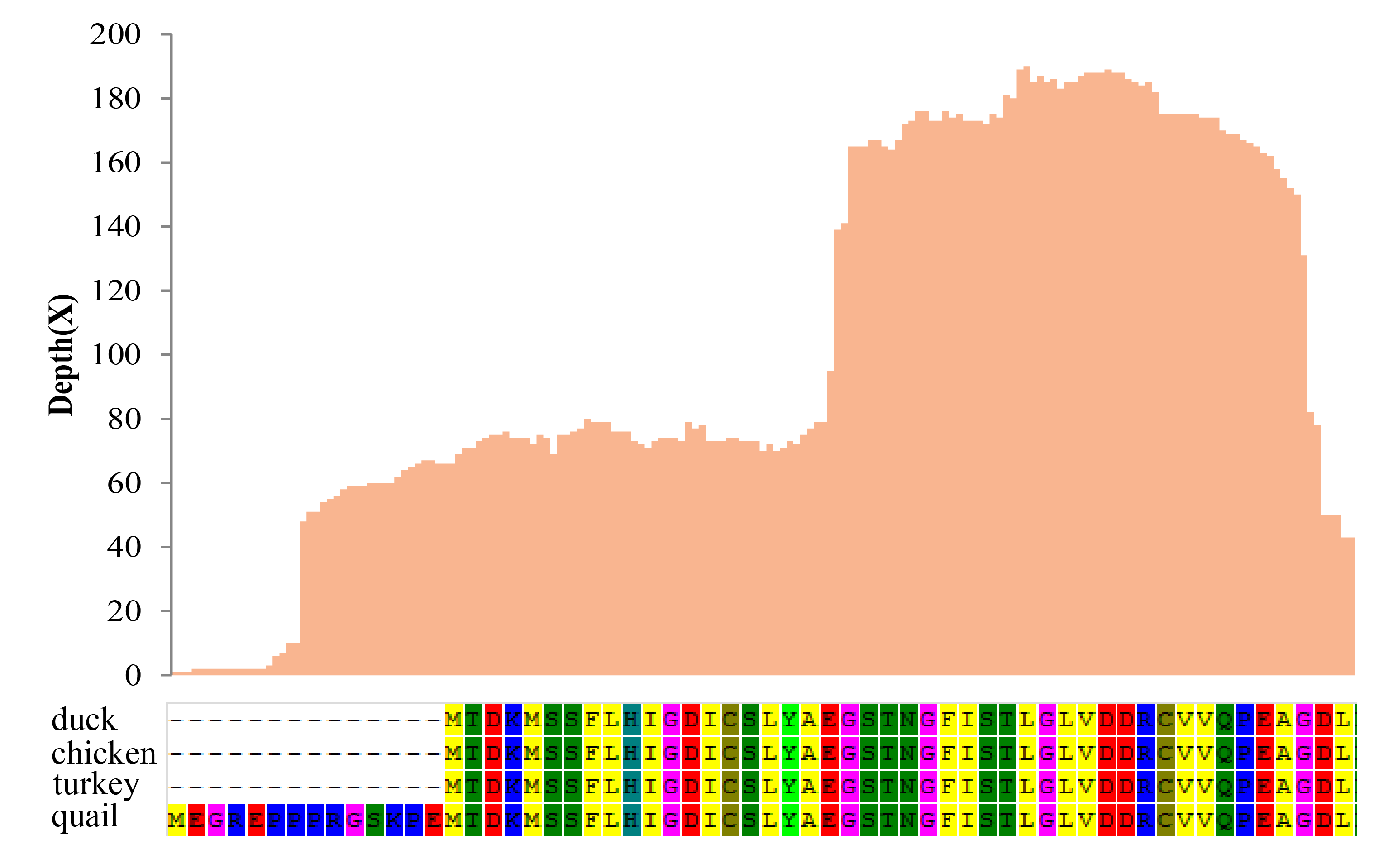
**

**Figure S16. Quail *ITPR1* protein sequence from amino acid residues 1 to 60.** The orange histogram bars indicate the depth of corresponding coding sequence mapped using clean RNA-Seq reads.

**
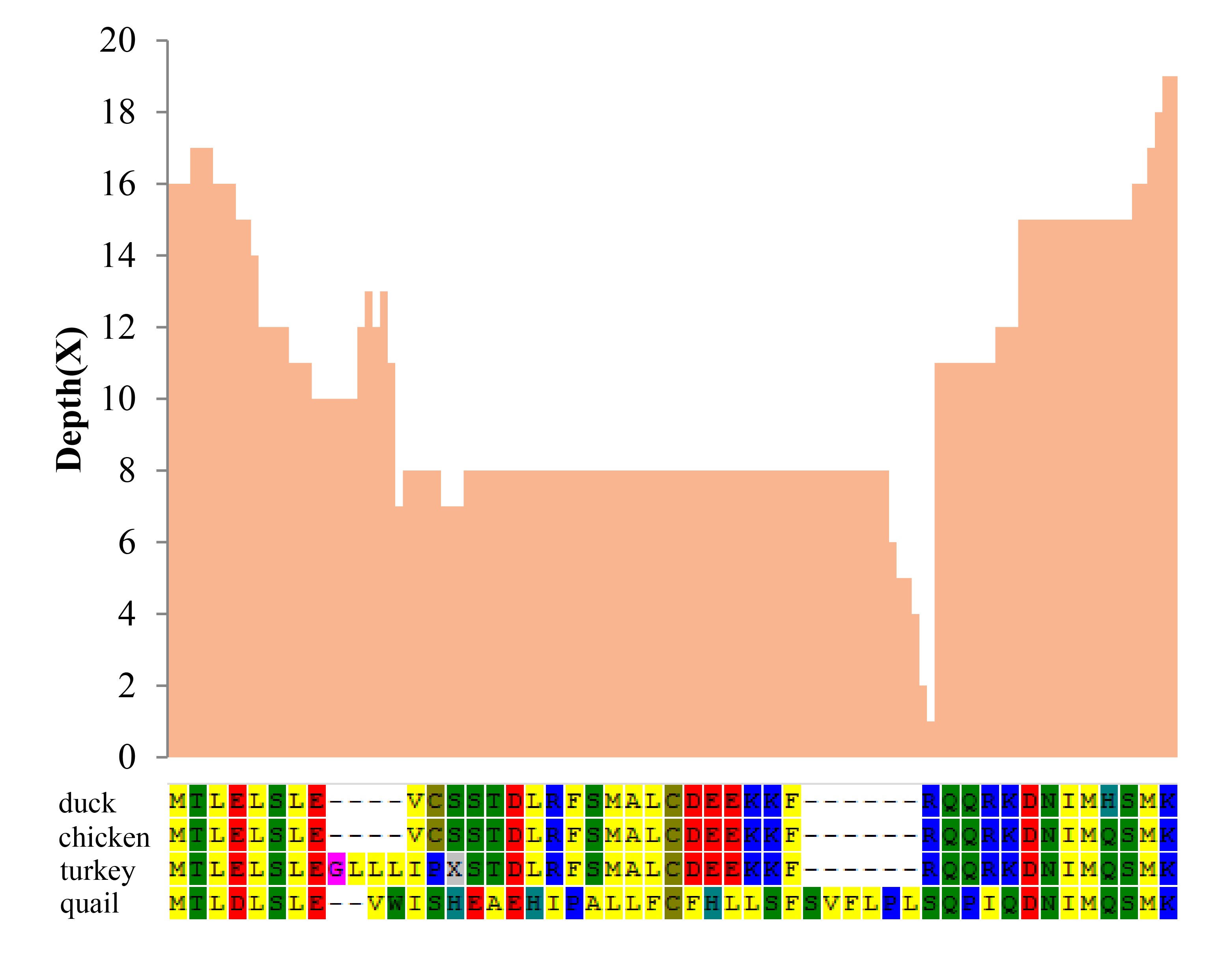
**

**Figure S17. Quail *PLA2G4* protein sequence from amino acid residues 130 to 178.** The orange histogram bars indicate the depth of corresponding coding sequence mapped using clean RNA-Seq reads.

**
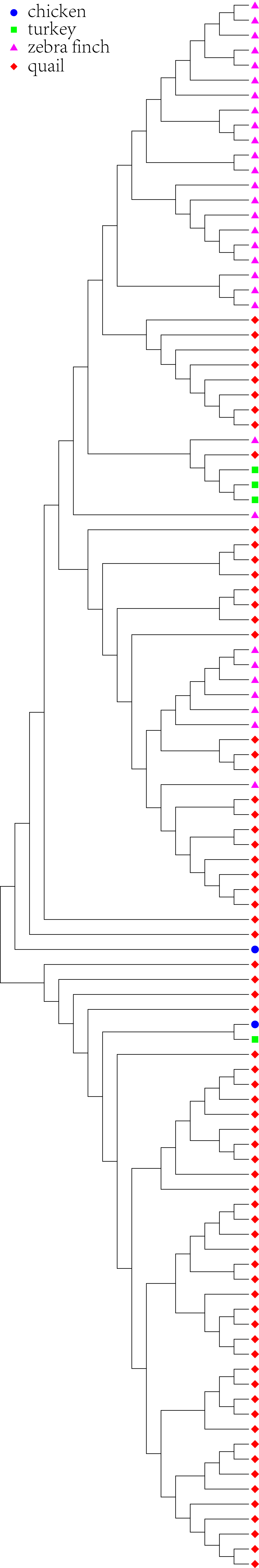
**

**Figure S18.** Phylogenetic tree of genes encoding a putative ‘Reverse transcriptase or Reverse transcriptase domain’ in *Coturnix japonica* (quail), *Gallus gallus* (chicken), *Meleagris gallopavo* (turkey) or *Taeniopygia guttata* (zebra finch).


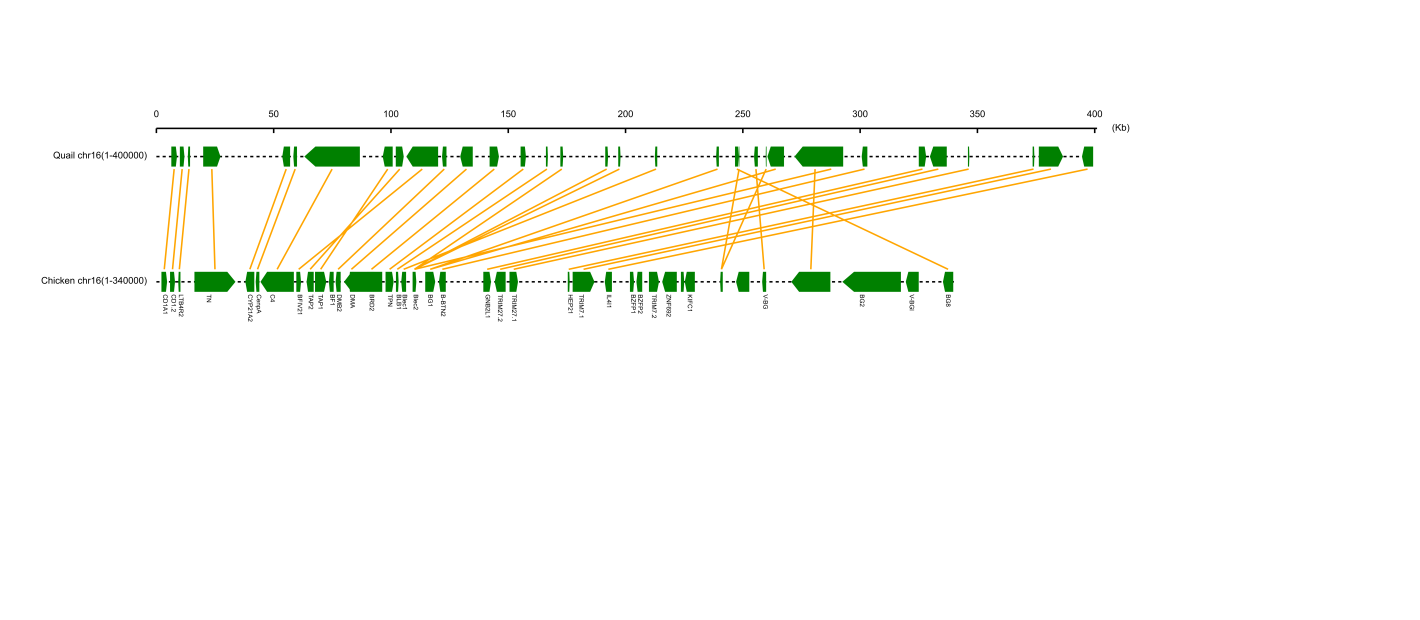


**Figure S19. Comparison of the *MHC-B* region on chromosome 16 between the genome of quail and chicken**. The green blocks represent genes on chromosome 16 and the yellow lines connect corresponding gene pairs in *Coturnix japonica* and *Gallus gallus*.


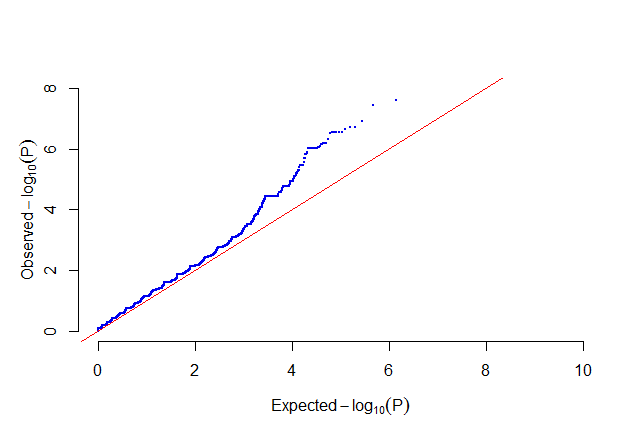


**Figure S20.** QQ plot of GWAS for plumage color in quail. The Q-Q plot displays the observed association *P*-values for all SNPs on the *y*-axis against the expected uniform distribution of *P*-values under the null hypothesis of no association on the *x*-axis. The SNP deviating from the diagonal at the upper-right end of the plot indicates that these SNPs are strongly associated with plumage color.

**
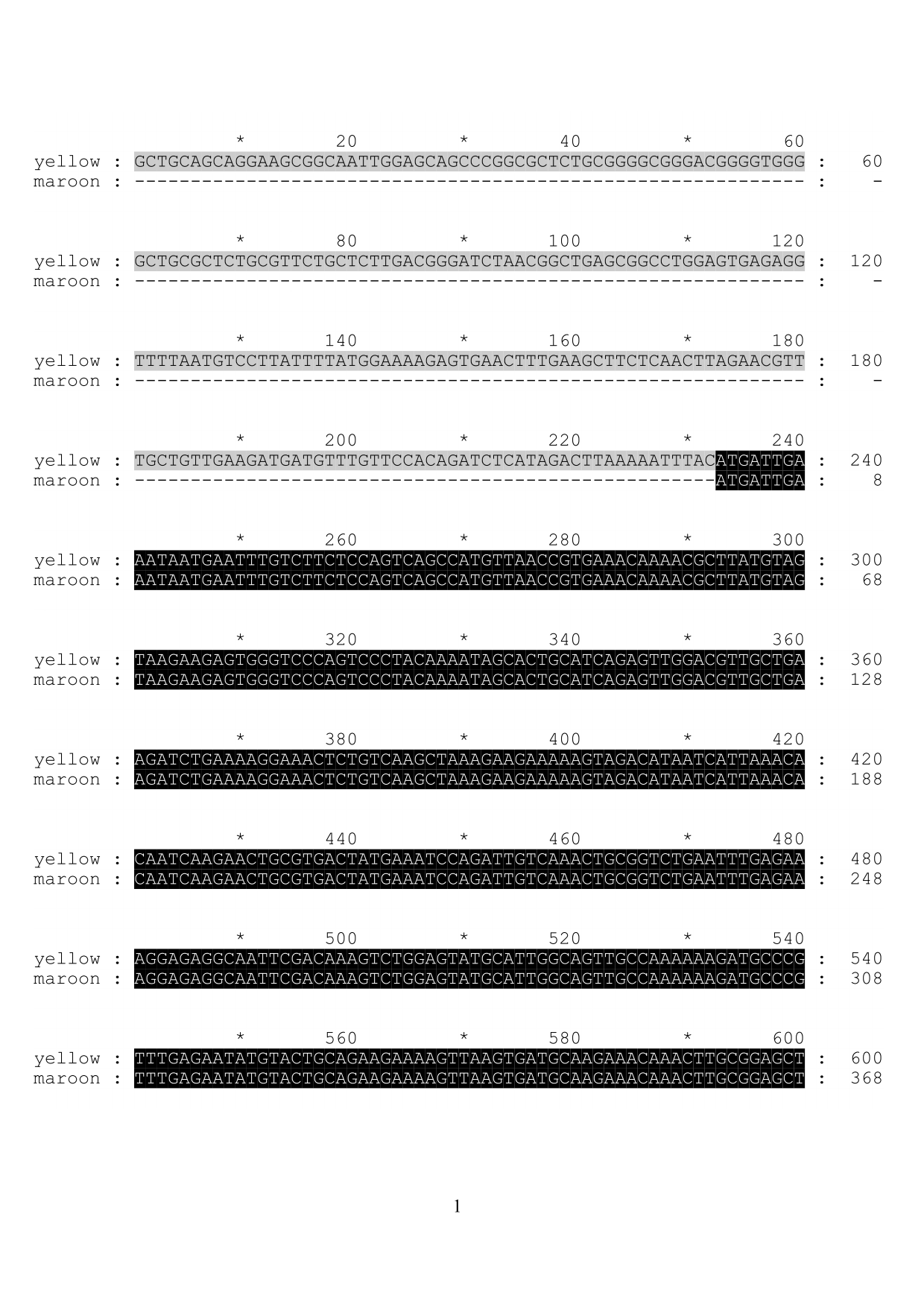

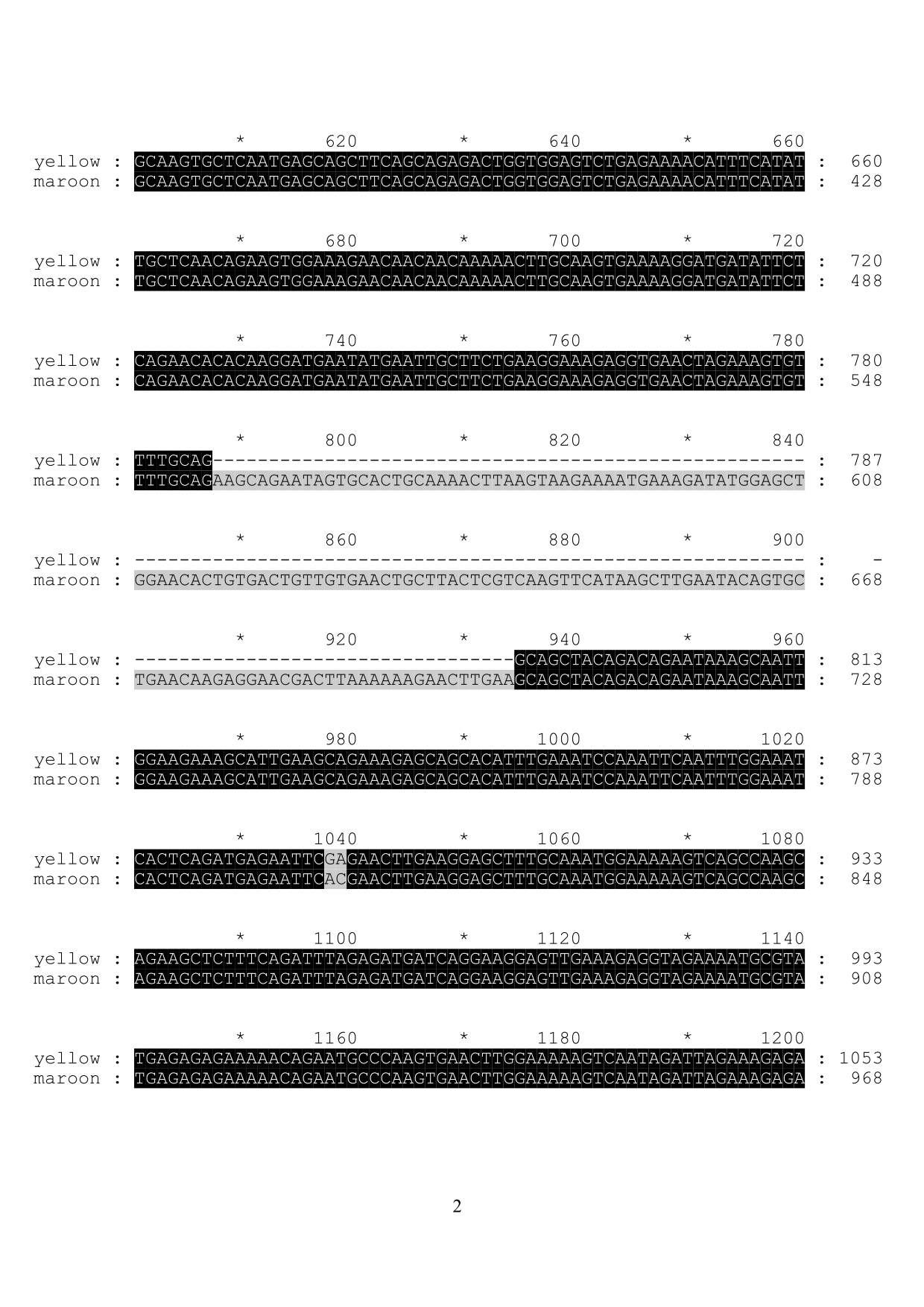

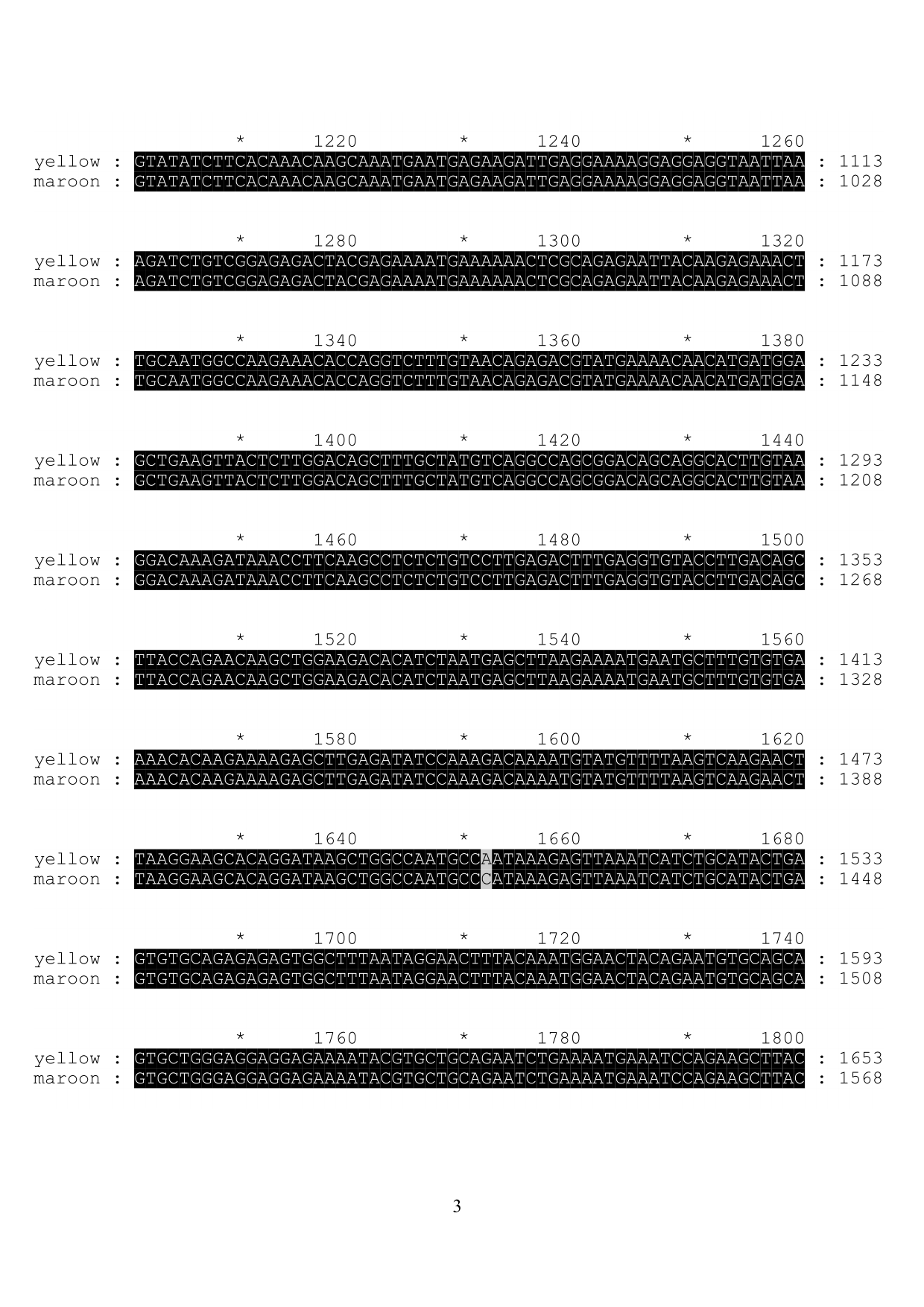

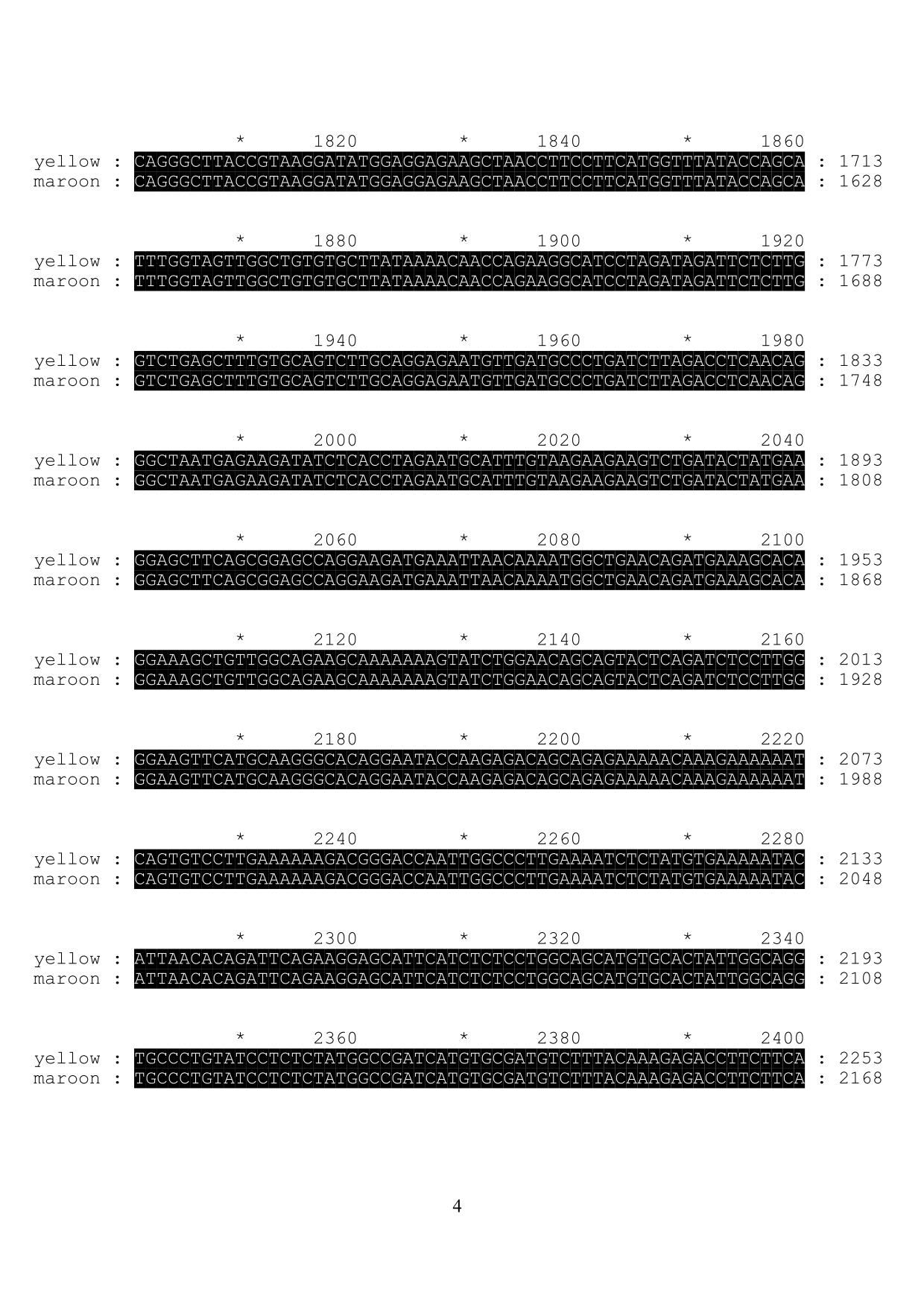

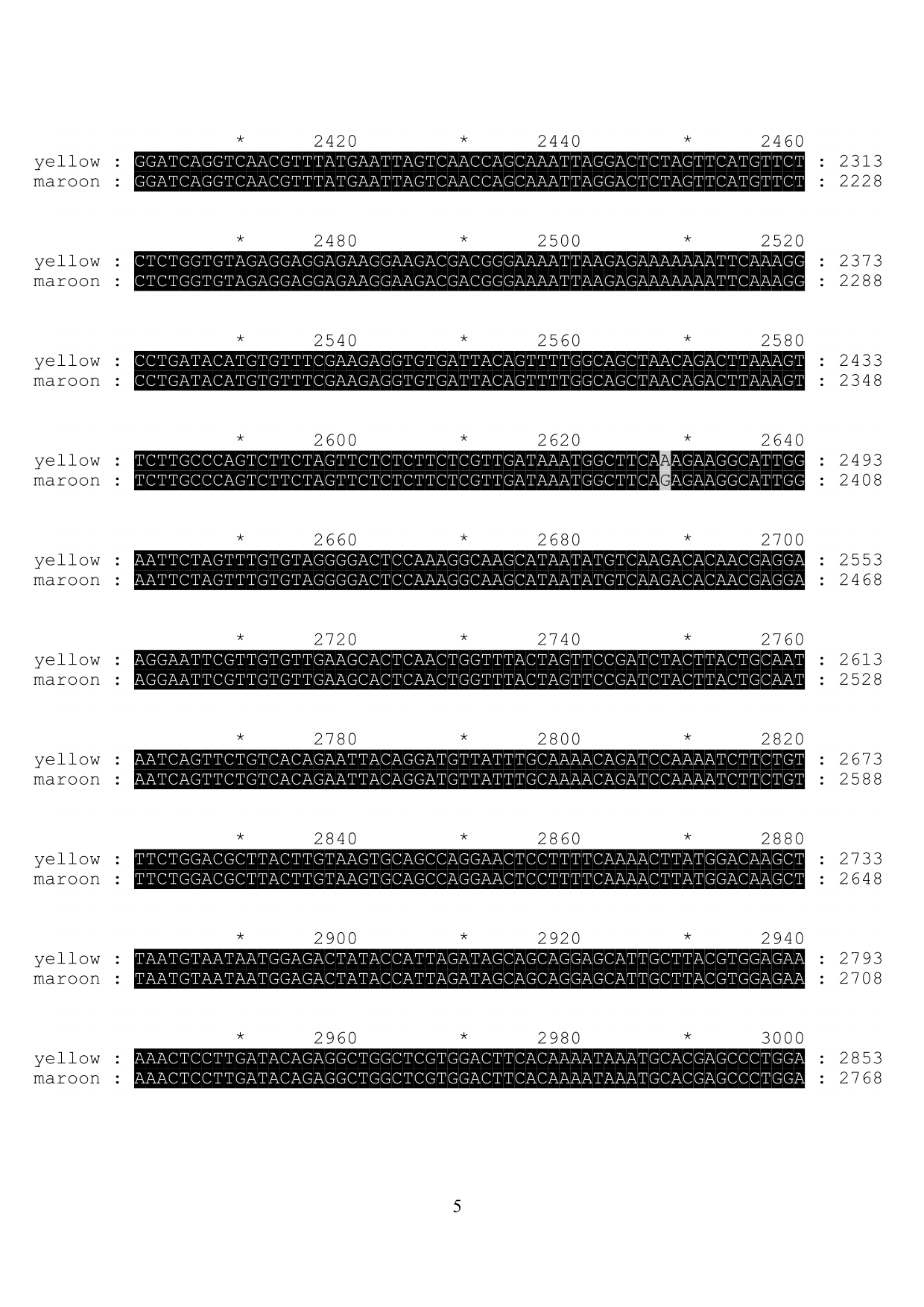

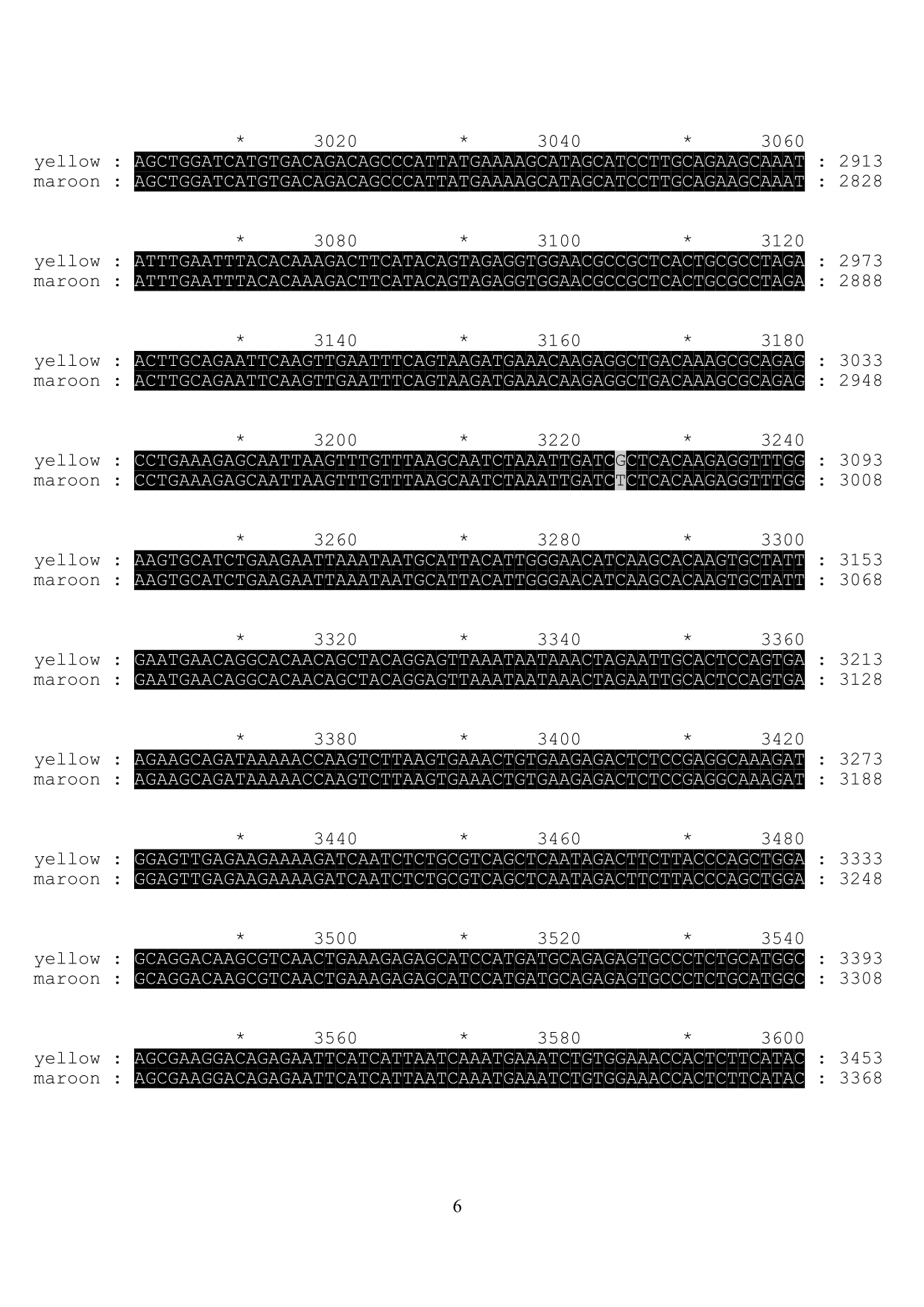

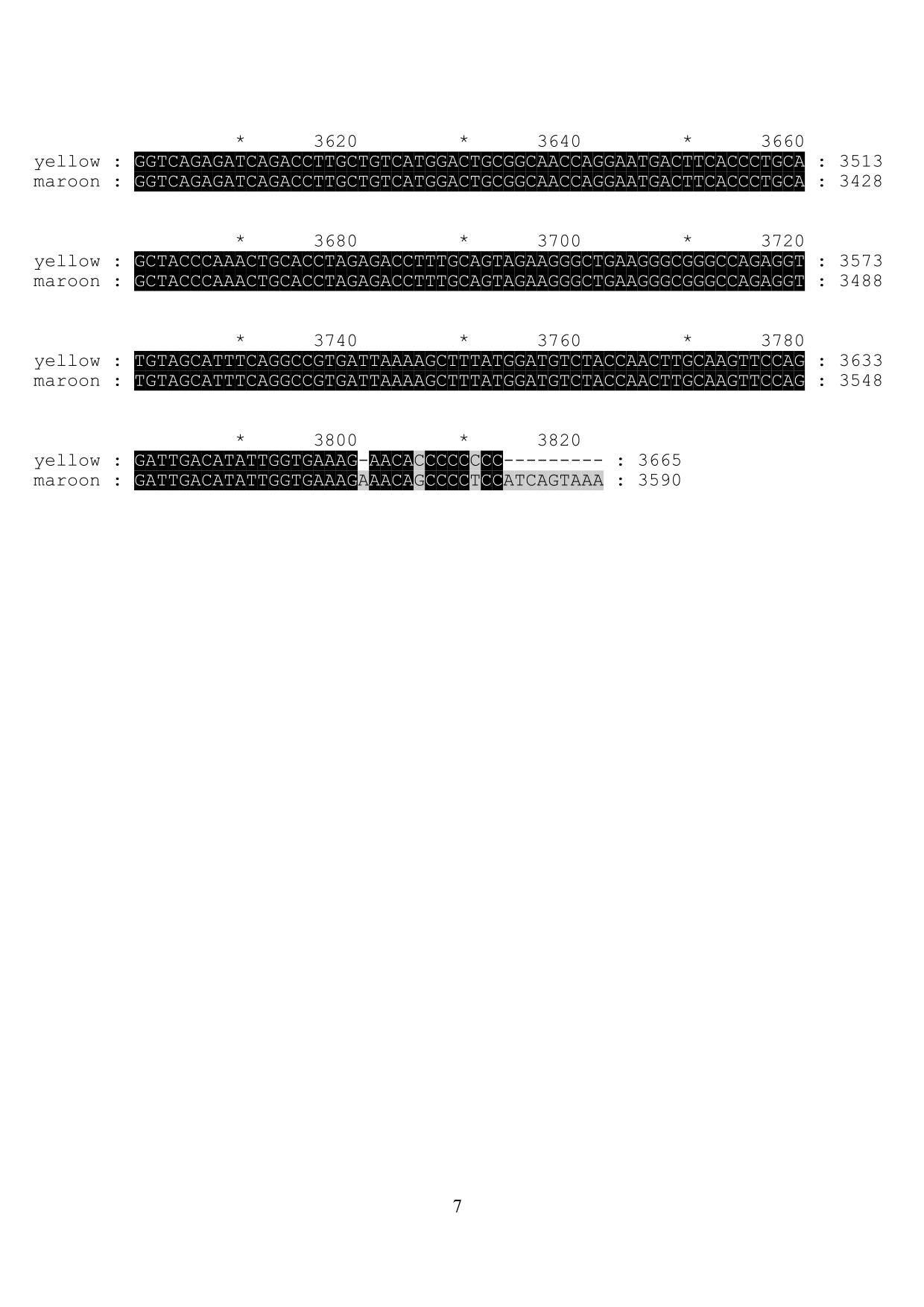
**

**Figure S21.** The sequencing result of *CCDC171* gene transcripts for yellow and maroon quail

**Supplementary part 2: Tables**

**Table S1. Characteristics of sequencing libraries and data**

| **Insert size** | **Read Lengths (bp)** | **Raw Data** | | **Filtered data** | |
| --- | --- | --- | --- | --- | --- |
|  |  | **Total Data (Gb)** | **Depth (X)** | **Total Data (Gb)** | **Depth (X)** |
| 170 bp | 100 | 77.258 | 70.234 | 70.442 | 64.038 |
| 500 bp | 100 | 69.133 | 62.848 | 56.107 | 51.006 |
| 800 bp | 100 | 16.311 | 14.829 | 12.661 | 11.51 |
| 2 kb | 49 | 29.524 | 26.84 | 23.119 | 21.017 |
| 5 kb | 49 | 26.507 | 24.097 | 16.987 | 15.442 |
| 10 kb | 49 | 17.824 | 16.203 | 12.368 | 11.244 |
| 20 kb | 49 | 13.036 | 11.851 | 4.409 | 4.008 |
| 40 kb | 49 | 12.498 | 11.361 | 3.355 | 3.05 |
| Total | - | 262.091 | 238.263 | 199.448 | 181.315 |

**Table S2. Summary statistics for the quail genome assembly**

| **Features** | **Contig** | | **Scaffold** | |
| --- | --- | --- | --- | --- |
|  | **Size (bp)** | **Number** | **Size (bp)** | **Number** |
| **N90** | 4,178 | 41,756 | 116,205 | 780 |
| **N80** | 10,857 | 27,726 | 587,803 | 449 |
| **N70** | 16,436 | 20,202 | 963,031 | 312 |
| **N60** | 22,004 | 14,884 | 1,379,345 | 222 |
| **N50** | 27,934 | 10,786 | 1,820,733 | 156 |
| **Longest** | 300,852 | - | 15,141,834 | - |
| **Total Size** | 1,013,840,816 | - | 1,039,933,626 | - |
| **Total Number (≥100 bp)** | - | 357,783 | - | 302,999 |
| **Total Number (≥2 kb)** | - | 49,795 | - | 3,556 |

**Table S3. Statistics for alignment of fosmid clone sequences to the quail genome assembly**

| **Fosmid ID** | **Length (bp)** | **Coverage Ratio (%)** | **Alignment Blocks** | **Aligned Scaffold** | **Aligned Scaffold Length (bp)** | **Gap** | **Gap Length (bp)** |
| --- | --- | --- | --- | --- | --- | --- | --- |
| **wswaxa** | 43,979 | 99.65 | 6 | 1 | 5,109,890 | 3 | 763 |
| **wswbxa** | 40,565 | 98.45 | 9 | 1 | 4,270,311 | 5 | 743 |
| **wswdxa** | 39,157 | 98.81 | 6 | 1 | 4,854,659 | 3 | 393 |
| **wswgxa** | 37,880 | 92.22 | 5 | 1 | 5,081,132 | 1 | 37 |
| **wswhxa** | 36,554 | 99.27 | 5 | 1 | 3,813,628 | 1 | 68 |
| **wswixa** | 40,970 | 99.67 | 4 | 1 | 9,678,044 | 2 | 80 |
| **wswjxa** | 35,061 | 99.95 | 5 | 1 | 2,003,839 | 1 | 10 |

| **Dataset** | | **Number** | **Total length (bp)** | **Coverage by assembly (%)** | **with >90% sequence in one scaffold** | | **with >50% sequence in one scaffold** | |
| --- | --- | --- | --- | --- | --- | --- | --- | --- |
|  |  |  |  |  | **Number** | **Percentage** | **Number** | **Percentage** |
| **BL** | >200 bp | 63,363 | 49,513,508 | 93.57 | 57,165 | 90.21 | 60,052 | 94.77 |
|  | >500 bp | 26,615 | 38,404,514 | 93.34 | 23,555 | 88.50 | 24,926 | 93.65 |
|  | >1 kb | 13,921 | 29,512,512 | 93.55 | 12,256 | 88.03 | 13,048 | 93.72 |
| **L** | >200 bp | 44,250 | 19,639,492 | 93.65 | 40,173 | 90.78 | 42,214 | 95.39 |
|  | >500 bp | 10,185 | 9,595,297 | 92.63 | 9,041 | 88.76 | 9,519 | 93.46 |
|  | >1 kb | 2,882 | 4,648,094 | 92.60 | 2,518 | 87.36 | 2,684 | 93.12 |
| **LP** | >200 bp | 58,660 | 47,960,237 | 93.55 | 52,641 | 89.73 | 55,961 | 95.39 |
|  | >500 bp | 25,779 | 37,859,639 | 93.46 | 22,797 | 88.43 | 24,138 | 93.63 |
|  | >1 kb | 13,513 | 29,270,555 | 93.79 | 11,935 | 88.32 | 12,694 | 93.93 |

**Table S4. Evaluation of the quail genome assembly using *de novo* assmbly of RNA-Seq reads**

**Table S5. Evaluation of the quail genome assembly by using BUSCO**

| **Description** | **Gene number** | **Percent (%)** |
| --- | --- | --- |
| **Complete BUSCOs (C)** | **2491** | **96.33** |
| **Complete and single-copy BUSCOs (S)** | **2465** | **95.32** |
| **Complete and duplicated BUSCOs (D)** | **26** | **1.01** |
| **Fragmented BUSCOs (F)** | **60** | **2.32** |
| **Missing BUSCOs (M)** | **35** | **1.35** |
| **Total BUSCO groups searched** | **2586** | **100.00** |

**Table S6. Statistics of transposable element(TE) content in the quail genome**

| **Type** | Repbase TEs | | Predicted TE proteins | | *De novo* predicted TEs | | Combined TEs | |
| --- | --- | --- | --- | --- | --- | --- | --- | --- |
|  | Length (bp) | (%) | Length (bp) | (%) | Length (bp) | (%) | Length (bp) | (%) |
| **DNA** | 11182662 | 1.07 | 2,745,517 | 0.26 | 9,058,540 | 0.87 | 15,168,264 | 1.46 |
| **LINE** | 60073991 | 5.77 | 36,747,298 | 3.53 | 85,671,803 | 8.24 | 97,958,163 | 9.42 |
| **SINE** | 543290 | 0.05 | 0 | 0 | 324,176 | 0.03 | 857,353 | 0.08 |
| **LTR** | 12561168 | 1.20 | 2,508,751 | 0.24 | 31,412,297 | 3.02 | 37,214,344 | 3.58 |
| **Other** | 10609 | 0.001 | 0 | 0 | 0 | 0.00 | 10,609 | 0.00 |
| **Unknown** | 482870 | 0.04 | 0 | 0 | 3,870,713 | 0.37 | 4,353,497 | 0.42 |
| **Total** | 83799678 | 8.06 | 41,988,136 | 4.03 | 117,596,126 | 11.31 | 128,731,999 | 12.38 |

**Table S7. Summary of gene annotations for the quail genome**

| **Prediction Method** | **Gene set** | | No. | Avg. transcript length (bp) | Avg. CDS length (bp) | Avg. no. exons per gene | | | Avg. exon length (bp) | Avg. intron length (bp) | |
| --- | --- | --- | --- | --- | --- | --- | --- | --- | --- | --- | --- |
| ***De novo*** | **AUGUSTUS** | 16,015 | | 20,141.10 | 1,543.79 | | 9.49 | 162.66 | | 2,190.32 |  |
|  | **GENSCAN** | 28,127 | | 24,305.93 | 1,426.42 | | 8.63 | 165.21 | | 2,997.08 |  |
| **Homology** | ***H. sapiens*** | 16,497 | | 16,493.44 | 1,423.18 | | 8.23 | 173.02 | | 2,085.74 |  |
|  | ***G. gallus*** | 18,740 | | 15,627.19 | 1,381.76 | | 8.18 | 168.94 | | 1,984.27 |  |
|  | ***M. gallopavo*** | 16,328 | | 18,358.50 | 1,398.49 | | 8.46 | 165.21 | | 2,272.00 |  |
|  | ***A. platyrhynchos*** | 16,062 | | 17,527.16 | 1,455.51 | | 8.93 | 163.04 | | 2,027.40 |  |
|  | ***T. guttata*** | 19,023 | | 13,944.23 | 1,211.23 | | 7.18 | 168.72 | | 2,060.67 |  |
|  | **GLEAN** | | 16,030 | 24,599.20 | 1,673.01 | 10.20 | | | 164.00 | 2,491.58 | |
|  | **RNA-Seq** | | 15,844 | 12,563.50 | 1,228.89 | 6.97 | | | 176.36 | 1,899.12 | |
|  | **Final gene set** | | 16,210 | 25,060.32 | 1,702.86 | 10.40 | | | 163.73 | 2,484.80 | |

Note: Avg. means average, CDS means coding sequence of a gene.

**Table S8. Summary of functional annotation of genes in the quail genome**

|  | **Number** | **Percent (%)** |
| --- | --- | --- |
| **Total** | 16,210 | 100 |
| **InterPro** | 14,456 | 89.18 |
| **GO** | 11,347 | 70.00 |
| **KEGG** | 12,309 | 75.93 |
| **Swiss-Prot** | 15,492 | 95.57 |
| **TrEMBL** | 15,954 | 98.42 |
| **Annotated** | 15,972 | 98.53 |
| **Unannotated** | 238 | 1.47 |

**Table S9.** **Summary of alignment of quail and chicken genome**s

| **Quail** | **Chicken_top1_chr** | **Mapped length (bp)** | **Mapped coverage** | **Chicken_top2_chr** | **Mapped length (bp)** | **Mapped coverage** |
| --- | --- | --- | --- | --- | --- | --- |
| Chr 1 | 1 | 143,155,404 | 97.15% | Z | 1,055,394 | 0.72% |
| Chr 2 | 2 | 117,839,379 | 97.28% | 1 | 1,051,807 | 0.87% |
| Chr 3 | 3 | 85,765,094 | 97.39% | 1 | 692,245 | 0.79% |
| Chr 4 | 4 | 74,901,805 | 97.91% | 1 | 479,659 | 0.63% |
| Chr 5 | 5 | 45,288,130 | 98.38% | 1 | 219,452 | 0.48% |
| Chr 6 | 6 | 23,661,361 | 97.97% | 1 | 130,361 | 0.54% |
| Chr 7 | 7 | 22,685,613 | 98.11% | 1 | 115,558 | 0.50% |
| Chr 8 | 8 | 20,116,961 | 97.88% | 1 | 100,397 | 0.49% |
| Chr 9 | 9 | 14,717,711 | 97.63% | 1 | 83,395 | 0.55% |
| Chr 10 | 10 | 17,384,546 | 97.91% | 1 | 103,129 | 0.58% |
| Chr 11 | 11 | 16,955,788 | 98.38% | 1 | 55,264 | 0.32% |
| Chr 12 | 12 | 15,388,360 | 98.49% | Z | 47,659 | 0.31% |
| Chr 13 | 13 | 11,762,046 | 98.41% | 1 | 36,882 | 0.31% |
| Chr 14 | 14 | 12,270,986 | 94.85% | 1 | 166,610 | 1.29% |
| Chr 15 | 15 | 8,489,030 | 98.15% | 1 | 37,019 | 0.43% |
| Chr 17 | 17 | 8,518,025 | 97.20% | 8 | 159,023 | 1.81% |
| Chr 18 | 18 | 8,744,769 | 96.18% | 1 | 78,897 | 0.87% |
| Chr 19 | 19 | 8,076,394 | 96.80% | 1 | 65,709 | 0.79% |
| Chr 20 | 20 | 9,801,526 | 96.93% | Z | 70,364 | 0.70% |
| Chr 21 | 21 | 5,695,111 | 97.67% | 1 | 34,574 | 0.59% |
| Chr 22 | 22 | 3,076,208 | 96.97% | 1 | 22,546 | 0.71% |
| Chr 23 | 23 | 4,679,303 | 97.70% | 1 | 23,967 | 0.50% |
| Chr 24 | 24 | 5,366,338 | 97.20% | Z | 60,378 | 1.09% |
| Chr 25 | 25 | 862,210 | 96.06% | Z | 8,391 | 0.93% |
| Chr 26 | 26 | 3,419,531 | 95.22% | 1 | 39,048 | 1.09% |
| Chr 27 | 27 | 3,731,771 | 91.11% | 1 | 81,940 | 2.00% |
| Chr 28 | 28 | 3,345,899 | 94.26% | Z | 67,152 | 1.89% |
| Chr 29 | LGE22C19W28_E50C23 | 693,952 | 94.83% | 1 | 10,634 | 1.45% |
| Chr 30 | LGE64 | 276,372 | 56.25% | 1 | 61,769 | 12.57% |
| Chr Z | Z | 48,799,819 | 93.65% | 1 | 1,175,679 | 2.26% |

Note: Chicken_top1_chr and Chicken_top2_chr are the chromosomes in chicken that best match the quail chromosomes.

**Table S10. Inversions detected between the quail and chicken genomes**

| **Chr Pair** | **block, length: direction;** |
| --- | --- |
| chr 10_10 | 63592-155354,91763:1;164833-186544,21712:-1;188430-19267350,19078921:1 |
| chr11_11 | 22669-2458865,2436197:-1;2473608-18582430,16108823:1 |
| chr12_12 | 2-2710181,2710180:1;2790387-3691497,901111:-1;3712516-17793349,14080834:1 |
| chr13_13 | 21885-5758012,5736128:-1;5758862-6778491,1019630:1;6803351-6808462,5112:-1;6814907-11265774,4450868:1;11266156-13171674,1905519:-1;13171781-13193581,21801:1;13197927-13527289,329363:-1 |
| chr17_17 | 10930-7010860,6999931:1;7014396-7034557,20162:-1;7036041-9635365,2599325:1 |
| chr18_18 | 4365-4609320,4604956:1;4614979-8811559,4196581:-1;8813364-9623860,810497:1 |
| chr19_19 | 26080-42517,16438:-1;80663-9386287,9305625:1 |
| chr1_1 | 39495-9119349,9079855:1;9119836-9125414,5579:-1;9129918-33607695,24477778:1;33790064-33796870,6807:-1;33799180-34812750,1013571:1;34825468-34834897,9430:-1;34836932-45474919,10637988:1;45973131-47115855,1142725:-1;47243486-50697745,3454260:1;50698669-50706248,7580:-1;50707092-51111973,404882:1;51125308-54204798,3079491:-1;54219609-54225659,6051:1;54227030-57310276,3083247:-1;57313721-108569634,51255914:1;108572523-108593176,20654:-1;108595520-109301334,705815:1;109305445-109329310,23866:-1;109464003-113634037,4170035:1;114795777-114828079,32303:-1;117018857-176973079,59954223:1;176974916-176981545,6630:-1;176982769-179249888,2267120:1;179258585-179268940,10356:-1;179270492-179494315,223824:1 |
| chr20_20 | 68-9073010,9072943:1;9079445-10122297,1042853:-1;10123910-10141894,17985:1;10142372-10397447,255076:-1;10404999-11818997,1413999:1 |
| chr21_21 | 4670-4465449,4460780:1;4513855-6426582,1912728:-1 |
| chr22_22 | 17054-885458,868405:-1;1351686-2062522,710837:1;2173063-2734354,561292:-1;2771979-4234014,1462036:1 |
| chr25_25 | 9120-98522,89403:1;749186-1341669,592484:-1;1553839-2112056,558218:1 |
| chr27_27 | 2373-315426,313054:1;327200-332550,5351:-1;370567-386961,16395:1;415086-420170,5085:-1;426932-5048563,4621632:1 |
| chr29_LGE22C19W28_E50C23 | 110318-1044081,933764:1;1189745-1252445,62701:-1;1624459-1922298,297840:1 |
| chr2_2 | 757-10426,9670:1;32219-70013,37795:-1;148211-43609753,43461543:1;43780978-69820750,26039773:-1;69826937-69832150,5214:1;69832910-75555275,5722366:-1;75559038-75928084,369047:1;75933508-75940764,7257:-1;75947940-77094962,1147023:1;77104937-79554783,2449847:-1;79561376-86144884,6583509:1;86151982-86157242,5261:-1;86158680-89860704,3702025:1;89863963-89933711,69749:-1;89937115-138169954,48232840:1 |
| chr30_LGE64 | 126656-182602,55947:1;232376-310517,78142:-1;342708-522064,179357:1 |
| chr3_3 | 34377-1211472,1177096:1;1215226-5246322,4031097:-1;5412172-7070773,1658602:1;7117712-8812244,1694533:-1;8815794-8821221,5428:1;8823657-9272901,449245:-1;10800977-31900666,21099690:1;31903496-31937045,33550:-1;31937575-102390827,70453253:1 |
| chr4_4 | 4926-4301426,4296501:1;4302136-10103055,5800920:-1;10116547-79394750,69278204:1;79396178-79435834,39657:-1;79436646-84098010,4661365:1 |
| chr5_5 | 11450-2785407,2773958:1;4025365-6628926,2603562:-1;6773522-7126354,352833:1;7148807-10617939,3469133:-1;12276270-16401059,4124790:1;16428416-17952260,1523845:-1;18053300-51016227,32962928:1;51021552-51032134,10583:-1;51033043-55357476,4324434:1 |
| chr6_6 | 7-3996532,3996526:1;4945405-9097552,4152148:-1;9107639-24898508,15790870:1;24898954-24904098,5145:-1;24904846-30040885,5136040:1 |
| chr7_7 | 1887284-5341325,3454042:1;5343016-5370057,27042:-1;5371692-19097172,13725481:1;19102079-28158172,9056094:-1;28165441-31784518,3619078:1;31798283-31980903,182621:-1;32041707-35159408,3117702:1 |
| chr8_8 | 84-4525050,4524967:1;4526506-4643921,117416:-1;4644634-4719421,74788:1;4724933-7593137,2868205:-1;7599650-23853704,16254055:1 |
| chr9_9 | 13378-2439868,2426491:-1;2444152-16201218,13757067:1 |
| chrZ_Z | 7209-5350468,5343260:1;5365594-5381731,16138:-1;5397926-11156893,5758968:1;11175090-11188330,13241:-1;11190688-15019060,3828373:1;15020520-15027410,6891:-1;15030160-19651644,4621485:1;19653203-19658772,5570:-1;19663024-20533624,870601:1;20533887-20539895,6009:-1;20545001-20671157,126157:1;20671182-20679654,8473:-1;20684604-22985812,2301209:1;23047504-24024635,977132:-1;24049135-37684245,13635111:1;37774721-38046771,272051:-1;38056949-39541129,1484181:1;39546350-39560233,13884:-1;39560866-48197230,8636365:1;48206623-48212211,5589:-1;48222680-50214362,1991683:1;50716294-50762643,46350:-1;50926286-54010559,3084274:1;54010691-54021587,10897:-1;54022213-60659388,6637176:1 |

**Table S11. Phenotypic information for 10 wild and 21 domesticated quail samples used for population structure analysis**

| **Sample** | **Code** | **Sex** | **Description** | **Plumage Color** | **source** |
| --- | --- | --- | --- | --- | --- |
| wildquail-1603-M | Wild-M-1603 | **Male** | **Wild quail** | **Maroon** | **Anyang, Henan** |
| wildquail-1604-M | Wild-M-1604 | **Male** | **Wild quail** | **Maroon** | **Anyang, Henan** |
| wildquail-1606-M | Wild-M-1606 | **Male** | **Wild quail** | **Maroon** | **Anyang, Henan** |
| wildquail-1614-M | Wild-M-1614 | **Male** | **Wild quail** | **Maroon** | **Anyang, Henan** |
| wildquail-wu-M | Wild-M-wu | **Male** | **Wild quail** | **Maroon** | **Weishan, Shandong** |
| wildquail-8601-F | Wild-F-8601 | **Female** | **Wild quail** | **Maroon** | **Anyang, Henan** |
| wildquail-8607-F | Wild-F-8607 | **Female** | **Wild quail** | **Maroon** | **Anyang, Henan** |
| wildquail-8609-F | Wild-F-8609 | **Female** | **Wild quail** | **Maroon** | **Anyang, Henan** |
| wildquail-8646-F | Wild-F-8646 | **Female** | **Wild quail** | **Maroon** | **Weishan, Shandong** |
| wildquail-8648-F | Wild-F-8648 | **Female** | **Wild quail** | **Maroon** | **Weishan, Shandong** |
| eggquail-E-117283-F | Egg-FE-117283 | **Female** | **Egg-type quail** | **Maroon** | **Nanchang, Jiangxi** |
| eggquail-E-59172-F | Egg-FE-59172 | **Female** | **Egg-type quail** | **Maroon** | **Anlu, Hubei** |
| eggquail-E-59179-F | Egg-FE-59179 | **Female** | **Egg-type quail** | **Maroon** | **Anlu, Hubei** |
| eggquail-E-59180-F | Egg-FE-59180 | **Female** | **Egg-type quail** | **Maroon** | **Anlu, Hubei** |
| eggquail-E-59181-F | Egg-FE-59181 | **Female** | **Egg-type quail** | **Maroon** | **Anlu, Hubei** |
| eggquail-E-89270-F | Egg-FE-89270 | **Female** | **Egg-type quail** | **Maroon** | **Anlu, Hubei** |
| eggquail-L-107363-F | Egg-FL-107363 | **Female** | **Egg-type quail** | **Maroon** | **Wuhan, Hubei** |
| eggquail-L-107372-F | Egg-FL-107372 | **Female** | **Egg-type quail** | **Maroon** | **Wuhan, Hubei** |
| eggquail-L-107374-F | Egg-FL-107374 | **Female** | **Egg-type quail** | **Maroon** | **Wuhan, Hubei** |
| eggquail-L-107398-F | Egg-FL-107398 | **Female** | **Egg-type quail** | **Maroon** | **Wuhan, Hubei** |
| eggquail-L-59163-F | Egg-FL-59163 | **Female** | **Egg-type quail** | **Maroon** | **Anlu, Hubei** |
| meatquail-1262522-M | Meat-M-1262522 | **Male** | **Meat-type quail** | **-** | **Shanghai** |
| meatquail-1262526-M | Meat-M-1262526 | **Male** | **Meat-type quail** | **-** | **Shanghai** |
| meatquail-1262575-M | Meat-M-1262575 | **Male** | **Meat-type quail** | **-** | **Jining, Shandong** |
| meatquail-1262576-M | Meat-M-1262576 | **Male** | **Meat-type quail** | **-** | **Jining, Shandong** |
| meatquail-1262578-M | Meat-M-1262578 | **Male** | **Meat-type quail** | **-** | **Jining, Shandong** |
| meatquail-1262530-F | Meat-F-1262530 | **Female** | **Meat-type quail** | **-** | **Shanghai** |
| meatquail-1262536-F | Meat-F-1262536 | **Female** | **Meat-type quail** | **-** | **Shanghai** |
| meatquail-1262550-F | Meat-F-1262550 | **Female** | **Meat-type quail** | **-** | **Jining, Shandong** |
| meatquail-1262577-F | Meat-F-1262577 | **Female** | **Meat-type quail** | **-** | **Jining, Shandong** |
| meatquail-1262587-F | Meat-F-1262587 | **Female** | **Meat-type quail** | **-** | **Jining, Shandong** |

**Table S12. Sequencing and reads mapping statistics for 31 samples**

| **Sample** | **# reads** | **Mappng ratio (%)** | **Depth (X)** | **Coverage (%)** |
| --- | --- | --- | --- | --- |
| eggquail-E-117283-F | 168,922,442 | 77.20 | 19.27 | 96.69 |
| eggquail-E-59172-F | 167,227,942 | 79.27 | 19.70 | 96.80 |
| eggquail-E-59179-F | 158,396,478 | 80.22 | 18.87 | 96.79 |
| eggquail-E-59180-F | 166,876,306 | 75.49 | 18.59 | 96.73 |
| eggquail-E-59181-F | 194,659,852 | 78.20 | 22.43 | 96.84 |
| eggquail-E-89270-F | 140,499,720 | 74.67 | 15.54 | 96.50 |
| eggquail-L-107363-F | 166,368,488 | 77.92 | 19.16 | 96.69 |
| eggquail-L-107372-F | 158,303,532 | 79.88 | 18.80 | 96.65 |
| eggquail-L-107374-F | 172,442,546 | 77.69 | 19.79 | 96.76 |
| eggquail-L-107398-F | 164,092,316 | 77.86 | 18.83 | 96.75 |
| eggquail-L-59163-F | 147,091,876 | 77.38 | 16.83 | 96.74 |
| meatquail-1262522-M | 178,503,698 | 79.84 | 21.09 | 96.81 |
| meatquail-1262526-M | 202,021,564 | 77.01 | 22.94 | 96.87 |
| meatquail-1262530-F | 174,574,300 | 78.60 | 20.24 | 96.71 |
| meatquail-1262536-F | 159,233,752 | 77.77 | 18.34 | 96.63 |
| meatquail-1262550-F | 251,642,742 | 78.58 | 29.08 | 96.83 |
| meatquail-1262575-M | 159,673,154 | 79.67 | 18.87 | 96.70 |
| meatquail-1262576-M | 158,890,678 | 79.31 | 18.63 | 96.73 |
| meatquail-1262577-F | 144,665,940 | 79.53 | 17.12 | 96.63 |
| meatquail-1262578-M | 157,206,324 | 79.66 | 18.59 | 96.69 |
| meatquail-1262587-F | 154,314,760 | 79.21 | 18.13 | 96.63 |
| wildquail-1603-M | 149,307,000 | 78.69 | 17.43 | 96.68 |
| wildquail-1604-M | 167,116,254 | 76.52 | 18.90 | 96.74 |
| wildquail-1606-M | 159,326,098 | 75.87 | 17.91 | 96.70 |
| wildquail-1614-M | 153,089,470 | 78.33 | 17.80 | 96.72 |
| wildquail-8601-F | 167,822,004 | 74.24 | 18.38 | 96.76 |
| wildquail-8607-F | 152,996,548 | 76.33 | 17.29 | 96.67 |
| wildquail-8609-F | 161,815,692 | 75.68 | 18.18 | 96.73 |
| wildquail-8646-F | 149,335,240 | 78.75 | 17.46 | 96.74 |
| wildquail-8648-F | 164,763,488 | 74.77 | 18.16 | 96.78 |
| wildquail-wu-M | 165,966,562 | 78.25 | 19.26 | 96.79 |
| **Average** | **165,714,412** | **77.82** | **19.08** | **96.72** |

**Table S18.** **Comparison of gene family copy numbers of genes in the GnRH signaling pathway between quail, chicken, turkey, duck, pigeon and Chinese alligator as an outgroup**

| **Gene family** | **Quail** | **Chicken** | **Turkey** | **Duck** | **Pigeon** | **Chinese alligator** |
| --- | --- | --- | --- | --- | --- | --- |
| Protein kinase domain | 2 | 1 | 1 | 1 | 1 | 1 |
| Lysophospholipase, catalytic domain | 2 | 1 | 1 | 1 | 2 | 1 |
| EF-hand domain | 2 | 1 | 1 | 1 | 1 | 1 |
| Adenylate cyclase, conserved domain | 2 | 1 | 1 | 1 | 2 | 1 |
| G-protein alpha subunit, group S | 3 | 2 | 2 | 2 | 2 | 1 |
| Phospholipase A2 | 8 | 7 | 7 | 4 | 5 | 1 |
| Gonadotropin-releasing hormone 1 | 2 | 1 | 1 | 1 | 1 | 1 |

Note: quail (*Coturnix japonica*), chicken (*Gallus gallus*), turkey (*Meleagris gallopavo*), duck *Anas platyrhynchos*, pigeon (*Columba livia*) and Chinese alligator (*Alligator sinensis*).

**Table S20. Plumage color information for 40 quail samples used for association analysis**

| **No.** | **Code** | **Sex** | **Plumage Color** | |
| --- | --- | --- | --- | --- |
| 1 | YAM-1 | Male | | Yellow |
| 2 | CAF-1 | Female | | **Maroon** |
| 3 | YYF-1 | Female | | Yellow |
| 4 | CYM-1 | Male | | **Maroon** |
| 5 | YAM-2 | Male | | Yellow |
| 6 | CAF-2 | Female | | **Maroon** |
| 7 | YYF-2 | Female | | Yellow |
| 8 | CYM-2 | Male | | **Maroon** |
| 9 | YAM-3 | Male | | Yellow |
| 10 | CAF-3 | Female | | **Maroon** |
| 11 | YYF-3 | Female | | Yellow |
| 12 | CYM-3 | Male | | **Maroon** |
| 13 | YAM-4 | Male | | Yellow |
| 14 | CAF-4 | Female | | **Maroon** |
| 15 | YYF-4 | Female | | Yellow |
| 16 | CYM-4 | Male | | **Maroon** |
| 17 | YAM-5 | Male | | Yellow |
| 18 | CAF-5 | Female | | **Maroon** |
| 19 | YYF-5 | Female | | Yellow |
| 20 | CYM-5 | Male | | Chestnut |
| 21 | YAM-6 | Male | | Yellow |
| 22 | CAF-6 | Female | | **Maroon** |
| 23 | YYF-6 | Female | | Yellow |
| 24 | CYM-6 | Male | | **Maroon** |
| 25 | YAM-7 | Male | | Yellow |
| 26 | CAF-7 | Female | | **Maroon** |
| 27 | YYF-7 | Female | | Yellow |
| 28 | CYM-7 | Male | | **Maroon** |
| 29 | YAM-8 | Male | | Yellow |
| 30 | CAF-8 | Female | | **Maroon** |
| 31 | YYF-8 | Female | | Yellow |
| 32 | CYM-8 | Male | | **Maroon** |
| 33 | YAM-9 | Male | | Yellow |
| 34 | CAF-9 | Female | | **Maroon** |
| 35 | YYF-9 | Female | | Yellow |
| 36 | CYM-9 | Male | | **Maroon** |
| 37 | YAM-10 | Male | | Yellow |
| 38 | CAF-10 | Female | | **Maroon** |
| 39 | YYF-10 | Female | | Yellow |
| 40 | CYM-10 | Male | | **Maroon** |

**Table S21. SNP sites most significantly associated with plumage color in quail**

| **Chr.** | **SNP** | **Pos.** | **A1** | **F_A**^a^ **aa^a^** | **F_U**^a^ **^a^** | **A2** | ***P*** | **Adjusted**^b^ ***P* ^b^** |
| --- | --- | --- | --- | --- | --- | --- | --- | --- |
| 1 | 1:61102026 | 61102026 | T | 0.725 | 0.088 | C | 3.585e^-8^ | 0.028 |
| Z | Z:23173971 | 23173971 | T | 0.800 | 0.107 | C | 3.994e^-8^ | 0.019 |

^a^ Frequency of this allele in cases/controls.

^b^ Bonferroni single-step adjusted *P*-values

**Table S22. Primer sequences of transcripts clone and qPCR for *CCDC171***

| **Intent** | **type** | **Primer name** | **Primer sequnces** | **Products length (cds range)**  **Bp** |
| --- | --- | --- | --- | --- |
| **clone** | **Yellow** | Y-F1 | 5’-GCTGCAGCAGGAAGCGGCA-3’ | 1291 (1-1291) |
|  |  | Y-R1 | 5’-ACAAGTGCCTGCTGTCCGCT-3’ |  |
|  |  | Y-F2 | 5’-CGCAGAGAATTACAAGAG-3’ | 1681 (1151-2831) |
|  |  | Y-R2 | 5’-TGTGAAGTCCACGAGCCAGCC-3’ |  |
|  |  | Y-F3 | 5’-CCCAGTCTTCTAGTTCTCTC-3’ | 1152 (2439-3665) |
|  |  | Y-R3 | 5’-GGGGGGGGTGTTCTTTCA-3’ |  |
|  | **Maroon** | C-F1 | 5’-ATGATTGAAATAATGAATTTG-3’ | 1566 (1-1566) |
|  |  | C-R1 | 5’-AAGCTTCTGGATTTCATTTTC-3’ |  |
|  |  | C-F2 | 5’-GCCAATGCCCATAAAGAG-3’ | 729 (1411-2139) |
|  |  | C-R2 | 5’-ACATGATCGGCCATAGAGAGG-3’ |  |
|  |  | C-F3 | 5’-GGAGCTTCAGCGGAGCCAGG-3’ | 917 (1809-2725) |
|  |  | C-R3 | 5’-TCTGTATCAAGGAGTTTTTC-3’ |  |
|  |  | C-F4 | 5’-GGAATTCGTTGTGTTGAAGC-3’ | 1121 (2470-3590) |
|  |  | C-R4 | 5’-GGGGGGGGTGTTCTTTCACC-3’ |  |
| **qPCR** |  | F1 | 5’-CAGCAGGCACTTGTAAGGAC-3’ | 113 |
|  |  | R1 | 5’-AACCTCATTAGATGTGTCTTCCAG-3’ |  |
|  |  | F2 | 5’-TGCCCAGTCTTCTAGTTCTCTC-3’ | 123 |
|  |  | R2 | 5’-ATTCCTTCCTCGTTGTGTCTTG-3’ |  |
