# Supplementary material for: Population genomic data reveal genes related to important traits of quail
Source: Gigascience. 2018 May 11;7(5):giy049. doi: 10.1093/gigascience/giy049 (PMC5961004; doi:10.1093/gigascience/giy049)
Supplement: Response_to_Reviewer_Comments_Original_Submission.pdf [file giy049_response_to_reviewer_comments_original_submission.pdf]

Author's Response To Reviewer Comments

Close

We also attached a docx file named "Respond to Reviewers.docx" which shows respond details for all Reviewers.

Responses for Reviewers' comments:

Reviewer #1: I have previously reviewed this manuscript for Nature Communications, and felt it was an excellent stuc work and is well thought-out and executed. Previously my main points regarding the manuscript comprised of: 1) analysis and a recommendation to use GEMMA or GEMMAX to control for population substructure 2) Further det the study, in particular the origins of the different wild and domesticated breeds. 3) The conclusion for the selecti two separate domestication events occurred due to a lack of shared sweeps (when these could be a sign of more rec differentially selected in the broiler and layer breeds). 4) Gene expansion evidence indicating that GnRH is causal quail. All of these points have been well addressed. The authors have used GEMMAX and provided more detai the assertion that this is proof of two independent domestication events (they could maybe mention 'improvement s the gene expansion pertaining to GnRH and sexual maturity, I couldn't find the details of the numbers of different ge expanding, which I would like to see (as it allows the reader to evaluate how these genes in particular were targeted) that GnRH is strongly controlling time to sexual maturity and they have also mentioned that no selective sweeps over The language could be tidied up a little in places (the usual refrain about getting a naïve reader to look through it wit very happy with the manuscript (in fact, I was surprised it wasn't accepted by Nature Communications as I didn't feel have no hesitation in endorsing it.

RE: First of all, the authors are all thankful of your appreciation for our work. A very short sexual maturity time is the compared birds. It means that the quail has obtained this attribute after the split of quails from other birds. This is th (Table S18) and positively selected genes in the GnRH pathway; some functional studies also support our hypothesis. any sexual maturity-related genes in this study. The major reason is that we compared selective regions among three have this unique trait, we cannot detect selective sweep regions for sexual maturity.

Reviewer #2: In this manuscript, the authors present a chromosome level assembly of the quail genome, and RNA-seq address a large variety of questions regarding the relationship of the quail to other galliformes, and identify genes related to various traits such as immunity, sexual maturity and plumage. This is very interesting work, containi of useful results. Line 61-65: references required. Or the authors need to clarify that these statements are ane papers, see references 14-16. Line 78: The sentence "Some of the key avian … were included[25]." is a poorly for authors are referring to here. I assume that the authors mean that the phylogeny and genetic relationships between well resolved.

RE: We have revised the sentence. The revised sentence is as follows: "The phylogeny and genetic relationships for so (i.e., the chicken, turkey, and Japanese quail) are not well resolved". See line 78-79.

Line 118: What do the authors mean by consistency between the previously released genome and their assembly but a little more information on the statistics used to test for consistency between the genomes would be useful.

RE: We compared these two genomes at whole genome level to detect all synteny blocks which were defined as cons supplemented the statistic method and it is as follows "Here we employed Lastz (v1.02.00) with parameters 'T=2 C=2 compare 30 chromosomes of the two genomes and then calculated the synteny ratio of them after excluding 'N' base relationships of blocks more than 5 kb". See legend of Figure S2.

Line 123-126: The sentence "In addition … peak-laying (LP) (Table S4)." does not make any sense. It looks like the sentence.

RE: We have revised the sentence. The revised sentence is as follows: "To assess the integrity of protein-coding gene transcripts assembled from RNA-Seq data sampled from the hypothalamus and ovary of three stages of quail maturit peak-laying (LP)), were mapped to the assembled genome (Table S4) and ~96.33% of total complete BUSCO genes ca S5)." See line 124-129.

Line 178-181: Number of SNPs identified seems very high implying a very high nucleotide diversity. Were the SNPs id

so all 31 samples together - or were the SNPs identified using single sample callers. There are caveats in both these m
spurious SNP calls.
RE: The method of joint variant calling, the tool of "UnifiedGenotyper" in GATK3, was used in the combined populatio
identified in all 31 samples together. The high depth of sequencing data could help to reduce the probability of spurio
in the GWAS section which indicates the method is reliable (line 414-419).

Line 181-183: I was a bit confused by what the authors meant by the different number of SNPs in the different popula
among the ~22 million total SNPs that were polymorphic in each of the three populations?
RE: Firstly, joint variant calling was used in the combined population so we could get the total number of SNPs. Secon
were polymorphic in each of the three subpopulations (meat-type, egg-type and wild) so we could get the number of
181-186.

Line 189-191: The differences in the ratio of non-synonymous to synonymous SNPs in the quail compared to the two
due to differences in sample size, quality of SNPs, the filtering criteria etc. This might indicate that this ratio is not nec
these other two birds.
RE: Just as you said, the differences of N/S ratio in quail population compared to chicken and turkey were influenced
the sequencing depth and more SNPs were detected. There was no strong support for the difference in N/S ratio betw
turkey. See line 190-193.

Line 195-216: This entire paragraph needs to be rewritten for clarity. It was very difficult to follow the sequence of lo
authors. Firstly, they mention that their estimates of θw are different in the wild quail compared to the two domestic
estimate of π is similar in the three populations. Their conclusion from this is that diversity in the wild quail populatio
populations are pretty homogeneous within themselves. This conclusion can be tested by using dimension reduction
AMOVA to see if the within and between group variance support this conclusion. The Z chromosome section talking a
the entire Z chromosome in the egg type quail is a bit hard to believe and looks like it might be artefactual, since all th
low levels of diversity in all three populations.
RE: The entire paragraph has been rewritten for clarity as suggested. Some of the conclusions were also updated due
coverage (from 3.5X to 19X). The conclusions in this part are much more concise this time. Here, we emphasize that t
quail as compared to the domesticated populations. The phenomenon that genetic diversity on chromosome Z was r
and that this was a common finding according to previous studies. See line 196-203.

This implies that the differences on Z have to be driven by non genic regions. I would need strong evidence to bel
selection across the entire chromosome for this population. An alternative hypothesis for the reduced diversity on th
egg type. The egg type is also the population with the lowest number of SNPs, indicating that possibly the effective po
both the wild and meat type, and this in turn means that the Ne of the Z chromosome is lower in these and since Z is
reduced Ne than the autosomes, this effect is still detectable on the Z. Finally, Tajima's D is a statistic based on the di
confused that the autosomes do not show any signal in the D statistic, as the authors previously mention that the θw
for the egg type and the meat type, while the π are similar. A clarification would be immensely helpful.
RE: This is a similar or related question to the one above. We have rewritten these sections.

Lines 227-229: These sentences talk about the the clustering of the wild quail samples on PC1 and PC2. The autho
the homogeneity in wild quail. This is completely false. The first PC is separating out the wild from the domestics and
and meat type. In both these cases, they do not load SNPs that explain the variance within the wild quails. This close
expected, especially given the low sample sizes in all three groups.
RE: We rewrote the PCA plot result to make it clearer. We deleted the descriptions of variance within in the wild qua
that this is not much related to the PCA plot. See line 204-214.

Lines 234-241: Interpretation of the ADMIXTURE results. Firstly, given the admixture plots, it is clear that the admixtu
31 samples are not stable for the cases from K=4 to K=7. The ancestry clusters appear and disappear and clusters fou
Ks. Interpreting any of these results is in appropriate and does not lead to valid inferences. Therefore, I suggest that t
to K=2 and K=3 which are very nice results. The slight mixing shown between egg and meat type also explains the spr
RE: Interpretations of the ADMIXTURE results have been updated by restricting interpretations to K=2 and K=3 as the

Lines 245-248: I am not sure I follow how the quick LD decay is indicative of heterogeneity in populations resultin

recombination. Firstly, LD computed here is r2, which is not an indicator of recombination rates or frequencies, since
frequencies of the two variants under consideration. Second, D or D' is a much better candidate for measuring recom
pairwise r2 in windows is highly affected by low frequency variants which by default have low r2 with other variants.
frequency would help in this regard. One other thing which might be causing trouble in this and other analyses might
additional column indicating the number of singletons and the proportion of private variants in each of the three pop
1.
RE: We have revised this conclusion. See line 228-238.

Line 265: "complete bottleneck" is a meaningless term. I think the authors mean to say strong bottleneck.
RE: We agree with your opinion and have replaced the word "complete" with "strong". See line 250.

Lines 283-284: If the authors mean many large scale selection events in the genome, then I agree, but the senten
sounds like the authors claim multiple selective sweeps in the same loci, which is not possible to tell from the data th
RE: Yes, we were trying to say there were many large-scale selection events in the genome. We rephrased this part to

Lines 300-301: We do not need to invoke 2 domestications, but can say that the quail was probably domesticated
probably crowing), and the egg and meat traits were selected upon independently from this domesticated from start
RE: That's a very good suggestion. We rephrased the description by stating "It is worth noting that egg-type and mea
sweep regions when compared with wild quails (Figure S11), meaning that egg-type/meat-type quails might have bee
domestication or that there were two separate domestication events in quails." See line 447-451.

Lines 302-304: Did the authors perform RNA seq or similar to measure gene expression? The following paragraph (
expression but about different non-synonymous SNPs in domestics compared to the wild. Although this is very nice a
differential expression.
RE: Unfortunately, we did not have these RNAseq data.

Lines 335-340: The authors mention that higher FSHβ leads to reduction in mature FSH, which is required for sexu
this FSHβ is higher in quail compared to chicken, which would imply later maturity in quail. I might have missed some
RE：We revised the description in this sentence. FSHβ is the major gene for controlling hormone synthesis rate. It mo
higher hormone synthesis rate, and the hormone level could determine the sex maturity in animals. The revised sent
FSHβ gene is the rate-limiting step for in hormone synthesis which is required for ovarian folliculogenesis in females a
conjunction with testosterone." See line 322-324.

Lines 411-415: The additional quails added here come from different populations, which have been shown to the
to know what kind of corrections were performed for this, since the lower p-value might be an artifact of population
RE：Yes, the p-value was the corrected one by Bonferroni method.

Language related comments: Line 27: "That only one of them shared" should read "That only one of them is sh
Done.
Line 29: "marron" should reads "maroon" I think.
Done

Line 60: "than that do in males" should read "than males".
Done.

I stopped here, but there are lots of English related errors in the manuscript, and in its current form, it cannot be
although the content deserves the platform.
RE: We invited native English speakers to polish the manuscript to resolve any language issues.

Reviewer #3: In the Wu et al manuscript, the authors describe the sequencing of a Japanese quail at 238 fold c
with an N50 contig size of 27.9 kb and an N50 scaffold size of 1.8 Mb. This assembly is less complete than a separate
of 511 kb and N50 scaffold size of 3 Mb (1). The authors used their assembly to build a tree for the Phasianidae famil
(Japanese quail, chicken, and turkey). Their results were consistent with the Hackett et al study (2) and several other
that the quail and chicken are more closely related to each other than they are to the turkey. It is unclear how impac
confirm previous studies.
RE: Although some investigations have been done to explore the phylogenetic relationship, there are many conflicts a
for the first time, the use of whole genome data to perform the phylogenetic analysis as this can better confirm the r
The authors compared their quail assembly to 10 other bird genomes, along with a Chinese alligator as an outgroup.

the divergence times of the phasianidae. Including an aniseriform was a critical inclusion of the analysis. Figure S7 sh
phylogenic trees was a critical part of the study. Space permitting, the authors may consider moving this figure to th
RE: We agree with your opinion. However, there is not enough space to move this figure to the main text.

The authors then undertook the task of analyzing the genetic diversity in 3 distinct quail for a total of 31 individua approximately 10 individuals at an average of 3.5X coverage. However, there was no listing of the coverage for each individuals under 1X coverage.

RE: We have increased the average sequencing coverage to ~19X of the genome. All data have been updated accordi

The authors did a nice analysis on gene families that potentially lead to early sexual maturity. The quail reaches n It would be interesting to see a follow up study in a separate manuscript that compared the genes families found to t that reach sexual maturity early, like some parrots. Likewise, it would be good to compare these gene families to oth sexual maturity later in life.

RE: Thank you for your opinion. We plan to do more research in the future and expect a good result.

For the GNRH1 gene, they found one extra copy compared to the other genomes they studied. They based this o (VFLLLLWENLPPVQAGKAREGWVRLVGEKRQESLVHMWQSQLCITLGYVQEYDYINLDAPAVTMSLLTELKP) of the protein shov this "unique" peptide sequence, I was able to find it in chicken using a blast search (see reviewer fig 1 below). I also r PLCB4 fig S15. See my reviewer fig 2 below. It would be good if the authors could explain this. I also saw a similar ob XP_015148438) (not shown). This could be due to outdated annotations.

RE:

(1) For GNRH1 gene (Figure S14)

On the one hand, the data we used were from Ensembl release80 database and in the database, there is only one cop have checked the NCBI sequence ID X69491.1 supplied by you. The positive match may only occur at the nucleic acid as below(MEKSRKILVGVLLFTASVAICLAQHWSYGLQPGGKRNAENLVESFQEIANEMESLGEGQKAECPGSYQHPRLSDLKETMAS of X69491.1 is also as below. The sequence did not contain
VFLLLLWENLPPVQAGKAREGWVRLVGEKRQESLVHMWQSQLCITLGYVQEYDYINLDAPAVTMSLLTELKP.

G.gallus gene for gonadotrophin releasing hormone I
GenBank: X69491.1
FASTA Graphics
Go to:
LOCUS X69491 6282 bp DNA linear VRT 14-NOV-2006
DEFINITION G.gallus gene for gonadotrophin releasing hormone I.
ACCESSION X69491
VERSION X69491.1
KEYWORDS gonadotropin-releasing hormone; gonadotropin-releasing hormone-I;
neuroendocrine protein.
SOURCE Gallus gallus (chicken)
ORGANISM Gallus gallus
Eukaryota; Metazoa; Chordata; Craniata; Vertebrata; Euteleostomi;
Archelosauria; Archosauria; Dinosauria; Saurischia; Theropoda;
Coelurosauria; Aves; Neognathae; Galloanserae; Galliformes;
Phasianidae; Phasianinae; Gallus.
REFERENCE 1 (bases 1 to 6281)
AUTHORS Dunn,I.C., Chen,Y., Hook,C., Sharp,P.J. and Sang,H.M.
TITLE Characterization of the chicken preprogonadotrophin-releasing
hormone-I gene
JOURNAL J. Mol. Endocrinol. 11 (1), 19-29 (1993)
PUBMED 7902095
REFERENCE 2
AUTHORS Dunn,I.
TITLE Direct Submission
JOURNAL Submitted (30-NOV-1992) I. Dunn, Institute of Animal Physiology
and, Genetics Research, AFRC, Roslin, Midlothian EH 9PS, Scotland,

UK
REMARK revised by [3] MAT
REFERENCE 3 (bases 1 to 6282)
AUTHORS Dunn,I.
TITLE Direct Submission
JOURNAL Submitted (31-MAY-1994) I. Dunn, Institute of Animal Physiology
and, Genetics Research, AFRC, Roslin, Midlothian EH 9PS, Scotland,
UK
COMMENT On Jun 4, 1994 this sequence version replaced gi:311611.
FEATURES Location/Qualifiers
source 1..6282
/organism="Gallus gallus"
/mol_type="genomic DNA"
/strain="white leghorn"
/db_xref="taxon:9031"
/clone="lambda52"
/sub_clone="EDP1"
/clone_lib="SAU3AI digest genomic library in EMBL3"
/dev_stage="adult"
mRNA join(2996..3047,3485..3626,4296..4391,5899..5998)
exon 2996..3047
/number=1
intron 3048..3484
/number=1
exon 3485..3626
/number=2
gene order(3486..3626,4296..4391,5899..5940)
/gene="cGnRH-I"
CDS join(3486..3626,4296..4391,5899..5940)
/gene="cGnRH-I"
/codon_start=1
/product="chicken gonadotrophin releasing hormone-I"
/protein_id="CAA49246.1"
/db_xref="GOA:P37042"
/db_xref="InterPro:IPR002012"
/db_xref="InterPro:IPR004079"
/db_xref="InterPro:IPR019792"
/db_xref="UniProtKB/Swiss-Prot:P37042"
/translation="MEKSRKILVGVLLFTASVAICLAQHWSYGLQPGGKRNAENLVES
FQEIANEMESLGEGQKAECPGSYQHPRLSDLKETMASLIEGEARRKEI"
intron 3627..4295
/gene="cGnRH-I"
/number=2
exon 4296..4391
/gene="cGnRH-I"
/number=3
intron 4392..5898
/gene="cGnRH-I"
/number=3
exon 5899..5998
/number=4

(2) For PLCB4 gene (Figure S15)

We have checked the Ensembl database release 84/87/89/90/91 and for example, for the duck genome, we did not f including sequence "KKAVEDSEQEN". All the version of annotation information is as below.

>ENSAPLP00000013480.1 pep scaffold:BGI_duck_1.0:KB742503.1:690475:780114:1 gene:ENSAPLG00000013577.1 tr .1 gene_biotype:protein_coding transcript_biotype:protein_coding gene_symbol:PLCB4 description: phospholipase C [Source:HGNCSymbol;Acc:HGNC:9059]

MAKPYEFNWQKPVPSFMQDGAVFDRYEEESFVFESNCLFQVDEFGFFLSWKSEGKEGQVLECSLINSVRFGAIPKDPKILTALEAVGKS
IAKQWVEGLGSIIHNFRANNVSPMTCLKKHWMKLAFLTNTNGKIPVRSITRTFASGKTEK
VIFQALKELGLPSGKNDEIEPAAFTYDKFYELTQKICPRTDIEDLFKKINGDKTDYLTVD
QLVSFLNEHQRDPRLNEILFPFYDTKRAMQIIETYEPDEDLKSKGLISSDGFCRYLMSDE
NAPVFLDRLELYQEMDHPLAHYFISSSHNTYLTGRQFGGKSSVEMYRQVLLAGCRCVED
CWDGKGEDQEPIITHGKAMCTDILFKDVIQAIKETAFVTSEYPVILSFENHCSKYQQYKM
SKYCEDLFGDLLLKQPLETHPLDPGKALPSPNDLKRKILIKNKRLKPEVEKKQLDALKKM
MEAGETAAPVNILEDDNEEEIESADQEEEAHPEYKFGSDLTVDDYCQKEAVAISVKKGLV
TVEDEQAWMASYKYVGATTNIHPYLSTMVNYAQPVKFQGFDVAEERNIHHNMSSFNESG
LGYLKTHAIEFVNYNKRQMSRIYPKGGRVDSSNYMPQIFWNAGCQMVSLNYQTPDLAML
NQGKFEYNGSCGYLLKPDFMRRPDRTFDPFSETPVDGVIAATCSVQVISGQFLSDKKIGT
YVEVDMYGLPTDTIRKEFRTRMVMNNGLNPVYNEESFVFRKVILPDLAVLRIAVYDDNNK
LIGQRILPLDGLQAGYRHISLRNEGNKPLSLPTVFCNIVLKTYVPDGFGDIVDALSDPKK
FLSITEKRADQMRAMGIETSDIADVPNDTSKNDRKGKANNAKANVTPQSSSELRPSTTAG
FGSGTDAKKGIDLIAQVKIEDLKQMKAYIKHLKKQQKELNALKKKHAKEHSAMQKLHCTQ
IDKVVAQYDKEKVSYEKMLEKAIKKKGGSNCVELKKETEIKIQALTSDHKSKVKEIVAQH
TKEWSEMINTHSAEEQEIRDLHLNQQCELLKKLLINAHEQQTQQLKFSHDRESKEMRANQ
AKISMENSKAISQDKSIKNKAERERRVRELNSSNTKKFLEERKRLAMKQSKEMDQLKKVQ
LEHLEVLEKQNEQKQLLKSCHAVSQTQGKGDAADGEAGSRDGSQASNSGLKPQHVN

(3) For ITPR1 gene (Figure S16)

We thought it was due to different version of annotation. We checked Ensembl database release 80/84/90/91and dic "MEGREPPPRGSKPE". Only in Ensembl database release 87 did we find it.

The authors then did a nice analysis of genes from the immune system. This was very insightful. Were these extr IsoSeq data?

RE: We used RNAseq data to validate three gene families of Klf4 / ITCH / Foxa2 and the average coverage of all genes 90.32%, 91.33% and 88.23% respectively, which indicated that the expansion of gene families was real.

The authors also did an analysis of the 40 genomes they sequenced for correlation with plumage color. They four authors then added additional sequence data from 100 maroon and 100 yellow quails. This was an excellent additior

Suggestions * Include NCBI genome IDs for other bird genomes used, since multiple versions are available

RE: We have supplied the corresponding version ID or websites of all used genomes. See at Methods section "Gene e

* I would have not done the saker falcon since it is redundant. The Peregrine falcon would have been enough. I That being said, I don't think it is necessary to redo the analysis with budgie.

RE: At the initial time of analysis, we added the budgie. However, we deleted it ultimately because we identified too this bird.

* For figures S14, S15, S16, S17 were chicken IsoSeq reads used for analysis?

RE: In these figures, we used the RNAseq of quail for analysis. We did not use chicken IsoSeq reads.

* BUSCO or CEGMA should have been used to determine completeness of all the individual quail genomes.

RE: BUSCO analysis were done for assembled genome, transcriptome and gene peptide set. We did not assemble all i only mapped the resequencing reads to the reference genome. We supplied BUSCO for the reference genome (Table quail reads to the reference genome.

* Table S10 should be expanded to include the sequence coverage of all the individuals or an separate suppleme coverage and BUSCO summary.

RE: This is a similar question to the one above and we have supplied data in Table S11 and S12.

* An average coverage of 3.5X of the entire data set is not very telling. Please report the standard deviation for mapped reads for each individual.

RE: We have increased the sequencing coverage to about 19X and supplied the mapping information (Table S12).

* Based on the text in 217-220, I was expecting a phylogenetic tree in the supplement, rather than just a table.

RE: Figure 3a was the exact phylogenetic tree

* In figures S14-S17, the authors may want to consider using a lighter shade coloring for easier reading.

RE: We agree with your opinion while we thought the current forms of these figures were ok for reading.

Minor comments * From what population was the reference genome sequenced? Maybe I missed this?

RE: We have supplemented the information in methods section "Genome sequencing and assembly".

* Nice decision to include a good balance of both males and females in the study. * In table S6 you could ad used for the homology based portion of the annotations. Was IsoSeq data used? IsoSeq data is available to several c

RE: We only used RNAseq data.

* In reference to table S8, was an alignment of the quail to the turkey done and not shown?

RE: Sorry, we did not carry out the alignment with turkey because our focus was mainly on the comparison with chick

Major criticisms * It is unclear why the authors did not use one of the other quail genomes assemblies from opposed to creating their own. The main reference genome is not better than the recently assembly genomes. They higher coverage of the individuals in the population.

RE: More de novo assembly provides a chance for pan-genome analysis. Our new quail genome has been assembled more powerful when conducting GWAS-like study than the previously available genome.

* 3.5X coverage is not enough coverage. The studies that I have read in the past two years have all had at least 8- references to articles from journals showing that 3.5X coverage is enough. As genomics is a fast moving field, please 2015.

RE: We have increased the sequencing coverage to about 19X to improve the accuracy of SNP calling.

* BUSCO or CEGMA was not shown to validate the quality of the reference genome. This should be in the suppl previously sequenced quail genomes. I have heard that you will report this separately. However, I would have liked analysis in this paper.

RE: We have supplied the BUSCO analysis of the reference genome. See Table S5.

Conclusion Overall, the authors did a very good job analyzing the population data they had. However, I do not thi another reference genome added much value to the paper since the quail genome was already sequenced using a si the literature over the past couple years, I think studies should aim for a minimum of 10X coverage for resequencing. study is appropriate for Gigascience. However, if the editors feel 3.5X coverage is enough now or if the authors can s 3.5X or below coverage, then I will reconsider my opinion.

RE: We have increased the sequencing coverage to about 19X to improve the accuracy of SNPs calling. All data have b based on the updated data.

References 1. https://www.ncbi.nlm.nih.gov/assembly/GCA_001577835.1#/st 2. Hackett SJ, Ki Braun MJ, Chojnowski JL, Cox WA, Han KL, Harshman J, Huddleston CJ, Marks BD, Miglia KJ, Moore WS, Sheldon FH, S phylogenomic study of birds reveals their evolutionary history. Science. 2008 Jun 27;320(5884):1763-8. doi: 10.1126 Kimball RT, Braun EL, Liang B, Zhang Z (2013) Assessing Phylogenetic Relationships among Galliformes: A Multigene F Sampling in Phasianidae. PLoS ONE 8(5): e64312. 4. Sibley CG, Ahlquist JE (1990) Phylogeny and Classification of New Haven: Yale University Press. 5. Kimball and Braun (2014), Does more sequence data improve estimates of g radiation using a complete data matrix. PeerJ 2:e361; DOI 10.7717/peerj.361 6. https://www.illumina.com/scienc coverage.html 7. David Sims, Ian Sudbery, Nicholas E. Ilott, Andreas Heger & Chris P. Ponting. Sequencing depth a genomic analyses. Nature Reviews Genetics 15, 121-132 (2014) 8. Burri R, Nater A, Kawakami T, Mugal CF, Olaso Garamszegi LZ, Hogner S, Moreno J, Qvarnström A, Ružić M, Sæther SA, Sætre GP, Török J, Ellegren H. Linked selectio drive the evolution of the genomic landscape of differentiation across the speciation continuum of Ficedula flycatche 65.

Close
