# Supplementary material for: Population genomic data reveal genes related to important traits of quail
Source: Gigascience. 2018 May 11;7(5):giy049. doi: 10.1093/gigascience/giy049 (PMC5961004; doi:10.1093/gigascience/giy049)
Supplement: Reviewer_1_Report_(Original_Submission) -- Dominic Wright — 10/24/2017 Reviewed [file giy049_reviewer_1_report_(original_submission).pdf]

Reviewer Report

Title: Population genomic data reveal genes related to important traits of quail

Version: Original Submission **Date:** 10/24/2017

Reviewer name: Dominic Wright

Reviewer Comments to Author:

I have previously reviewed this manuscript for Nature Communications, and felt it was an excellent study then. It represents a large body of work and is well thought-out and executed. Previously my main points regarding the manuscript comprised of:

1) The use of PLINK for the association analysis and a recommendation to use GEMMA or GEMMAX to control for population substructure

2) Further details on the origin of the birds used for the study, in particular the origins of the different wild and domesticated breeds.

3) The conclusion for the selective sweep mapping experiments that two separate domestication events occurred due to a lack of shared sweeps (when these could be a sign of more recent 'improvement' genes being differentially selected in the broiler and layer breeds).

4) Gene expansion evidence indicating that GnRH is causal to the onset of sexual maturity in the quail.

All of these points have been well addressed. The authors have used GEMMAX and provided more details on the cross. They have toned down the assertion that this is proof of two independent domestication events (they could maybe mention 'improvement selection', but this is very minor). For the gene expansion pertaining to GnRH and sexual maturity, I couldn't find the details of the numbers of different gene families that were identified as expanding, which I would like to see (as it allows the reader to evaluate how these genes in particular were targeted). They have toned down the assertion that GnRH is strongly controlling time to sexual maturity and they have also mentioned that no selective sweeps overlapped the GnRH genes as a caveat. The language could be tidied up a little in places (the usual refrain about getting a naïve reader to look through it with fresh eyes to weed these out).

I am very happy with the manuscript (in fact, I was surprised it wasn't accepted by Nature Communications as I didn't feel that any of these were fatal flaws), and have no hesitation in endorsing it.

Sincerely
Dominic Wright

Methods

Are the methods appropriate to the aims of the study, are they well described, and are necessary controls included? Yes

Conclusions

Are the conclusions adequately supported by the data shown? Yes

Reporting Standards

Does the manuscript adhere to the journal's guidelines on minimum standards of reporting? Yes

Choose an item.

Statistics

Are you able to assess all statistics in the manuscript, including the appropriateness of statistical tests used? Yes, and I have assessed the statistics in my report.

Quality of Written English

Please indicate the quality of language in the manuscript: Needs some language corrections before being published

Declaration of Competing Interests

Please complete a declaration of competing interests, considering the following questions:

- Have you in the past five years received reimbursements, fees, funding, or salary from an organisation that may in any way gain or lose financially from the publication of this manuscript, either now or in the future?
- Do you hold any stocks or shares in an organisation that may in any way gain or lose financially from the publication of this manuscript, either now or in the future?
- Do you hold or are you currently applying for any patents relating to the content of the manuscript?
- Have you received reimbursements, fees, funding, or salary from an organization that holds or has applied for patents relating to the content of the manuscript?
- Do you have any other financial competing interests?
- Do you have any non-financial competing interests in relation to this paper?

If you can answer no to all of the above, write 'I declare that I have no competing interests' below. If your reply is yes to any, please give details below.

I declare that I have no competing interests

I agree to the open peer review policy of the journal. I understand that my name will be included on my report to the authors and, if the manuscript is accepted for publication, my named report including any attachments I upload will be posted on the website along with the authors' responses. I agree for my report to be made available under an Open Access Creative Commons CC-BY license (http://creativecommons.org/licenses/by/4.0/). I understand that any comments which I do not wish to be included in my named report can be included as confidential comments to the editors, which will not be published.

I agree to the open peer review policy of the journal

To further support our reviewers, we have joined with Publons, where you can gain additional credit to further highlight your hard work (see: https://publons.com/journal/530/gigascience). On publication of this paper, your review will be automatically added to Publons, you can then choose whether or not to claim your Publons credit. I understand this statement.

Yes Choose an item.
