# Supplementary material for: Population genomic data reveal genes related to important traits of quail
Source: Gigascience. 2018 May 11;7(5):giy049. doi: 10.1093/gigascience/giy049 (PMC5961004; doi:10.1093/gigascience/giy049)
Supplement: Reviewer_2_Report_(Original_Submission) -- Shyam Gopalakrishnan — 10/31/2017 Reviewed [file giy049_reviewer_2_report_(original_submission).pdf]

Reviewer Report

Title: Population genomic data reveal genes related to important traits of quail

Version: Original Submission **Date:** 10/31/2017

Reviewer name: Shyam Gopalakrishnan

Reviewer Comments to Author:

In this manuscript, the authors present a chromosome level assembly of the quail genome, and through comparative genomics and RNA-seq address a large variety of questions regarding the relationship of the quail to other galliformes, and identify several important and interesting genes related to various traits such as immunity, sexual maturity and plumage. This is very interesting work, containing well designed experiments and a lot of useful results.

Line 61-65: references required. Or the authors need to clarify that these statements are anecdotal.

Line 78: The sentence "Some of the key avian ... were included[25]." is a poorly formed sentence. I am not sure what the authors are referring to here. I assume that the authors mean that the phylogeny and genetic relationships between the three avian model systems are not well resolved.

Line 118: What do the authors mean by consistency between the previously released genome and their assembly? From Fig S2, it looks like it is synteny, but a little more information on the statistics used to test for consistency between the genomes would be useful.

Line 123-126: The sentence "In addition ... peak-laying (LP) (Table S4)." does not make any sense. It looks like the authors are missing some part of the sentence.

Line 178-181: Number of SNPs identified seems very high implying a very high nucleotide diversity. Were the SNPs identified in the combined populations - so all 31 samples together - or were the SNPs identified using single sample callers. There are caveats in both these methods that could lead to a lot of spurious SNP calls.

Line 181-183: I was a bit confused by what the authors meant by the different number of SNPs in the different populations. Were these the number of SNPs among the ~22 million total SNPs that were polymorphic in each of the three populations?

Line 189-191: The differences in the ratio of non-synonymous to synonymous SNPs in the quail compared to the two other galliforms mentioned might be due to differences in sample size, quality of SNPs, the filtering criteria etc. This might indicate that this ratio is not necessarily significantly different from these other two birds.

Line 195-216: This entire paragraph needs to be rewritten for clarity. It was very difficult to follow the sequence of logical conclusions being laid out by the authors. Firstly, they mention that their estimates of θw are different in the wild quail compared to the two domestic forms for the autosomes but that their estimate of π is similar in the three populations. Their conclusion from this is that diversity in the wild quail population is quite high but that the three populations are pretty homogeneous within themselves. This conclusion can be tested by using dimension reduction methods such as PCA, MDS or even AMOVA to see if the within and between group variance support this conclusion. The Z chromosome section talking about higher selective pressure across the entire Z chromosome in the egg type quail is a bit hard to believe and looks like it might be artefactual, since all the genic regions on the Z have similarly low levels of diversity in

all three populations. This implies that the differences on Z have to be driven by non genic regions. I would need strong evidence to believe that non-genic regions are driving selection across the entire chromosome for this population. An alternative hypothesis for the reduced diversity on the Z might lie in the demography of the egg type. The egg type is also the population with the lowest number of SNPs, indicating that possibly the effective population size of this type is lower than both the wild and meat type, and this in turn means that the Ne of the Z chromosome is lower in these and since Z is more adversely affected by the reduced Ne than the autosomes, this effect is still detectable on the Z. Finally, Tajima's D is a statistic based on the differences in θw and π, I am a bit confused that the autosomes do not show any signal in the D statistic, as the authors previously mention that the θw for the wild quail is different than that for the egg type and the meat type, while the π are similar. A clarification would be immensely helpful.

Lines 227-229: These sentences talk about the the clustering of the wild quail samples on PC1 and PC2. The authors claim that the clustering indicates the homogeneity in wild quail. This is completely false. The first PC is separating out the wild from the domestics and the second PC is separating the egg and meat type. In both these cases, they do not load SNPs that explain the variance within the wild quails. This close clustering of wild quail is to be expected, especially given the low sample sizes in all three groups.

Lines 234-241: Interpretation of the ADMIXTURE results. Firstly, given the admixture plots, it is clear that the admixture proportions being calculated for the 31 samples are not stable for the cases from K=4 to K=7. The ancestry clusters appear and disappear and clusters found at lower Ks are not found at higher Ks. Interpreting any of these results is in appropriate and does not lead to valid inferences. Therefore, I suggest that the authors restrict their interpretations to K=2 and K=3 which are very nice results. The slight mixing shown between egg and meat type also explains the spread along PC2 in the PCA plot.

Lines 245-248: I am not sure I follow how the quick LD decay is indicative of heterogeneity in populations resulting from gene segregation and high recombination. Firstly, LD computed here is r2, which is not an indicator of recombination rates or frequencies, since it depends on the matching of allele frequencies of the two variants under consideration. Second, D or D' is a much better candidate for measuring recombination. Third, measure mean pairwise r2 in windows is highly affected by low frequency variants which by default have low r2 with other variants. A lower bound on the minor allele frequency would help in this regard. One other thing which might be causing trouble in this and other analyses might be the excess of singletons. An additional column indicating the number of singletons and the proportion of private variants in each of the three populations would be very useful in table 1.

Line 265: "complete bottleneck" is a meaningless term. I think the authors mean to say strong bottleneck.

Lines 283-284: If the authors mean many large scale selection events in the genome, then I agree, but the sentence might need rephrasing. It now sounds like the authors claim multiple selective sweeps in the same loci, which is not possible to tell from the data they use.

Lines 300-301: We do not need to invoke 2 domestications, but can say that the quail was probably domesticated for other traits (as mentioned earlier probably crowing), and the egg and meat traits were selected upon independently from this domesticated from starting from the early 20th century.

Lines 302-304: Did the authors perform RNA seq or similar to measure gene expression? The following paragraph (Lines 305-) does not say anything about expression but about different non-synonymous SNPs in domestics compared to the wild. Although this is very nice analysis, it has nothing to do with differential expression.

Lines 335-340: The authors mention that higher FSHβ leads to reduction in mature FSH, which is required for sexual maturity. But they also mention that this FSHβ is higher in quail compared to chicken, which would imply later maturity in quail. I might have missed something but this is counterintuitive.

Lines 411-415: The additional quails added here come from different populations, which have been shown to the different before, so it would be useful to know what kind of corrections were performed for this, since the lower p-value might be an artifact of population structure in the samples.

Language related comments:
Line 27: "That only one of them shared" should read "That only one of them is shared"
Line 29: "marron" should reads "maroon" I think.
Line 60: "than that do in males" should read "than males".
I stopped here, but there are lots of English related errors in the manuscript, and in its current form, it cannot be published due to the language issues, although the content deserves the platform.

Methods

Are the methods appropriate to the aims of the study, are they well described, and are necessary controls included? Yes

Conclusions

Are the conclusions adequately supported by the data shown? Yes

Reporting Standards

Does the manuscript adhere to the journal's guidelines on minimum standards of reporting? Yes

Choose an item.

Statistics

Are you able to assess all statistics in the manuscript, including the appropriateness of statistical tests used? Yes, and I have assessed the statistics in my report.

Quality of Written English

Please indicate the quality of language in the manuscript: Not suitable for publication unless extensively edited

Declaration of Competing Interests

Please complete a declaration of competing interests, considering the following questions:

- Have you in the past five years received reimbursements, fees, funding, or salary from an organisation that may in any way gain or lose financially from the publication of this manuscript, either now or in the future?

- Do you hold any stocks or shares in an organisation that may in any way gain or lose financially from the publication of this manuscript, either now or in the future?

- Do you hold or are you currently applying for any patents relating to the content of the manuscript?
- Have you received reimbursements, fees, funding, or salary from an organization that holds or has applied for patents relating to the content of the manuscript?
- Do you have any other financial competing interests?
- Do you have any non-financial competing interests in relation to this paper?

If you can answer no to all of the above, write 'I declare that I have no competing interests' below. If your reply is yes to any, please give details below.

I declare that I have no competing interests

I agree to the open peer review policy of the journal. I understand that my name will be included on my report to the authors and, if the manuscript is accepted for publication, my named report including any attachments I upload will be posted on the website along with the authors' responses. I agree for my report to be made available under an Open Access Creative Commons CC-BY license (http://creativecommons.org/licenses/by/4.0/). I understand that any comments which I do not wish to be included in my named report can be included as confidential comments to the editors, which will not be published.

I agree to the open peer review policy of the journal

To further support our reviewers, we have joined with Publons, where you can gain additional credit to further highlight your hard work (see: https://publons.com/journal/530/gigascience). On publication of this paper, your review will be automatically added to Publons, you can then choose whether or not to claim your Publons credit. I understand this statement.

Yes Choose an item.
