# Supplementary material for: Population genomic data reveal genes related to important traits of quail
Source: Gigascience. 2018 May 11;7(5):giy049. doi: 10.1093/gigascience/giy049 (PMC5961004; doi:10.1093/gigascience/giy049)
Supplement: Reviewer_3_Report_(Original_Submission) -- Jason Travis Howard — 12/3/2017 Reviewed [file giy049_reviewer_3_report_(original_submission).pdf]

Reviewer Report

Title: Population genomic data reveal genes related to important traits of quail

Version: Original Submission **Date:** 12/3/2017

Reviewer name: Jason Travis Howard

Reviewer Comments to Author:

In the Wu et al manuscript, the authors describe the sequencing of a Japanese quail at 238 fold coverage. They generated an assembly with an N50 contig size of 27.9 kb and an N50 scaffold size of 1.8 Mb. This assembly is less complete than a separate quail assembly with an N50 contig size of 511 kb and N50 scaffold size of 3 Mb (1). The authors used their assembly to build a tree for the Phasianidae family using three different species (Japanese quail, chicken, and turkey). Their results were consistent with the Hackett et al study (2) and several other studies (3-5). These reports all show that the quail and chicken are more closely related to each other than they are to the turkey. It is unclear how impactful these results are other than they confirm previous studies.

The authors compared their quail assembly to 10 other bird genomes, along with a Chinese alligator as an outgroup. The authors did a good job resolving the divergence times of the phasianidae. Including an aniseriform was a critical inclusion of the analysis. Figure S7 showing the MrBayes and PhyML based phylogenic trees was a critical part of the study. Space permitting, the authors may consider moving this figure to the main text.

The authors then undertook the task of analyzing the genetic diversity in 3 distinct quail for a total of 31 individuals. The authors sequenced approximately 10 individuals at an average of 3.5X coverage. However, there was no listing of the coverage for each individual. There could be some individuals under 1X coverage. The authors did a nice analysis on gene families that potentially lead to early sexual maturity. The quail reaches maturity rapidly relative to other birds. It would be interesting to see a follow up study in a separate manuscript that compared the genes families found to the same gene families in other birds that reach sexual maturity early, like some parrots. Likewise, it would be good to compare these gene families to other birds, beyond chickens that reach sexual maturity later in life.

For the GNRH1 gene, they found one extra copy compared to the other genomes they studied. They based this on a peptide region (VFLLLLWENLPPVQAGKAREGWVRLVGEKRQESLVHMWQSQLCITLGYVQEYDYINLDAPAVTMSLLTELKP) of the protein shown in figure S14. However, when I used this "unique" peptide sequence, I was able to find it in chicken using a blast search (see reviewer fig 1 below). I also noticed a similar observation in the PLCB4 fig S15. See my reviewer fig 2 below. It would be good if the authors could explain this. I also saw a similar observation in figure S16 (chicken protein XP_015148438) (not shown). This could be due to outdated annotations.

The authors then did a nice analysis of genes from the immune system. This was very insightful. Were these extra copies validated with RNAseq or IsoSeq data?
The authors also did an analysis of the 40 genomes they sequenced for correlation with plumage color. They found the CCDC171 as a candidate. The authors then added additional sequence data from 100 maroon and 100 yellow quails. This was an excellent addition to the study.

Suggestions
* Include NCBI genome IDs for other bird genomes used, since multiple versions are available

* I would have not done the saker falcon since it is redundant. The Peregrine falcon would have been enough. I would have added the budgie instead. That being said, I don't think it is necessary to redo the analysis with budgie.
* For figures S14, S15, S16, S17 were chicken IsoSeq reads used for analysis?
* BUSCO or CEGMA should have been used to determine completeness of all the individual quail genomes.
* Table S10 should be expanded to include the sequence coverage of all the individuals or an separate supplemental table should be created to include coverage and BUSCO summary.
* An average coverage of 3.5X of the entire data set is not very telling. Please report the standard deviation for the entire dataset. Also include the % mapped reads for each individual.
* Based on the text in 217-220, I was expecting a phylogenetic tree in the supplement, rather than just a table.
* In figures S14-S17, the authors may want to consider using a lighter shade coloring for easier reading.

Minor comments
* From what population was the reference genome sequenced? Maybe I missed this?
* Nice decision to include a good balance of both males and females in the study.
* In table S6 you could add more information on the gene sets used for the homology based portion of the annotations. Was IsoSeq data used? IsoSeq data is available to several of the species listed in table S6.
* In reference to table S8, was an alignment of the quail to the turkey done and not shown?

Major criticisms
* It is unclear why the authors did not use one of the other quail genomes assemblies from other groups' efforts for mapping as opposed to creating their own. The main reference genome is not better than the recently assembly genomes. They could have spent their resources on higher coverage of the individuals in the population.
* 3.5X coverage is not enough coverage. The studies that I have read in the past two years have all had at least 8-10X coverage (6-8). Please give references to articles from journals showing that 3.5X coverage is enough. As genomics is a fast moving field, please only include articles published since 2015.
* BUSCO or CEGMA was not shown to validate the quality of the reference genome. This should be in the supplement along with a comparison to the previously sequenced quail genomes. I have heard that you will report this separately. However, I would have liked to have seen the BUSCO or CEGMA analysis in this paper.

Conclusion
Overall, the authors did a very good job analyzing the population data they had. However, I do not think the high coverage sequencing of another reference genome added much value to the paper since the quail genome was already sequenced using a similar sequencing strategy (1). Based on the literature over the past couple years, I think studies should aim for a minimum of 10X coverage for resequencing. Given this opinion, I do not think this study is appropriate for Gigascience. However, if the editors feel 3.5X coverage is enough now or if the authors can show a number of recent papers with 3.5X or below coverage, then I will reconsider my opinion.

References

1. https://www.ncbi.nlm.nih.gov/assembly/GCA_001577835.1#/st
2. Hackett SJ, Kimball RT, Reddy S, Bowie RC, Braun EL, Braun MJ, Chojnowski JL, Cox WA, Han KL, Harshman J, Huddleston CJ, Marks BD, Miglia KJ, Moore WS, Sheldon FH, Steadman DW, Witt CC, Yuri T. A phylogenomic study of birds reveals their evolutionary history. Science. 2008 Jun 27;320(5884):1763-8. doi: 10.1126/science.1157704.
3. Wang N, Kimball RT, Braun EL, Liang B, Zhang Z (2013) Assessing Phylogenetic Relationships among Galliformes: A Multigene Phylogeny with Expanded Taxon Sampling in Phasianidae. PLoS ONE 8(5): e64312.

4. Sibley CG, Ahlquist JE (1990) Phylogeny and Classification of Birds: A Study in Molecular Evolution. New Haven: Yale University Press.
5. Kimball and Braun (2014), Does more sequence data improve estimates of galliform phylogeny? Analyses of a rapid radiation using a complete data matrix. PeerJ 2:e361; DOI 10.7717/peerj.361
6. https://www.illumina.com/science/education/sequencing-coverage.html
7. David Sims, Ian Sudbery, Nicholas E. Ilott, Andreas Heger & Chris P. Ponting. Sequencing depth and coverage: key considerations in genomic analyses. Nature Reviews Genetics 15, 121-132 (2014)
8. Burri R, Nater A, Kawakami T, Mugal CF, Olason P, Smeds L, Suh A, Dutoit L, Bureš S, Garamszegi LZ, Hogner S, Moreno J, Qvarnström A, Ružić M, Sæther SA, Sætre GP, Török J, Ellegren H. Linked selection and recombination rate variation drive the evolution of the genomic landscape of differentiation across the speciation continuum of Ficedula flycatchers. Genome Res. 2015 Nov;25(11):1656-65.

.

Methods

Are the methods appropriate to the aims of the study, are they well described, and are necessary controls included? Yes

Conclusions

Are the conclusions adequately supported by the data shown? Yes

Reporting Standards

Does the manuscript adhere to the journal's guidelines on minimum standards of reporting? Yes

Choose an item.

Statistics

Are you able to assess all statistics in the manuscript, including the appropriateness of statistical tests used? No, and I do not feel adequately qualified to assess the statistics.

Quality of Written English

Please indicate the quality of language in the manuscript: Acceptable

Declaration of Competing Interests

Please complete a declaration of competing interests, considering the following questions:

- Have you in the past five years received reimbursements, fees, funding, or salary from an organisation that may in any way gain or lose financially from the publication of this manuscript, either now or in the future?

- Do you hold any stocks or shares in an organisation that may in any way gain or lose financially from the publication of this manuscript, either now or in the future?

- Do you hold or are you currently applying for any patents relating to the content of the manuscript?
- Have you received reimbursements, fees, funding, or salary from an organization that holds or has applied for patents relating to the content of the manuscript?
- Do you have any other financial competing interests?
- Do you have any non-financial competing interests in relation to this paper?

If you can answer no to all of the above, write 'I declare that I have no competing interests' below. If your reply is yes to any, please give details below.

I declare that I have no competing interests.

I agree to the open peer review policy of the journal. I understand that my name will be included on my report to the authors and, if the manuscript is accepted for publication, my named report including any attachments I upload will be posted on the website along with the authors' responses. I agree for my report to be made available under an Open Access Creative Commons CC-BY license (http://creativecommons.org/licenses/by/4.0/). I understand that any comments which I do not wish to be included in my named report can be included as confidential comments to the editors, which will not be published.

I agree to the open peer review policy of the journal

To further support our reviewers, we have joined with Publons, where you can gain additional credit to further highlight your hard work (see: https://publons.com/journal/530/gigascience). On publication of this paper, your review will be automatically added to Publons, you can then choose whether or not to claim your Publons credit. I understand this statement.

Yes Choose an item.
